# Supplementary material for: HEARTSVG: a fast and accurate method for identifying spatially variable genes in large-scale spatial transcriptomics
Source: Nat Commun. 2024 Jul 7;15:5700. doi: 10.1038/s41467-024-49846-1 (PMC11228050; doi:10.1038/s41467-024-49846-1)
Supplement: Supplementary file 1 — Supplementary Information [file 41467_2024_49846_MOESM1_ESM.pdf]

**Supplementary of HEARTSVG**

**Xin Yuan, Yanran Ma, Ruitian Gao, Shuya Cui, Yifan Wang, Botao Fa, Shiyang Ma, Ting Wei,**

**Shuangge Ma, and Zhangsheng Yu**

# 1. Simulation Results

## 1.1 Simulation 1 additional figures

We used $F_1$ score, recall, precision, specificity, and false positive (FP) to comprehensively evaluate the performance of HEARTSVG and SpatialDE, SPARK, and SPARK-X. All simulation settings were illustrated in the two sections, Simulation and Methods. Each simulation scenario has 50 replications. Simulation datasets were generated by varying the sample size (from 1500 to 50000), ZINB parameters ($size = 0.15,\ 0.5,\ 1.5, mu = 0.5, 5, 15$), and SVG percentages (hotspot, streak =5%, gradient=15%). The paper presented the simulation scenario with ZINB parameters ($size = 0.5, mu = 0.5$). The complete simulation results were shown in this supplementary.

**All simulation results of the hotspot pattern**

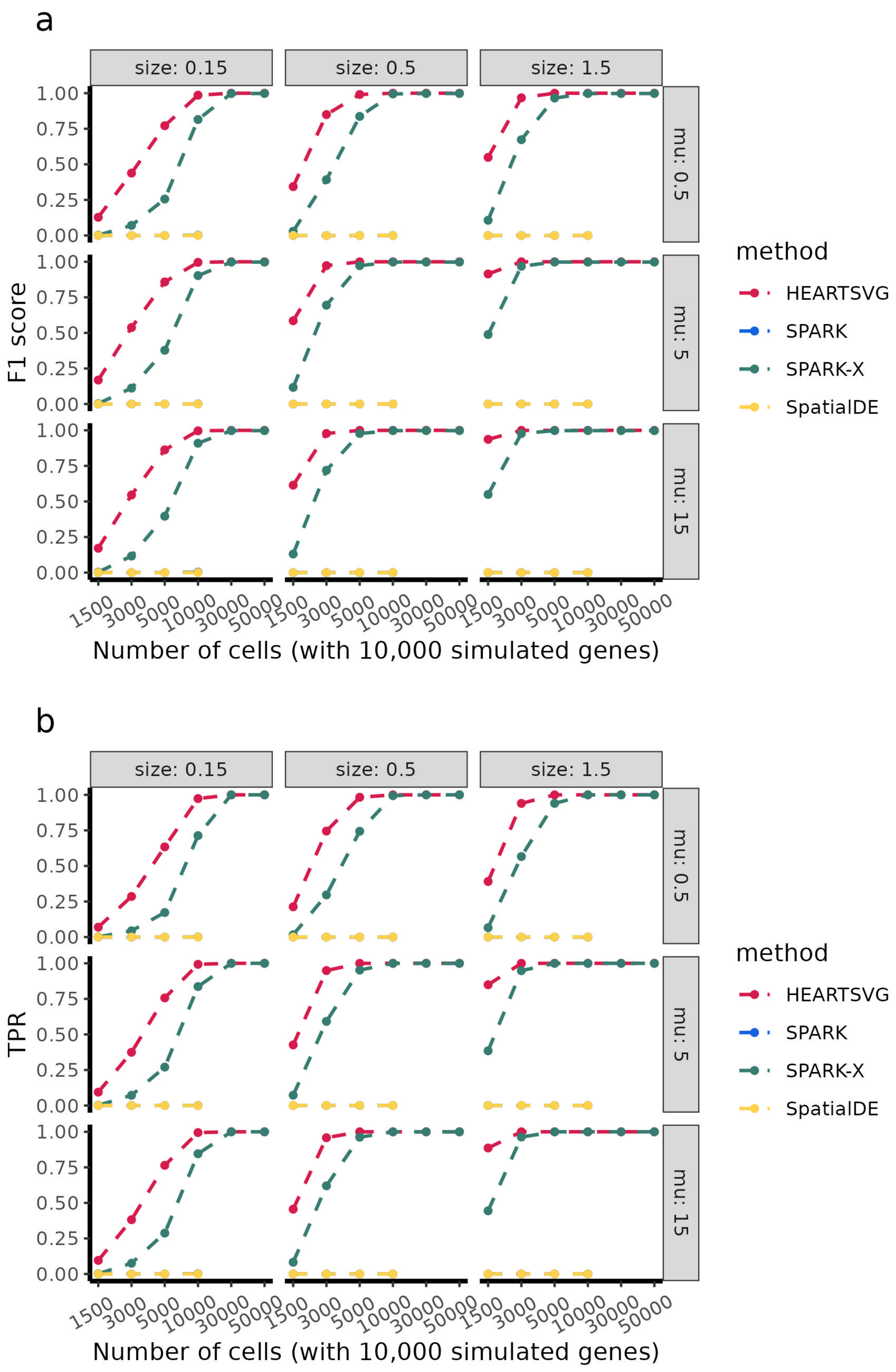

c

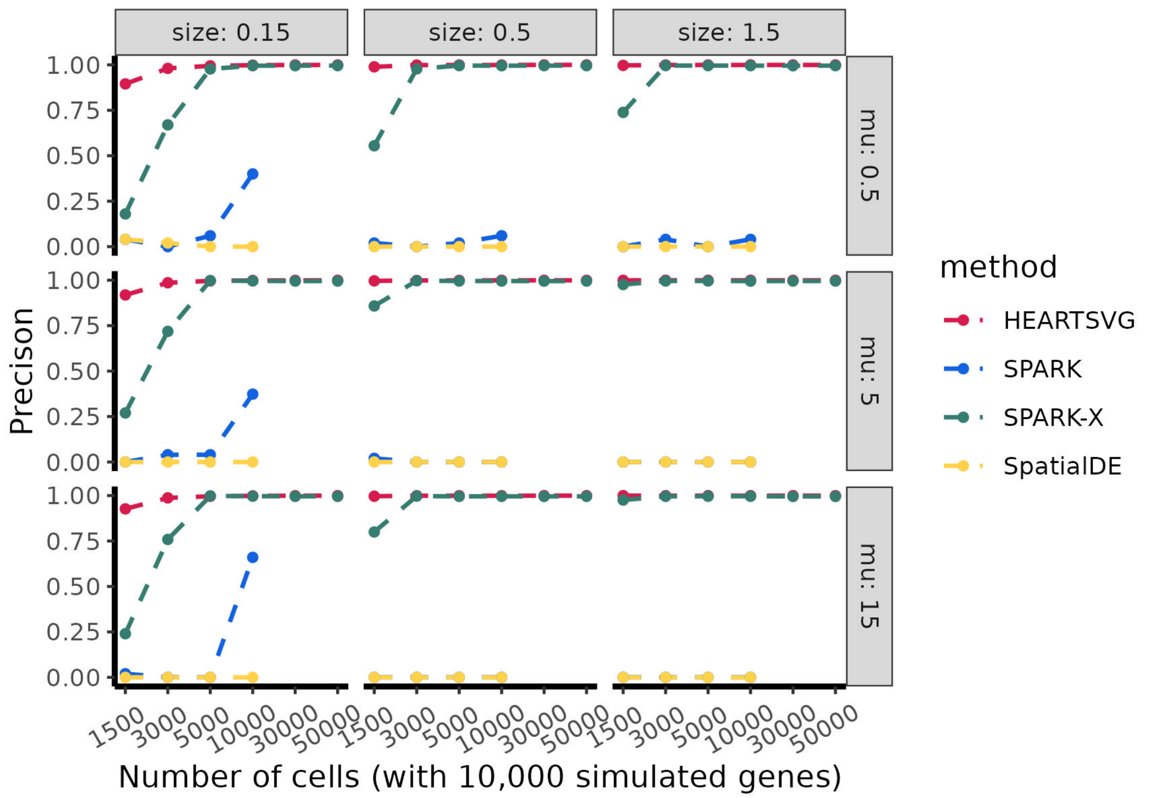


d

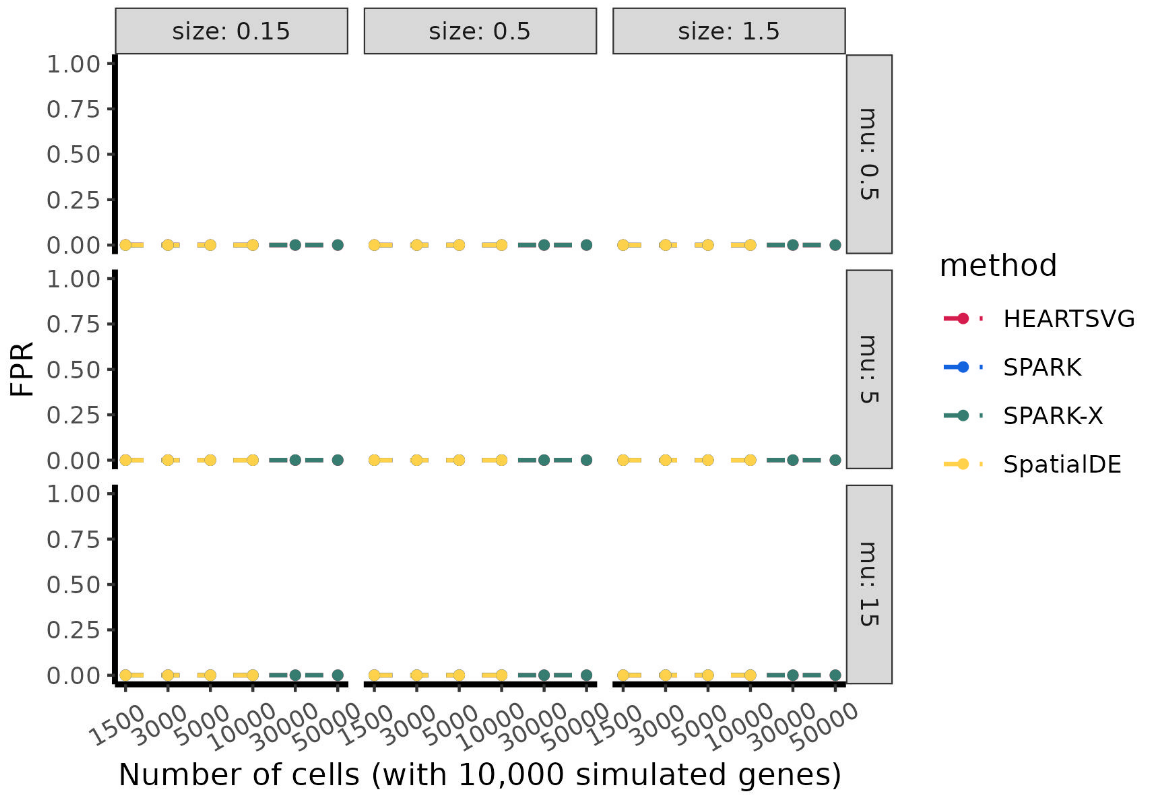


**Figure S1** Simulation results of hotspot pattern. **a,** F1 score, **b,** recall, **c,** precision, **d,** FPR. Source data are provided with this paper.

**All simulation results of streak pattern**

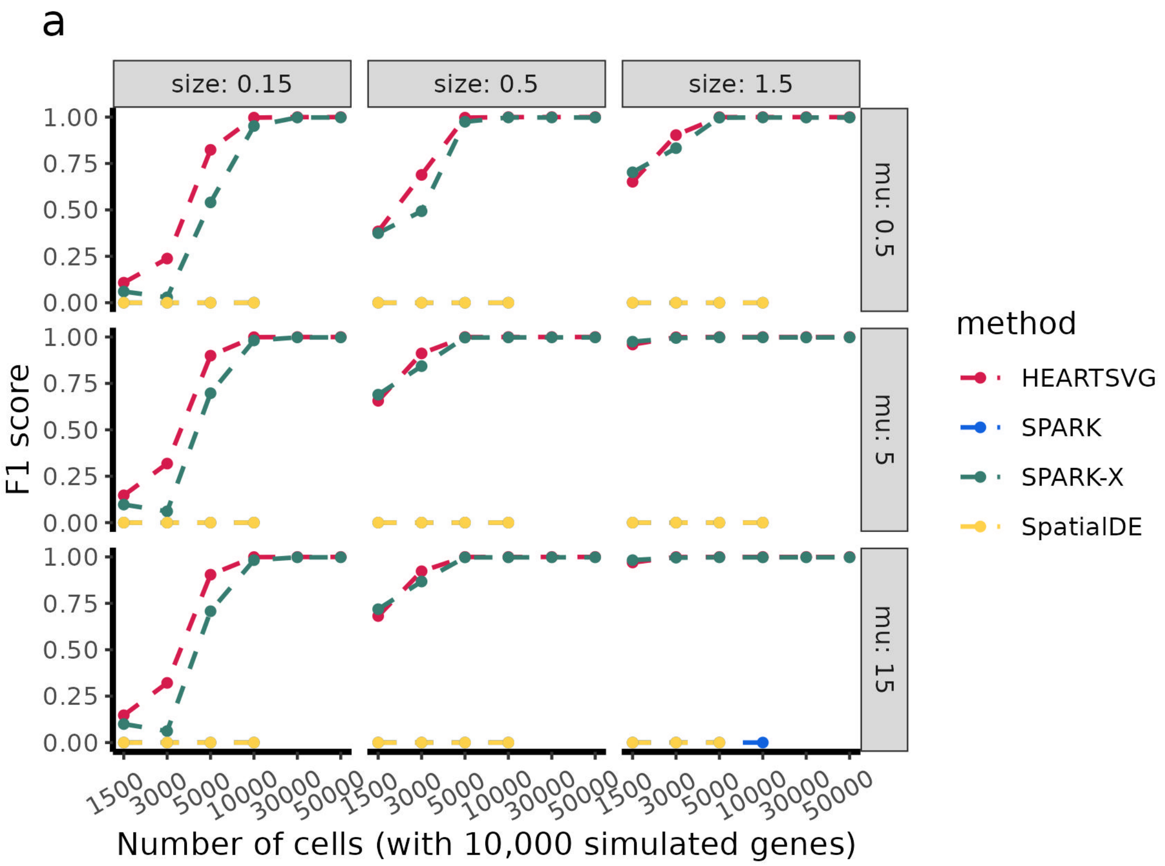


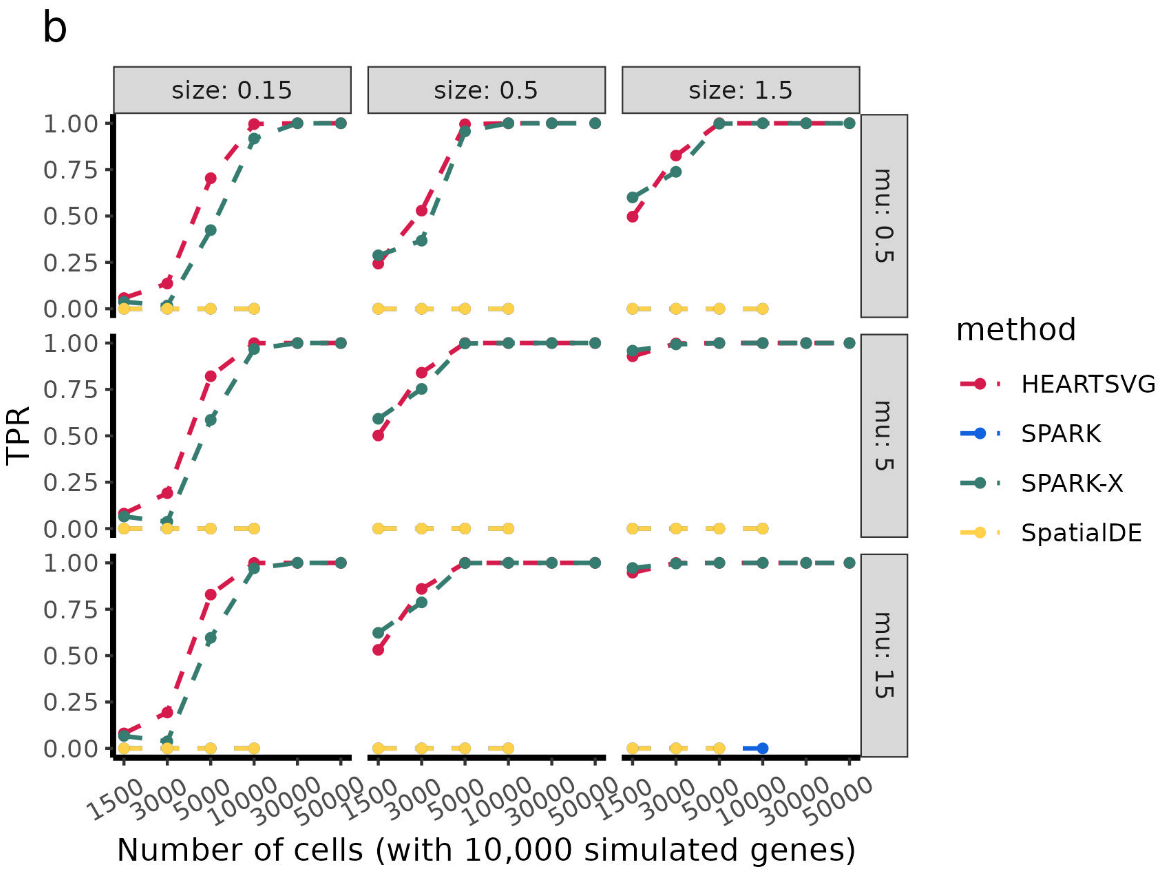

c

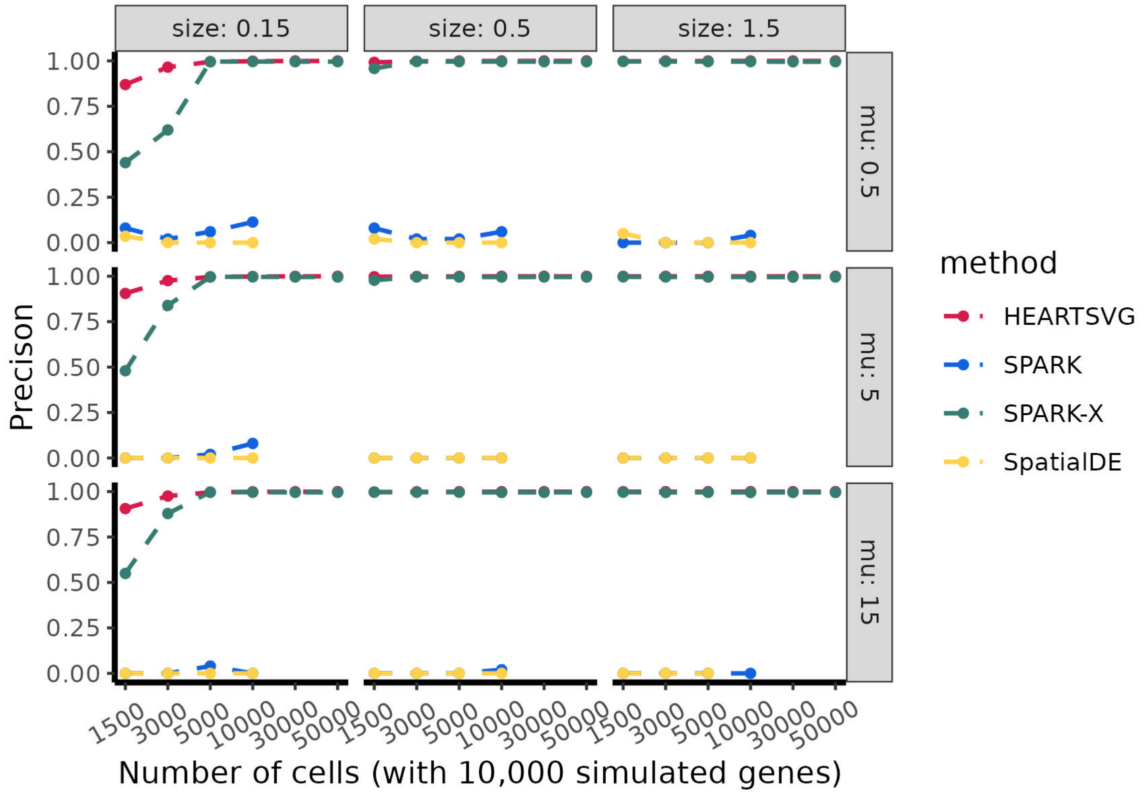


d

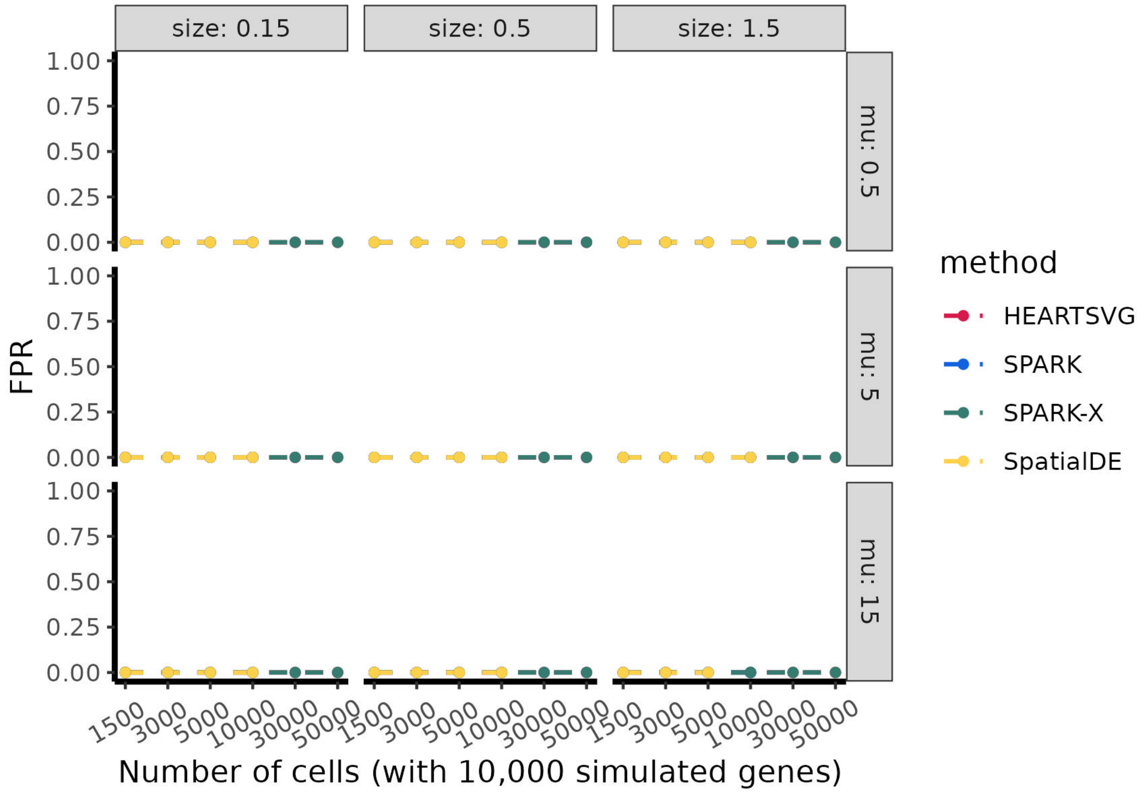


**Figure S2** Simulation results of streak pattern. **a,** F1 score, **b,** recall, **c,** precision, **d,** FPR. Source data are provided with this paper.

**All simulation results of gradient patter**

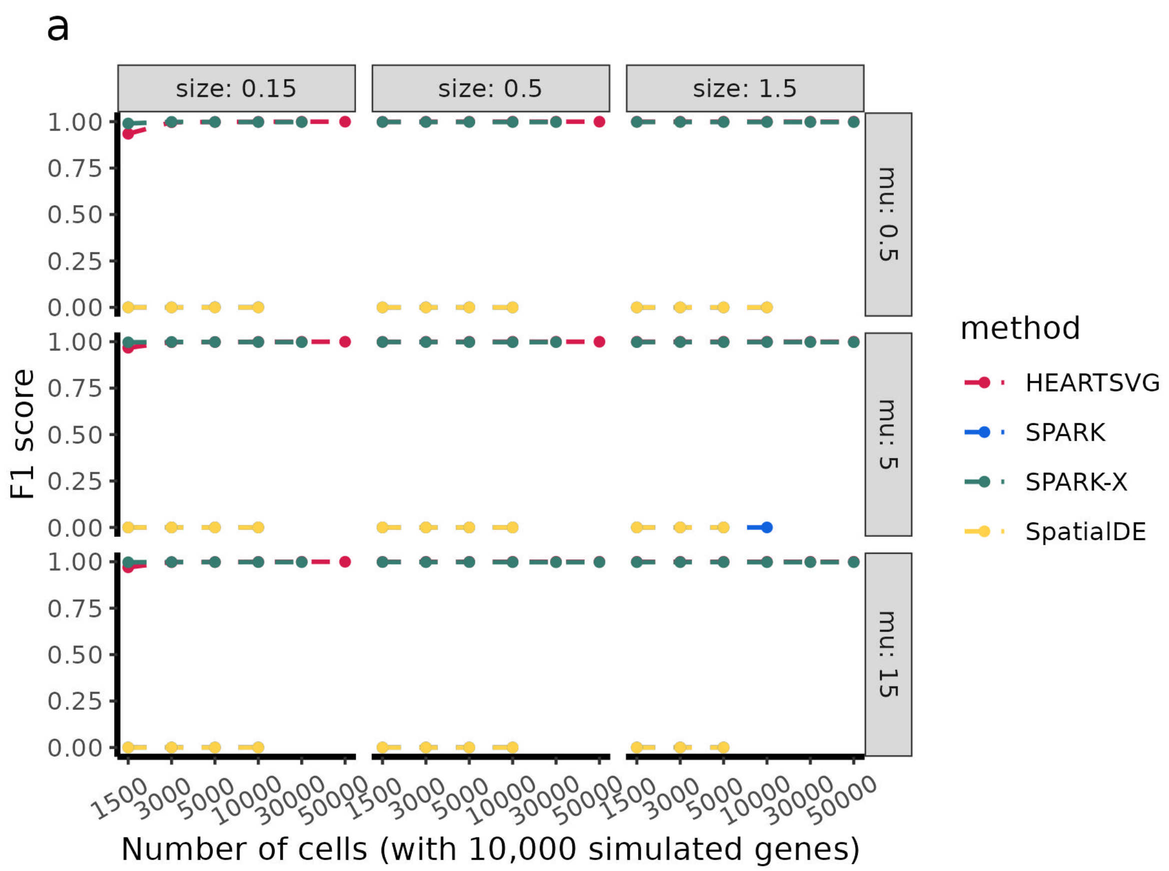


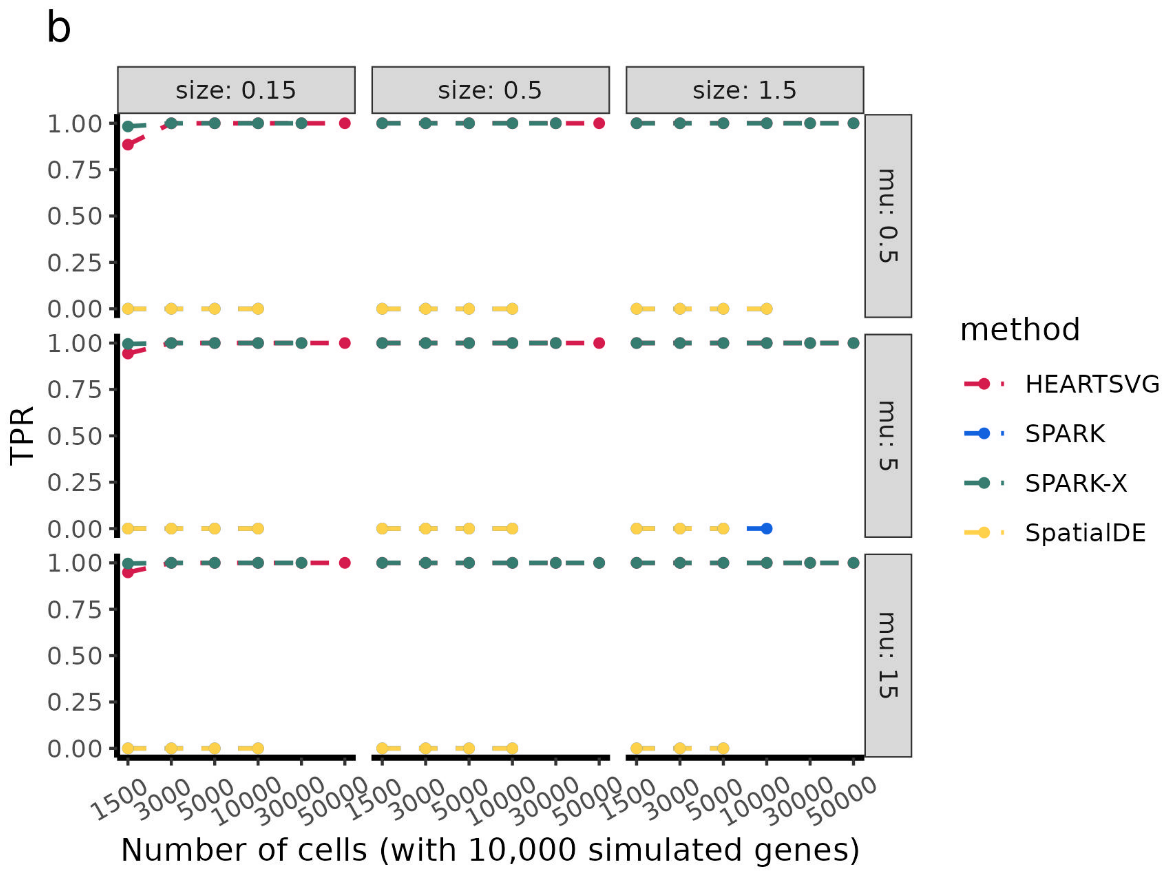

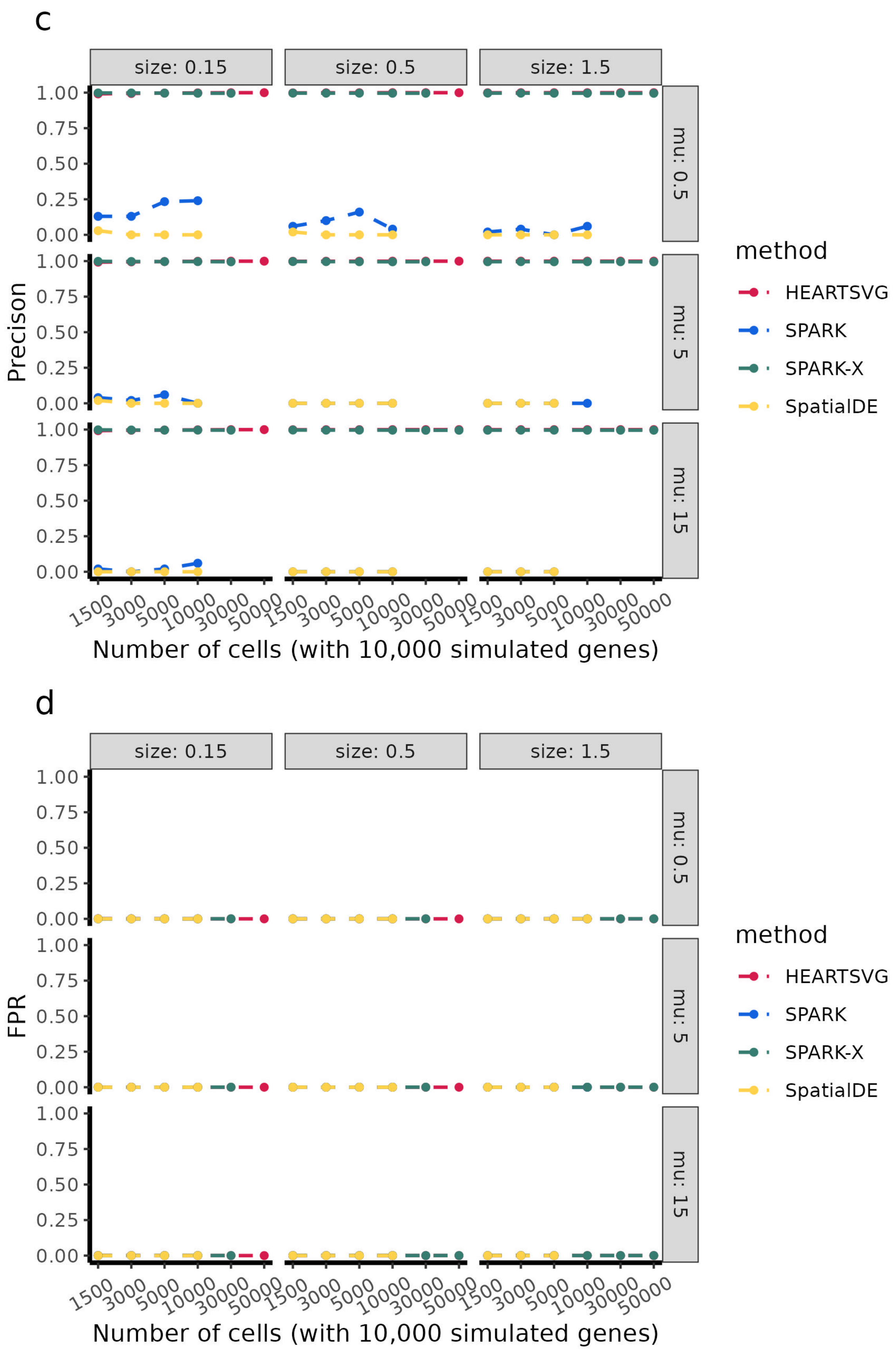


**Figure S3** Simulation results of gradient pattern. **a,** F1 score, **b,** recall, **c,** precision, **d,** FPR. Source data are provided with this paper.

### 1.2 Simulation 2 additional figures

Because the uncertainty surrounding the number of SVGs in real data is unknown, we generated simulation datasets with varying percentages (from 0% to 50%) of SVGs and sample sizes (from 3000 to 10,000) in three representative spatial patterns. We also compared the performance of HEARTSVG and SPARK-X using $F_1$ score, and false positive (FP). The paper presented the simulation scenario with ZINB parameters ($size = 0.5, mu = 0.5$) and moderate sample size (n=5000). The complete simulation results were shown in this supplementary.

**Hotspot pattern with varying percentages of SVGs**

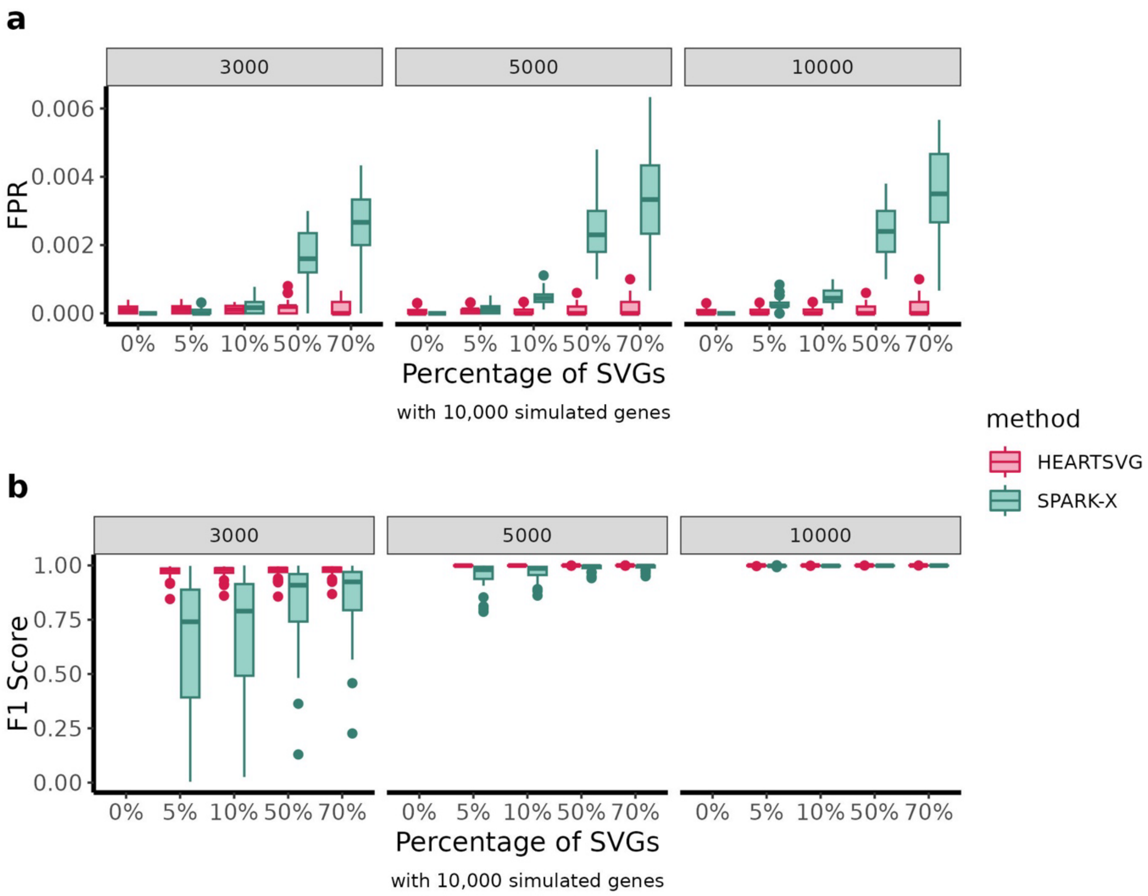


**Figure S4** Simulation2 results of hotspot pattern. **a,** FPR comparison between HEARTSVG and SPARK-X for different percentages of SVGs. **b,** $F_1$ score comparison between HEARTSVG and SPARK-X for the same conditions. In each boxplot, the lower hinge, upper hinge, and center line represent the 25th percentile (first quartile), 75th

percentile (third quartile), and 50th percentile (median value), respectively. Whiskers extend no further than ±1.5 times the inter-quartile range. Data beyond the end of the whiskers are considered outliers and are plotted individually. Source data are provided with this paper.

**Streak pattern with varying percentages of SVGs**

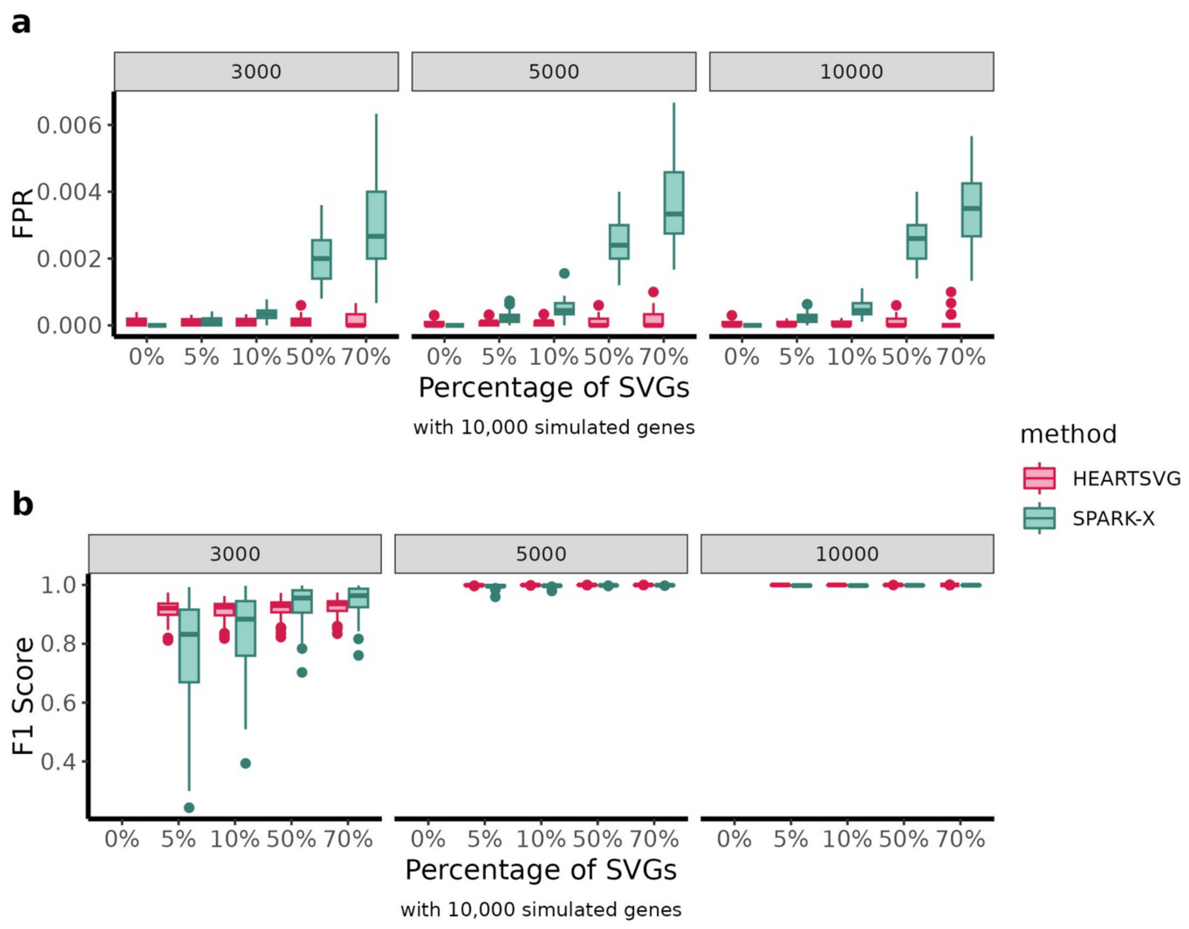


**Figure S5** Simulation2 results of streak pattern. **a,** FPR comparison between HEARTSVG and SPARK-X for different percentages of SVGs. **b,** $F_1$ score comparison between HEARTSVG and SPARK-X for the same conditions. In each boxplot, the lower hinge, upper hinge, and center line represent the 25th percentile (first quartile), 75th percentile (third quartile), and 50th percentile (median value), respectively. Whiskers extend no further than ±1.5 times the inter-quartile range. Data beyond the end of the whiskers are considered outliers and are plotted individually. Source data are provided with this paper.

**Gradient pattern with varying percentages of SVGs**

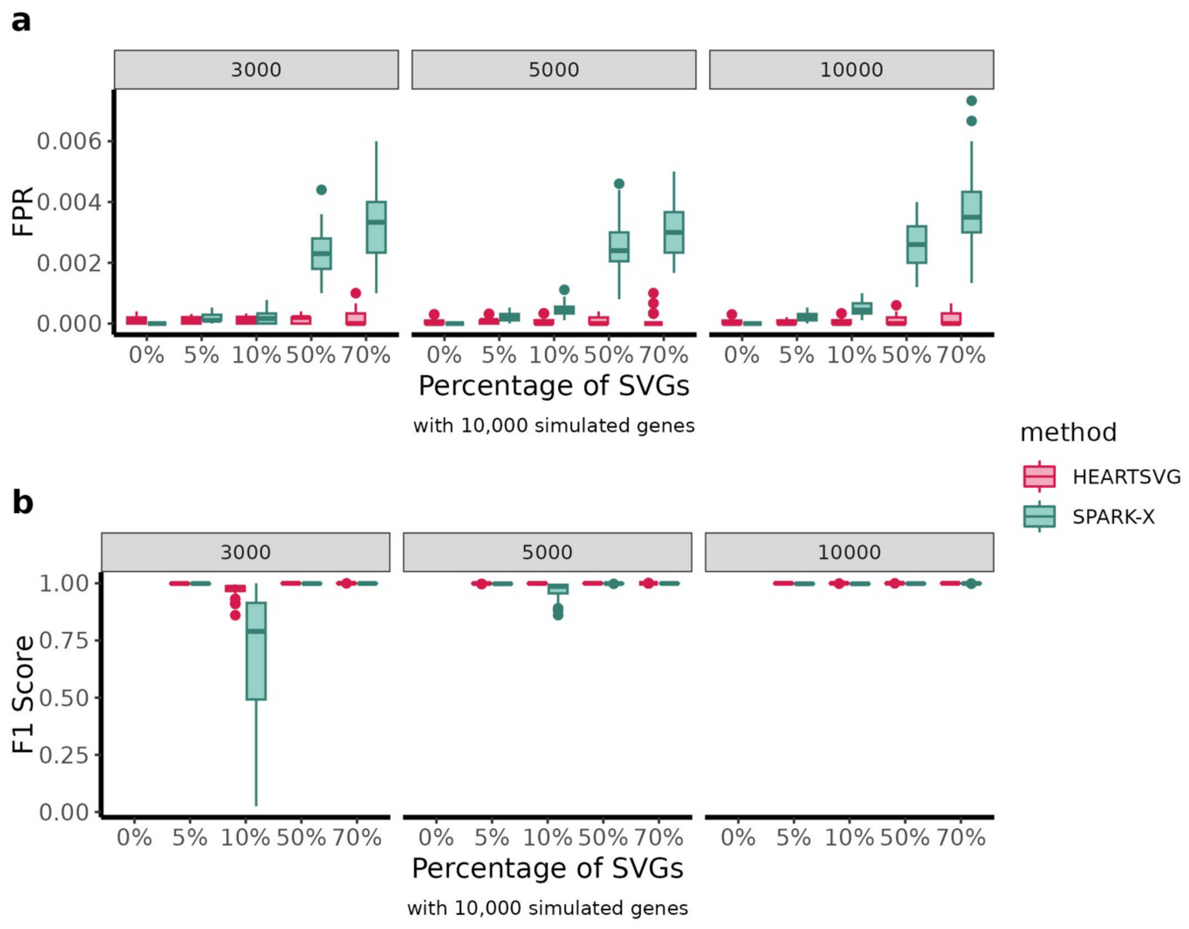


**Figure S6** Simulation2 results of gradient pattern. **a,** FPR comparison between HEARTSVG and SPARK-X for different percentages of SVGs. **b,** $F_1$ score comparison between HEARTSVG and SPARK-X for the same conditions. In each boxplot, the lower hinge, upper hinge, and center line represent the 25th percentile (first quartile), 75th percentile (third quartile), and 50th percentile (median value), respectively. Whiskers extend no further than ±1.5 times the inter-quartile range. Data beyond the end of the whiskers are considered outliers and are plotted individually. Source data are provided with this paper.

### 1.3 More comparisons

We evaluated time consumption and memory requirements of four methods (HEARTSVG, SPARK-X, scGCO, and Squidpy) on real biological samples from mouse hypothalamus, comprising 1027,848 cells and 161 genes (**Figure S7)**. HEARTSVG required 1.43 mins and 7.31 GB, scGCO needed a runtime of 112 mins and 14.72 GB, SPARK-X took 0.62 mins and 5.78 GB, and Squidpy took 3.73 mins, and 7.78GB. We attempted to compare the performance of HEARTSVG, scGCO, SPARK-X and Squidpy on simulated data with 2 million cells (1000 simulated genes). HEARTSVG completed the computation in 4 to .5 minutes and 82.70GB, Squidpy took 188.5 minutes and 343.7 GB, while SPARK-X and scGCO failed to scale to the dataset with 2 million cells. The new results are presented below and depicted in both the revised manuscript and the supplementary materials.

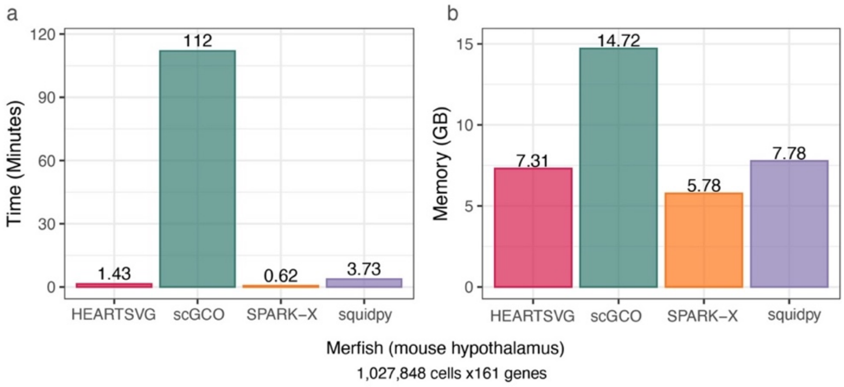


**Figure S7** **a,** Bar diagram shows time consumption (y-axis) of four methods on mouse hypothalamus data (1027,848 cells and 161 genes) by MERFISH technology. **b,** Bar diagram shows memory requirements (y-axis) of four methods.

## 2. Methods

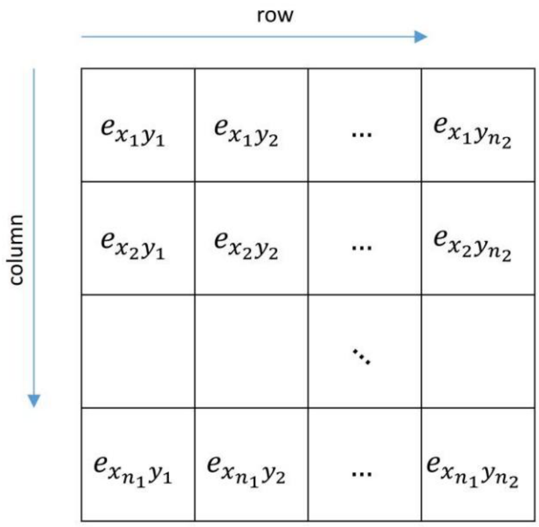


**Figure S8** Example of ST data.

### 2.1 The following demonstrates the rationality of HEARTSVG.

For a gene $g$ without the spatial pattern in the ST dat**a,** its expression count $e$ is independent of its coordinates $(x, y)$. Define $f(x, y, e) = g(e|\boldsymbol{\theta})$, the gene $g$'s expression $e$ of each spot is only dependent on the parameters $\boldsymbol{\theta}$ (e.g., $\boldsymbol{\theta}$ could be the $\lambda$ of the Poisson distribution, $(\alpha, \beta)$ of the Gamma distribution, etc.).

That is, the gene $g$'s expression counts $e$ of the SRT data are independent and identically distributed random variables (Abbreviated as iid random variables).

We assume that every row has $n_2$ elements, every column has $n_1$ elements and expression matrix are a $n_1 * n_2$ matrix (Fig.**S8)**. Next, take the marginal expression series obtained by the semi-pooling process with the row direction and feature map $(1 * n_2)$ as an example.

The gene g's expressions of the first row are denoted by $r_{x_1} = \left(e_{x_1y_1}, \dots, e_{x_1y_j}, \dots, e_{x_1y_{n_2}}\right)^{\boldsymbol{T}}$. The joint distribution of the first row is $f(r_{x_1}|\boldsymbol{\theta}) = f\left(e_{x_1y_1}, \dots, e_{x_1y_j}, \dots, e_{x_1y_{n_2}}\middle|\boldsymbol{\theta}\right) = \prod_{j=1}^{n_2} g\left(e_{x_1y_j}\middle|\boldsymbol{\theta}\right)$. Similarly, the expression vector and joint distribution of the second row are $r_{x_2} = \left(e_{x_2y_1}, \dots, e_{x_2y_j}, \dots, e_{x_2y_{n_2}}\right)^{\boldsymbol{T}}$ and $f\left(e_{x_2y_1}, \dots, e_{x_2y_j}, \dots, e_{x_2y_{n_2}}\middle|\boldsymbol{\theta}\right) = \prod_{j=1}^{n_2} g(e_{x_2y_j}|\boldsymbol{\theta})$, respectively. The expression vector and joint distribution of the $i$-th row

are $r_{x_i} = \left(e_{x_i y_1}, \dots, e_{x_i y_j}, \dots, e_{x_i y_{n_2}}\right)^{T}$ and $f\left(e_{x_i y_1}, \dots, e_{x_i y_j}, \dots, e_{x_i y_{n_2}} \middle| \boldsymbol{\theta}\right) = \prod_{j=1}^{n_2} g(e_{x_i y_j}|\boldsymbol{\theta})$, respectively. Obviously, the joint distributions of each row's expressions are identically distributed. $r_{x_1}$ and $r_{x_2}$ have no overlapping elements. Hence, it is easy to prove that $f(r_{x_1}, r_{x_2}|\boldsymbol{\theta}) = f(r_{x_1}|\boldsymbol{\theta}) * f(r_{x_2}|\boldsymbol{\theta})$, which means $r_{x_1}$ and $r_{x_2}$ are independent and identically distributed random variables.

Similarly, we can prove that, $\forall\, i_1, i_2 = 1, \dots, n_1,\ and\ i_1 \neq i_2,\ r_{x_{i_1}}$ and $r_{x_{i_2}}$ are iid variables. By the similar derivation process as above, we can prove that the elements of the marginal expression series obtained by semi-pooling parameters with different parameters are iid variables. This is a very strong condition and is hard to verify empirically.

In practice, we assume that the expression counts of the non-SVG gene at a given location $(x_i, y_j)$ are independent of expressions at nearby locations. Therefore, we applied the Portmanteau test to test several autocorrelations of $r_t$ that are simultaneously at zero to determine whether the gene is a SVG. The null and alternative hypotheses are:

$$\mathrm{H_0}: \rho_1 =, \dots, = \rho_m = 0, \mathrm{H_A}: \exists\, k \in \{1, \dots, m\}, \rho_k \neq 0$$

To simplify the symbolic representation, we rewrite the subscript of the marginal expression series as $\mathbf{r} = (r_1, .., r_t, \dots, r_T)^T$, define the autocovariance of order $m$ as:

$\gamma_k = Cov(r_t, r_{t-k}) = Cov(r_t, r_{t+k})$, for all $k \geq 0$, and the $j$th order autocorrelation (ACF) as $\rho_k = \frac{\gamma_k}{\gamma_0}$.

If gene $g$ is non-SVG without a spatial pattern in ST dat**a,** our purpose is to test the null hypothesis: $\mathrm{H_0}: \rho_1 = \cdots = \rho_m = 0$.

The test statistic is defined as $Q_m = T \sum_{k=1}^{m} \hat{\rho}_k^2$ followed by chi-distribution with $m$ degree of freedom, where $\hat{\gamma}_k = \frac{1}{T-k} \sum_{t=1+k}^{T} (r_t - \bar{r})(r_{t-k} - \bar{r}), k = 0, \dots, T-1$, $\bar{r}$ is the mean of $\mathbf{r}$, and introduce $\hat{\rho}_k = \frac{\hat{\gamma}_k}{\hat{\gamma}_0}$. The $P$ value for testing the null hypothesis can be calculated by $p = \mathrm{P}(\chi^2(df = m) > Q_m \mid \mathrm{H_0} \text{ is true})$

**Stouffer's method**

We combined all four P values into a single P value by Stouffer's method. The Stouffer's statistic is defined as $z_{stouffer} = \sum_{i=1}^{4} \frac{z_i}{\sqrt{4}} \sim N(0,1)$, where $z_i = \Phi^{-1}(1 - p_i)$, $\Phi^{-1}(\cdot)$ is

the inverse of the cumulative distribution function of a standard normal distribution. Hence, the combined p of four p-values is calculated by $\boldsymbol{p_c = 1 - \Phi(z)}$.

**Why use the Stouffer Combination?**

In the 10X Visium colorectal cancer dat**a,** we selected the top 500 overlaps from the results of all six methods as SVGs and created non-SVGs by randomly rearranging gene expressions of SVGs. Then, we investigated the distributions of p-values obtained from the autocorrelation test applied to the marginal expression time series (**Figure S10 a-d**). Moreover, we compared two common combination methods: Fisher's combination method and Stouffer's method. Fisher's method ($z_{Fisher} = -2\sum_{i=1}^{4}\ln(\mathrm{p_i}) \sim \chi^2(\mathrm{df} = 8)$) directly processed the p-values, while Stouffer's method ($z_{Stouffer} = \sum_{i=1}^{4}\frac{\Phi^{-1}(1-p_i)}{\sqrt{4}} \sim N(0,1)$) first transformed the p-values into Z-scores, and then combined them. We calculated the combined p-value statistics of the two methods, plotted the density histograms (**Figure S10e, S10f**), and compared them with their theoretical distribution density curves (the black solid lines in the figure), as shown in **Figure S10.** It can be seen that the distribution of Stouffer's statistic was closer to the theoretical distribution, indicating that choosing Stouffer's method was reasonable.

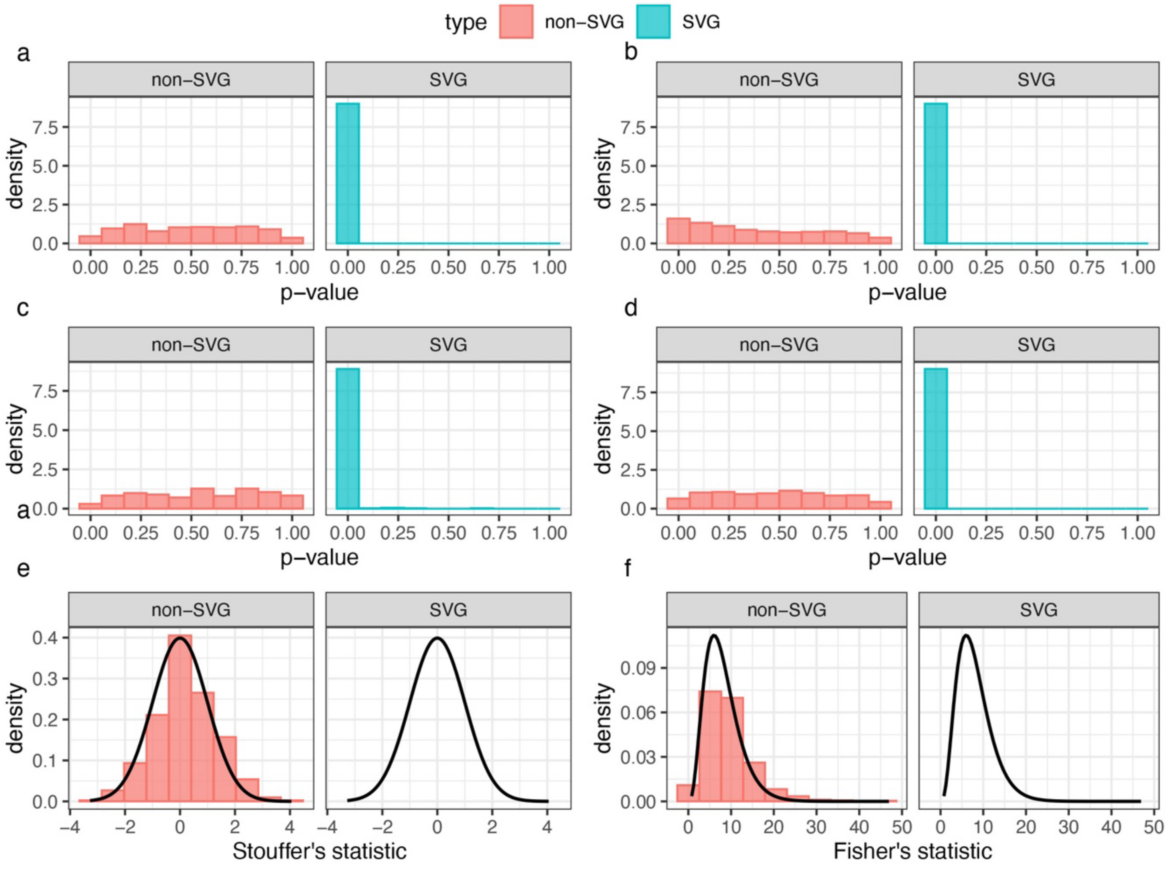


**Figure S9** **a-d,** The density distributions of p-values obtained from the autocorrelation test applied to the marginal expression time series. **e,** The density distributions of Stouffer's statistic. The black solid line show the theoretical distribution ($N(0,1)$) of the Stouffer's statistic. **f,** The density distributions of Fisher's statistic. The black solid line shows the theoretical distribution ($\chi^2(df = 8)$ of the Fisher's statistic.

## 2.2 Semi-pooling process

The semi-pooling process needs two parameters: direction parameter and feature map parameter. For each gene, the spatial expression data was averaged according to the given direction and step parameters, and the mean value was used as the new marginal expression value (**Figure S12**). HEARTSVG used four sets of different semi-pooling parameters, which are:

1) Direction: row direction, feature map: $1 \times n_{row}$;
2) Direction: row direction, step: feature map: $1 \times [\ln(n_{row})]$;
3) Direction: column direction, feature map: $1 \times n_{col}$;
4) Direction: column direction, step: feature map: $1 \times [\ln(n_{col})]$

where $n_{row}$ is the number of rows in the spatial transcriptome data, $n_{col}$ is the number of columns in the spatial transcriptome data, and $[\cdot]$ means rounding to the nearest integer.

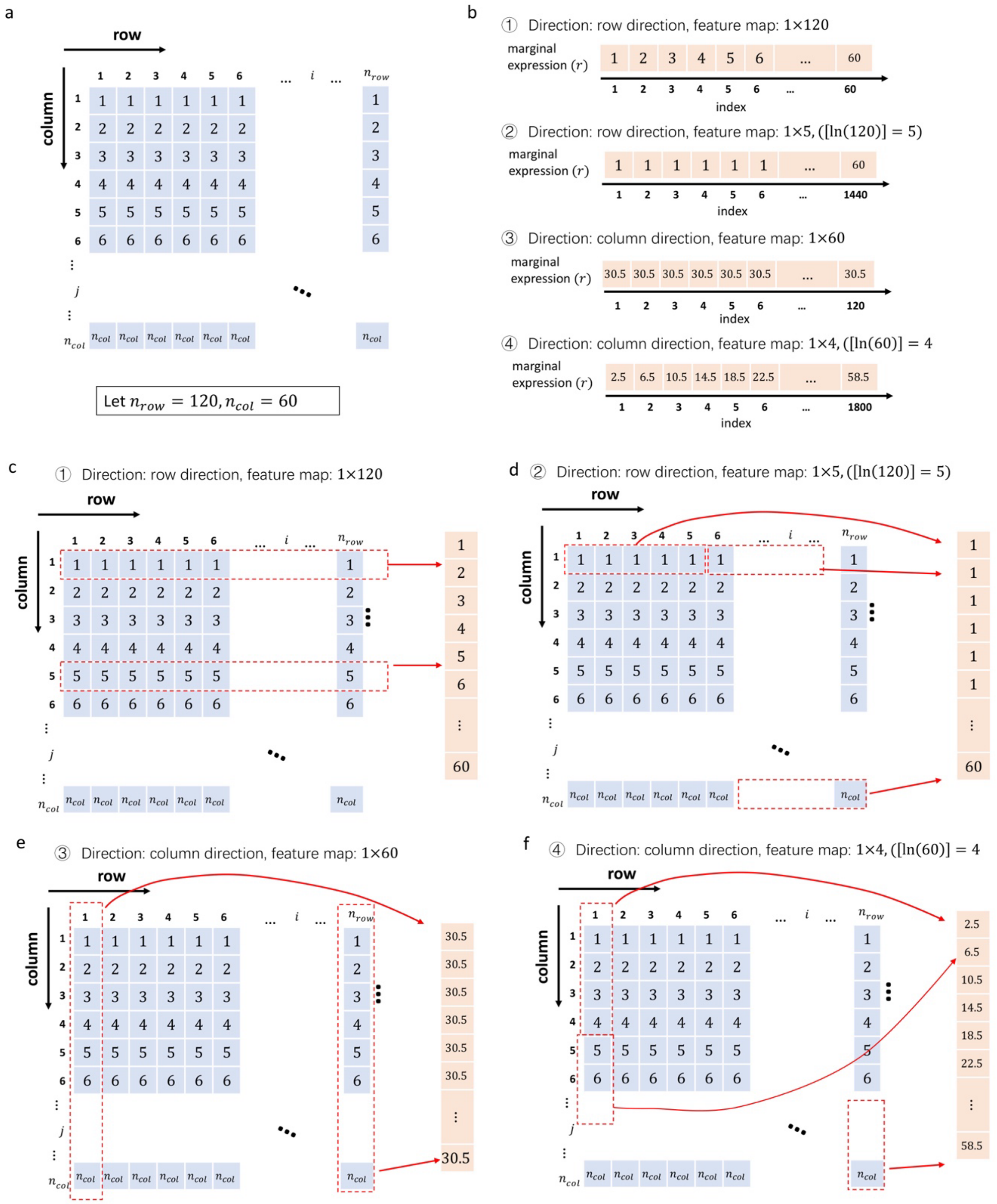


**Figure S10 Illustration of the semi-pooling process. a,** Spatial expression data schematic diagram. We assume the expression value of the gene in each cell is the order value of the column it belongs to. **b,** Four marginal expression series. **c-f,** The flowchart of each new marginal expression series based on four different sets of parameters.

## 2.3 Auto-clustering module

The auto-clustering module uses the hierarchical clustering algorithm to cluster SVGs into different spatial expression patterns based on their expression and location similarity. The steps of the auto-clustering module are as follows:

**Step 1:** Calculate the similarity between each pair of genes based on spatial expression and generation of the distance matrix.

We calculated the Euclidean distance between each pair of SVGs based on their expression and positions, serving as a measure of similarity among SVGs.

**Step 2:** Construct a clustering tree based on the distance matrix using the complete linkage criterion. The resulting hierarchy of clusters can be visualized as a dendrogram.

Initially, each gene is assigned to its own cluster. Then, in each iteration, the two closest clusters are merged into a new cluster using the complete linkage method, which is a method that determines the distance between two clusters by the maximum distance between any two data points from different clusters, to ensure significant dissimilarity between clusters. This process repeats until all data points are eventually merged into a single cluster (a clustering tree).

**Step 3:** Determination of the final clustering results by cutting the dendrogram at a certain height or distance threshold. The cutting height is chosen using the maximum breakpoint of all breakpoints selected by the Yamamoto test.

After forming the hierarchical structure, we arrange the heights in ascending order and utilize the Yamamoto test to identify breakpoints at different height thresholds. The maximum breakpoint value is employed as the cutting height to determine the final number of clusters. The Yamamoto test employs the rolling window method to calculate the difference of heights before and after each point. If the difference exceeds the threshold, the i-th point is considered a breakpoint. The details were listed below.

We had a serial: $h_1, \dots h_i, \dots, h_N$, set window width: $2 * n_{period}$. Then we calculated the difference of heights before and after the i-th point: $D(h_i) = \frac{|mean(\mathrm{H}_{i-before}) - mean(\mathrm{H}_{i-after})|}{sd(H_{i-before}) + sd(\mathrm{H}_{i-after})}$, where, $\mathrm{H}_{i-before} = (h_{i-1}, \dots, h_{i-n_{period}})$, $\mathrm{H}_{i-after} = (h_{i+1}, \dots, h_{i+n_{period}})$, mean($\cdot$) and

sd(·) are the mean and standard error function, the $threshold = \frac{t_{1-\alpha-(df=n_{period}-1)}}{\sqrt{n_{period}}}$. If $D(h_i) > threshold$, the i-th point is a breakpoint. We identified all breakpoints of the serial and chose the max breakpoint as the cutting height of the cluster tree.

The rationale behind the Yamamoto test lies in the characteristics of hierarchical clustering. SVG clusters with similar spatial expression patterns have smaller distances and form clusters at lower heights in the tree structure, while SVG clusters with different patterns have larger distances and form clusters at higher heights in the tree structure. Thus, when merging clusters of SVGs with different patterns, there is a noticeable jump in height.

### 2.4 Simulation parameters

**Table S1 Simulation settings of simulated data**

| | non-SVG and non-marked area of SVGs | | | marked area of SVGs | | |
|---|---|---|---|---|---|---|
| | probability of extra zeros | mu/lambda | size | probability of extra zeros | mu/lambda | size |
| ZINB | 0.8 | 0.5 | 0.5 | 0.267 | 1 | 1 |
| ZIP | 0.6 | 2 | - | 0.2 | 6 | - |
| NB | - | 0.5 | 1.5 | - | 1.5 | 1.5 |
| Poisson | - | 0.5 | - | - | 1.5 | - |

We used four distributions, Poisson, ZIP, NB, and ZINB, in our simulation. ZINB distribution is suitable for simulating highly sparse data. Poisson, ZIP, NB are suitable for simulating moderately sparse dat**a,** where the mean and variance of Poisson distribution are equal, while ZIP, NB can simulate higher dispersion than Poisson distribution. Over-dispersion is a characteristic of spatial transcriptomics data and single-cell data.

**We used Poisson distribution to generate simulated data and followed the parameter settings of SPARK**[1]. Firstly, we introduce the parameters used in the SPARK manuscript. For the gene expression in the *i*-th spot/cell of non-SVG and non-marked area of SVG, the parameter of the Poisson distribution is:

$$\lambda_{non} = N_i * exp(-10.2 + \tau_i)$$

For the gene expression in the marked area of SVG, the parameter of the Poisson distribution is:

$$\lambda_{SVG} = N_i * \exp(-9.1 + \tau_i)$$

Where $N_i$ is the total read counts obtained from the real data seqFISH data[2], $\tau_i$ is drawn from a normal distribution with mean zero and variance being 0.35. According to the above method, the range of $\lambda_{non}$is about $(0.01,1)$, and the range of $\lambda_{SVG}$ is about $(0.03,3)$. To simplify our simulation, we set $\lambda_{non} = 0.5$, $\lambda_{SVG} = 1.5$, which is three times of the former.

**For the simulated data based on the NB distribution,** we followed the simulation parameter settings of SPARK-X[3]. For gen non-SVGs and non-marked area of SVGs, gene expression follows $NB(size = 1.5, \mu = 0.5)$. For marked area of SVGs, gene expression follows $NB(size = 1.5, \mu = 1.5)$. The parameter size $size = 1.5$ remains unchanged, and $mu_{SVG} = 1.5$ is three times the value $mu_{non} = 0.5$.

**For both ZIP and ZINB distributions,** which are zero-inflated models, we need to set a zero-proportion parameter to control the proportion of zeros. To determine this parameter, we refer to the criteria for SVG in SPA-GCN[4]: "(1) the percentage of spots expressing the gene in the target domain, that is, in-fraction, is >80%; (2) for each neighboring domain, the ratio of the percentages of spots expressing the gene in the target domain and the neighboring domain(s), that is, in/out fraction ratio, is >1; and (3) the expression fold change between the target and neighboring domain(s) is >1.5. If a user is interested in finding SVGs for a particular combination of spatial domains, SpaGCN offers the option to do so."[4]

**Therefore, in the ZIP distribution,** for the gene expression of non-SVG and non-marked area of SVGs, following $ZIP(0.6,2)$. For the gene expression of marked area of SVG, following $ZIP(0.2,6)$. In this case, in the ZIP distribution, the zero proportion of the gene of non-SVG and non-marked area of SVG is 0.654, and the mean is 0.8. The zero proportion of the gene of the marked area of SVG is 0.202, and the mean is 4.8. Compared with the Poisson distribution, the zero proportions of both are close, but the dispersion and expression level of the gene of the marked area of SVG are higher.

**For the ZINB distribution,** we followed the parameter settings for highly sparse data in the SPARK-X[3] and the criteria for SVG in the SPA-GCN[4]. We assumed that the gene expression

of non-SVG and non-marked area of SVG had more than 94% zeros, while the gene of marked area of SVG had a significantly lower zero proportion. Specifically, for non-SVG and non-marked area of SVG, the probability of extra zeros was 0.8, the probability of extra zeros marked area of SVG was 0.8/3. Thus, the gene expression of non-SVG and non-marked area of SVGs, follows $ZINB(0.8,0.5,0.5)$. The gene expression of marked area of SVG, following $ZINB(0.267,1,1)$. In this case, in the ZINB distribution, the zero proportion of the gene of non-SVG and non-marked area of SVG was 0.941, and the mean was 0.1. The zero proportion of the gene of marked area of SVG was 0.633, and the mean was 0.733. Here, we note that the SPARK-X[3] used the NB distribution to generate highly sparse simulated data. For the gene expression of non-SVG and non-marked area of SVG, the parameters of the NB distribution were: $mu_{non} = 0.005, size_{non} = 2.5$, resulting in 99.5% zeros. For the gene expression of marked area of SVG, the parameters of the NB distribution were: $mu_{non} = 0.015, size_{non} = 2.5$, resulting in 98.5% zeros. This means that the genes of non-marked area of SVG and marked area of SVG had very high zero proportions (>98.5%), and very small non-zero expression values. We think that, when the expression of the marked area of SVG is almost zero, it is hard to determine whether a gene is a biologically meaningful SVG, so we also referred to the criteria in the SPA-GCN paper[4].

# 3. Application to colorectal cancer data by 10X Visium

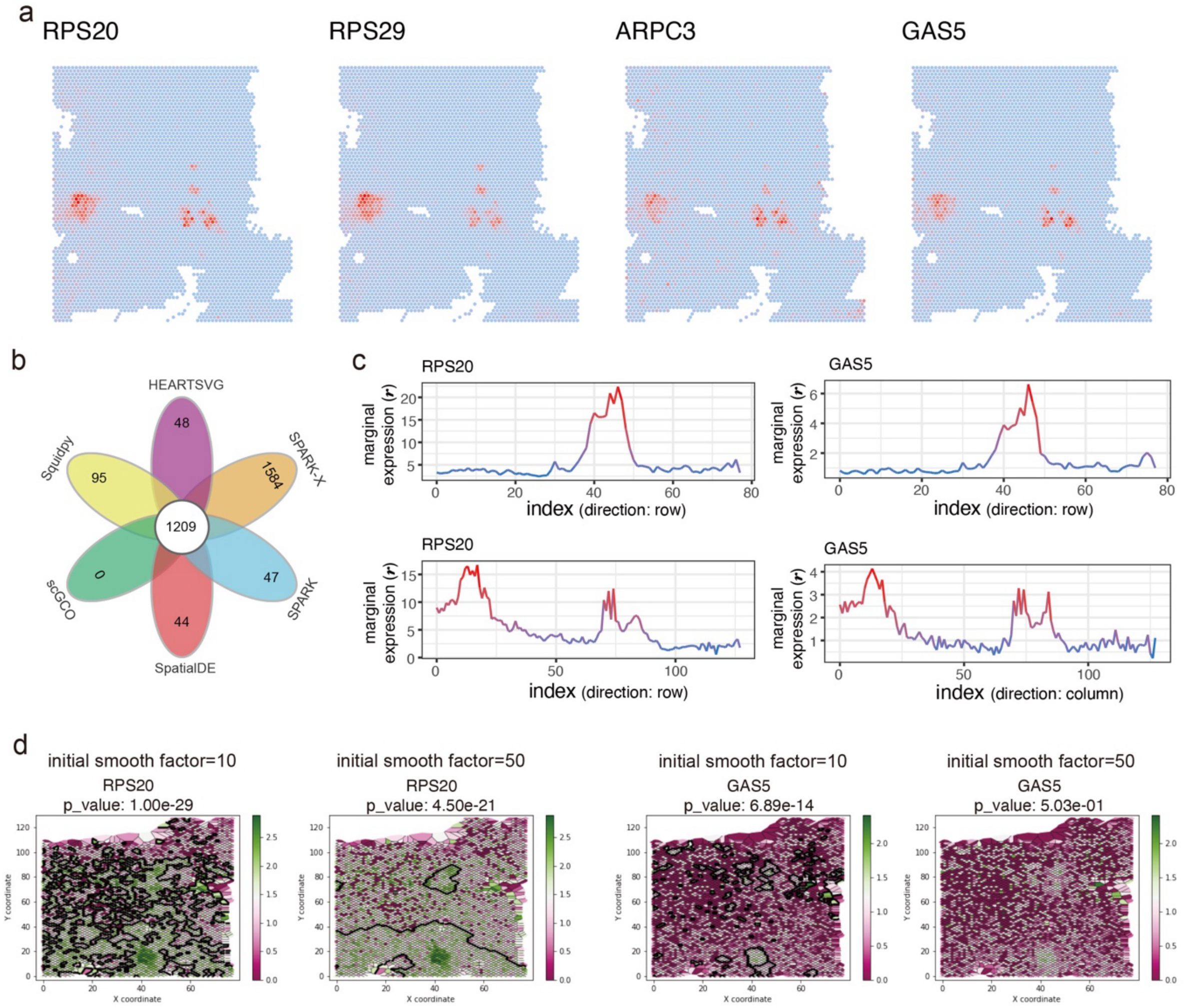


**Figure S11 scGCO missed SVGs (RPS29, ARPC3, GAS5) with clear spatial expression patterns comparing with other methods. a,** Visualizations of spatial expressions of gene RPS20, RPS29, ARPC3, and GAS5. **b,** Venn diagrams of SVGs in the colorectal cancer data identified by HEARTSVG, SpatialDE, SPARK, SPARK-X, scGCO, and Squidpy. **c,** Marginal expression plots of gene RPS20, and GAS5 by HEARTSVG. Source data are provided with this paper. **d,** Visualizations of graph cuts by scGCO with different initial smooth factor of gene RPS20, and GAS5 by HEARTSVG.

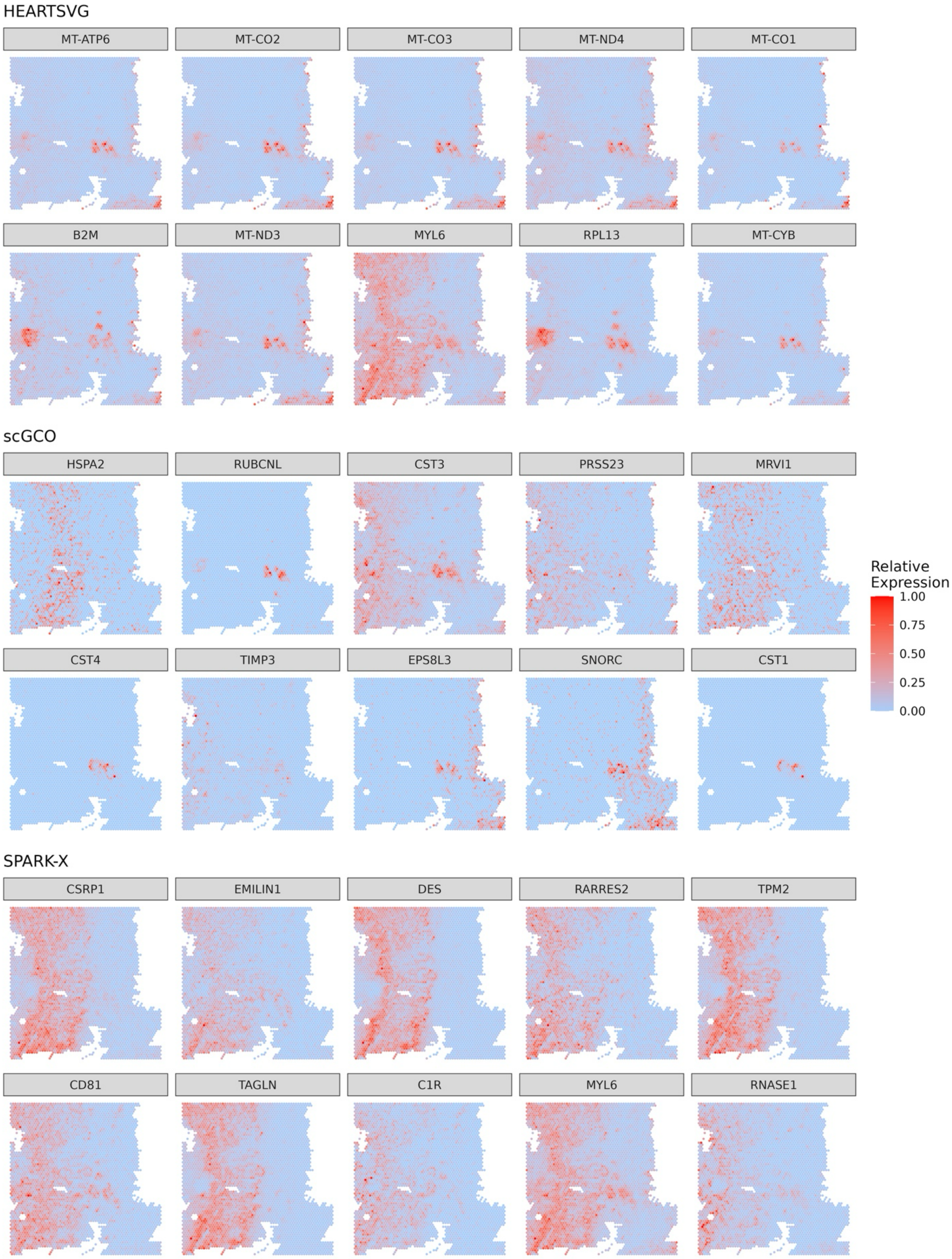
HEARTSVG
MT-ATP6
MT-CO2
MT-CO3
MT-ND4
MT-CO1
B2M
MT-ND3
MYL6
RPL13
MT-CYB
scGCO
HSPA2
RUBCNL
CST3
PRSS23
MRVI1
CST4
TIMP3
EPS8L3
SNORC
CST1
SPARK-X
CSRP1
EMILIN1
DES
RARRES2
TPM2
CD81
TAGLN
C1R
MYL6
RNASE1
Relative Expression
1.00
0.75
0.50
0.25
0.00

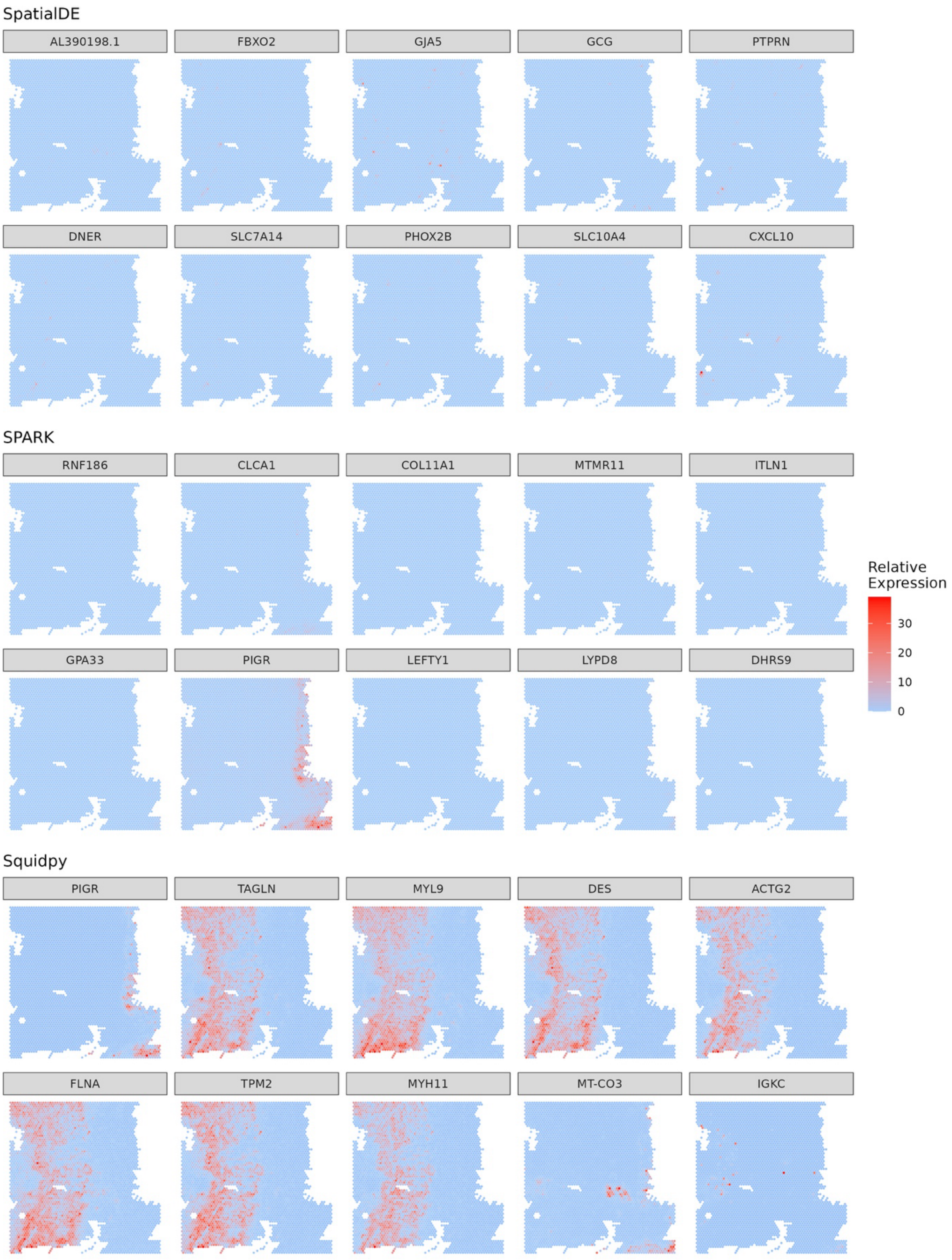


**Figure S12** Top 10 SVGs identified by each method. The top 10 genes identified by HEARTSVG, scGCO SPARK-X and Squidpy showed stronger spatial expression patterns compared to SpatialDE and SPARK (Fig.S10-17). SpatialDE's top 10 selected SVGs exhibited minimal spatial patterns, whereas SPARK performed better than SpatialDE to a certain extent.

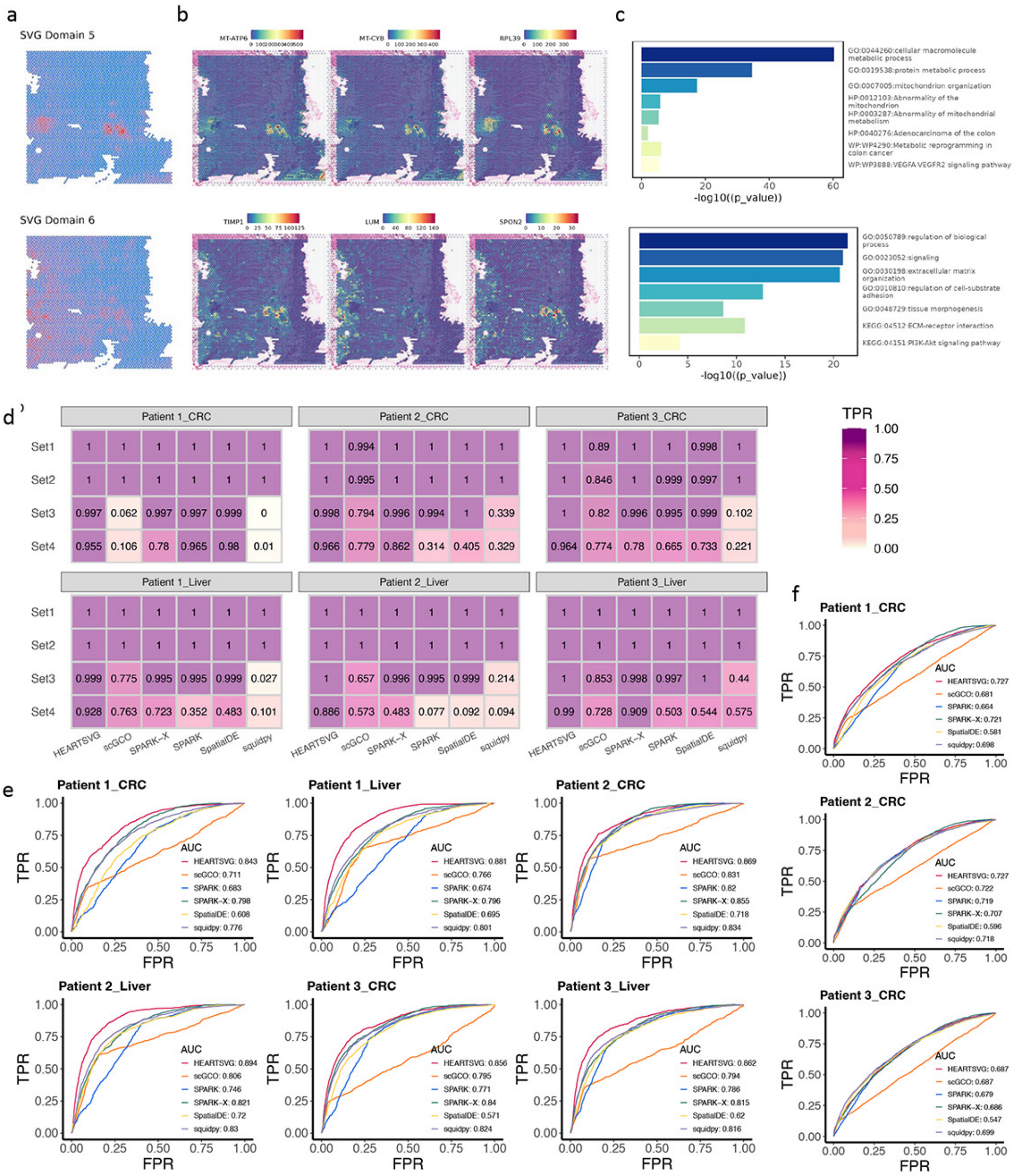


**Figure S13** **a,** HEARTSVG predicts spatial domains 5 and 6 based on SVGs and graphs the average expression of SVGs in each spatial domain. **b,** Representative SVGs correspond to spatial domains 5 and 6. **c,** Enrichment analysis of spatial domains 5 and 6. **d,** The heatmap shows the comparison of recall values among four genesets on three colorectal cancer ST datasets and three corresponding liver metastasis ST datasets. Set 1 represents the true SVGs set, derived by selecting the top 500 overlaps from the results of all six methods. Set 4 corresponds to the non-SVGs set, obtained by randomly rearranging the gene expressions within Set 1. Set 2 and Set 3 are generated by introducing noise to the true SVGs set and non-SVGs set, respectively. **e,** ROC curves were used to assess the true positive rate (TPR)

and false positive rate (FPR) of six different methods, using common gene modules of tumor microenvironments as gold standards for true spatially variable genes (SVGs). The figures consisted of six sub-figures representing three colorectal cancer ST datasets and three corresponding liver metastasis ST datasets. Each method was represented by a different colored line, and the area under the ROC curve (AUC) was calculated. **f,** ROC curves were used to evaluate the TPR and FPR of six different methods, using consensus molecular markers of colorectal cancer subtypes as gold standards for true SVGs. The figures comprised three sub-figures corresponding to three colorectal cancer ST datasets. Source data are provided with this paper.

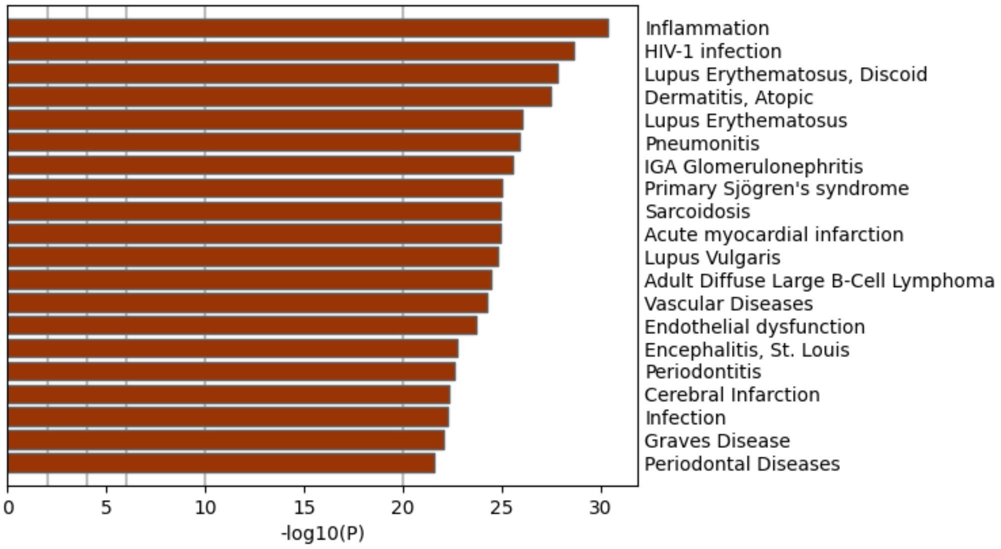


**Figure S14** Enrichment results of Spatial domain 1.

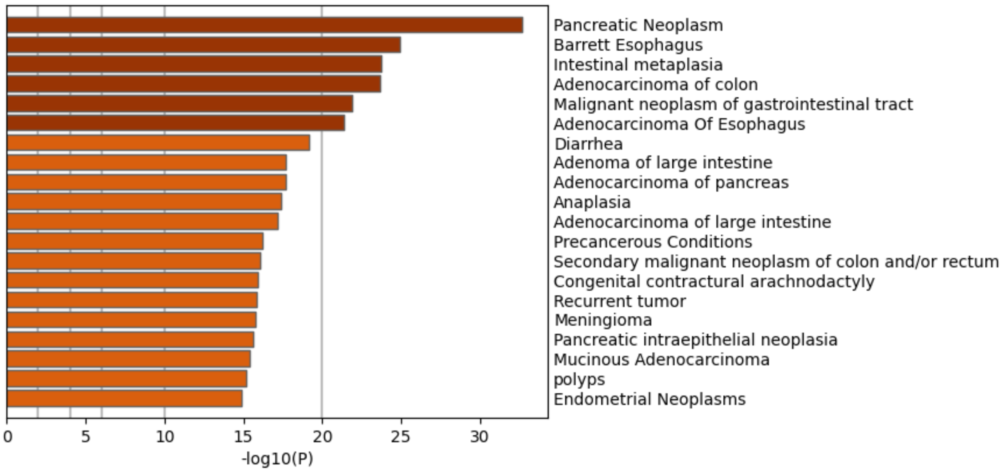


**Figure S15** Enrichment results of Spatial domain 2.

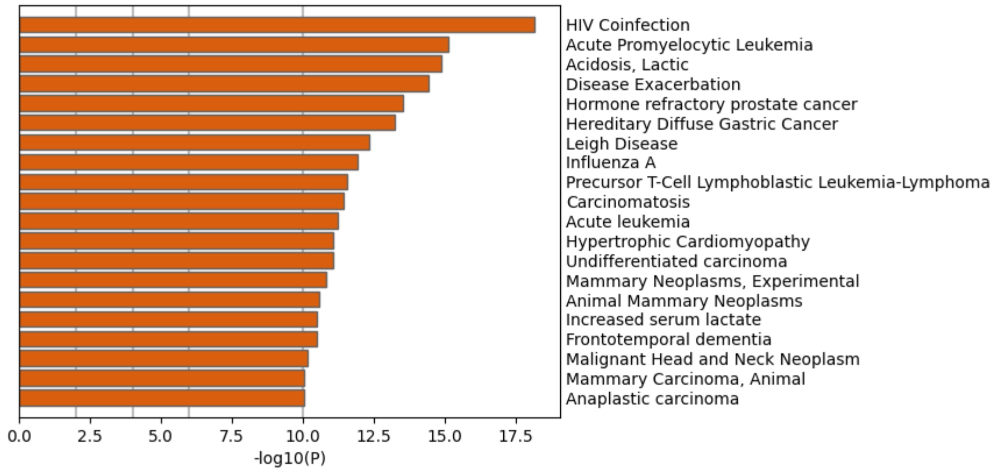


**Figure S16** Enrichment results of Spatial domain 3.

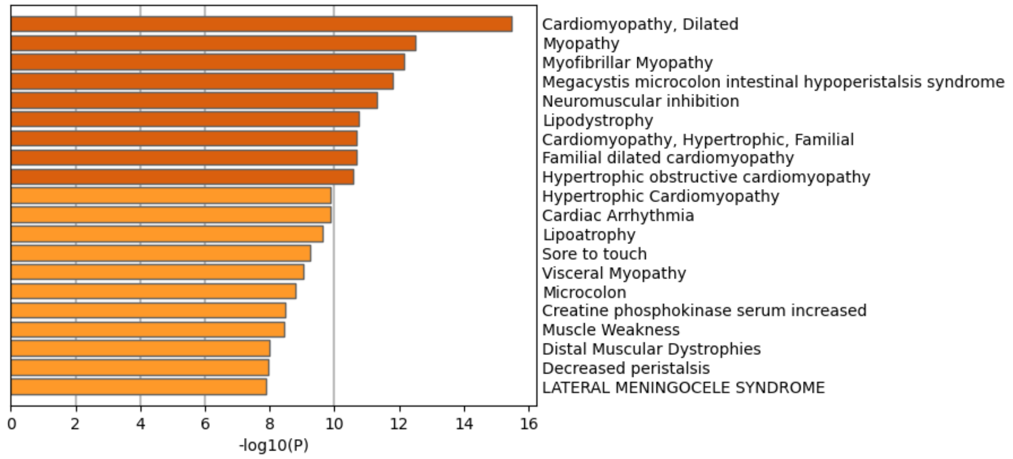


**Figure S17** Enrichment results of Spatial domain 4.

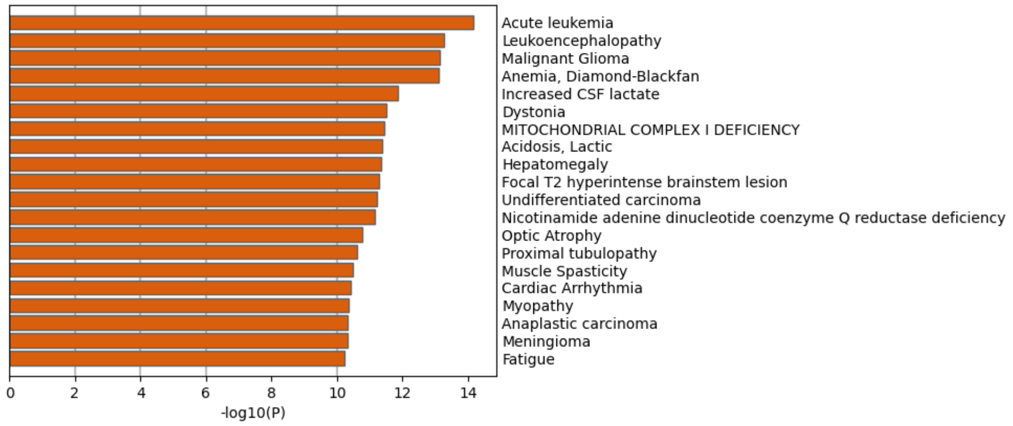


**Figure S18** Enrichment results of Spatial domain 5.

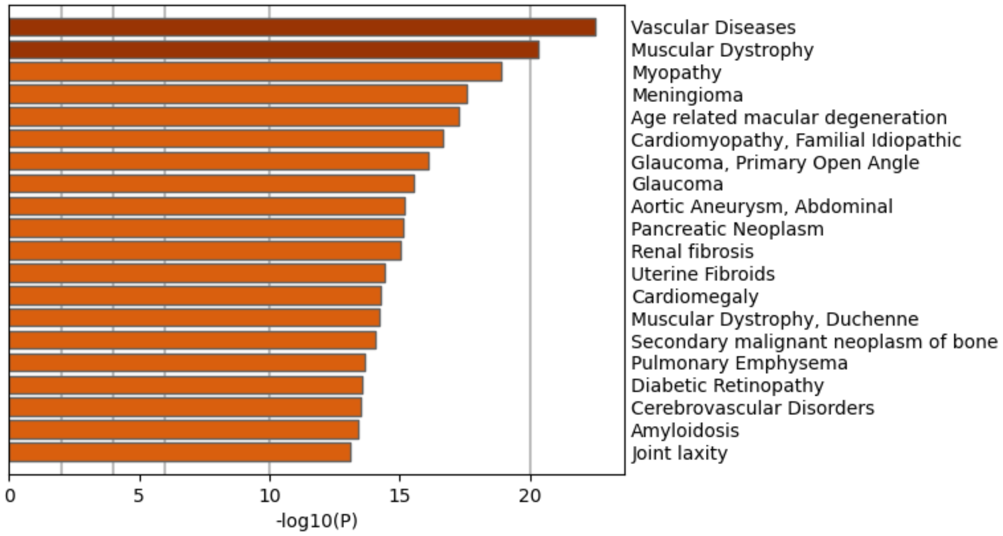


**Figure S19** Enrichment results of Spatial domain 6.

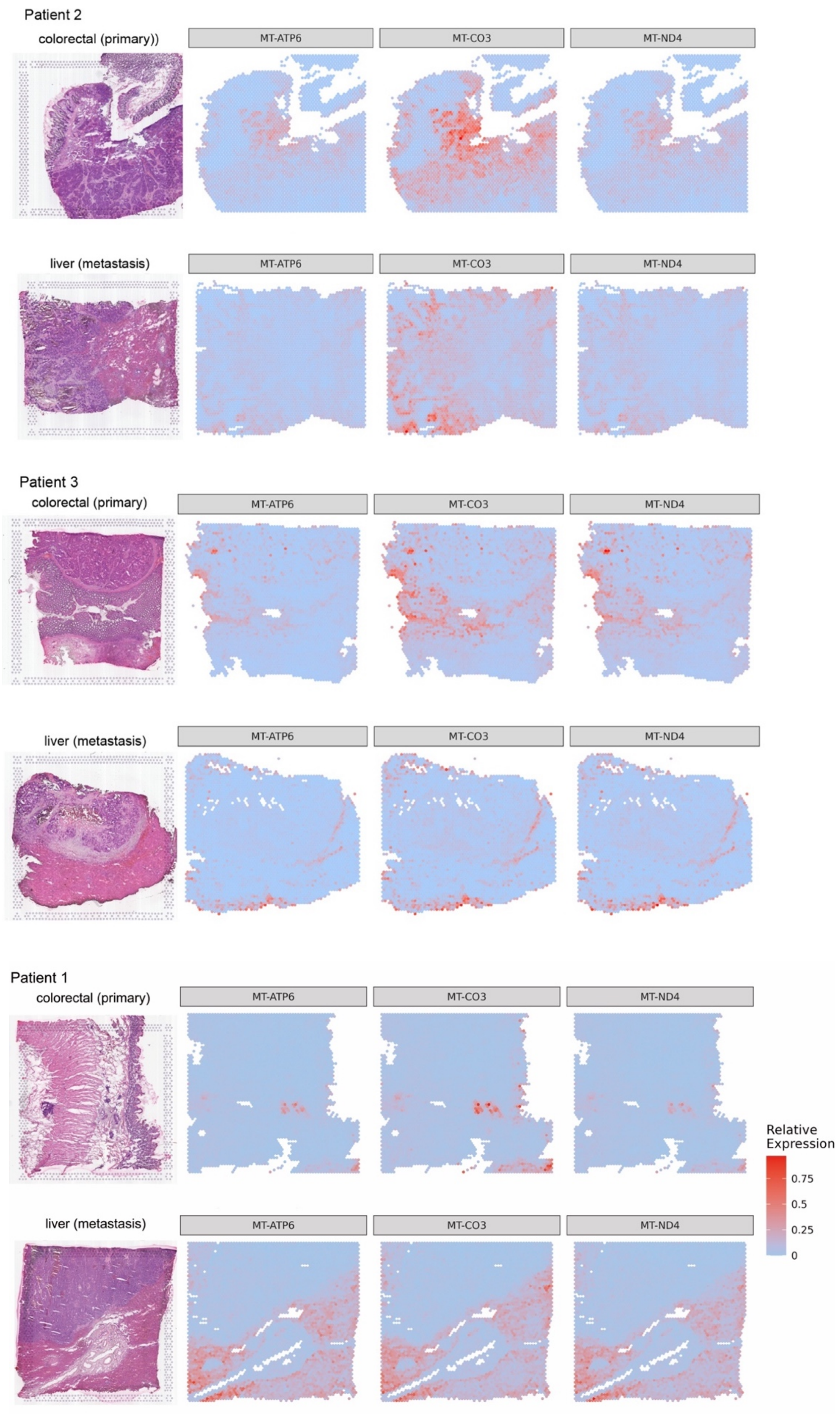


**Figure S20** Mitochondrial-encoded (MT-) genes in primary colorectal cancer tissue and liver metastasis cancer tissue.

# 4. Application to mouse cerebellum data by Slide-seqV2

**Table S2 Tissue-specificity enrichment analysis results of each method. The p-values were obtained through one-sided tests.**

| Method | caudate | cerebellum | cerebral cortex | endometrium | hippocampus | rectum | skin | SUM |
|---|---|---|---|---|---|---|---|---|
| HEARTSVG | 0 | 87.5% (35) | 10% (4) | 0 | 2.5% (1) | 0 | 0 | 100% (40) |
| scGCO | 0 | 92.31% (24) | 7.69% (2) | 0 | 0 | 0 | 0 | 100% (26) |
| SPARK | 5.15% (5) | 43.3% (42) | 7.22% (7) | 0 | 4.12% (4) | 3.09% (3) | 37.11% (36) | 100% (97) |
| SPARK-X | 2% (1) | 76% (38) | 8% (4) | 0 | 4% (2) | 6% (3) | 4% (2) | 100% (50) |
| SpatialDE | 0 | 86.27% (44) | 7.84% (4) | 0 | 3.92% (2) | 1.96% (1) | 0 | 100% (51) |
| Squidpy | 1.79% (1) | 87.5% (49) | 7.14% (4) | 1.79% (1) | 1.79% (1) | 0 | 0 | 100% (56) |

**Table S3 Rectum and endometrium specific pathways.**

| Method | HPA: ID | tissue | p_value | Intersection size | term_size | TPR |
|---|---|---|---|---|---|---|
| SPARK | HPA:0400242 | rectum | 1.22E-10 | 28 | 192 | 0.146 |
| SPARK | HPA:0400241 | rectum | 8.01E-09 | 30 | 260 | 0.115 |
| SPARK | HPA:0400243 | rectum | 8.46E-09 | 20 | 113 | 0.177 |
| SPARK-X | HPA:0400242 | rectum | 1.25E-06 | 25 | 192 | 0.13 |
| SPARK-X | HPA:0400241 | rectum | 2.61E-06 | 29 | 260 | 0.112 |
| SPARK-X | HPA:0400243 | rectum | 8.50E-06 | 18 | 113 | 0.159 |
| SpatialDE | HPA:0400241 | rectum | 0.001901336 | 34 | 260 | 0.131 |
| Squidpy | HPA:0641531 | endometrium | 0.006927202 | 14 | 49 | 0.286 |

**Table S4 Some SVGs results of each method. The p-values were obtained through two-sided tests and adjusted using Holm's method**

| gene | method | rank | p_adj | |
|---|---|---|---|---|
| Calm1 | HEARTSVG | 6 | 0 | *** |
| | scGCO | 1044 | 0.5013 | |
| | SPARK | 25 | 4.64E-15 | *** |
| | SPARK-X | 49 | 4.33E-10 | *** |
| | SpatialDE | 204 | 0 | *** |
| | Squidpy | 337 | 3.96E-12 | *** |
| Calm2 | HEARTSVG | 8 | 0 | *** |
| | scGCO | 1031 | 0.501 | |
| | SPARK | 26 | 4.64E-15 | *** |
| | SPARK-X | 63 | 1.68E-08 | *** |
| | SpatialDE | 292 | 8.35E-14 | *** |
| | Squidpy | 113 | 0 | *** |
| Car8 | HEARTSVG | 66 | 0 | *** |
| | scGCO | 2 | 4.46E-28 | *** |
| | SPARK | 3 | 4.64E-15 | *** |
| | SPARK-X | 585 | 0.050 | |
| | SpatialDE | 37 | 0 | *** |
| | Squidpy | 4 | 0 | *** |
| Itm2b | HEARTSVG | 11 | 0 | *** |
| | scGCO | 1269 | 0.501 | |
| | SPARK | 75 | 4.64E-15 | *** |
| | SPARK-X | 322 | 0.006 | * |
| | SpatialDE | 116 | 0 | *** |
| | Squidpy | 148 | 0 | *** |
| Pcp2 | HEARTSVG | 38 | 0 | *** |
| | scGCO | 5 | 4.46E-28 | *** |
| | SPARK | 110 | 4.64E-15 | *** |
| | SPARK-X | 804 | 0.139 | |
| | SpatialDE | 145 | 0 | *** |
| | Squidpy | 13 | 0 | *** |
| Pcp4 | HEARTSVG | 27 | 0 | *** |
| | scGCO | 4 | 4.46E-28 | *** |
| | SPARK | 5 | 4.64E-15 | *** |
| | SPARK-X | 2485 | 1 | |
| | SpatialDE | 48 | 0 | *** |
| | Squidpy | 3 | 0 | *** |

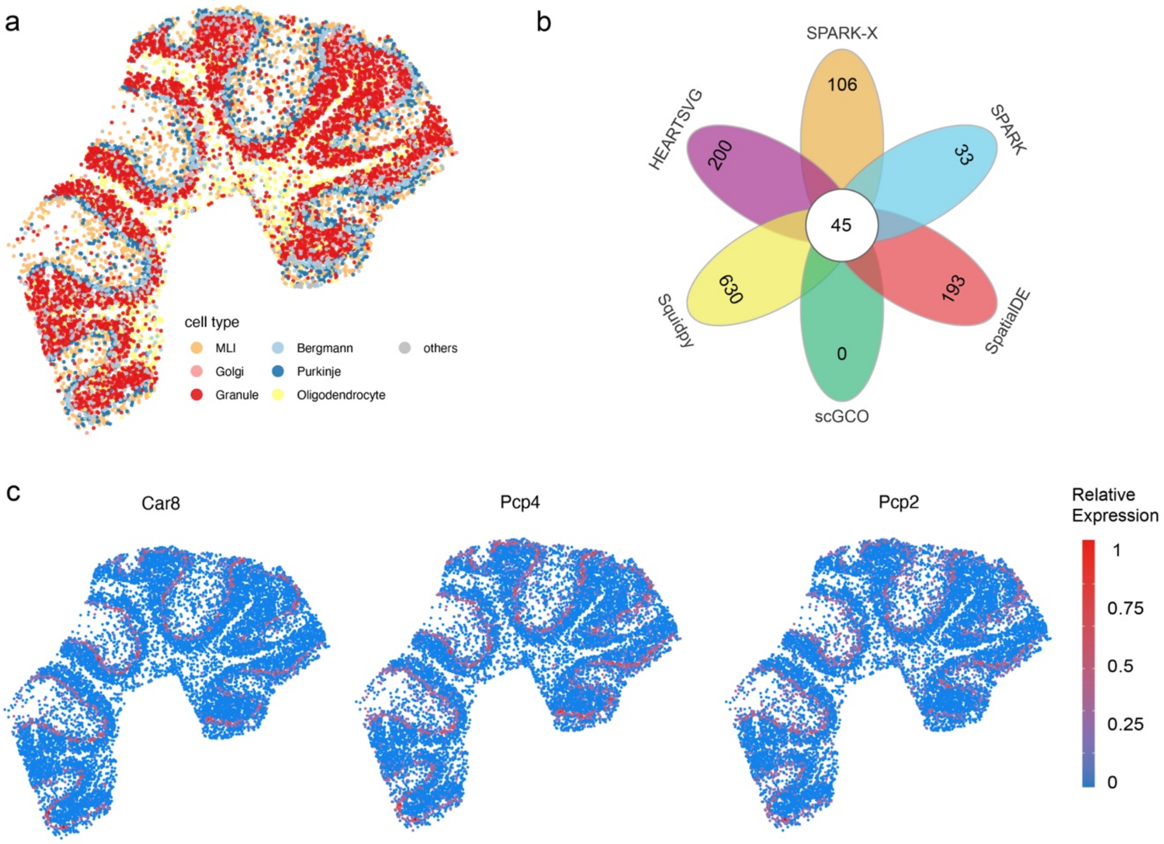


**Figure S21 a,** Visualization of unsupervised spatial clustering results. **b,** Venn diagrams of SVGs in the mouse cerebellum data identified by HEARTSVG, SpatialDE, SPARK, SPARK-X, scGCO and Squidpy. Source data are provided with this paper. **c,** Visualizations of marker genes of Purkinje cells in the mouse cerebellum data by Slide-seqV2. Source data are provided with this paper.

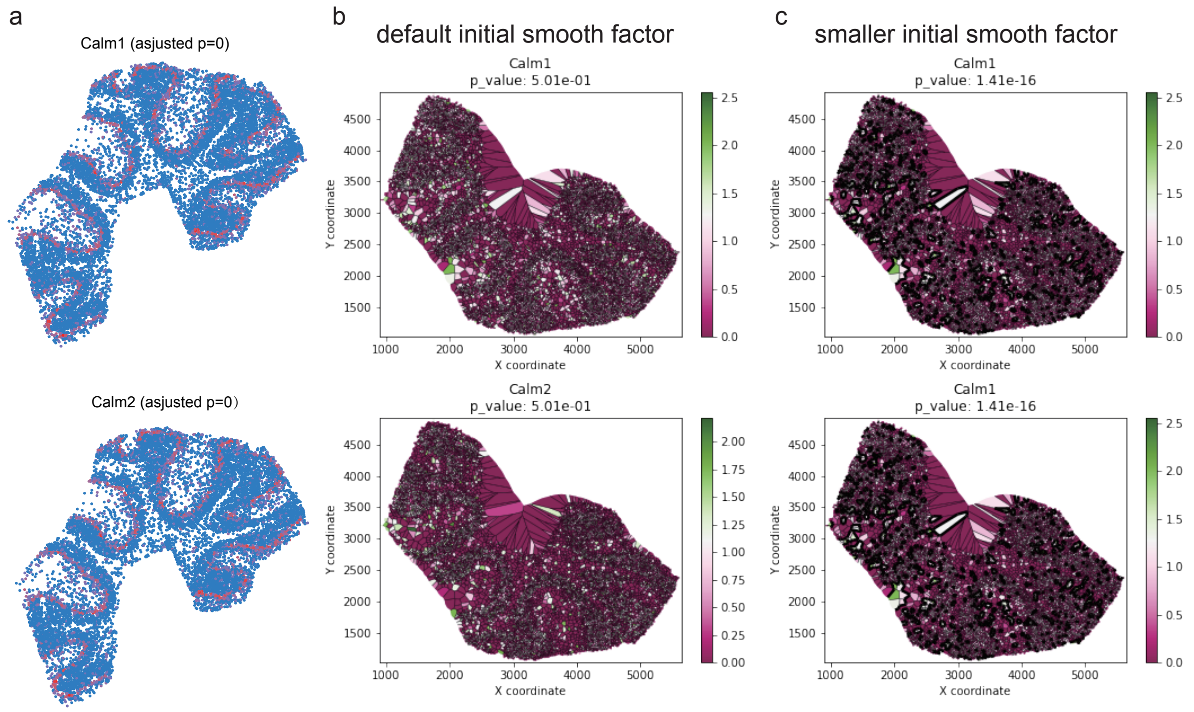


**Figure S22 scGCO missed SVGs (Calm1, Calm2) with clear spatial expression patterns comparing with other methods. a,** Visualizations of spatial expressions of Calm1 and Calm2 in the in the Slide-seqV2 cerebellum data. **b,** Visualizations of graph cuts by scGCO with default initial smooth factor of Calm1 and Calm2. **c,** Visualizations of graph cuts by scGCO with smaller initial smooth factor of Calm1 and Calm2.

## 5. Application to mouse preoptic hypothalamus data by MERFISH

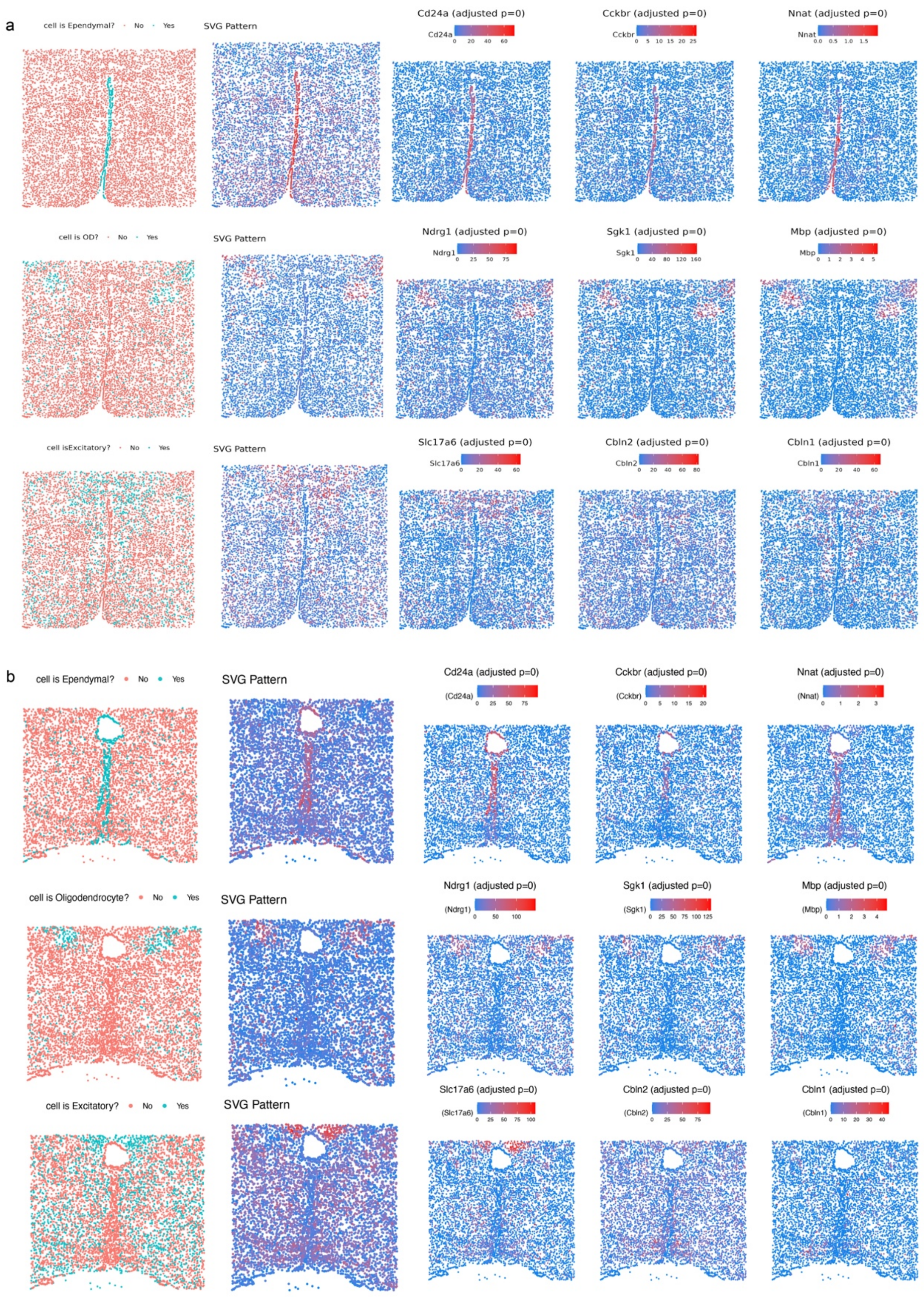


**Figure S22 a,** Visualizations of marker genes and cell type of the MERFISH data 1. **b,** Visualizations of marker genes and cell type of the MERFISH data 2.

## 6. Application to mouse olfactory bulb data by HDST

a

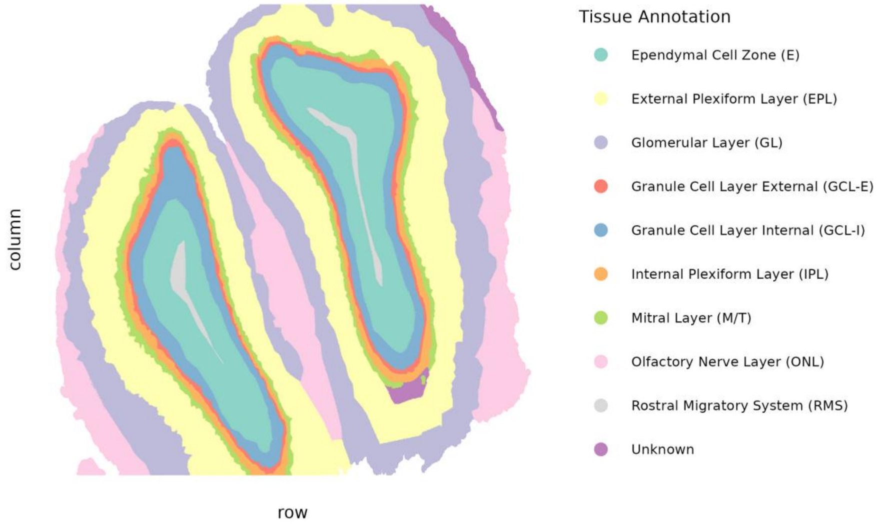


b

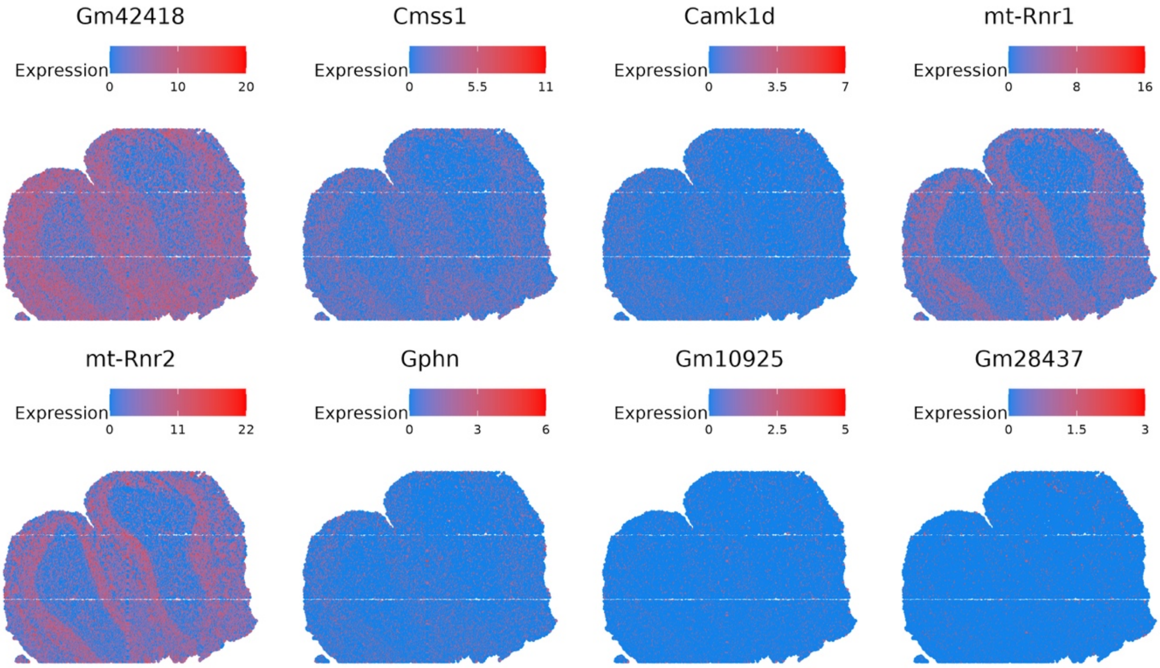


**Figure S23** **a,** Cell annotations of HDST data. **b,** Representative svgs identified by HEARTSVG.

# 7. Application to primary liver cancer data by 10X Visium

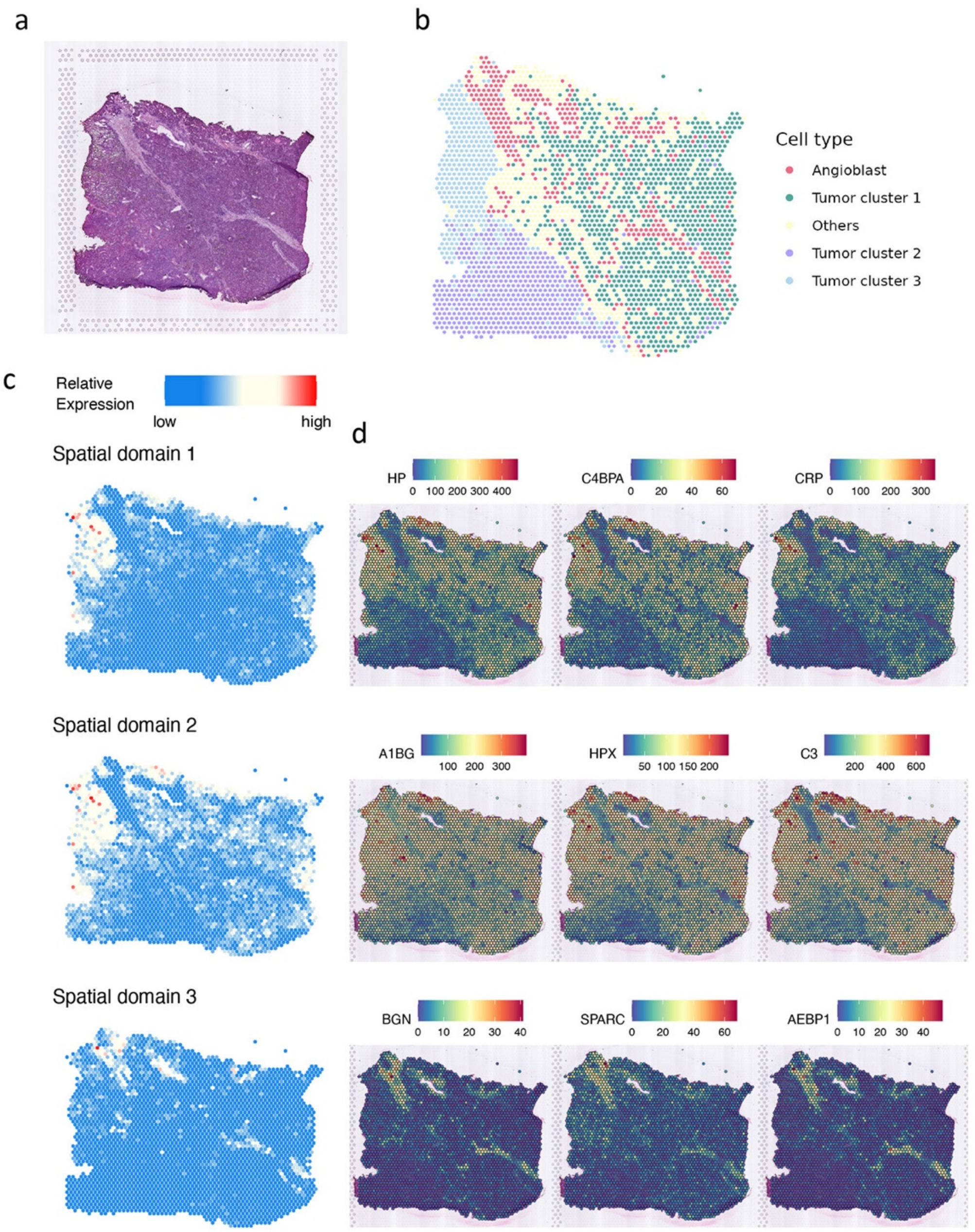


**Figure S24 a,** Original hematoxylin and eosin stained (H&E) tissue image. **b,** Unsupervised spatial clustering results. **c,** SVGs cluster patterns. **d,** Representative genes of six SVG clusters.

# 8. Application to prenal clear cell cancer brain metastasis data by 10X Visium

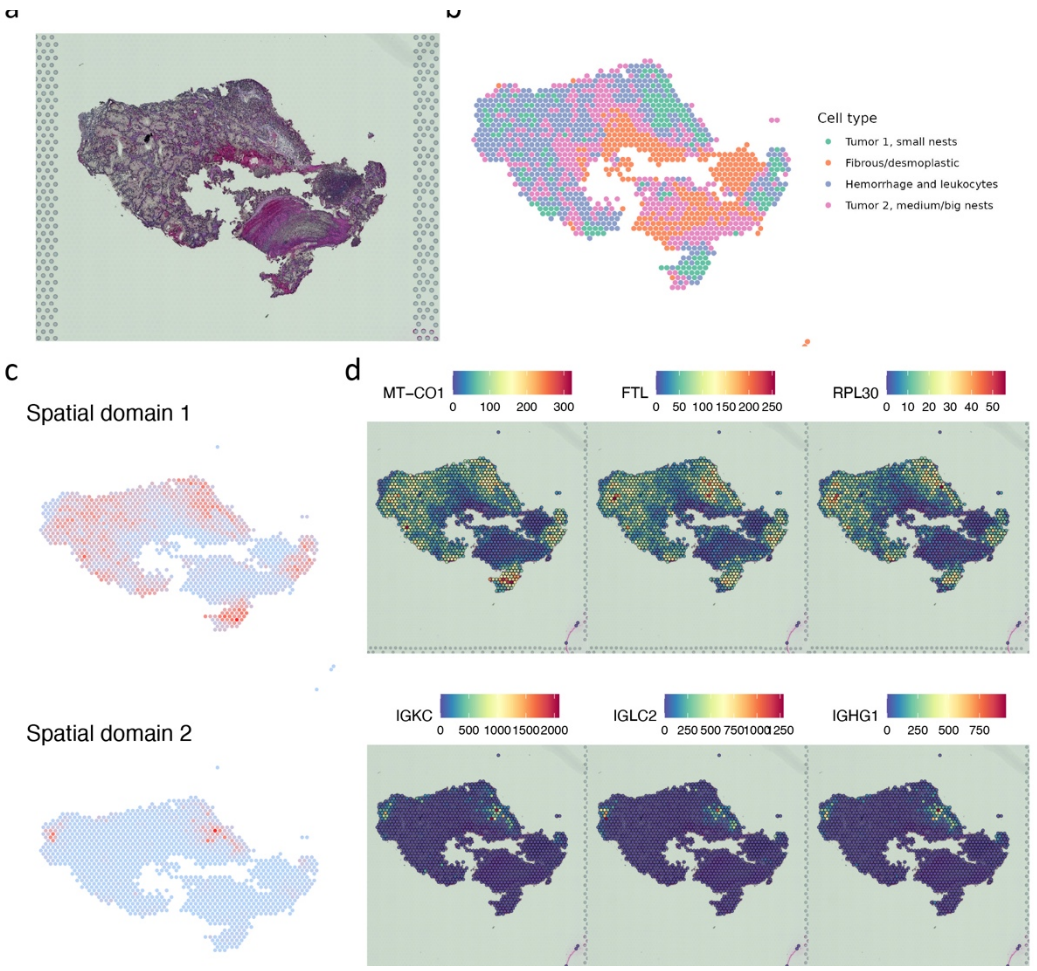


**Figure S25 a,** Original hematoxylin and eosin stained (H&E) tissue image. **b,** Unsupervised spatial clustering results. **c,** SVGs cluster patterns. **d,** Representative genes of six SVG clusters.

# 9. Additional analysis

We applied HEARTSVG to analyze three datasets used in the scGCO study, Mouse olfactory bulb data (MOB data) amd Breast cancer data (BC data) generated by Spatial Transcriptomics technology, and Mouse neuron tissue data generated by LCM technology (LCM data).

### 11.1. Mouse olfactory bulb data (MOB data)

HEARTSVG identified 1610 SVGs in the MOB data. We reproduced scGCO's identification and detected 830 SVGs (in the original paper, it was reported as 796 SVGs).

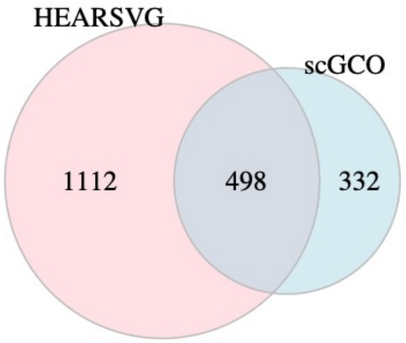


**Figure S26** Venn diagrams of SVGs in the MOB data identified by HEARTSVG, and scGCO.

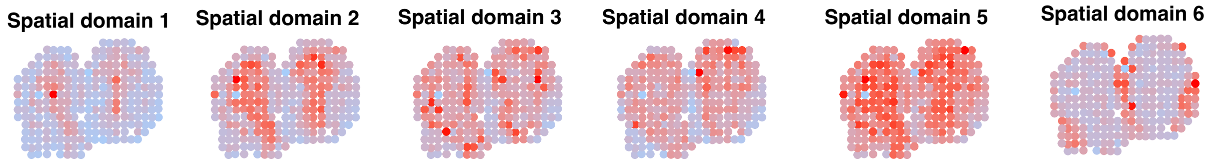


**Figure S27** HEARTSVG predicts spatial domains based on SVGs of the MOB data and graphed the average expression of SVGs in each spatial domain.

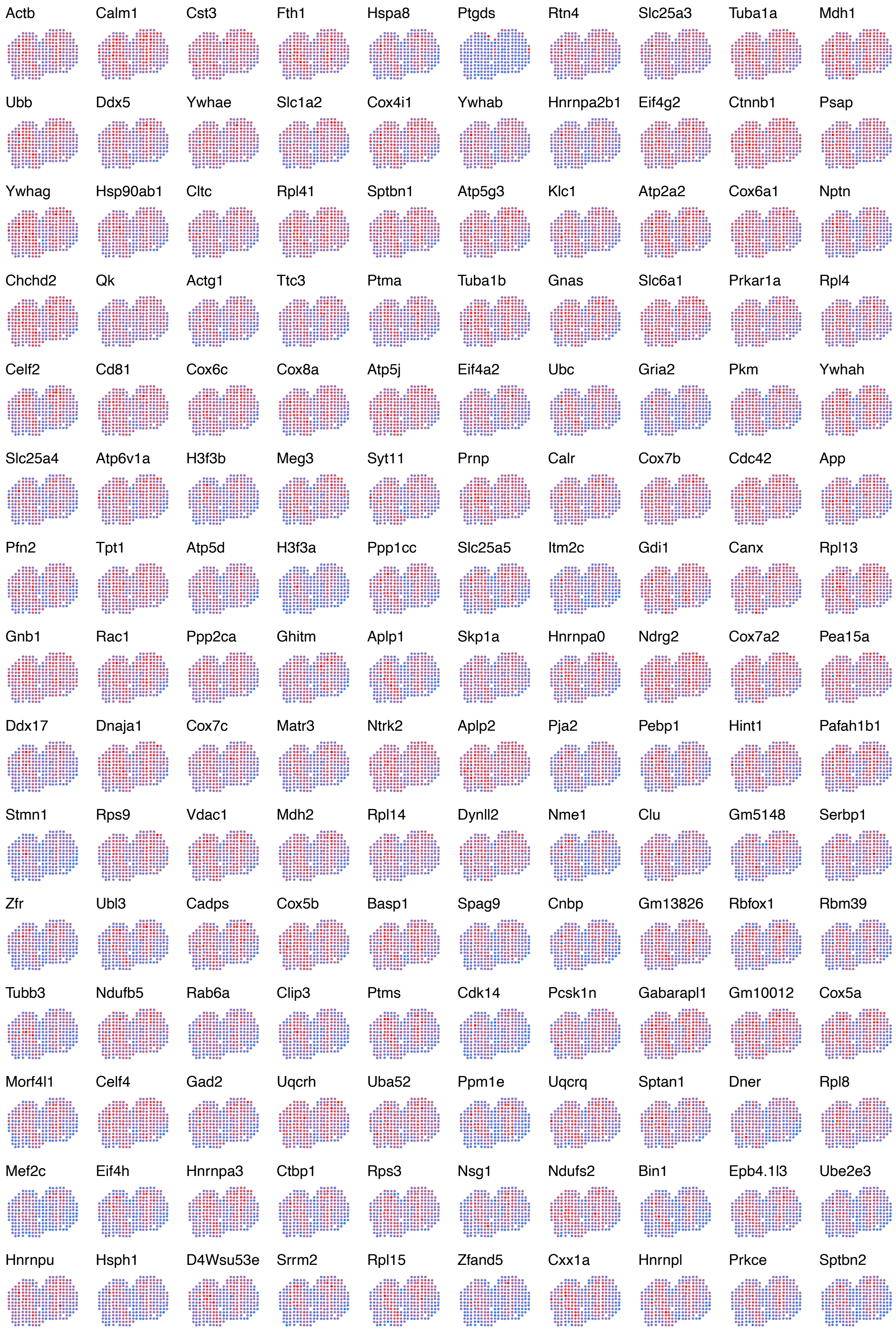


**Figure S28** Top 150 SVGs in the **HEARTSVG-only** SVG list of the MOB data.

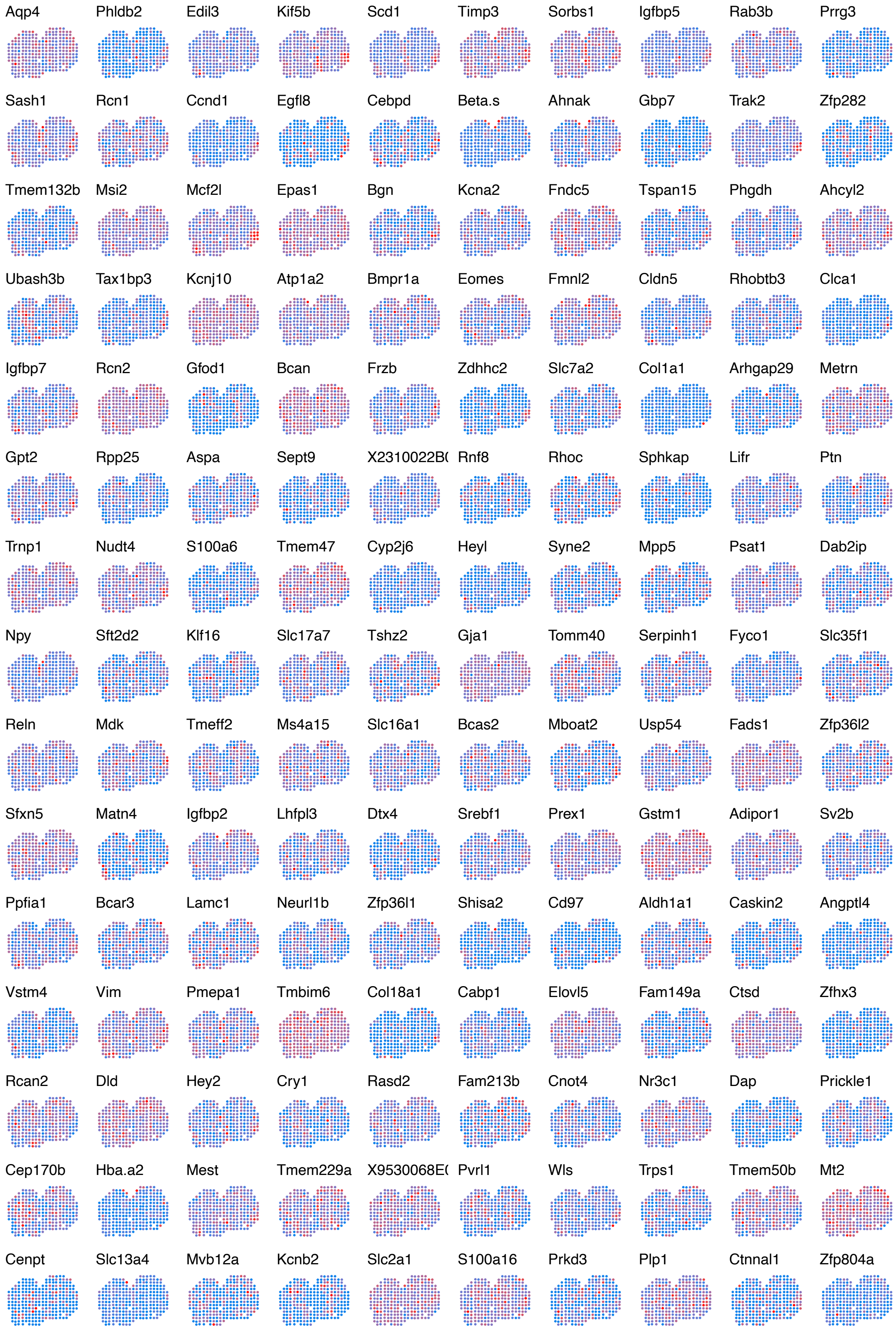


**Figure S29** Top 150 SVGs in the **scGCO-only** SVG list of the MOB data.

### 11.2. Breast cancer data (BC data)

HEARTSVG identified 287 SVGs in the BC data. We reproduced scGCO's identification and detected 330 SVGs (in the original paper, it was reported as 309 SVGs).

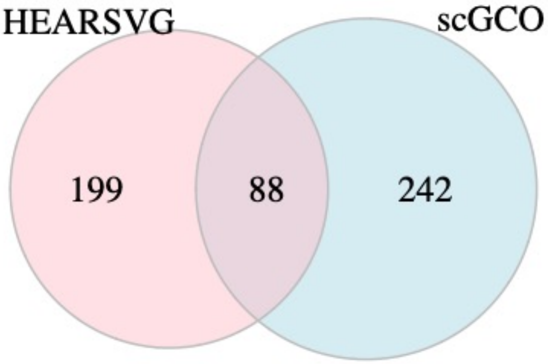


**Figure S30** Venn diagrams of SVGs in the BC data identified by HEARTSVG, and scGCO

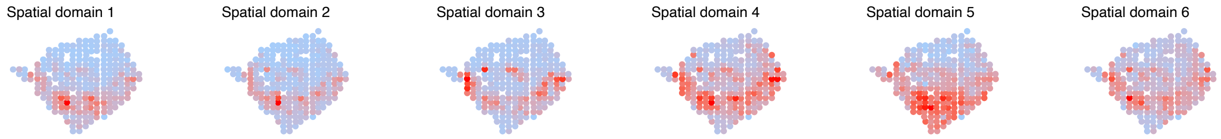


**Figure S31** HEARTSVG predicts spatial domains based on SVGs of the BC data and graphed the average expression of SVGs in each spatial domain.

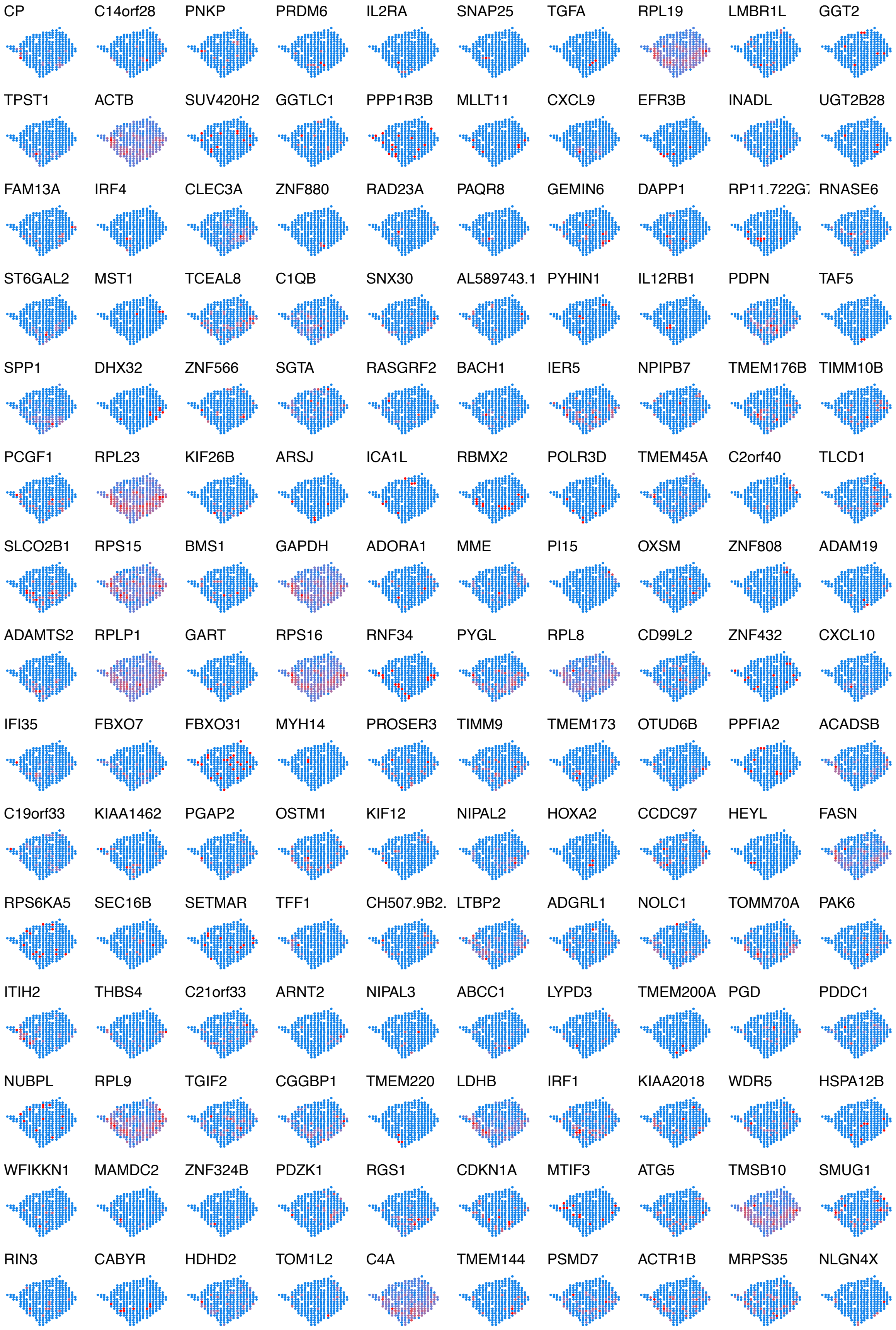


**Figure S32** Top 150 SVGs in the **HEARTSVG-only** SVG list of the BC data.

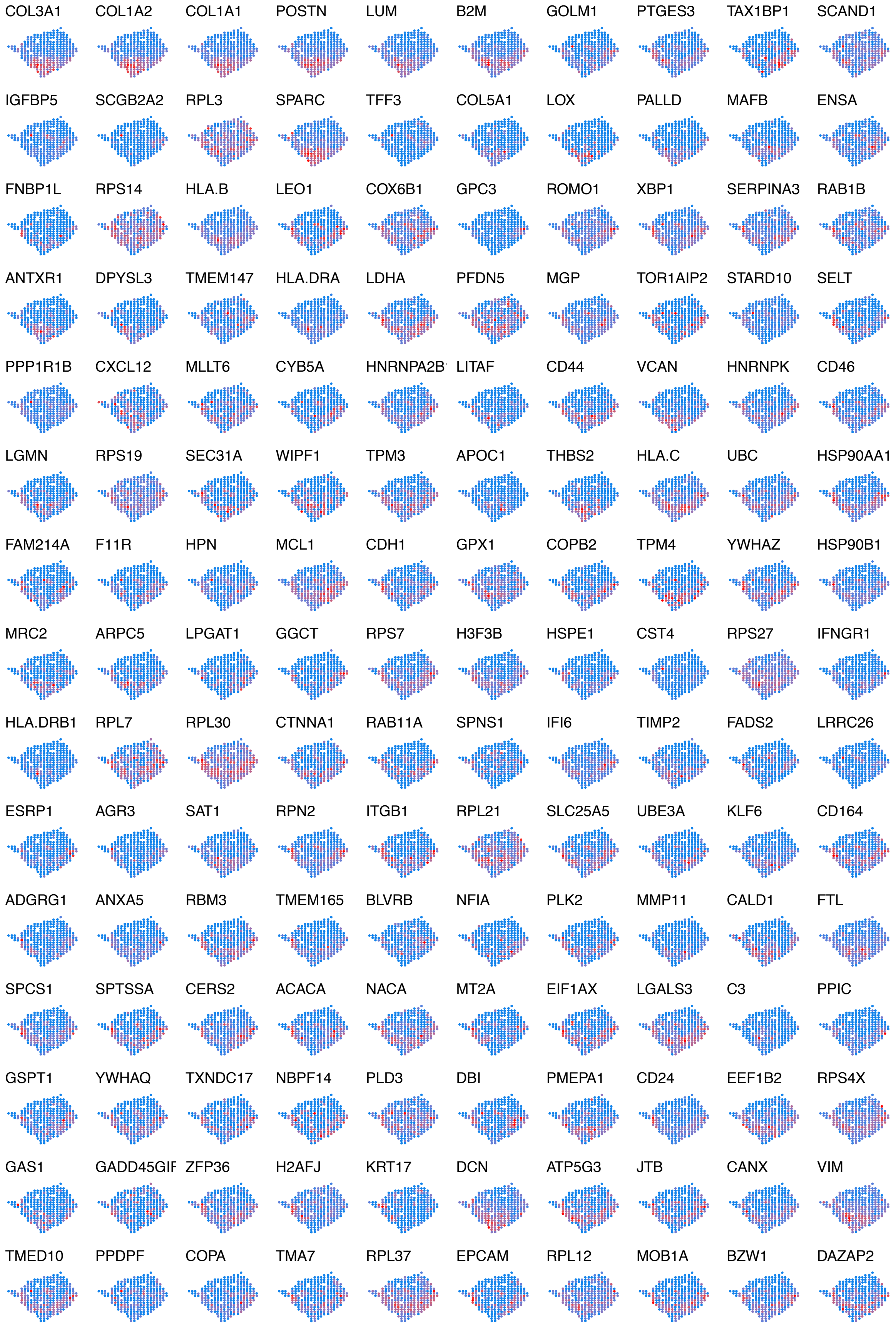


**Figure S33** Top 150 SVGs in the **scGCO-only** SVG list of the BC data.

### 11.3. Mouse neuron tissue data with LCM technology (LCM data)

HEARTSVG identified 420 SVGs in the LCM data. We reproduced scGCO's identification and detected 754 SVGs (in the original paper, it was reported as 3867 SVGs). In our analysis, HEARTSVG and scGCO did not report spike genes as false positives.

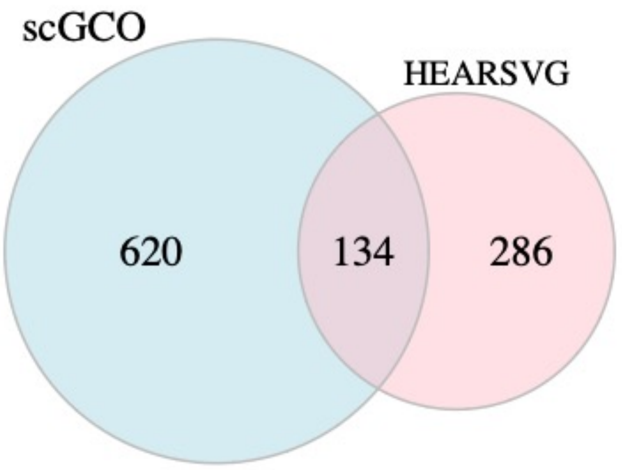


**Figure S34** Venn diagrams of SVGs in the LCM data identified by HEARTSVG, and scGCO

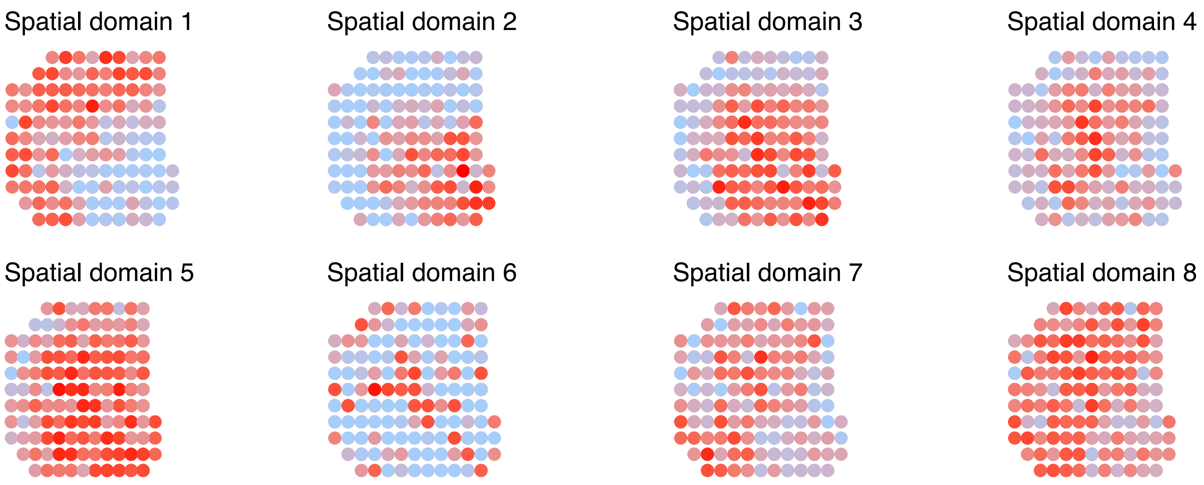


**Figure S35** HEARTSVG predicts spatial domains based on SVGs of the LCM data and graphed the average expression of SVGs in each spatial domain.

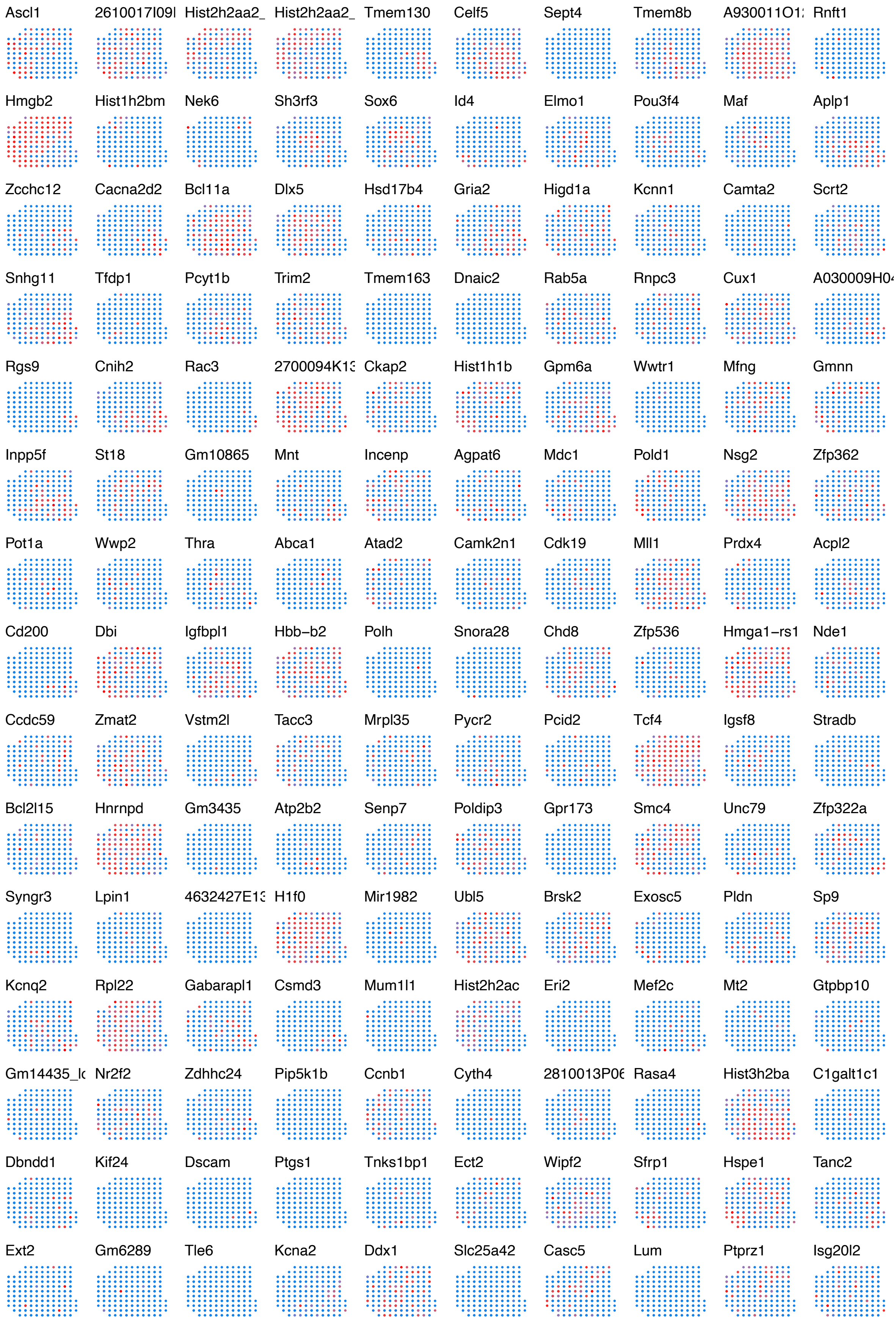


**Figure S36** Top 100 SVGs in the **HEARTSVG-only** SVG list of the LCM data.

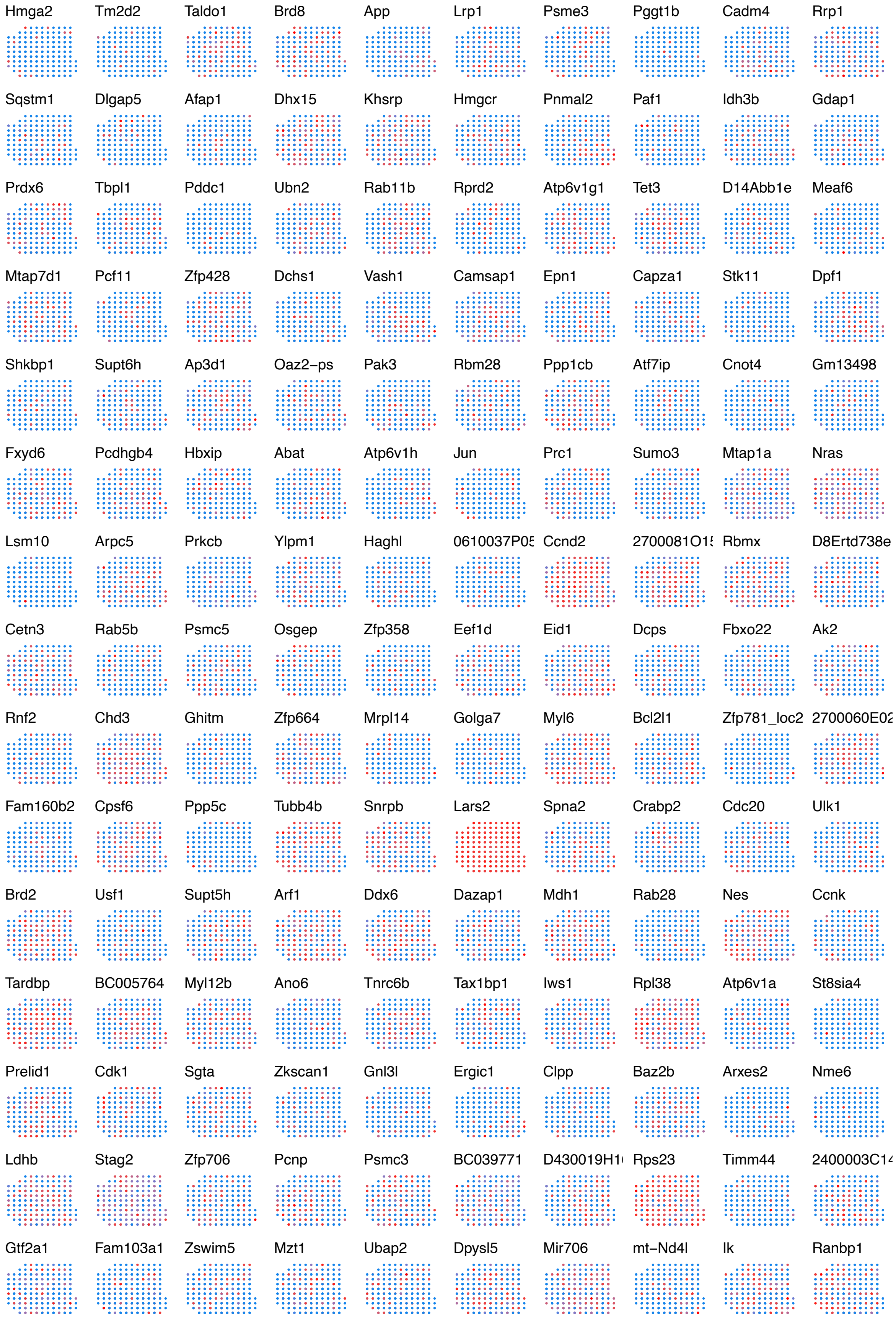


**Figure S37** Top 100 SVGs in the **scGCO-only** SVG list of the LCM data.

# 10. Simulation with noise

## Figures and Tables

**Table S5 Spatial patterns and corresponding proportions of marked area for SVGs(%)**

| Pattern | Proportion of marked area of the SVGs (%) |
|---|---|
| Hotspot | 5 |
| Streak | 5 |
| Gradient | 15 |
| Ring | 15 |
| Nested rings | 15 |
| Streaks | 10 |
| Curve | 7.5 |
| Rectangles | 5 |
| Big triangles | 15 |
| Big circles | 15 |
| Big squares | 15 |
| Small triangles | 7.5 |
| Small circles | 7.5 |
| Small squares | 7.5 |
| Big circles II | 15 |
| Small triangles II | 15 |
| Pattern I | 20 |
| Pattern II | 20 |
| Pattern III | 20 |
| Irreg pat I | 10 |
| Irreg pat II | 5 |
| Irreg pat III | 5 |

### 10.1 Results of simulations with mixture noise

For simulated data with mixture noise, we generated 1000 simulated SVGs and randomly rearranged the gene expressions to generate non-SVGs. Then, we mix their expression to create non-SVGs, SVGs with noise, and non-SVGs with noise.

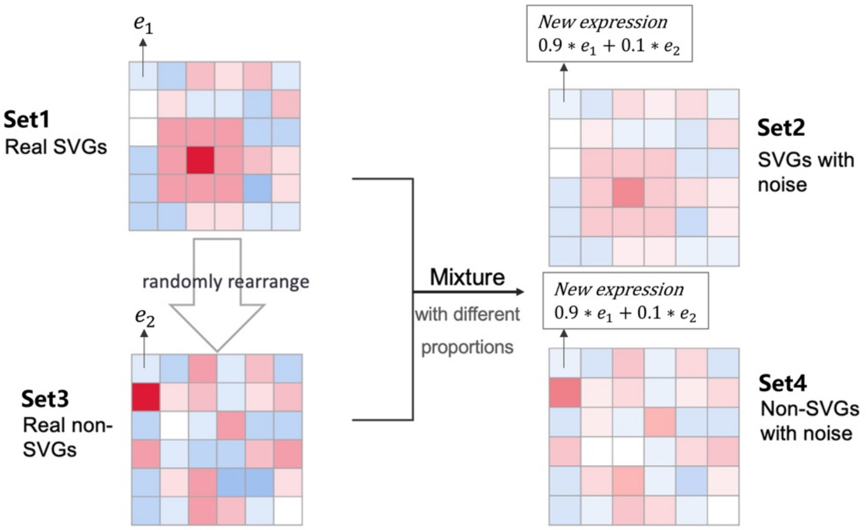


**Figure S38** The schematic of simulated data with mixture noise. Set 1 represents the true SVGs set, derived by selecting the top 500 overlaps from the results of all six methods. Set 3 is the non-SVGs set, created by randomly rearranging gene expressions within Set 1. Set 2 and Set 4 are generated by using a mixture of SVGs and non-SVGs to simulate data with noise.

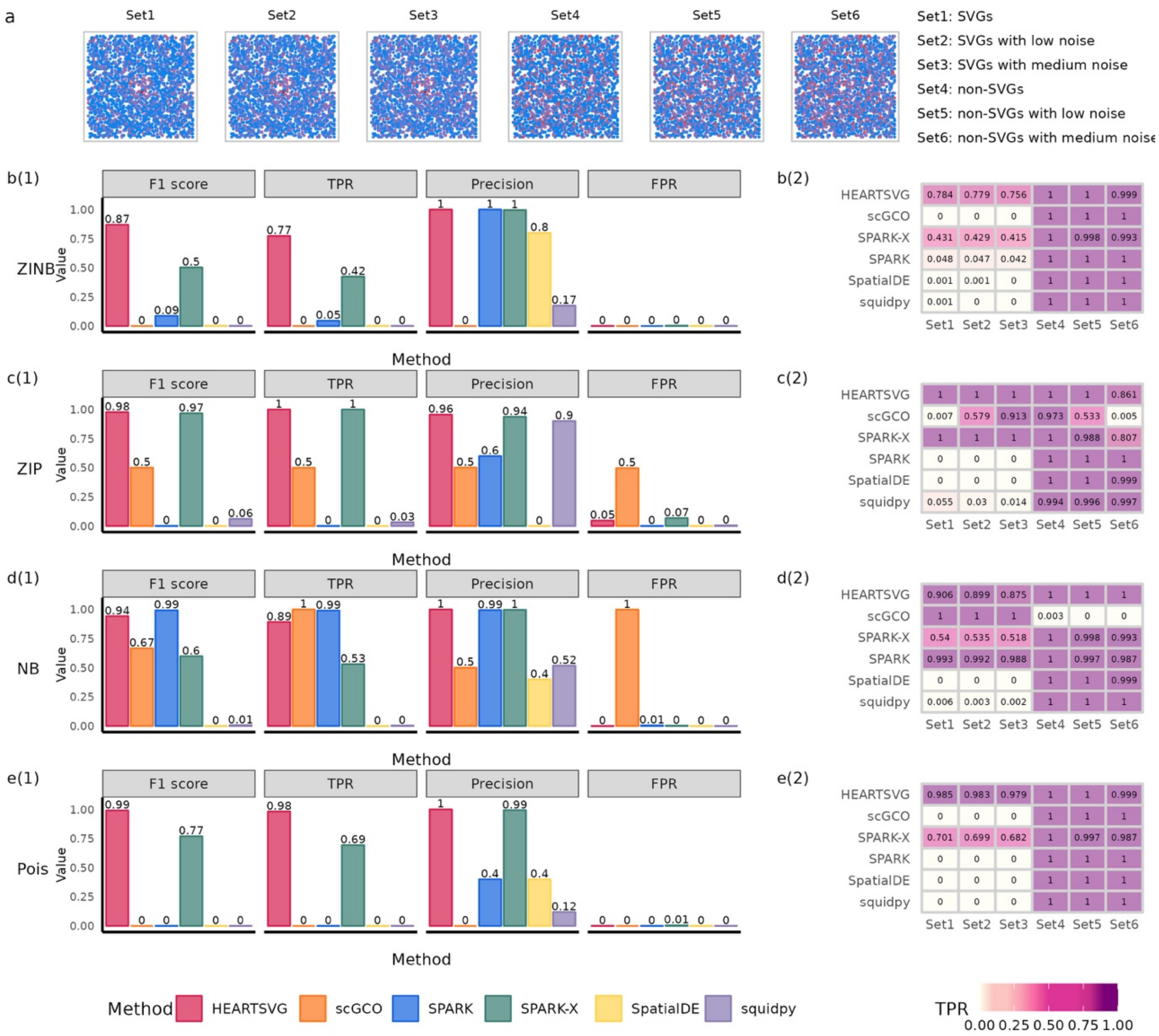

**Figure S39** Simulation results of SVGs identification using simulated data with mixture noise. **a,** Visualization of Pattern: Hotspot with mixture noise. **b-e,** Simulation results of six different methods (HEARTSVG, scGCO, SPARK, SPARK-X, Squidpy) on simulated data generated by four distinct distributions (ZINB, ZIP, NB, Poisson). The bar diagram (sub-panel (1)) shows F1 scores, TPRs, precisions, and FPRs. The heatmap (sub-panel (2)) depicts the comparison of TPR values among six genesets. Source data are provided with this paper.

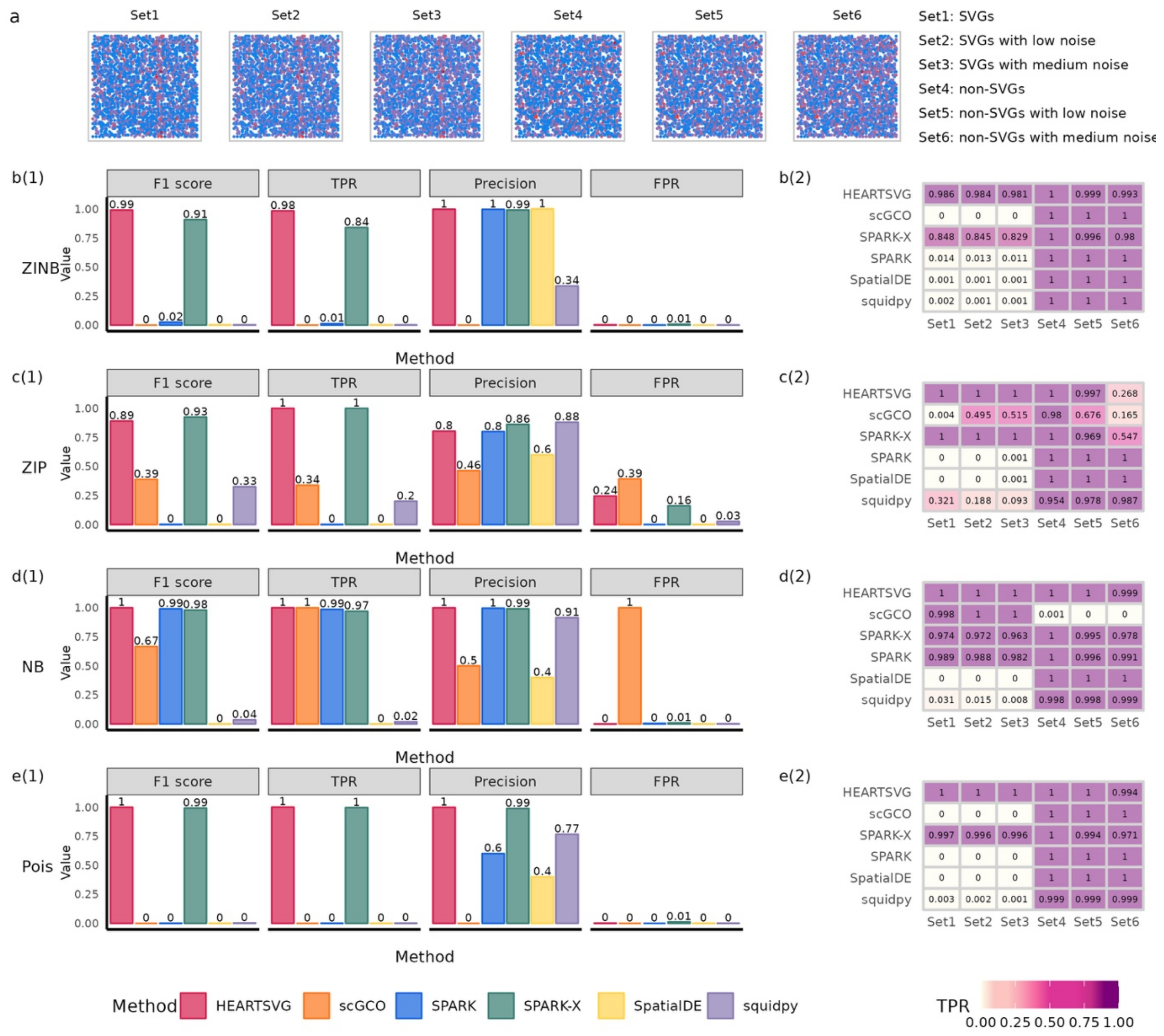


**Figure S40** Simulation results of SVGs identification using simulated data with mixture noise. **a,** Visualization of Pattern: Streak with mixture noise. **b-e,** Simulation results of six different methods (HEARTSVG, scGCO, SPARK, SPARK-X, Squidpy) on simulated data generated by four distinct distributions (ZINB, ZIP, NB, Poisson). The bar diagram (sub-panel (1)) shows F1 scores, TPRs, precisions, and FPRs. The heatmap (sub-panel (2)) depicts the comparison of TPR values among six genesets. Source data are provided with this paper.

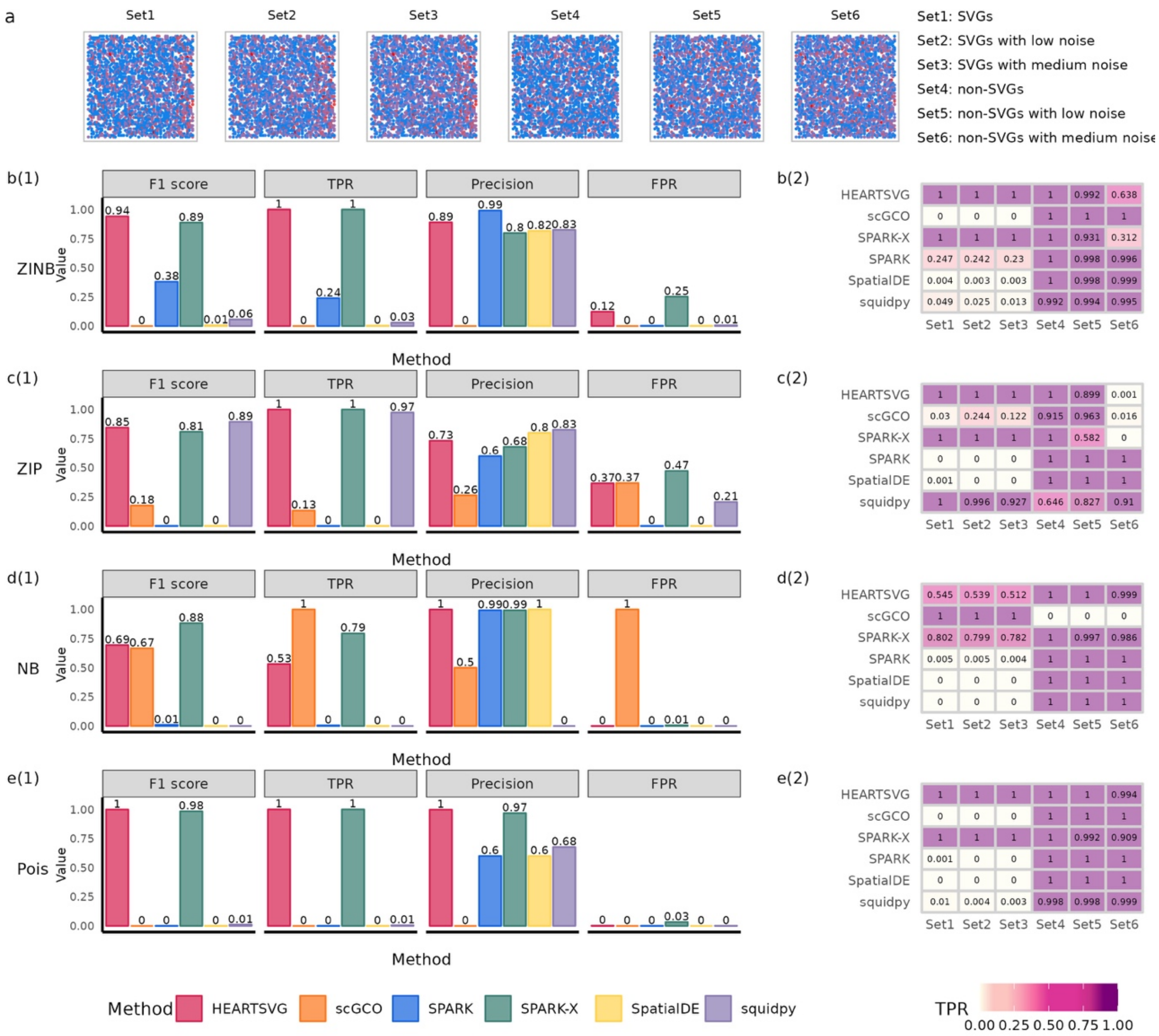


**Figure S41** Simulation results of SVGs identification using simulated data with mixture noise. **a,** Visualization of Pattern: Gradient with mixture noise. **b-e,** Simulation results of six different methods (HEARTSVG, scGCO, SPARK, SPARK-X, Squidpy) on simulated data generated by four distinct distributions (ZINB, ZIP, NB, Poisson). The bar diagram (sub-panel (1)) shows F1 scores, TPRs, precisions, and FPRs. The heatmap (sub-panel (2)) depicts the comparison of TPR values among six genesets. Source data are provided with this paper.

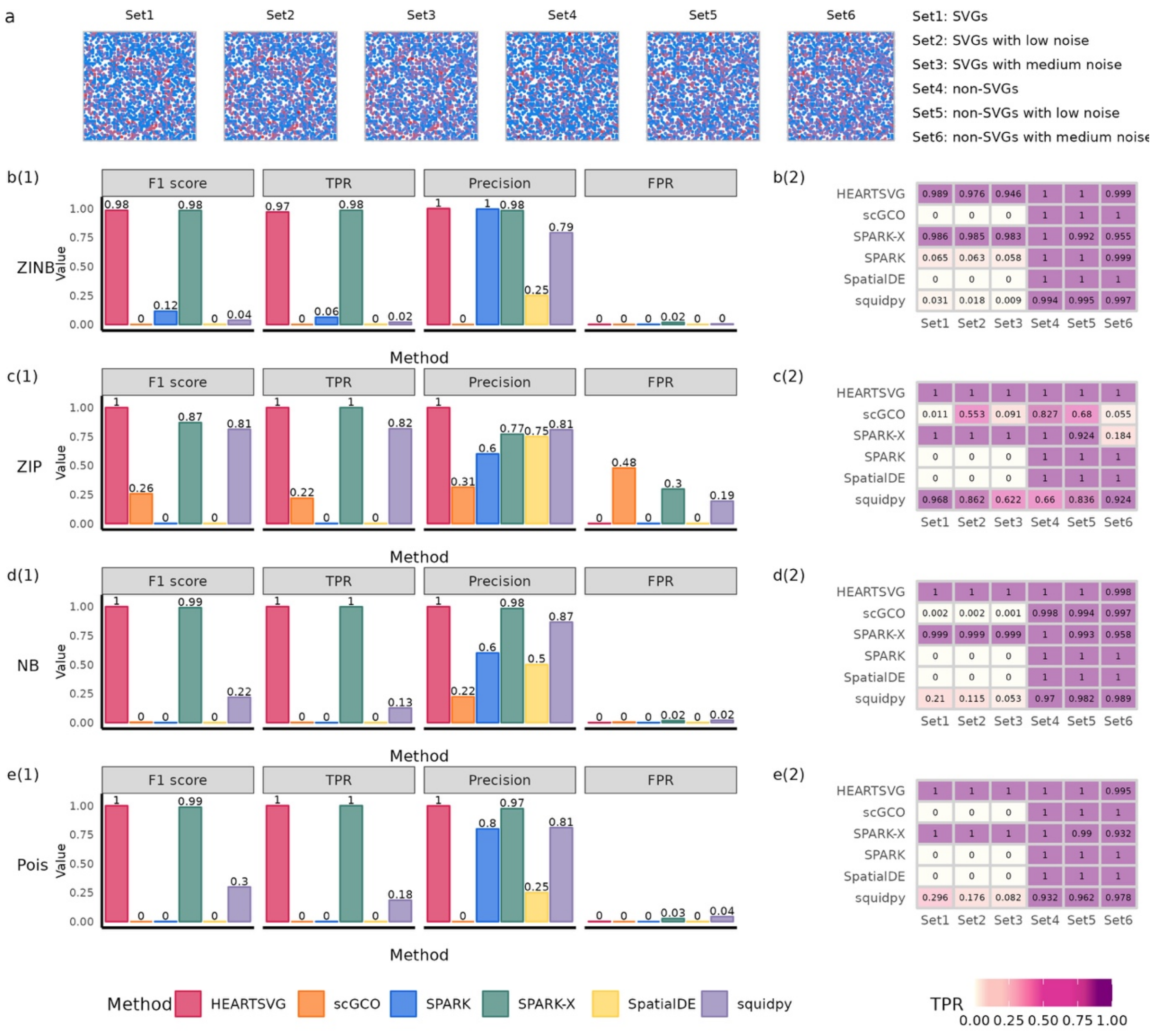


**Figure S42** Simulation results of SVGs identification using simulated data with mixture noise. **a,** Visualization of Pattern: Ring with mixture noise. **b-e,** Simulation results of six different methods (HEARTSVG, scGCO, SPARK, SPARK-X, Squidpy) on simulated data generated by four distinct distributions (ZINB, ZIP, NB, Poisson). The bar diagram (sub-panel (1)) shows F1 scores, TPRs, precisions, and FPRs. The heatmap (sub-panel (2)) depicts the comparison of TPR values among six genesets. Source data are provided with this paper.

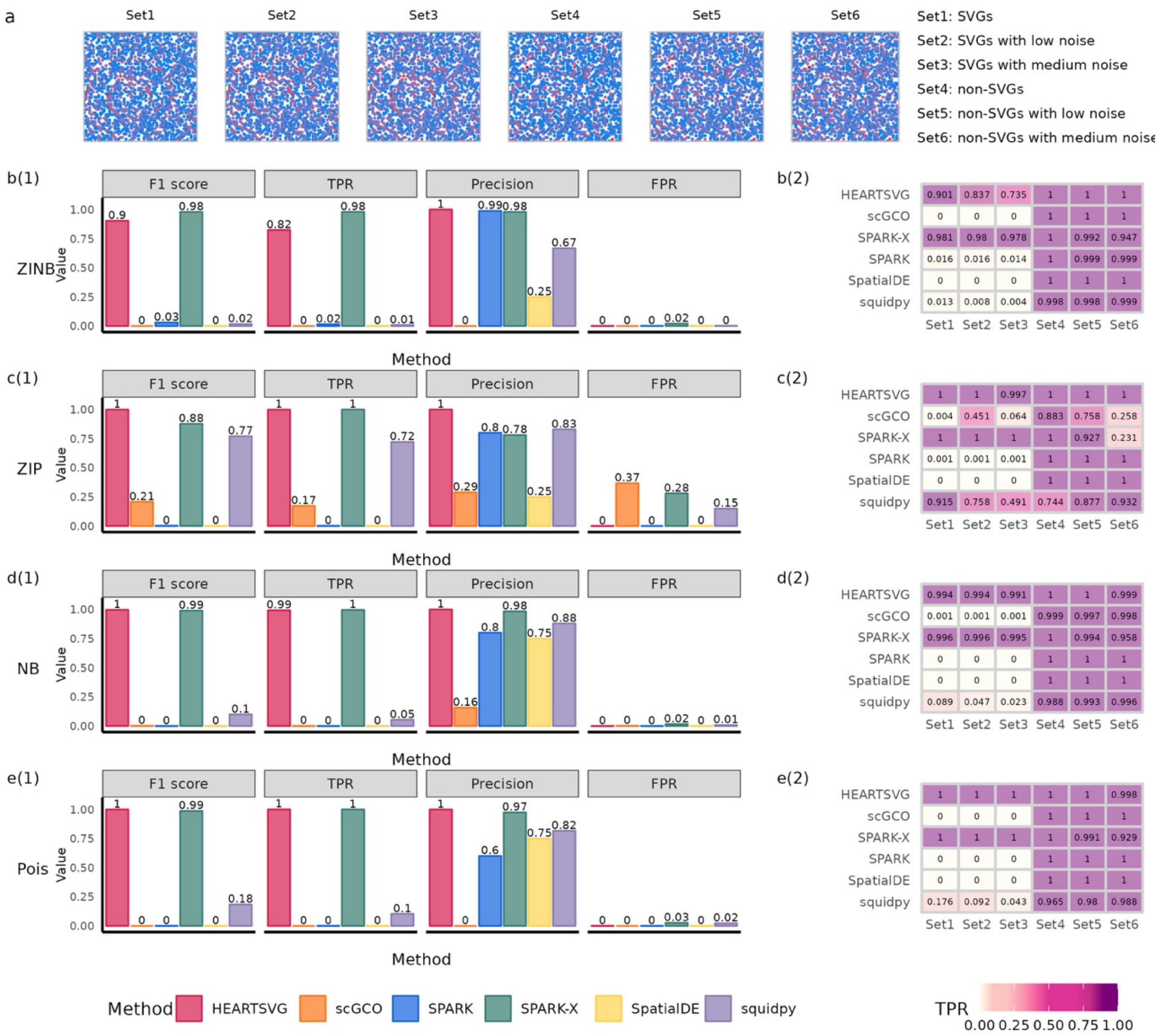


**Figure S43** Simulation results of SVGs identification using simulated data with mixture noise. **a,** Visualization of Pattern: Nested rings with mixture noise. **b-e,** Simulation results of six different methods (HEARTSVG, scGCO, SPARK, SPARK-X, Squidpy) on simulated data generated by four distinct distributions (ZINB, ZIP, NB, Poisson). The bar diagram (sub-panel (1)) shows F1 scores, TPRs, precisions, and FPRs. The heatmap (sub-panel (2)) depicts the comparison of TPR values among six genesets. Source data are provided with this paper.

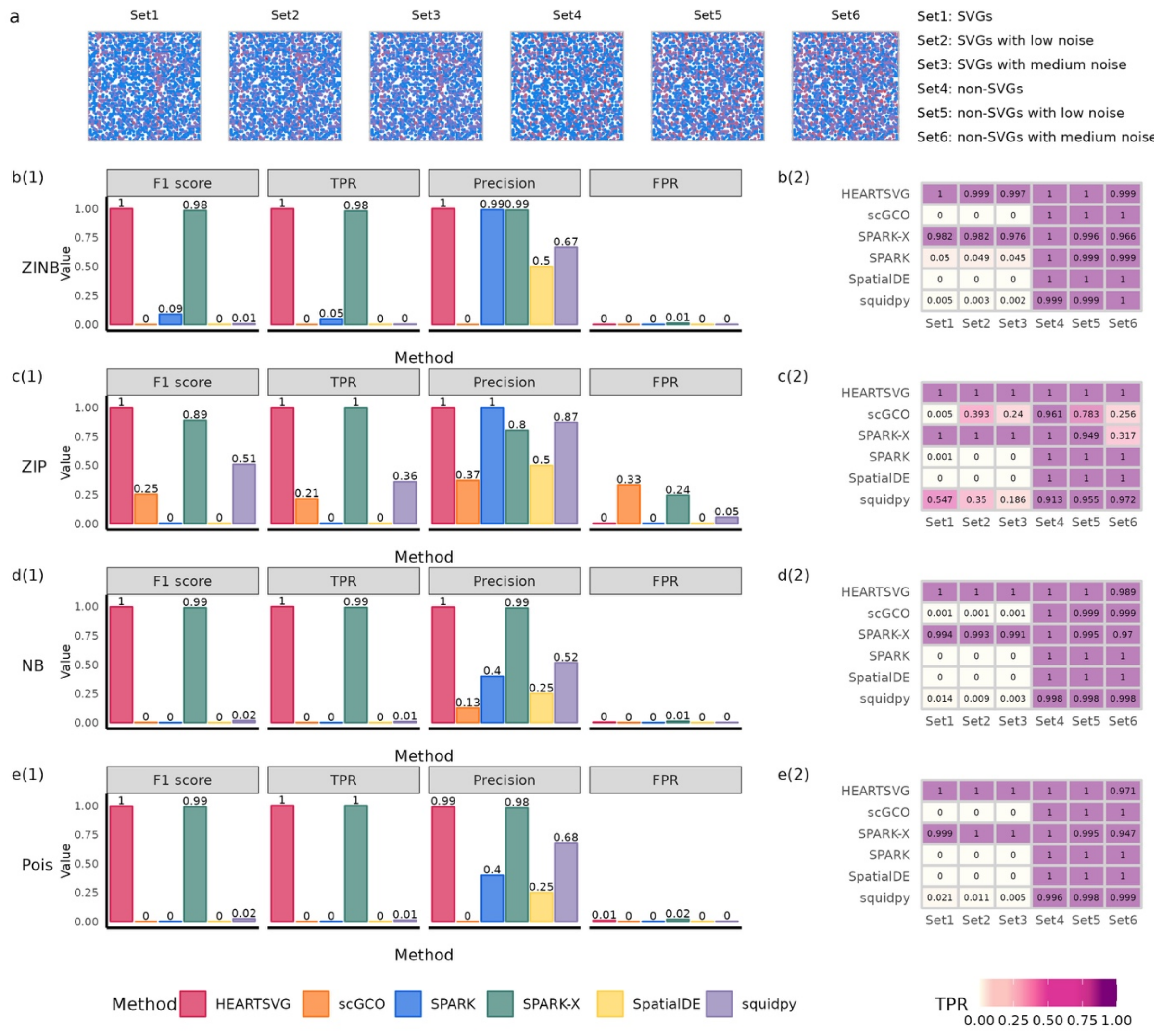

**Figure S44** Simulation results of SVGs identification using simulated data with mixture noise. **a,** Visualization of Pattern: Streaks with mixture noise. **b-e,** Simulation results of six different methods (HEARTSVG, scGCO, SPARK, SPARK-X, Squidpy) on simulated data generated by four distinct distributions (ZINB, ZIP, NB, Poisson). The bar diagram (sub-panel (1)) shows F1 scores, TPRs, precisions, and FPRs. The heatmap (sub-panel (2)) depicts the comparison of TPR values among six genesets. Source data are provided with this paper.

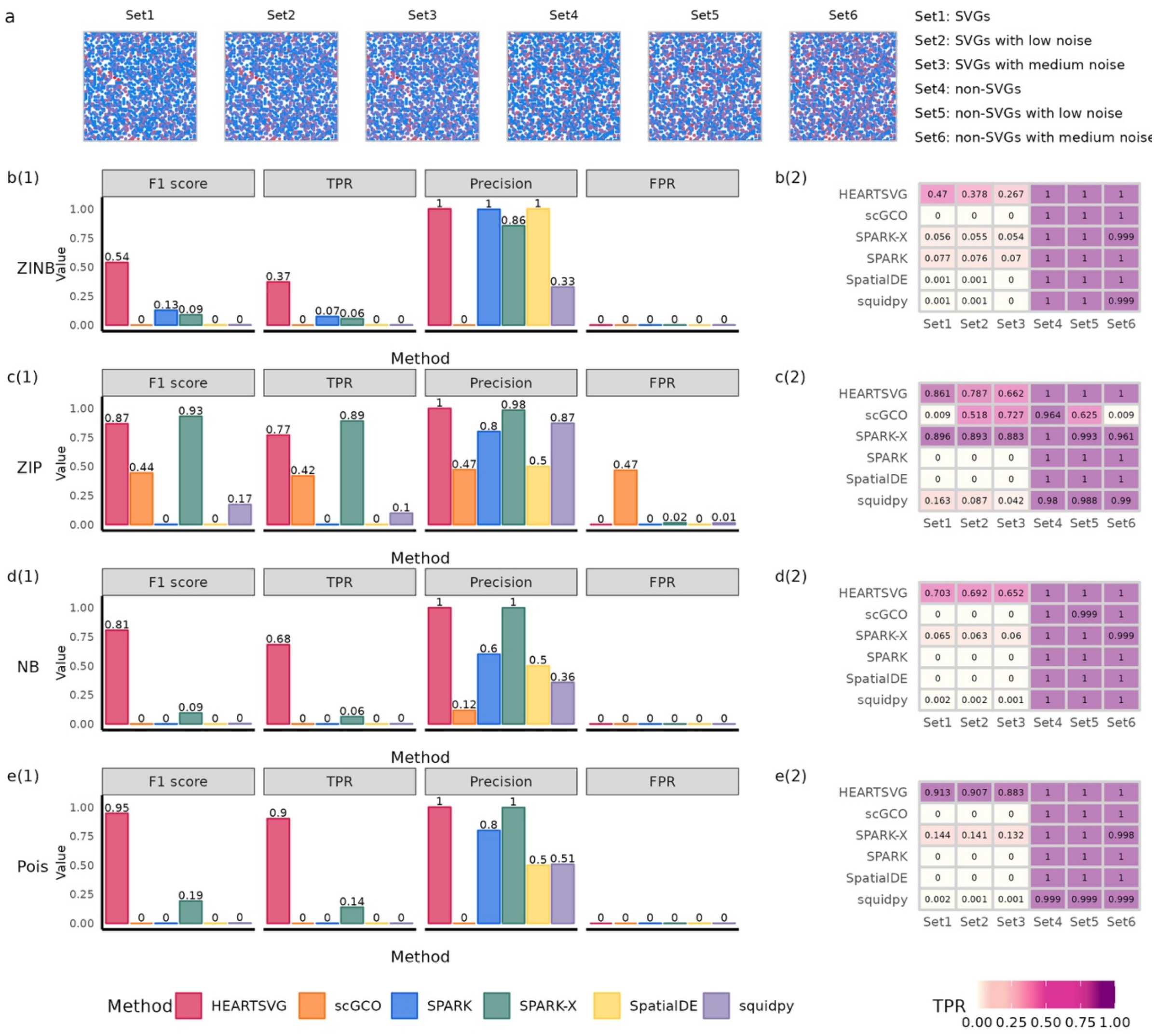


**Figure S45** Simulation results of SVGs identification using simulated data with mixture noise. **a,** Visualization of Pattern: Curve with mixture noise. **b-e,** Simulation results of six different methods (HEARTSVG, scGCO, SPARK, SPARK-X, Squidpy) on simulated data generated by four distinct distributions (ZINB, ZIP, NB, Poisson). The bar diagram (sub-panel (1)) shows F1 scores, TPRs, precisions, and FPRs. The heatmap (sub-panel (2)) depicts the comparison of TPR values among six genesets. Source data are provided with this paper.

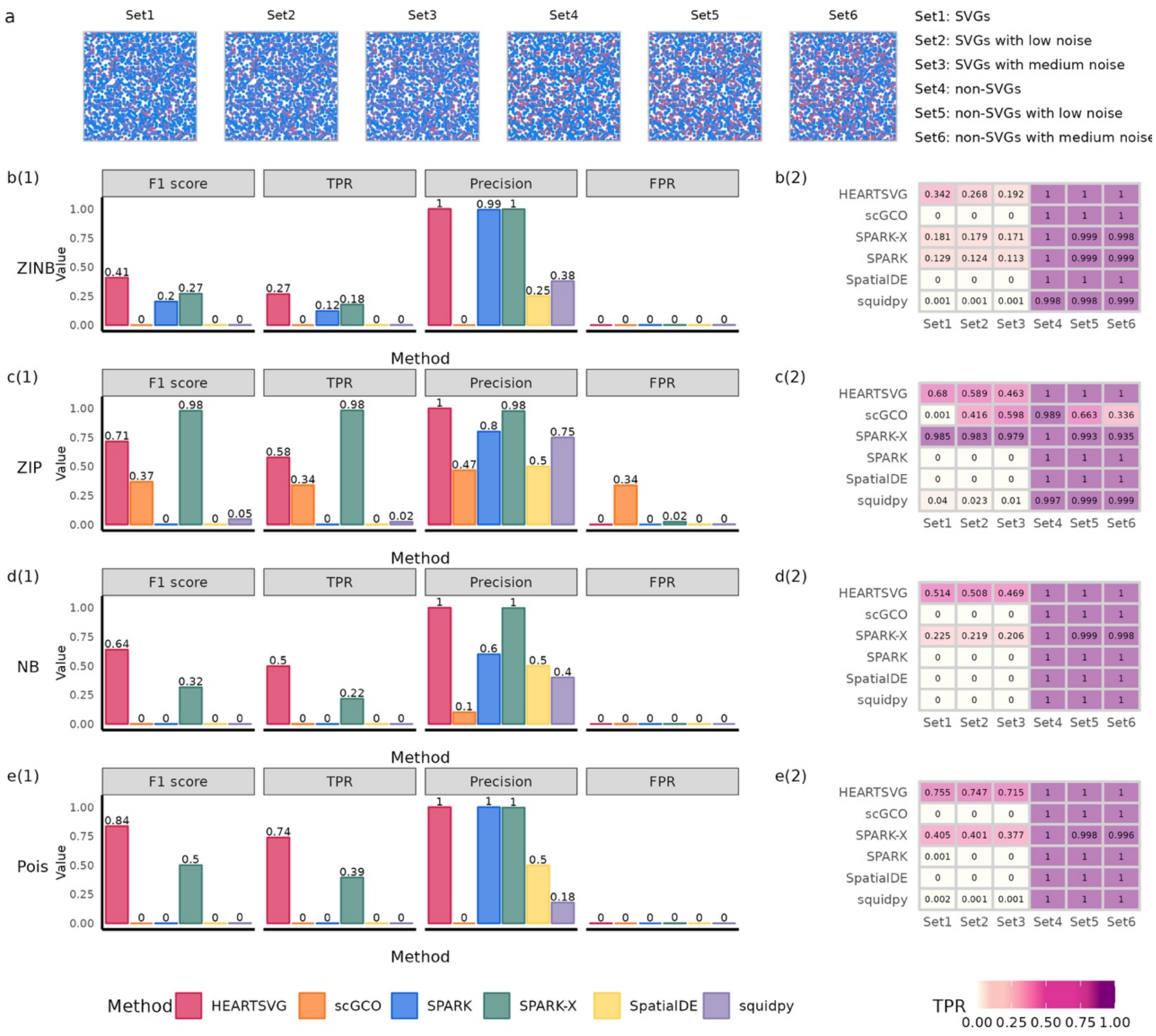


**Figure S46** Simulation results of SVGs identification using simulated data with mixture noise. **a,** Visualization of Pattern: Rectangles with mixture noise. **b-e,** Simulation results of six different methods (HEARTSVG, scGCO, SPARK, SPARK-X, Squidpy) on simulated data generated by four distinct distributions (ZINB, ZIP, NB, Poisson). The bar diagram (sub-panel (1)) shows F1 scores, TPRs, precisions, and FPRs. The heatmap (sub-panel (2)) depicts the comparison of TPR values among six genesets. Source data are provided with this paper.

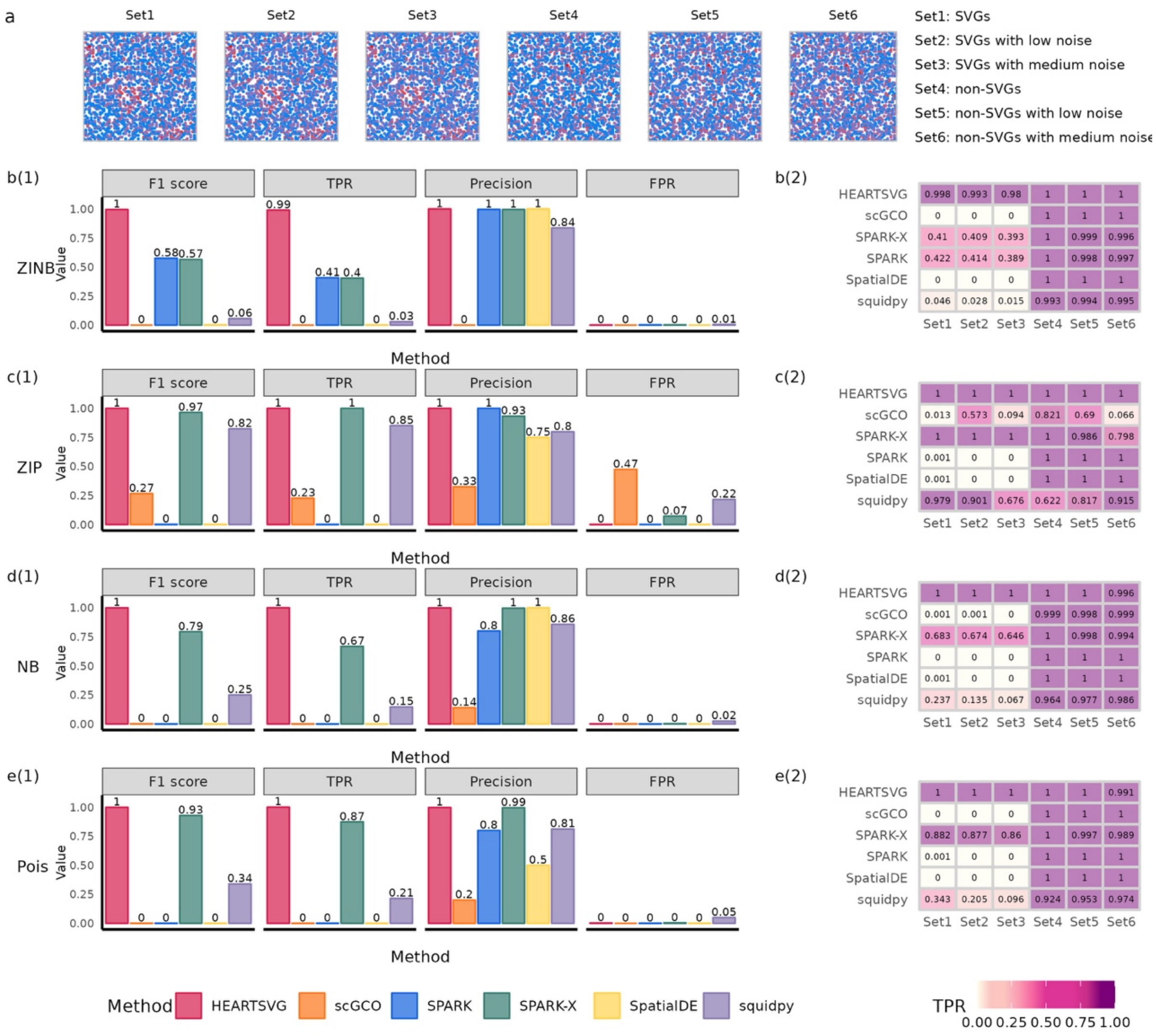


**Figure S47** Simulation results of SVGs identification using simulated data with mixture noise. **a,** Visualization of Pattern: Big triangles with mixture noise. **b-e,** Simulation results of six different methods (HEARTSVG, scGCO, SPARK, SPARK-X, Squidpy) on simulated data generated by four distinct distributions (ZINB, ZIP, NB, Poisson). The bar diagram (sub-panel (1)) shows F1 scores, TPRs, precisions, and FPRs. The heatmap (sub-panel (2)) depicts the comparison of TPR values among six genesets. Source data are provided with this paper.

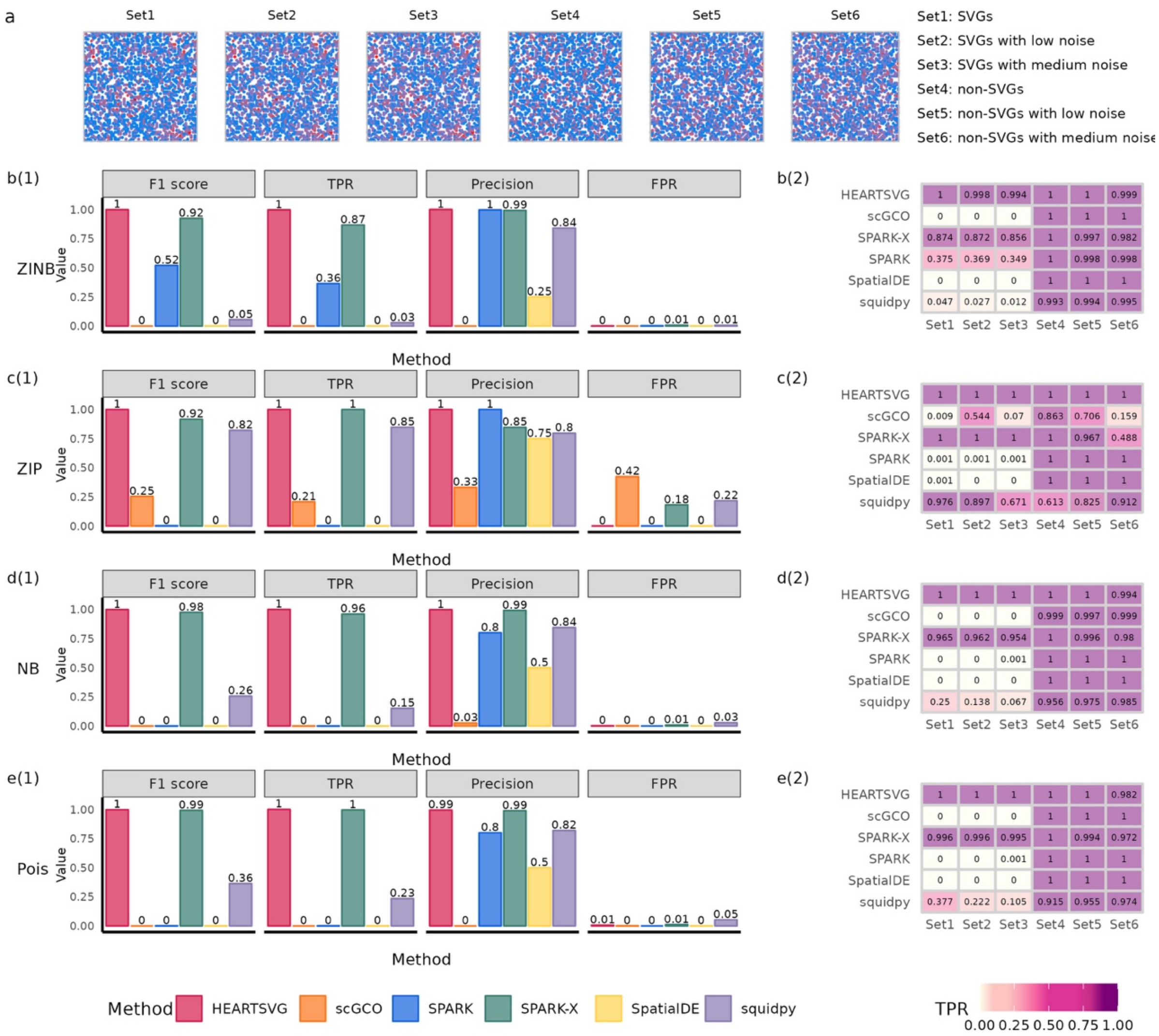


**Figure S48** Simulation results of SVGs identification using simulated data with mixture noise. **a,** Visualization of Pattern: Big circles with mixture noise. **b-e,** Simulation results of six different methods (HEARTSVG, scGCO, SPARK, SPARK-X, Squidpy) on simulated data generated by four distinct distributions (ZINB, ZIP, NB, Poisson). The bar diagram (sub-panel (1)) shows F1 scores, TPRs, precisions, and FPRs. The heatmap (sub-panel (2)) depicts the comparison of TPR values among six genesets. Source data are provided with this paper.

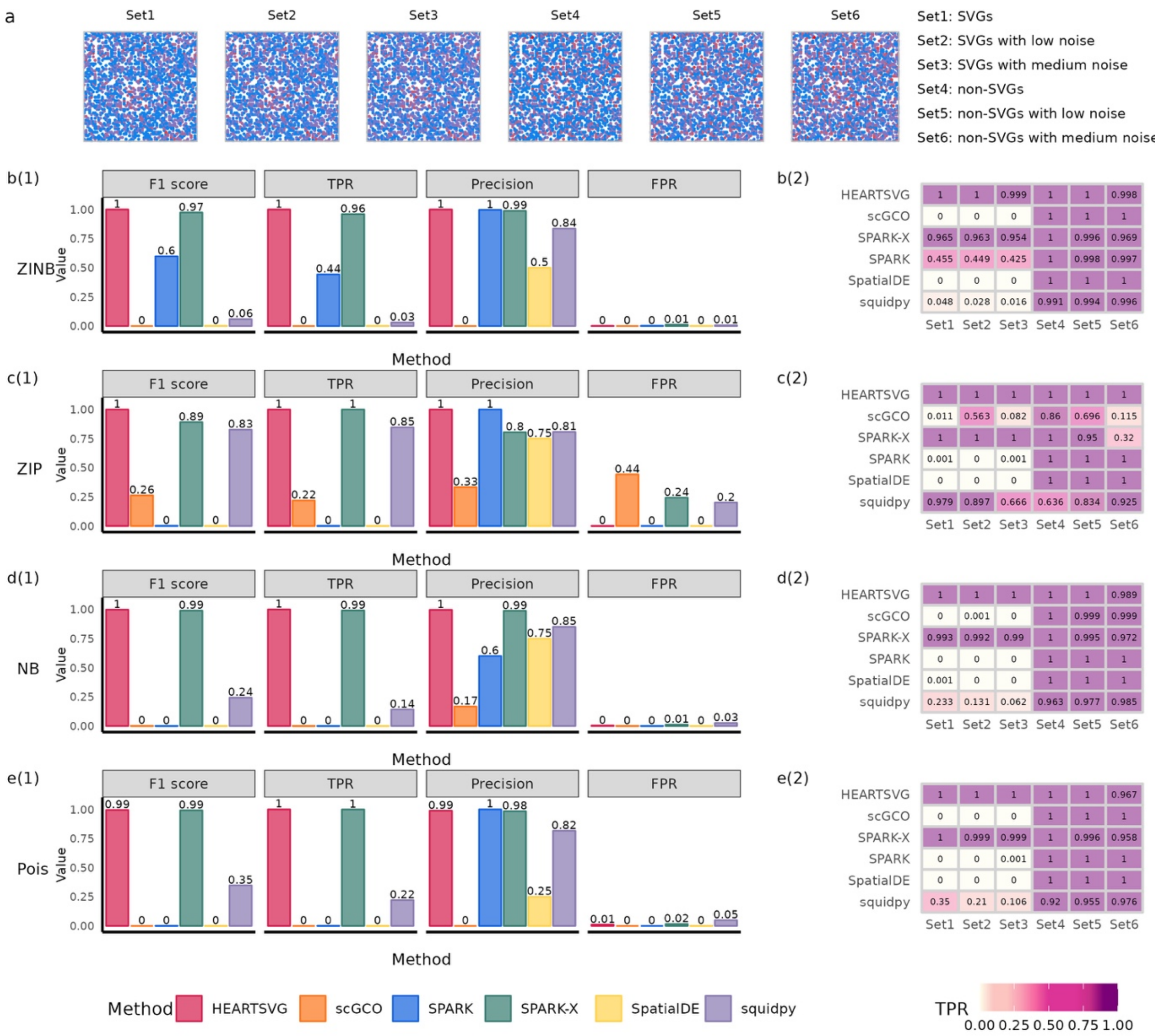


**Figure S49** Simulation results of SVGs identification using simulated data with mixture noise. **a,** Visualization of Pattern: Big squares with mixture noise. **b-e,** Simulation results of six different methods (HEARTSVG, scGCO, SPARK, SPARK-X, Squidpy) on simulated data generated by four distinct distributions (ZINB, ZIP, NB, Poisson). The bar diagram (sub-panel (1)) shows F1 scores, TPRs, precisions, and FPRs. The heatmap (sub-panel (2)) depicts the comparison of TPR values among six genesets. Source data are provided with this paper.

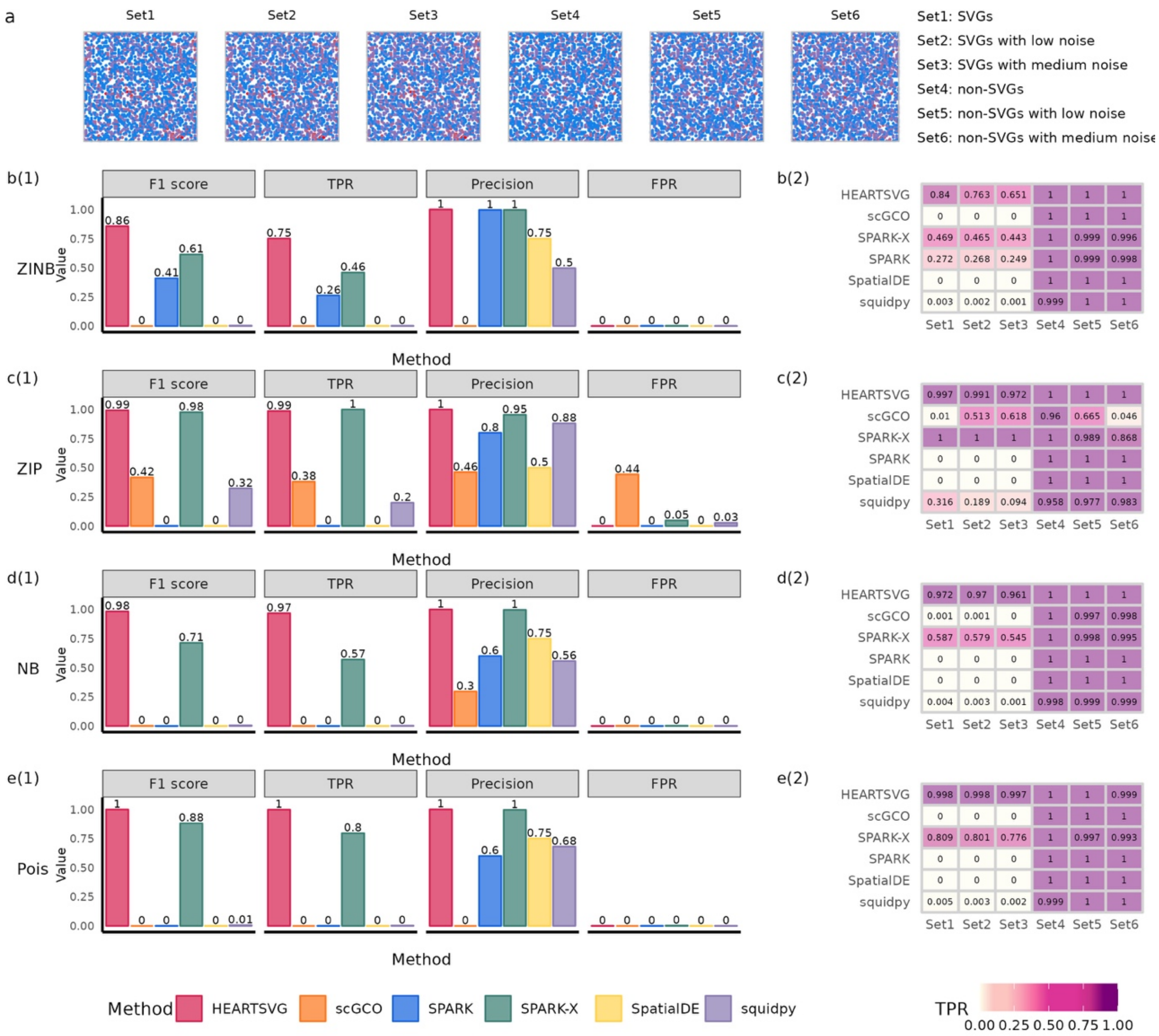


**Figure S50** Simulation results of SVGs identification using simulated data with mixture noise. **a,** Visualization of Pattern: Small triangles with mixture noise. **b-e,** Simulation results of six different methods (HEARTSVG, scGCO, SPARK, SPARK-X, Squidpy) on simulated data generated by four distinct distributions (ZINB, ZIP, NB, Poisson). The bar diagram (sub-panel (1)) shows F1 scores, TPRs, precisions, and FPRs. The heatmap (sub-panel (2)) depicts the comparison of TPR values among six genesets. Source data are provided with this paper.

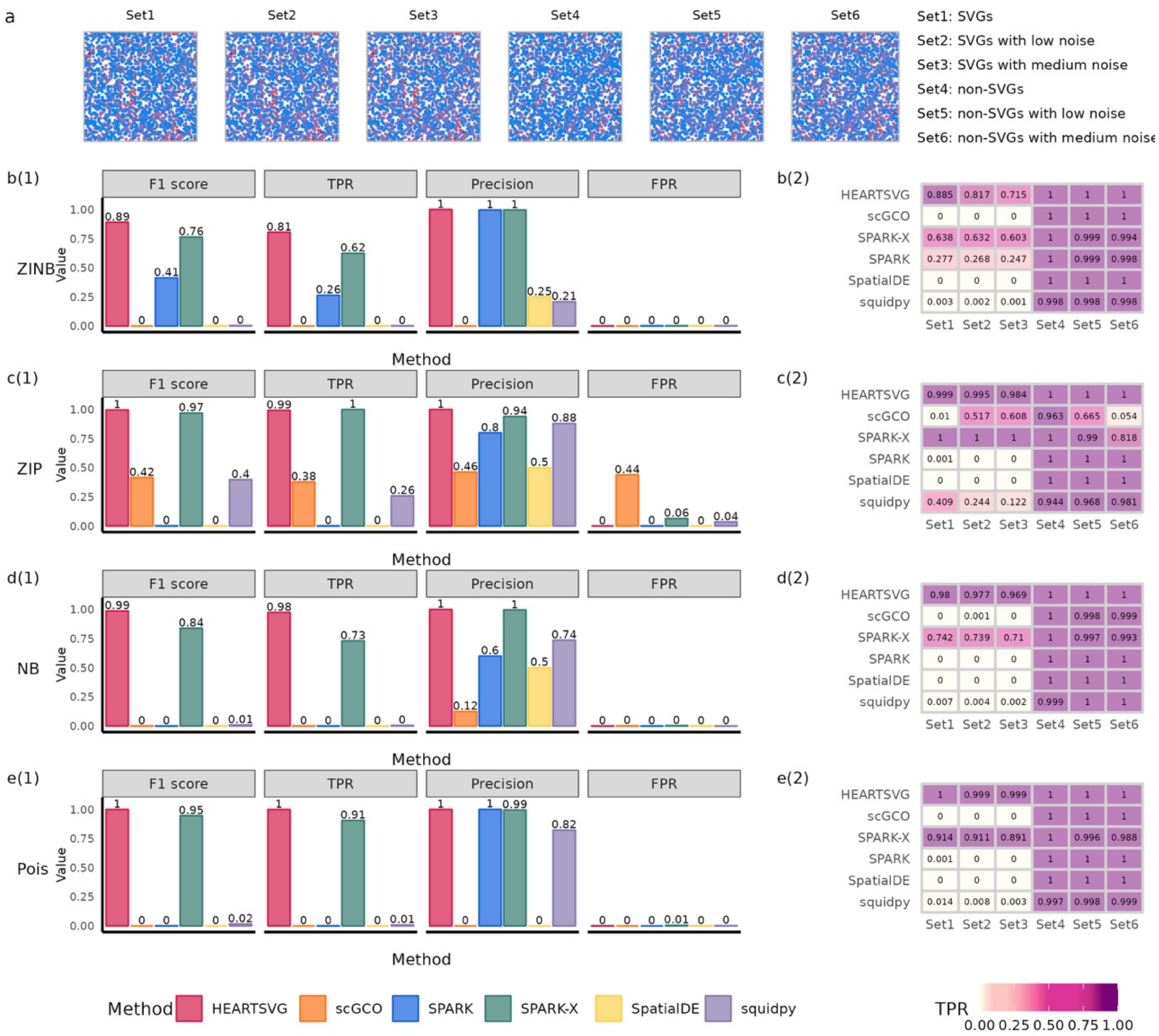


**Figure S51** Simulation results of SVGs identification using simulated data with mixture noise. **a,** Visualization of Pattern: Small circles with mixture noise. **b-e,** Simulation results of six different methods (HEARTSVG, scGCO, SPARK, SPARK-X, Squidpy) on simulated data generated by four distinct distributions (ZINB, ZIP, NB, Poisson). The bar diagram (sub-panel (1)) shows F1 scores, TPRs, precisions, and FPRs. The heatmap (sub-panel (2)) depicts the comparison of TPR values among six genesets. Source data are provided with this paper.

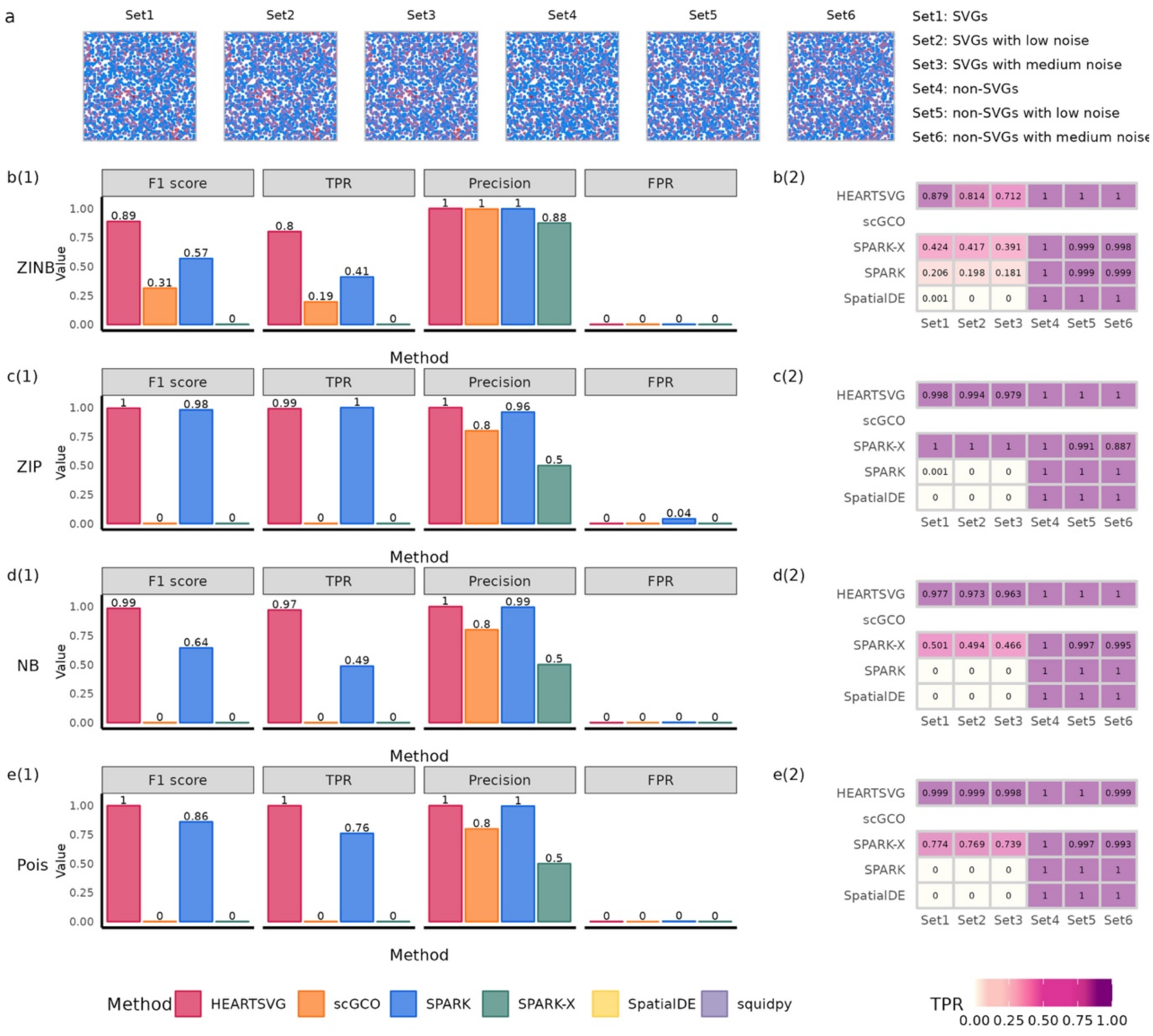


**Figure S52** Simulation results of SVGs identification using simulated data with mixture noise. **a,** Visualization of Pattern: Small squares with mixture noise. **b-e,** Simulation results of six different methods (HEARTSVG, scGCO, SPARK, SPARK-X, Squidpy) on simulated data generated by four distinct distributions (ZINB, ZIP, NB, Poisson). The bar diagram (sub-panel (1)) shows F1 scores, TPRs, precisions, and FPRs. The heatmap (sub-panel (2)) depicts the comparison of TPR values among six genesets. Source data are provided with this paper.

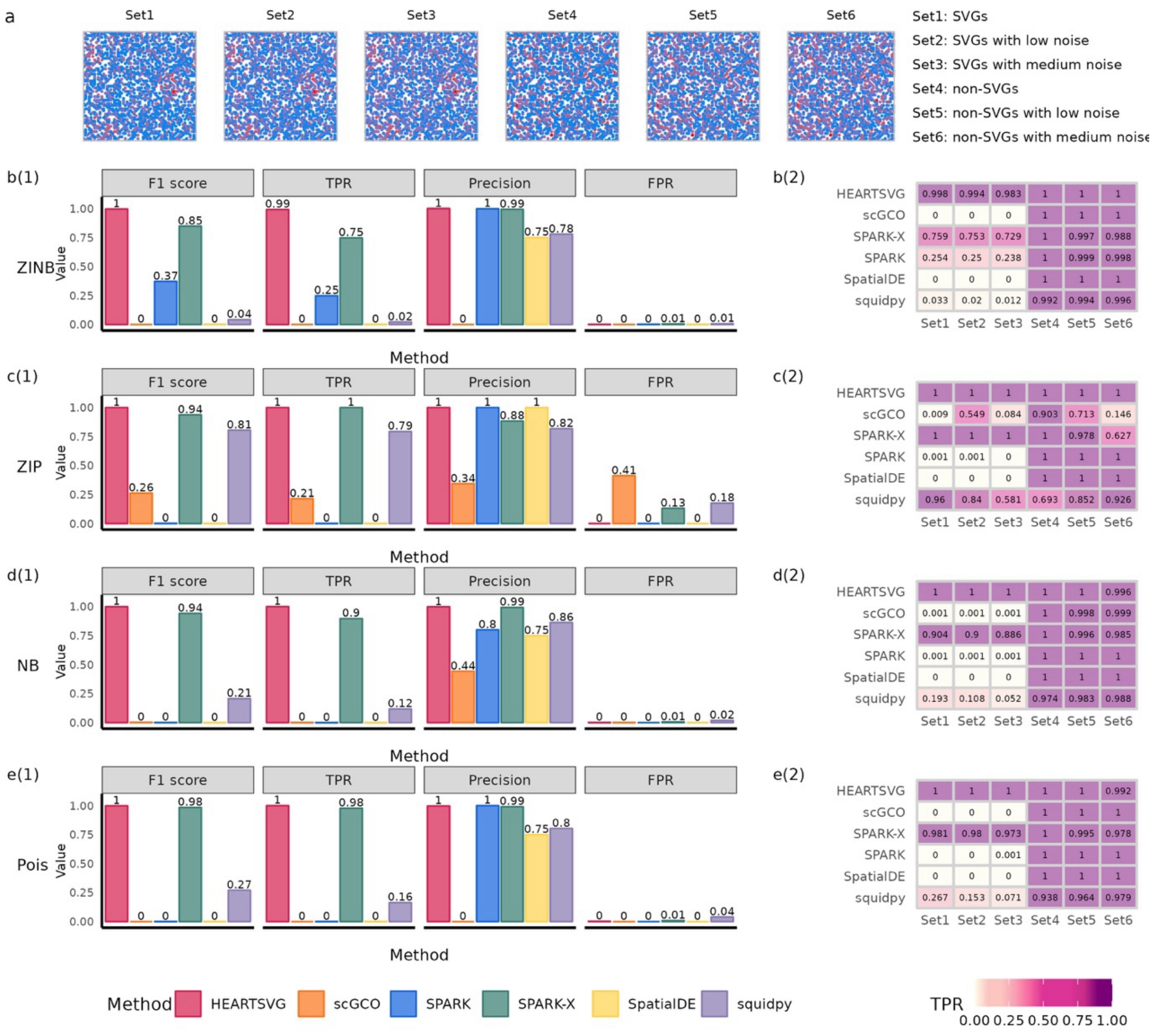


**Figure S53** Simulation results of SVGs identification using simulated data with mixture noise. **a,** Visualization of Pattern: Big circles II with mixture noise. **b-e,** Simulation results of six different methods (HEARTSVG, scGCO, SPARK, SPARK-X, Squidpy) on simulated data generated by four distinct distributions (ZINB, ZIP, NB, Poisson). The bar diagram (sub-panel (1)) shows F1 scores, TPRs, precisions, and FPRs. The heatmap (sub-panel (2)) depicts the comparison of TPR values among six genesets. Source data are provided with this paper.

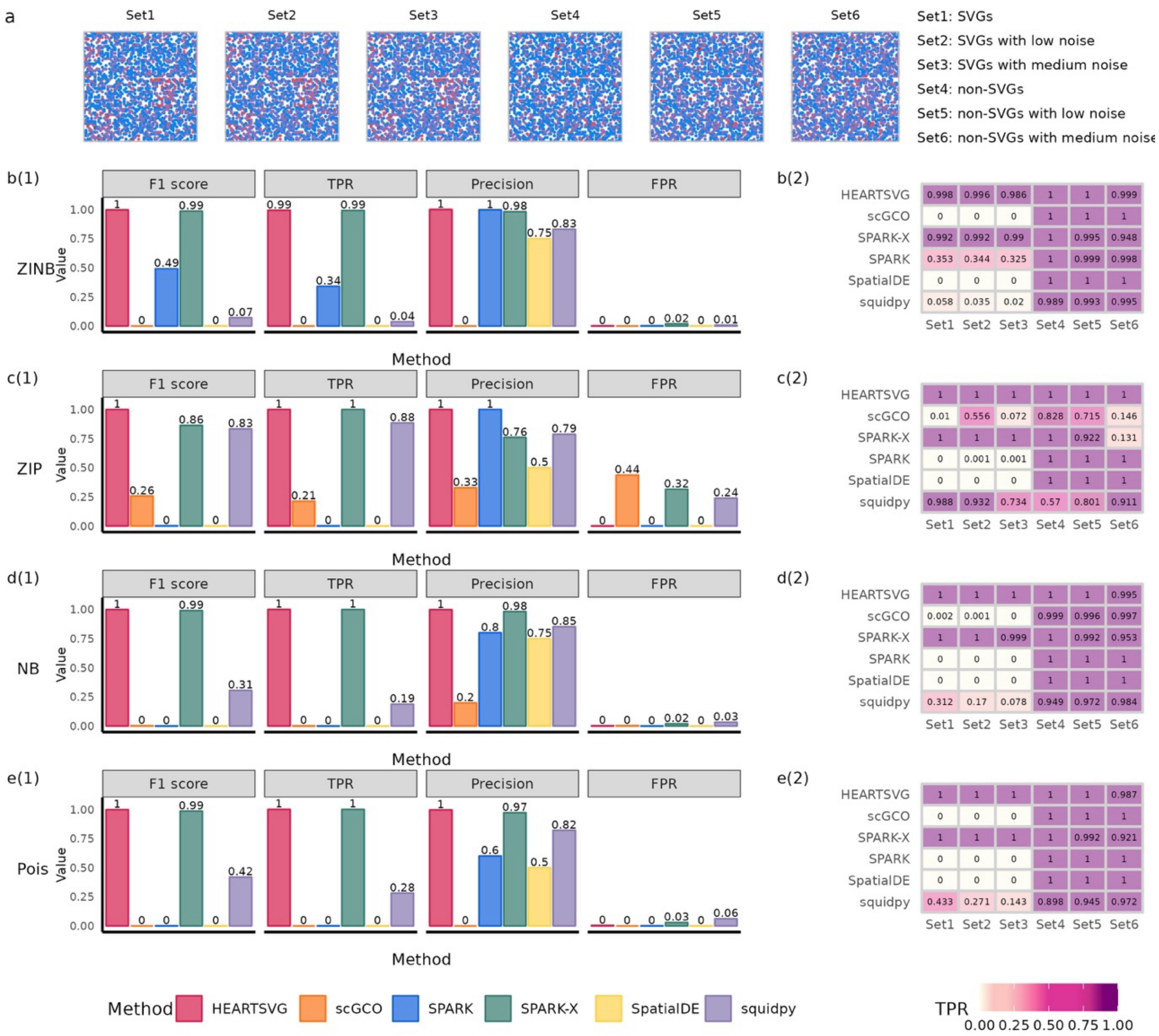


**Figure S54** Simulation results of SVGs identification using simulated data with mixture noise. **a,** Visualization of Pattern: Small triangles II with mixture noise. **b-e,** Simulation results of six different methods (HEARTSVG, scGCO, SPARK, SPARK-X, Squidpy) on simulated data generated by four distinct distributions (ZINB, ZIP, NB, Poisson). The bar diagram (sub-panel (1)) shows F1 scores, TPRs, precisions, and FPRs. The heatmap (sub-panel (2)) depicts the comparison of TPR values among six genesets. Source data are provided with this paper.

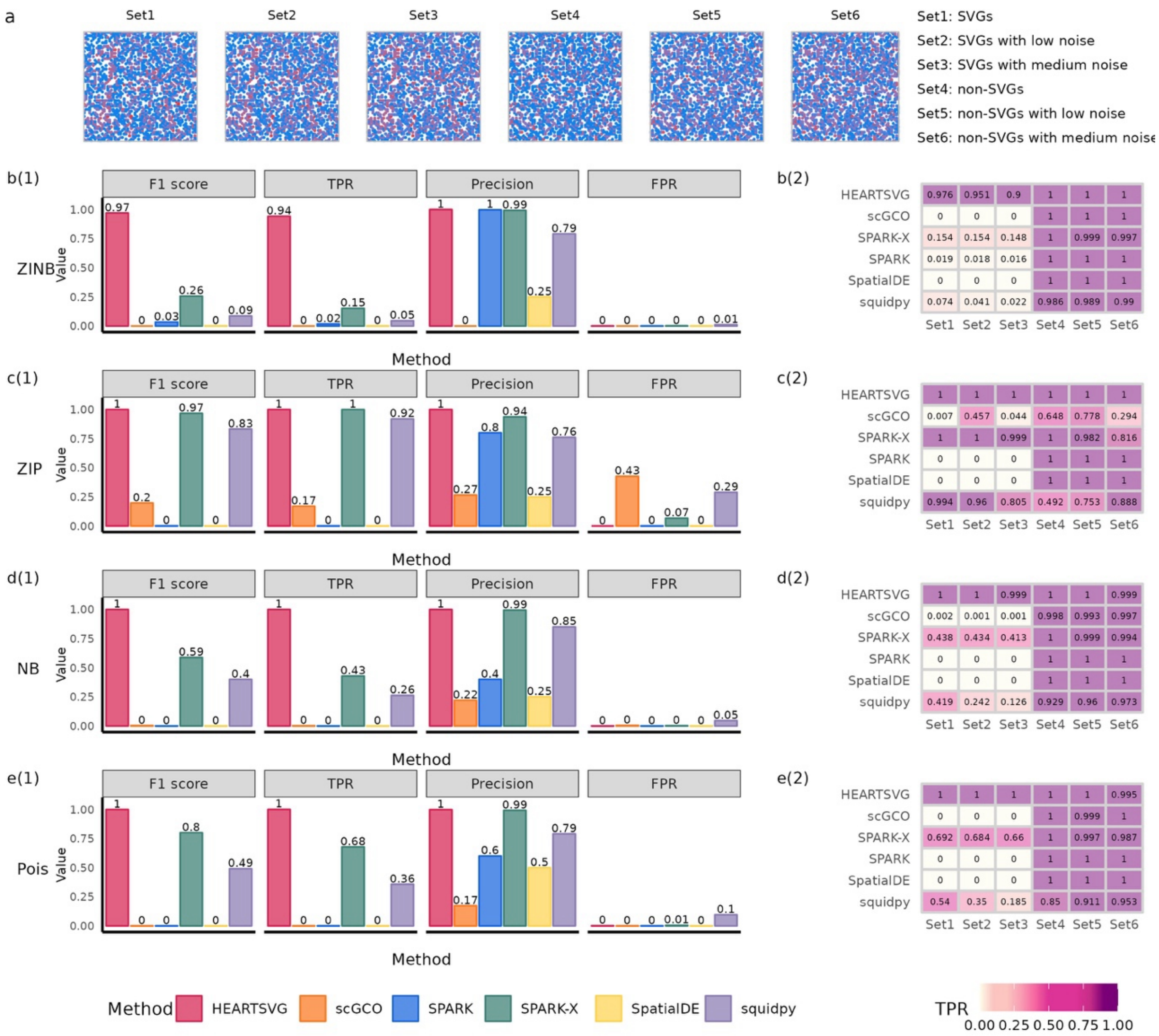

**Figure S55** Simulation results of SVGs identification using simulated data with mixture noise. **a,** Visualization of Pattern: Pattern I with mixture noise. **b-e,** Simulation results of six different methods (HEARTSVG, scGCO, SPARK, SPARK-X, Squidpy) on simulated data generated by four distinct distributions (ZINB, ZIP, NB, Poisson). The bar diagram (sub-panel (1)) shows F1 scores, TPRs, precisions, and FPRs. The heatmap (sub-panel (2)) depicts the comparison of TPR values among six genesets. Source data are provided with this paper.

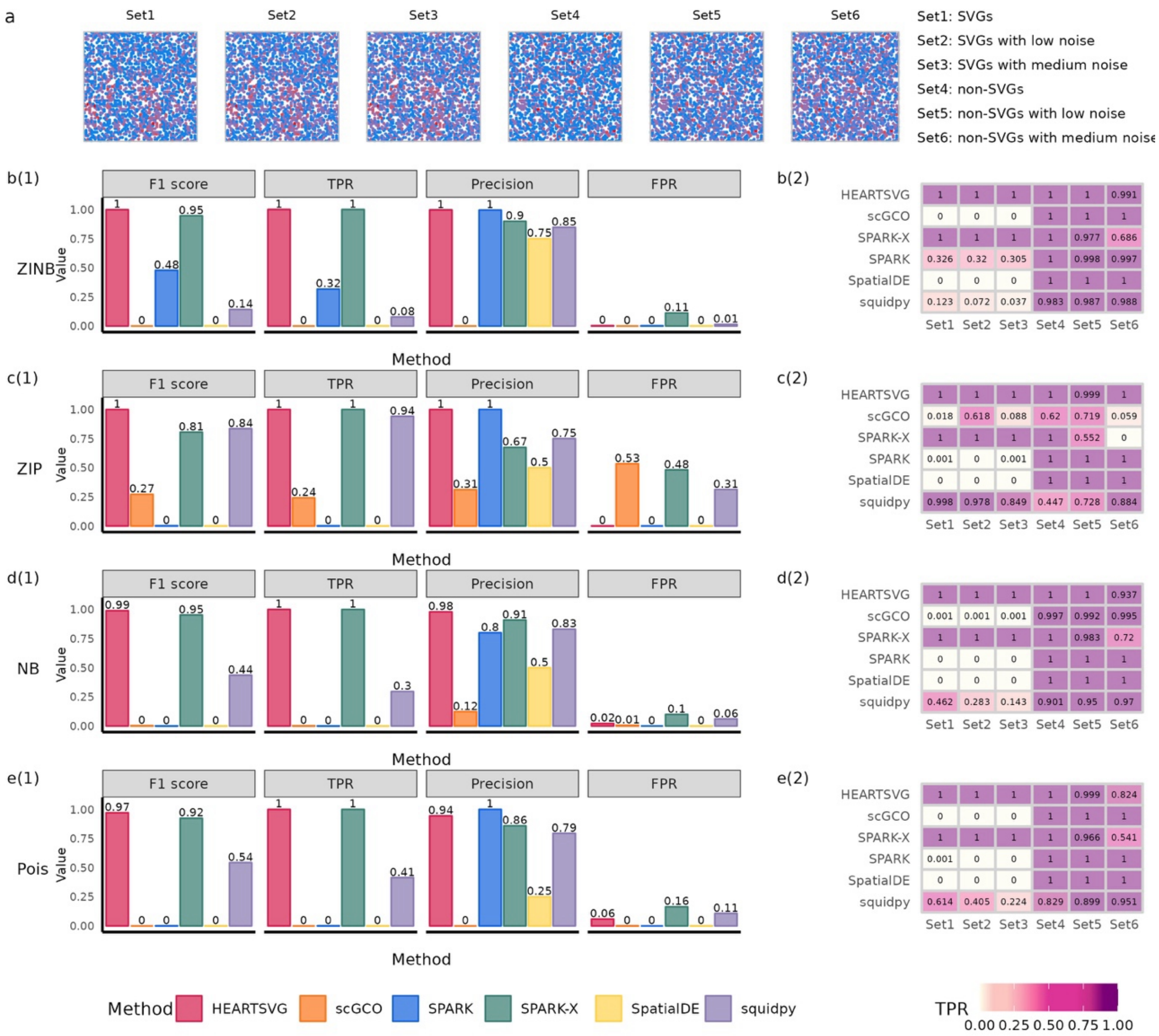


**Figure S56** Simulation results of SVGs identification using simulated data with mixture noise. **a,** Visualization of Pattern: Pattern II with mixture noise. **b-e,** Simulation results of six different methods (HEARTSVG, scGCO, SPARK, SPARK-X, Squidpy) on simulated data generated by four distinct distributions (ZINB, ZIP, NB, Poisson). The bar diagram (sub-panel (1)) shows F1 scores, TPRs, precisions, and FPRs. The heatmap (sub-panel (2)) depicts the comparison of TPR values among six genesets. Source data are provided with this paper.

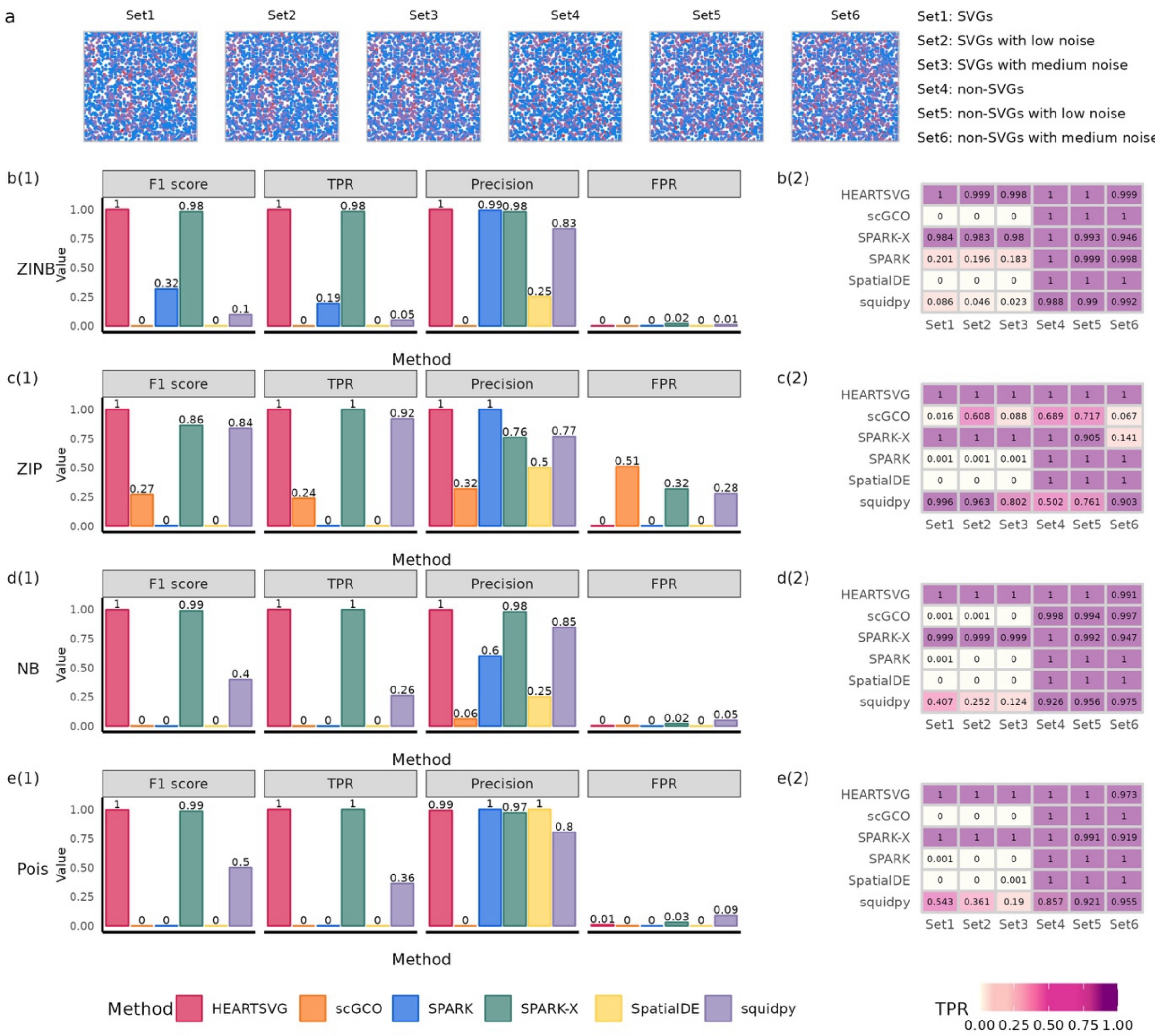


**Figure S57** Simulation results of SVGs identification using simulated data with mixture noise. **a,** Visualization of Pattern: Pattern III with mixture noise. **b-e,** Simulation results of six different methods (HEARTSVG, scGCO, SPARK, SPARK-X, Squidpy) on simulated data generated by four distinct distributions (ZINB, ZIP, NB, Poisson). The bar diagram (sub-panel (1)) shows F1 scores, TPRs, precisions, and FPRs. The heatmap (sub-panel (2)) depicts the comparison of TPR values among six genesets. Source data are provided with this paper.

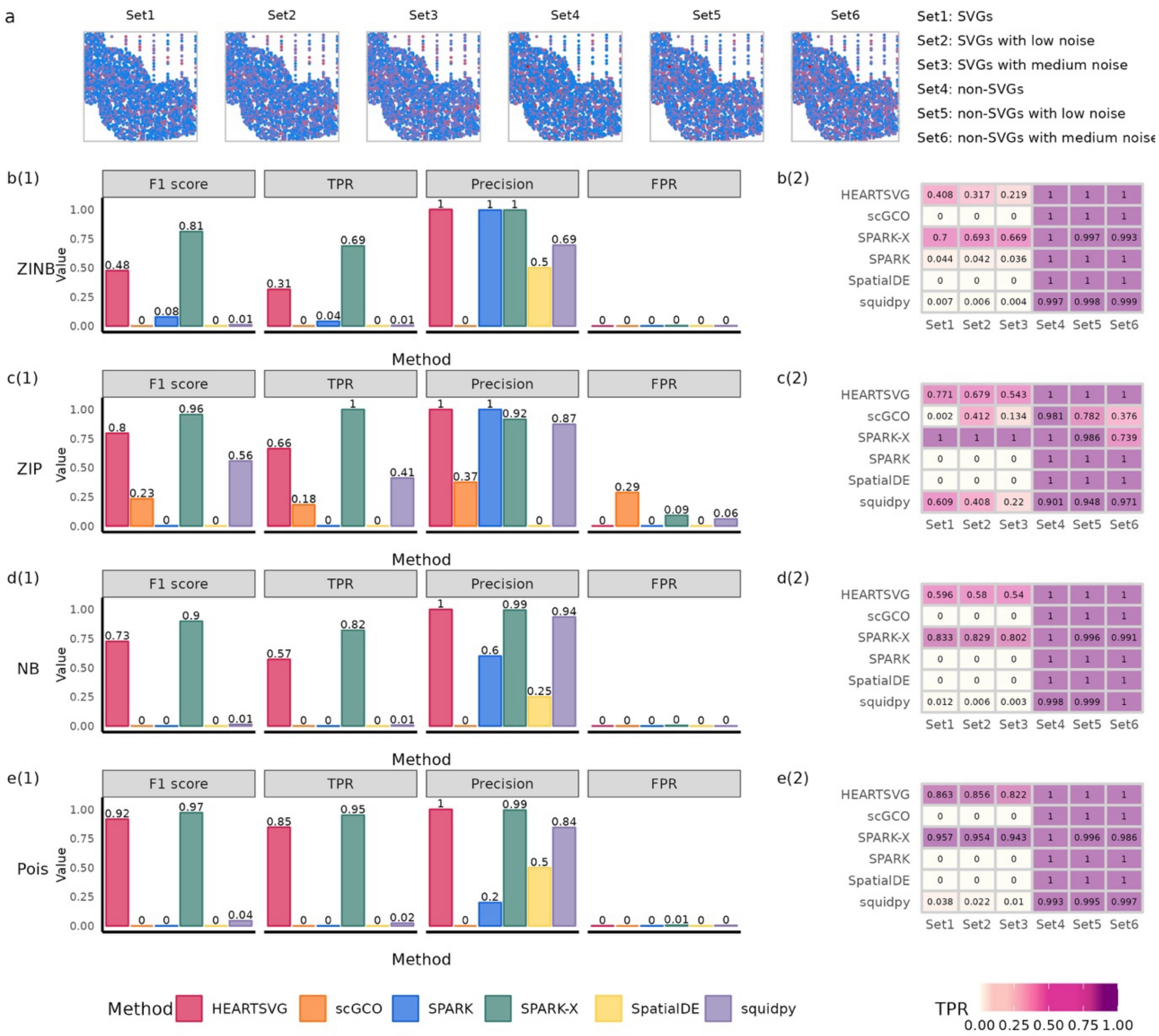


**Figure S58** Simulation results of SVGs identification using simulated data with mixture noise. **a,** Visualization of Pattern: Irreg pat I with mixture noise. **b-e,** Simulation results of six different methods (HEARTSVG, scGCO, SPARK, SPARK-X, Squidpy) on simulated data generated by four distinct distributions (ZINB, ZIP, NB, Poisson). The bar diagram (sub-panel (1)) shows F1 scores, TPRs, precisions, and FPRs. The heatmap (sub-panel (2)) depicts the comparison of TPR values among six genesets. Source data are provided with this paper.

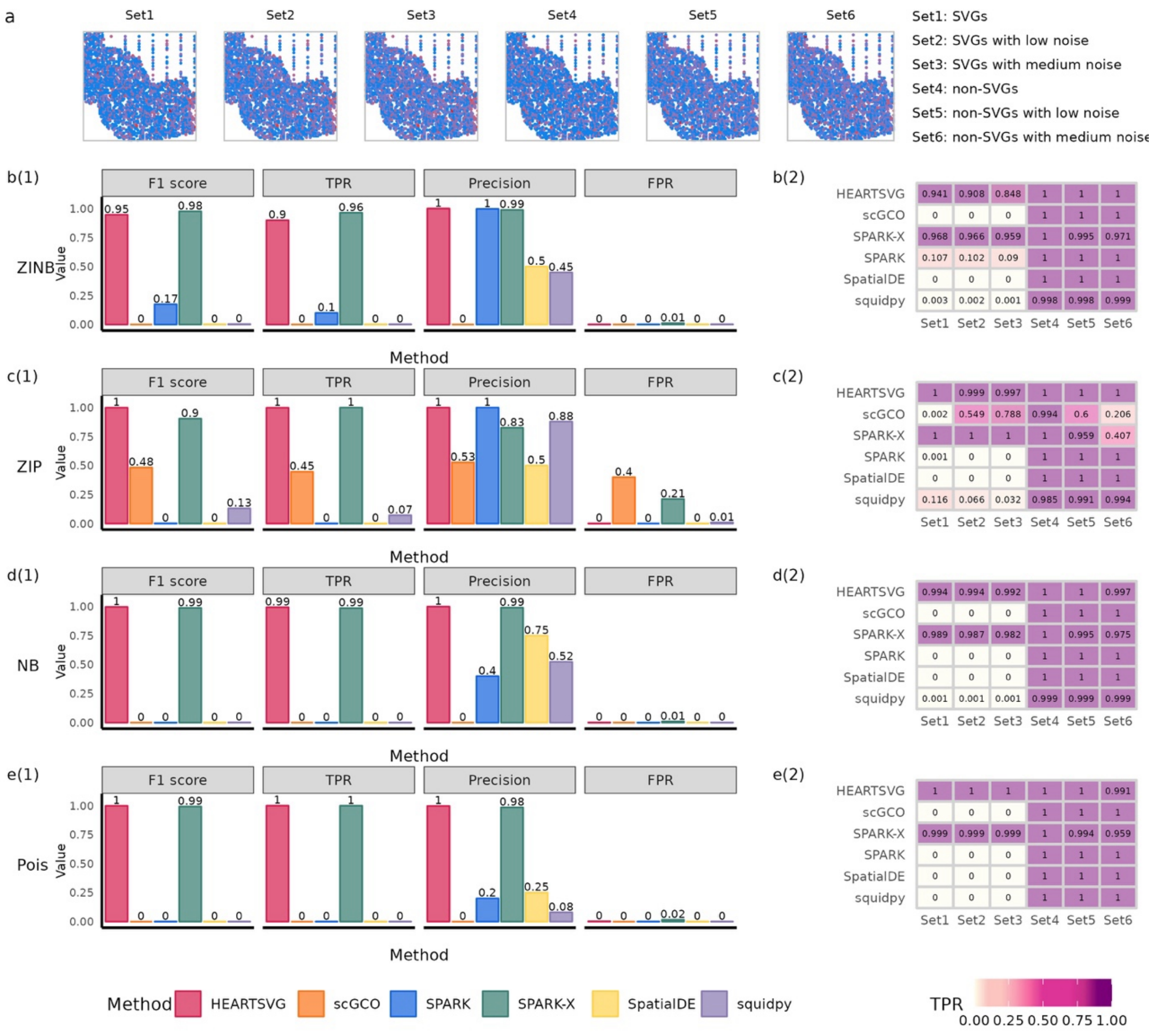


**Figure S59** Simulation results of SVGs identification using simulated data with mixture noise. **a,** Visualization of Pattern: Irreg pat II with mixture noise. **b-e,** Simulation results of six different methods (HEARTSVG, scGCO, SPARK, SPARK-X, Squidpy) on simulated data generated by four distinct distributions (ZINB, ZIP, NB, Poisson). The bar diagram (sub-panel (1)) shows F1 scores, TPRs, precisions, and FPRs. The heatmap (sub-panel (2)) depicts the comparison of TPR values among six genesets. Source data are provided with this paper.

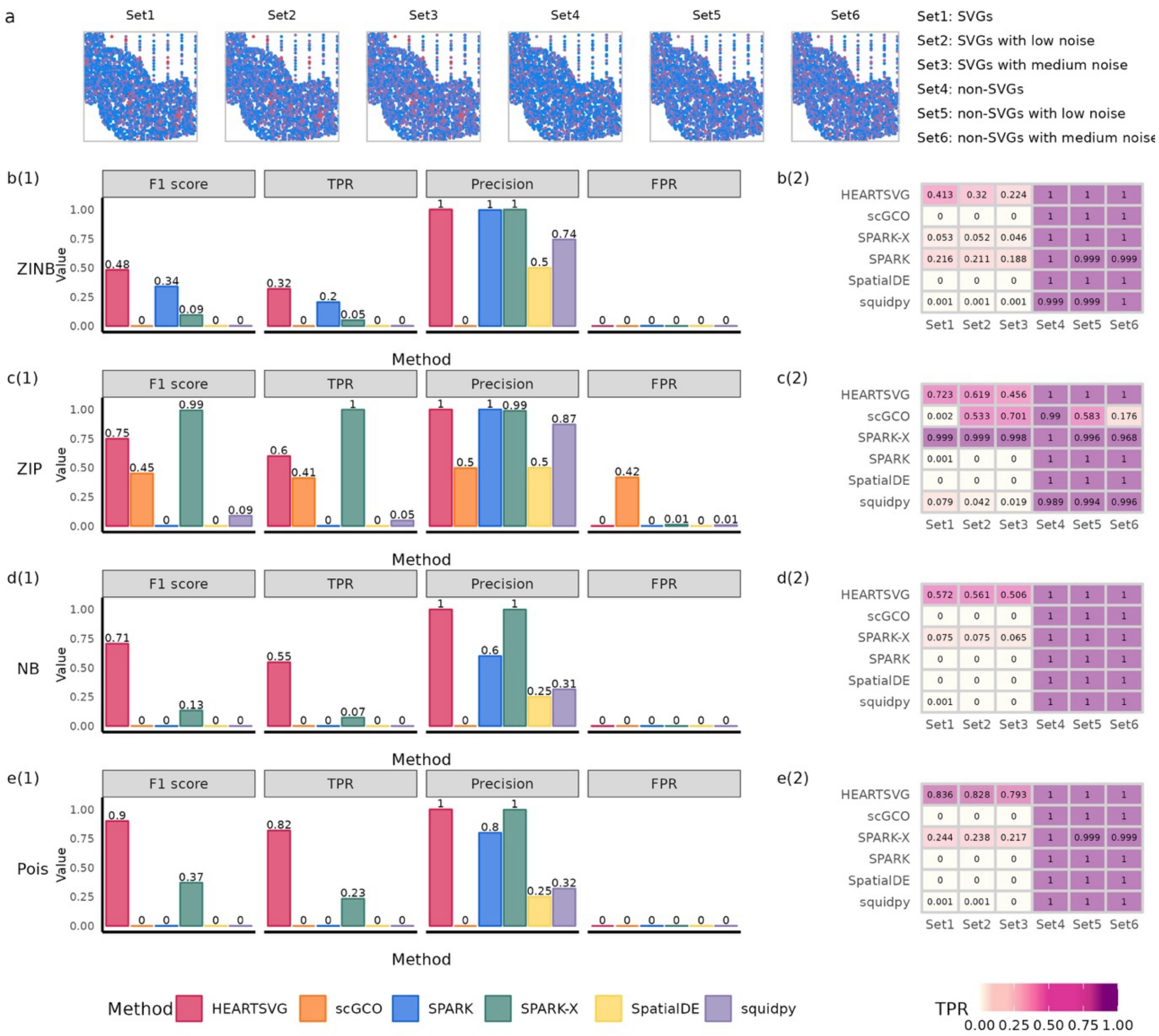


**Figure S60** Simulation results of SVGs identification using simulated data with mixture noise. **a,** Visualization of Pattern: Irreg pat III with mixture noise. **b-e,** Simulation results of six different methods (HEARTSVG, scGCO, SPARK, SPARK-X, Squidpy) on simulated data generated by four distinct distributions (ZINB, ZIP, NB, Poisson). The bar diagram (sub-panel (1)) shows F1 scores, TPRs, precisions, and FPRs. The heatmap (sub-panel (2)) depicts the comparison of TPR values among six genesets. Source data are provided with this paper.

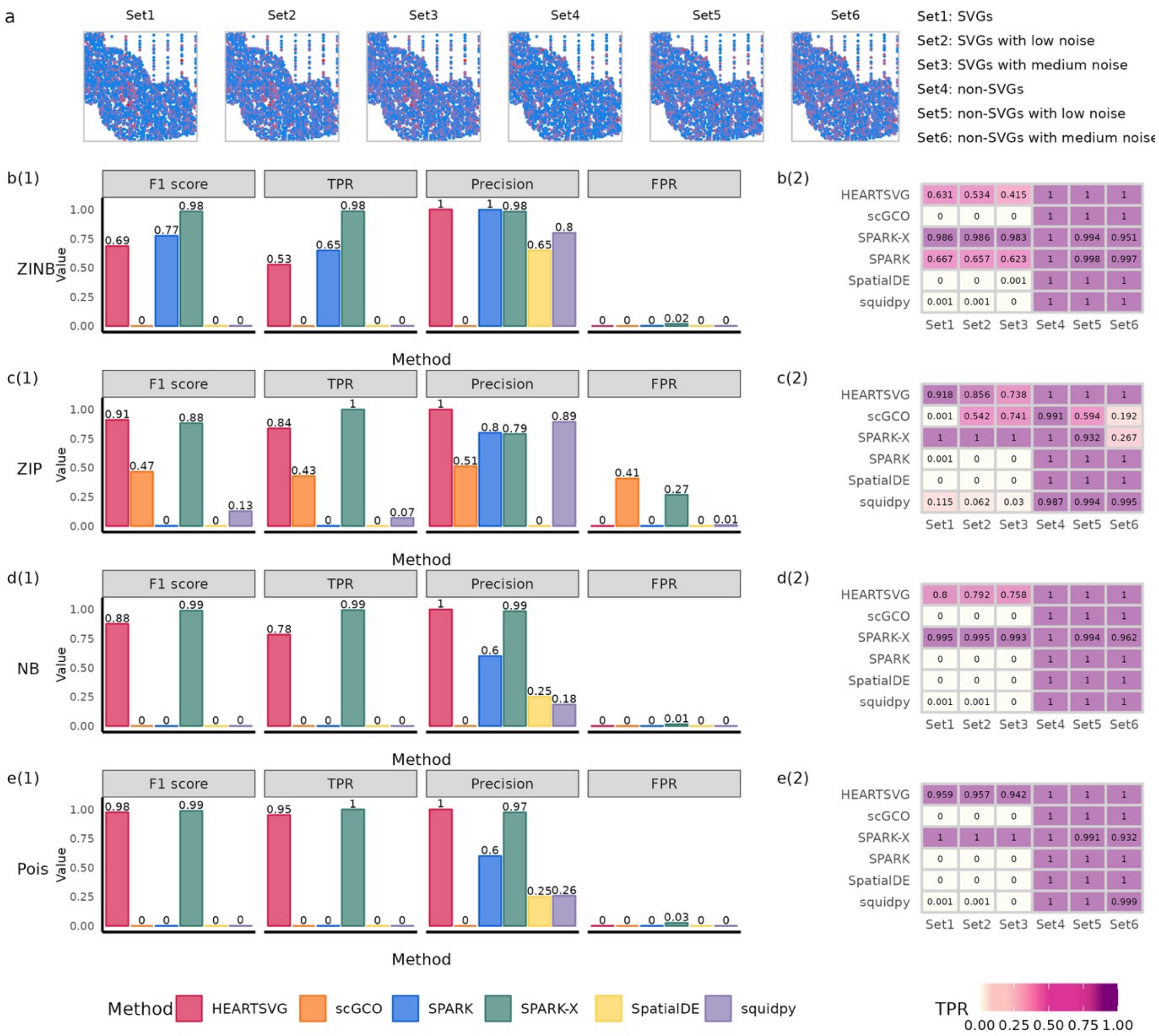


**Figure S61** Simulation results of SVGs identification using simulated data with mixture noise. **a,** Visualization of Pattern: Irreg pat IV with mixture noise. **b-e,** Simulation results of six different methods (HEARTSVG, scGCO, SPARK, SPARK-X, Squidpy) on simulated data generated by four distinct distributions (ZINB, ZIP, NB, Poisson). The bar diagram (sub-panel (1)) shows F1 scores, TPRs, precisions, and FPRs. The heatmap (sub-panel (2)) depicts the comparison of TPR values among six genesets. Source data are provided with this paper.

## 10.2 Results of noise-free simulations

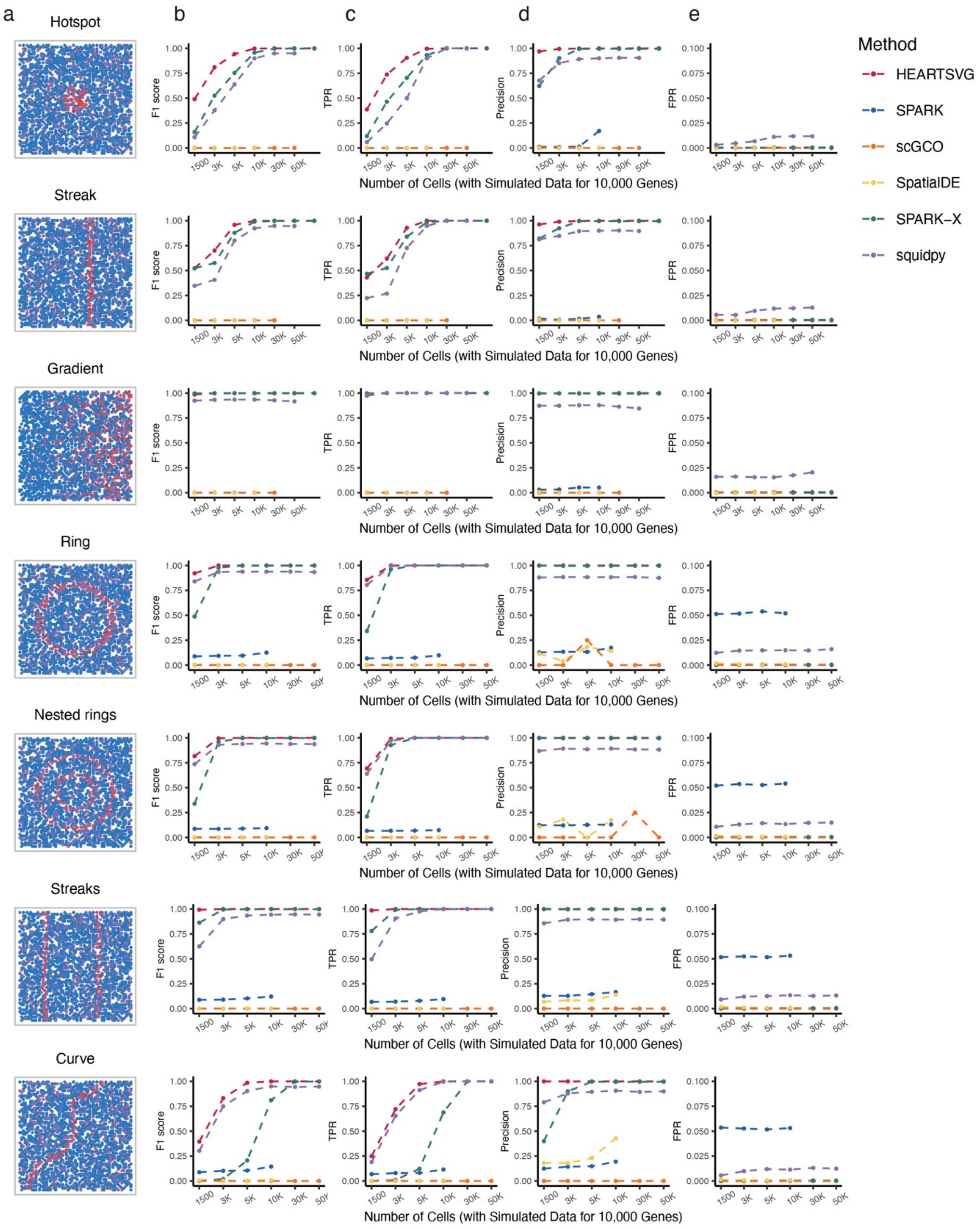

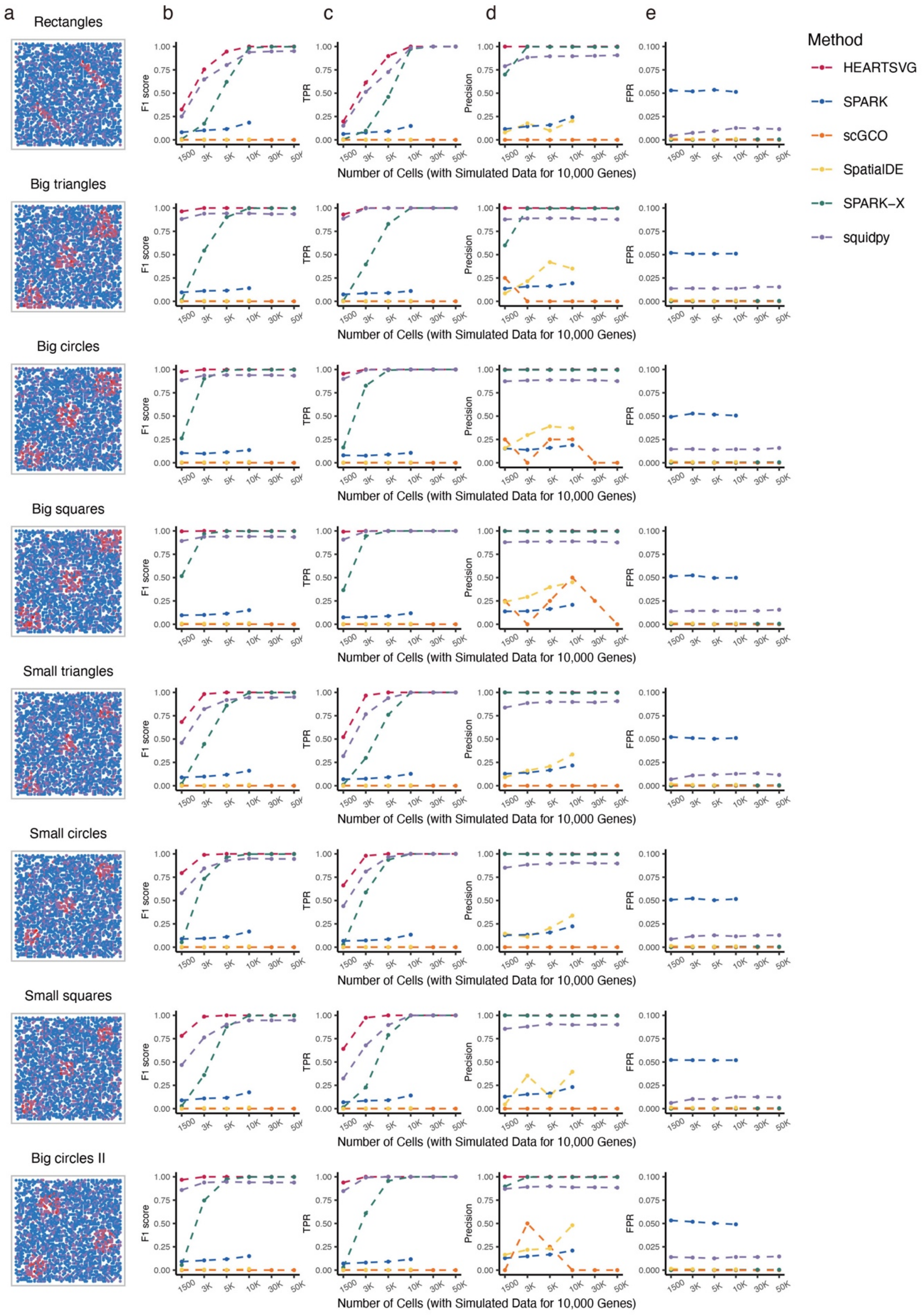

a
b
c
d
e
Rectangles
Big triangles
Big circles
Big squares
Small triangles
Small circles
Small squares
Big circles II
F1 score
TPR
Precision
FPR
Number of Cells (with Simulated Data for 10,000 Genes)
1500
3K
5K
10K
30K
50K
Method
HEARTSVG
SPARK
scGCO
SpatialDE
SPARK−X
squidpy

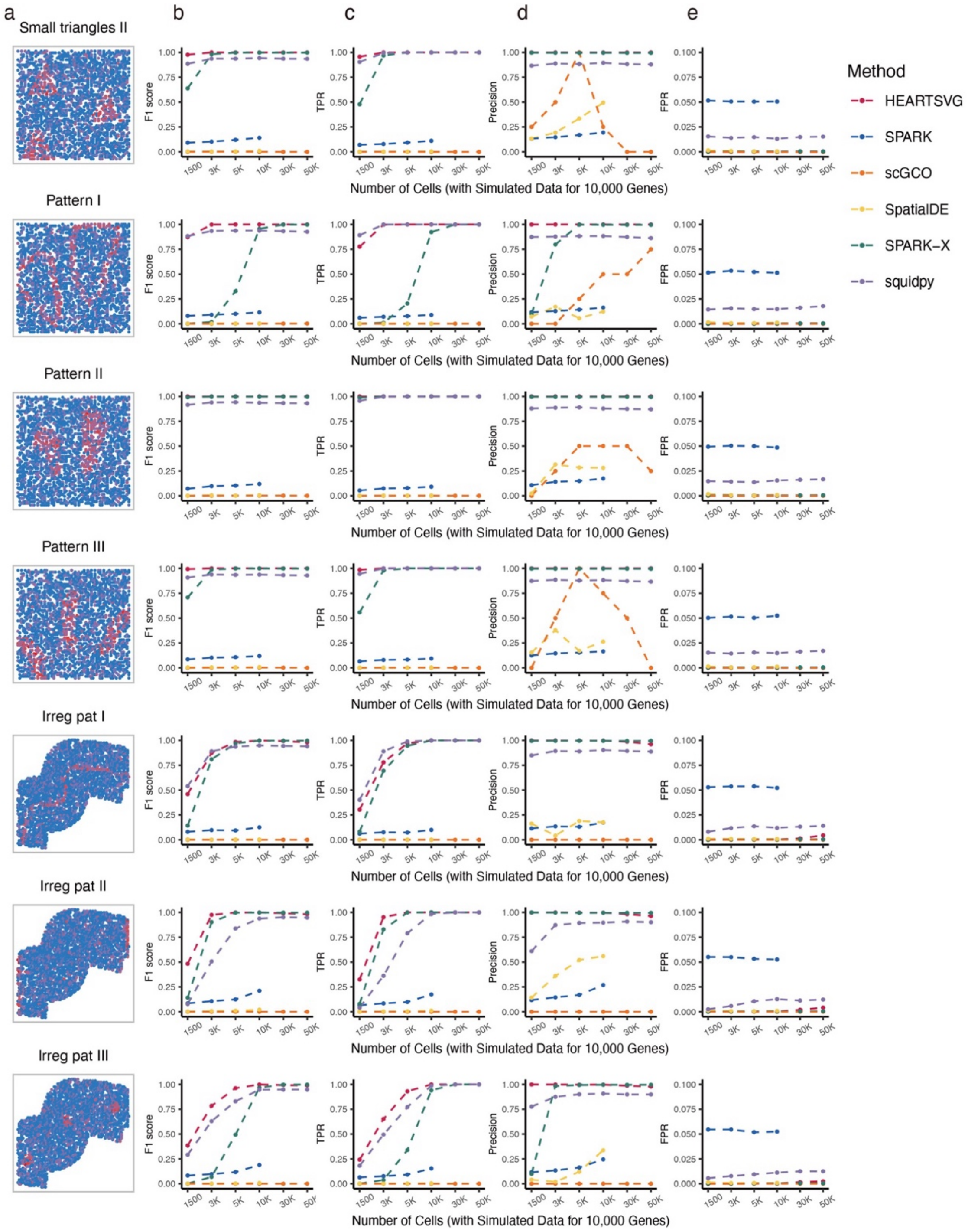


**Figure S62 a,** Visualization of 22 representative spatial expression patterns: Hotspot, Streak, Gradient, Ring, Nested rings, Streaks, Curve, Rectangles, Big triangles, Big circles, Big squares, Small triangles, Small circles, Small squares, Big circles II, Small triangles II, Pattern I, Pattern II, Pattern III, Irreg pat I, Irreg pat II, Irreg pat III. b-e, F1 score plots, TPR plots, Precision plots, and FPR plots compare the index values (y-axis) of HEARTSVG (red), scGCO (orange), SpatialDE (yellow), SPARK (blue), SPARK-X (green), and Squidpy (purple) in simulation data. The comparison is based on varying sample sizes (x-axis) at an adjusted p-value cutoff of 0.05. Each plot corresponds to the left spatial patterns in sub-figure **a**. Source data are provided with this paper.

### 10.3 Results of simulations with Gaussian noise

Gaussian noise is commonly used in the field of computer vision. Consequently, we referred to articles in the field of computer vision and modified the approach for adding Gaussian noise. To ensure a consistent impact across all images, Min-Max normalization is performed before noise addition. We simulated 3000 cells with 10,000 genes (1000 SVGs and 9000 non-SVGs) in each scenario. We added varying levels (ranging from 0 to 0.4) of noises to noise-free data to create simulated datasets with different degrees of noise. The parameters of the four distributions we used were shown in Tables S1.

New adding Gaussian noise:

1. **Min-Max normalization:**
   We first normalized the expression dat**a,** scaling gene expression values to a uniform range between 0 and 1. This helps maintain consistency in expression levels across different distributions.
2. **Generating and adding noise:**
   We generated noise from a Gaussian distribution $N(0, \sigma)$ and added it to the normalized dat**a,** ensuring that the post-noise addition pixel values remained within the valid range.
3. **Reverse normalization:**
   After noise addition, we reverted the expression values from their normalized state back to their original scale.
4. **We applied methods to identify SVGs.**

Following this modified Gaussian noise addition approach, we found that adding the same level of Gaussian noise consistently impacted different data features. The variation in $F_1$ $scores$ for all methods was also consistent across the different distributions of simulated data with Gaussian noise (Fig. **S63-S65**).

**ZIP**: Patterns became blurred when noise exceeded 0.3, and three methods' $F_1$ $scores$ decreased from 0.3 noise.

**Pois**: Patterns became blurred when noise exceeded 0.2, leading to a noticeable decline in performance across methods.

**NB**: Similar to Poisson, patterns blurred when noise exceeded 0.1, three methods' $F_1$ $score$ declined significantly.

**ZINB**: Given its inherent sparsity and dispersion, initial patterns were already somewhat blurred. Adding 0.05 noise resulted in blurred spatial pattern, and three methods' $F_1$ $score$ declined significantly from 0.05 noise onward.

Similar to our observations in the sensitivity analysis, we found that the performance of scGCO declines with increased Gaussian noise, leading to unreliable outcomes and fluctuations. We hypothesize that this is due to the fixed hyperparameter, the initial factor, but the influence of the initial factor on the direction of result variations remains unclear.

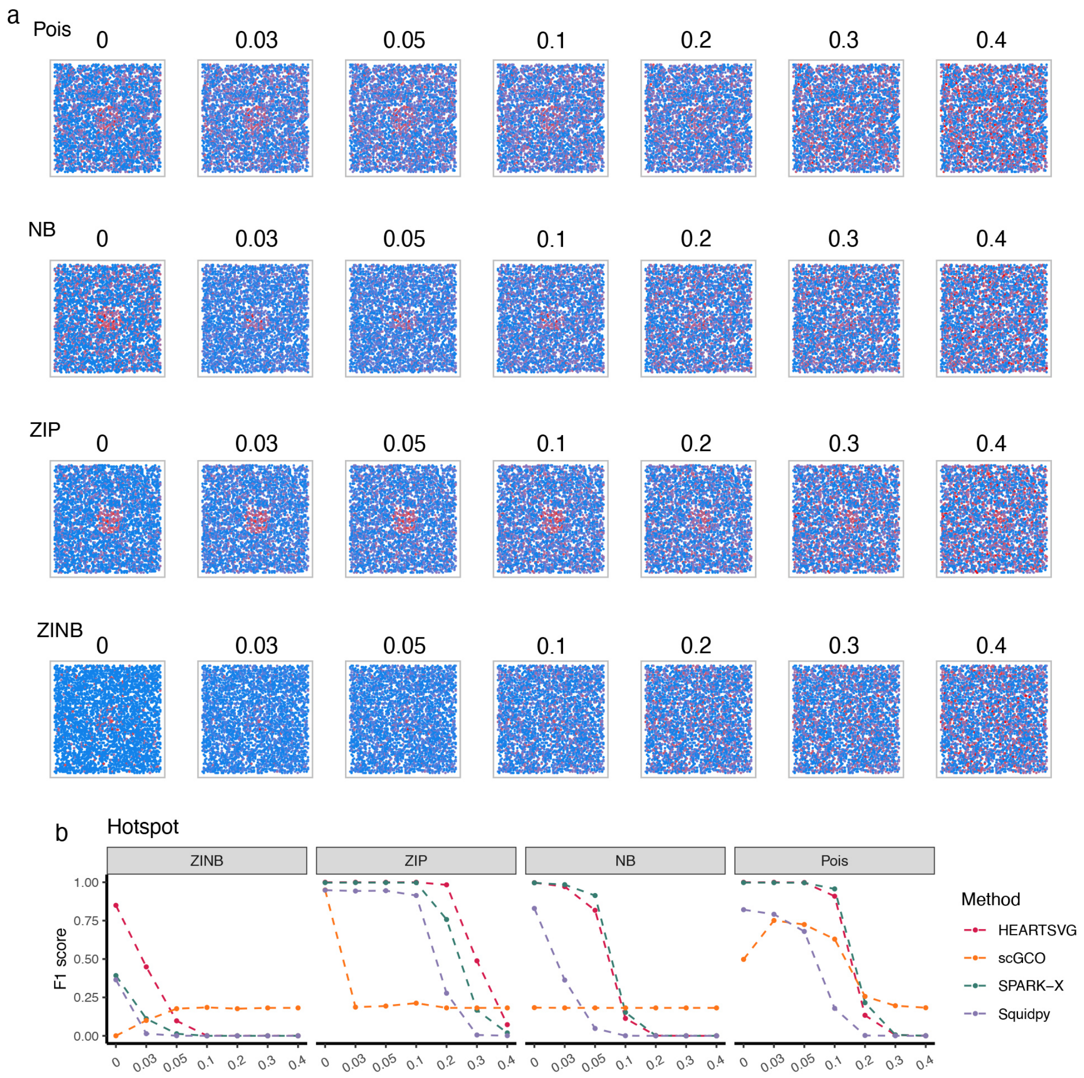


**Figure S63** Hotspot Pattern. **a,** Visualizations of simulated data from various distributions, incorporating noise using the modified Gaussian noise addition approach. **b,** Comparison of $F_1$ $scores$ on the new Gaussian noise simulated data. Source data are provided with this paper.

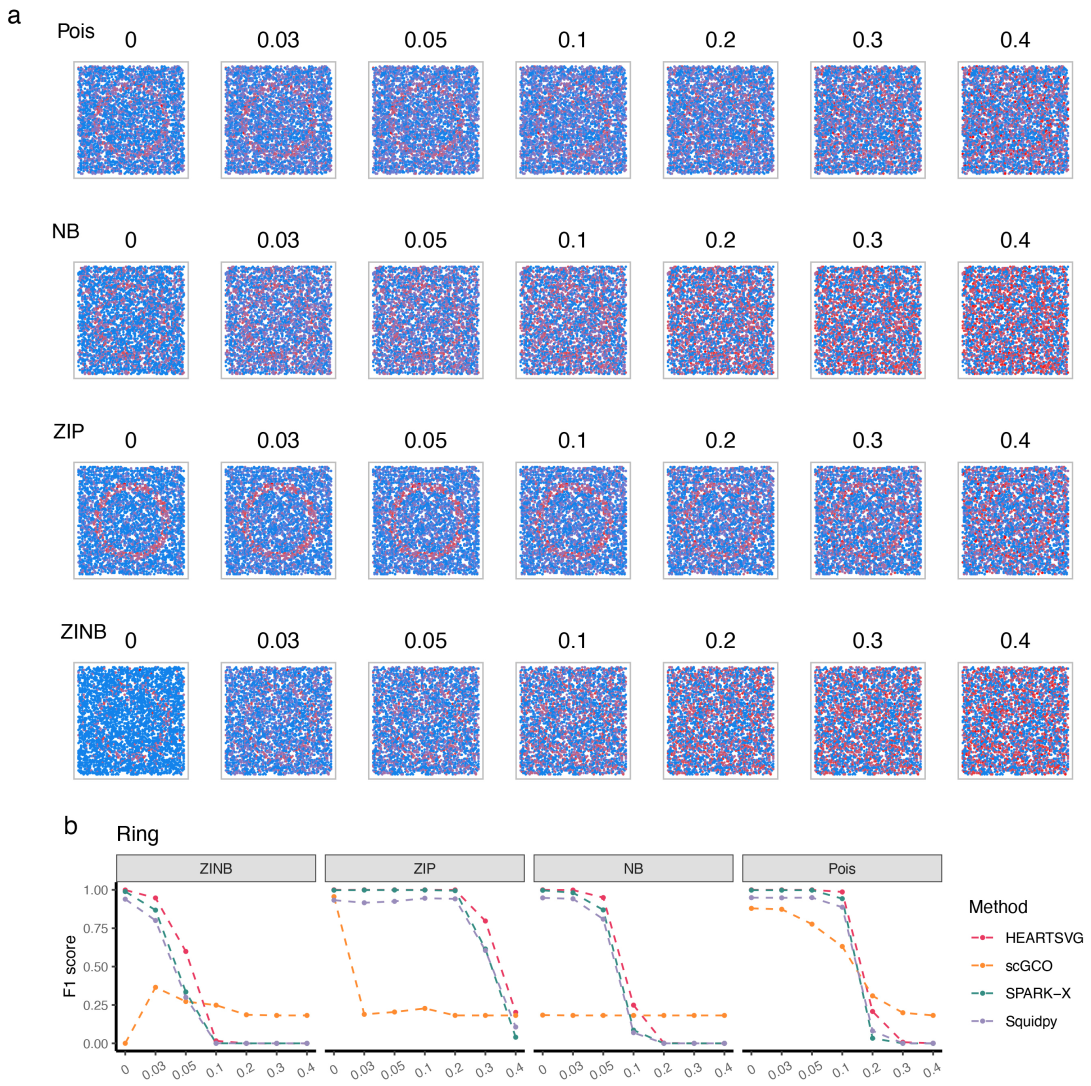


**Figure S64** Ring Pattern. **a,** Visualizations of simulated data from various distributions, incorporating noise using the modified Gaussian noise addition approach. **b,** Comparison of $F_1\ scores$ on the new Gaussian noise simulated data. Source data are provided with this paper.

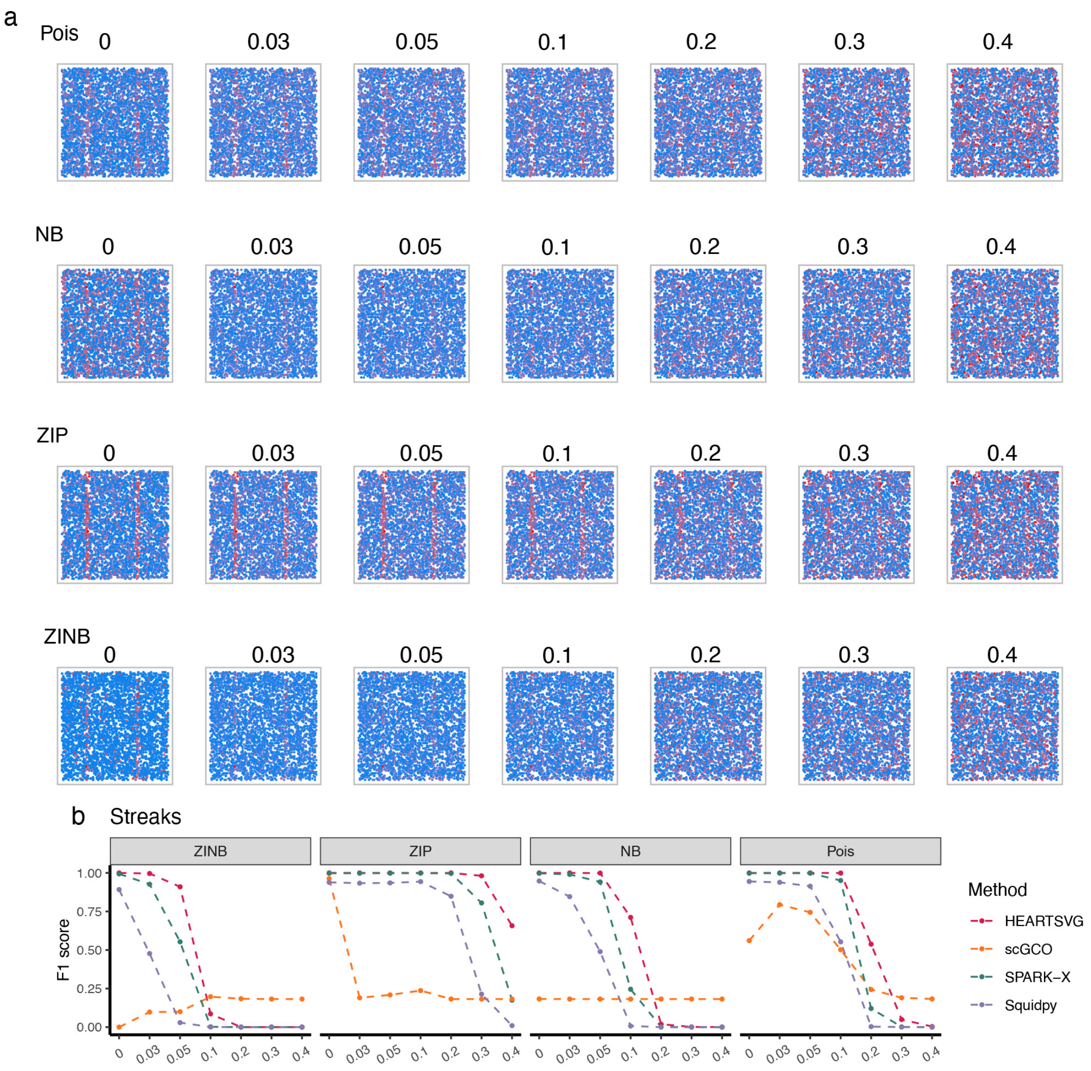


**Figure S65** Streaks Pattern. **a,** Visualizations of simulated data from various distributions, incorporating noise using the modified Gaussian noise addition approach. **b,** Comparison of $F_1$ *scores* on the new Gaussian noise simulated data. Source data are provided with this paper.

## 10.4 Results of simulations with noise of 'Randomly Exchanging Expression Values of Selected Nodes'

Due to the different ways of generating noise,we created new simulation data (both expression and coordinates) with noise, instead of transforming the noise-free data. We used the same parameter settings as in Section 1-3 to generate the spatial patterns and simulated 3000 cells with 10,000 genes (1000 SVGs and 9000 non-SVGs) in each scenario. The parameters of the four distributions we used are shown in Tables S1 and S5.

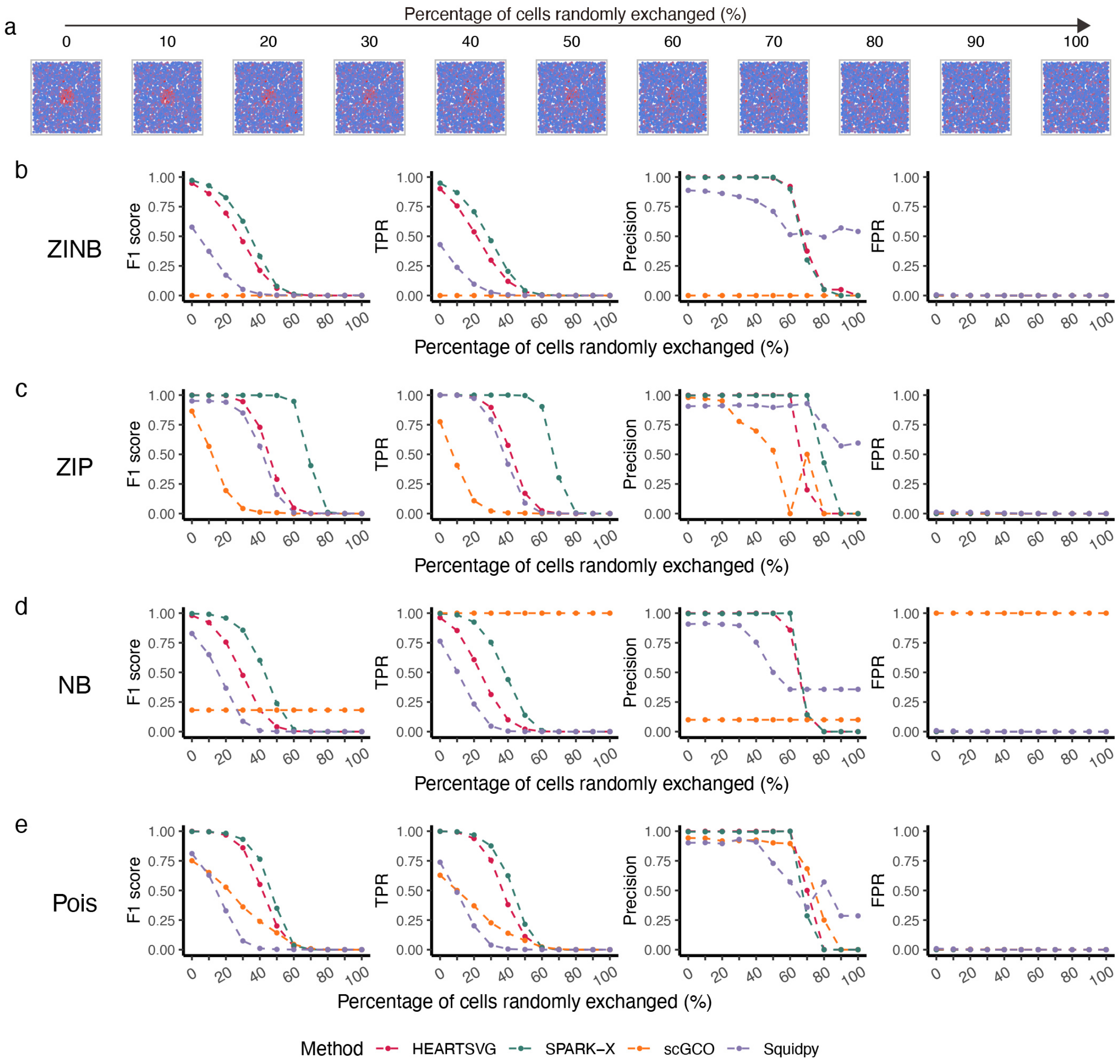


**Figure S66** Simulation results for identifying SVGs using simulated data with noise of 'Randomly Exchanging Expression Values of Selected Nodes.' **a,** Visualization of Pattern: Hotspot with different percentage of cells random exchanges (%). **b-e,** Simulation results of four different methods (HEARTSVG, scGCO, SPARK-X and Squidpy) on simulated data generated by four distinct distributions (ZINB, ZIP, NB, Poisson). F1 score plots, TPR plots,

Precision plots, and FPR plots compare the index values (y-axis) of HEARTSVG (red), scGCO (orange), SPARK-X (green), and Squidpy (purple) across different percentage of cells random exchanges (x-axis). Index values were calculated at the adjusted p-value cutoff of 0.05. Source data are provided with this paper.

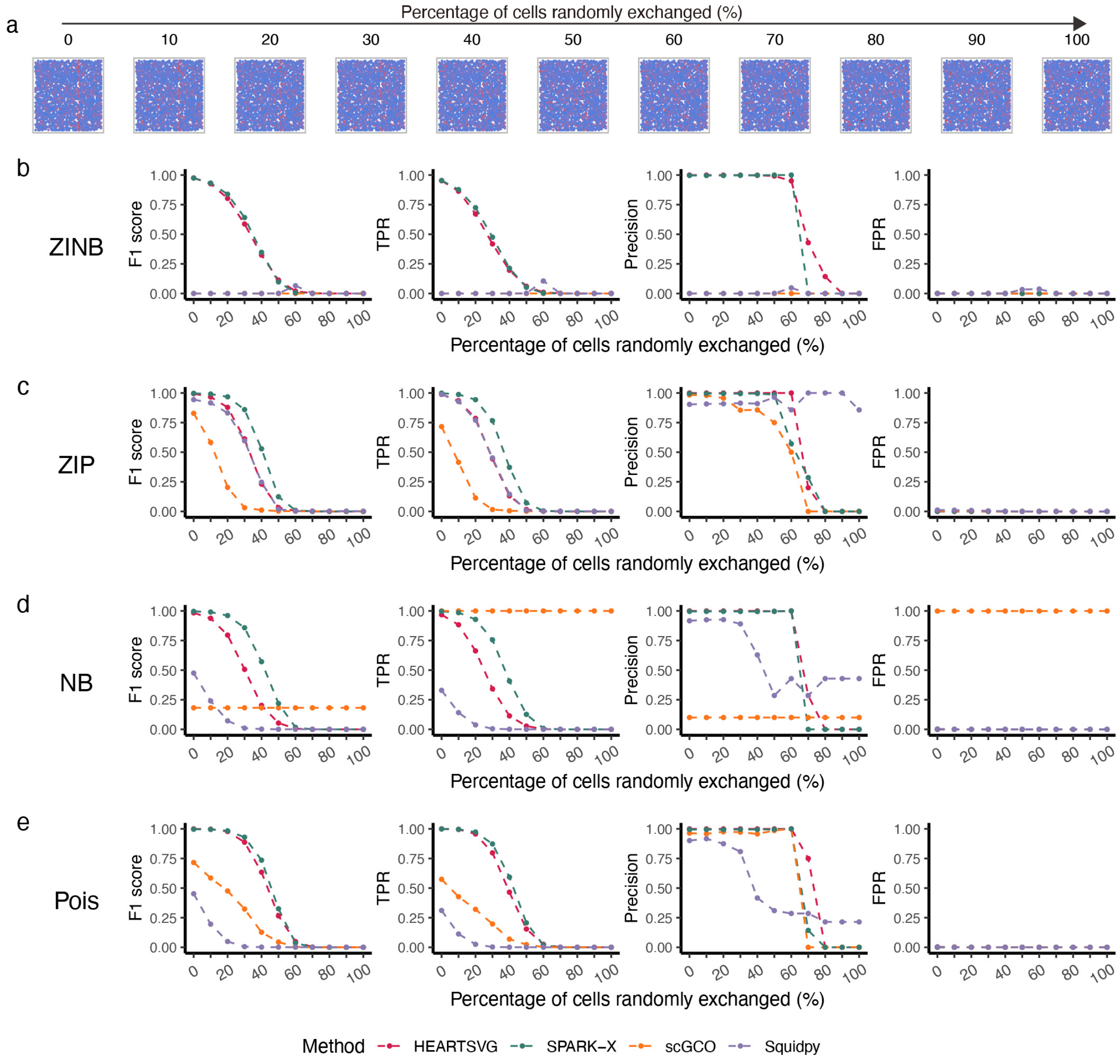


**Figure S67** Simulation results for identifying SVGs using simulated data with noise of 'Randomly Exchanging Expression Values of Selected Nodes.' **a,** Visualization of Pattern: Streak with different percentage of cells random exchanges (%). **b-e,** Simulation results of four different methods (HEARTSVG, scGCO, SPARK-X and Squidpy) on simulated data generated by four distinct distributions (ZINB, ZIP, NB, Poisson). F1 score plots, TPR plots, Precision plots, and FPR plots compare the index values (y-axis) of HEARTSVG (red), scGCO (orange), SPARK-X (green), and Squidpy (purple) across different percentage of cells random exchanges (x-axis). Index values were calculated at the adjusted p-value cutoff of 0.05. Source data are provided with this paper.

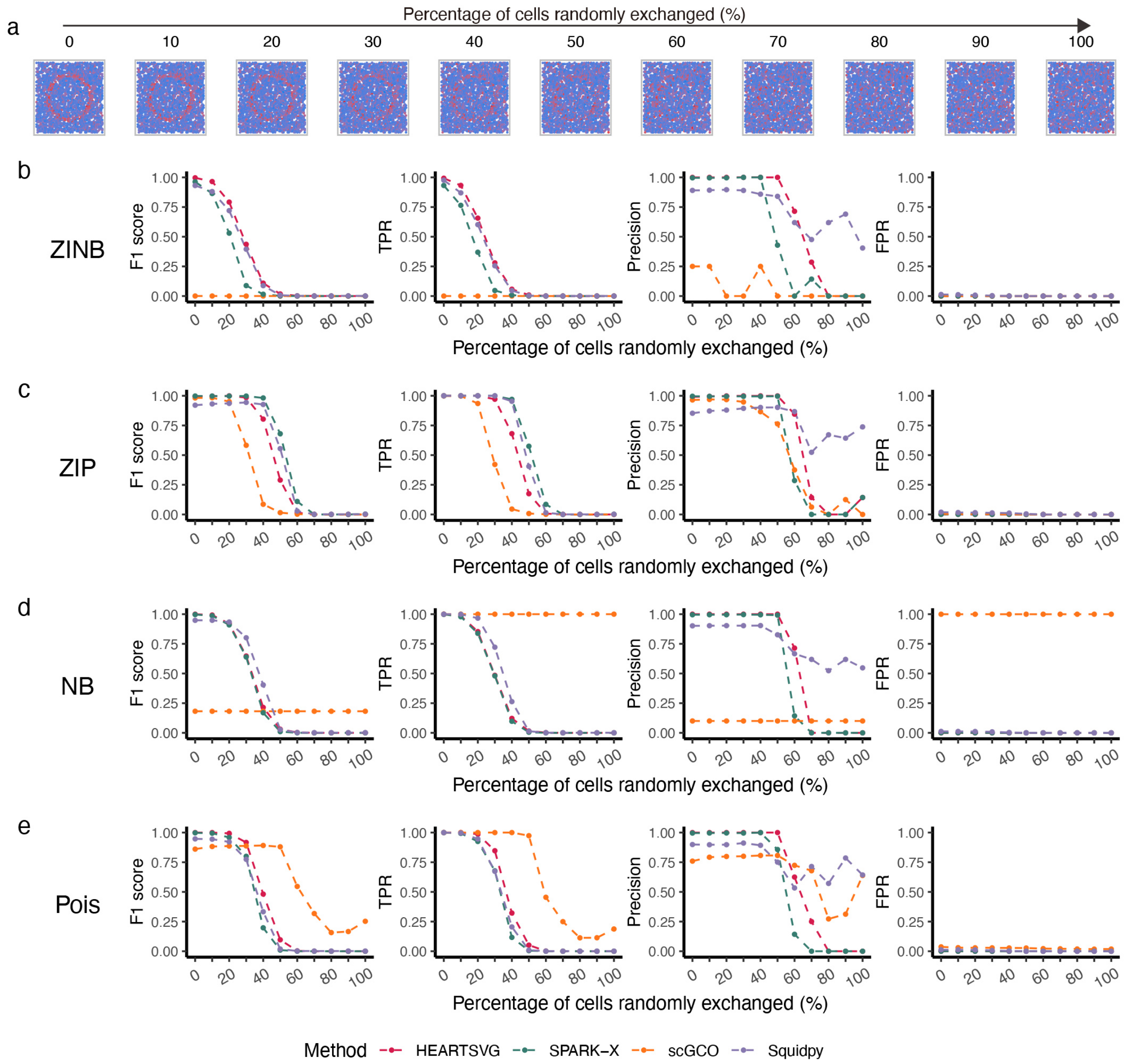


**Figure S68** Simulation results for identifying SVGs using simulated data with noise of 'Randomly Exchanging Expression Values of Selected Nodes.' **a,** Visualization of Pattern: Ring with different percentage of cells random exchanges (%). **b-e,** Simulation results of four different methods (HEARTSVG, scGCO, SPARK-X and Squidpy) on simulated data generated by four distinct distributions (ZINB, ZIP, NB, Poisson). F1 score plots, TPR plots, Precision plots, and FPR plots compare the index values (y-axis) of HEARTSVG (red), scGCO (orange), SPARK-X (green), and Squidpy (purple) across different percentage of cells random exchanges (x-axis). Index values were calculated at the adjusted p-value cutoff of 0.05. Source data are provided with this paper.

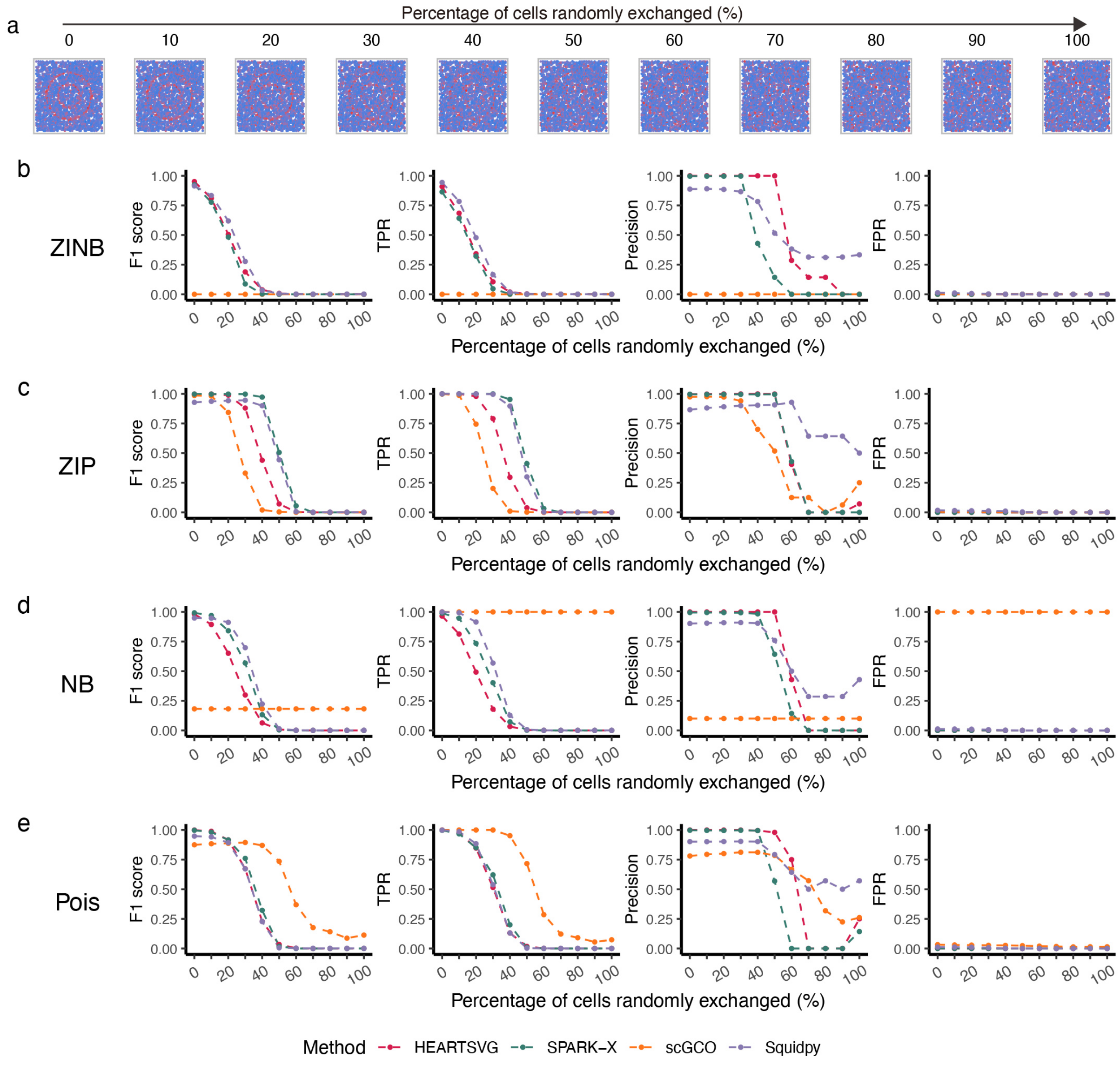


**Figure S69** Simulation results for identifying SVGs using simulated data with noise of 'Randomly Exchanging Expression Values of Selected Nodes.' **a,** Visualization of Pattern: Nested rings with different percentage of cells random exchanges (%). **b-e,** Simulation results of four different methods (HEARTSVG, scGCO, SPARK-X and Squidpy) on simulated data generated by four distinct distributions (ZINB, ZIP, NB, Poisson). F1 score plots, TPR plots, Precision plots, and FPR plots compare the index values (y-axis) of HEARTSVG (red), scGCO (orange), SPARK-X (green), and Squidpy (purple) across different percentage of cells random exchanges (x-axis). Index values were calculated at the adjusted p-value cutoff of 0.05. Source data are provided with this paper.

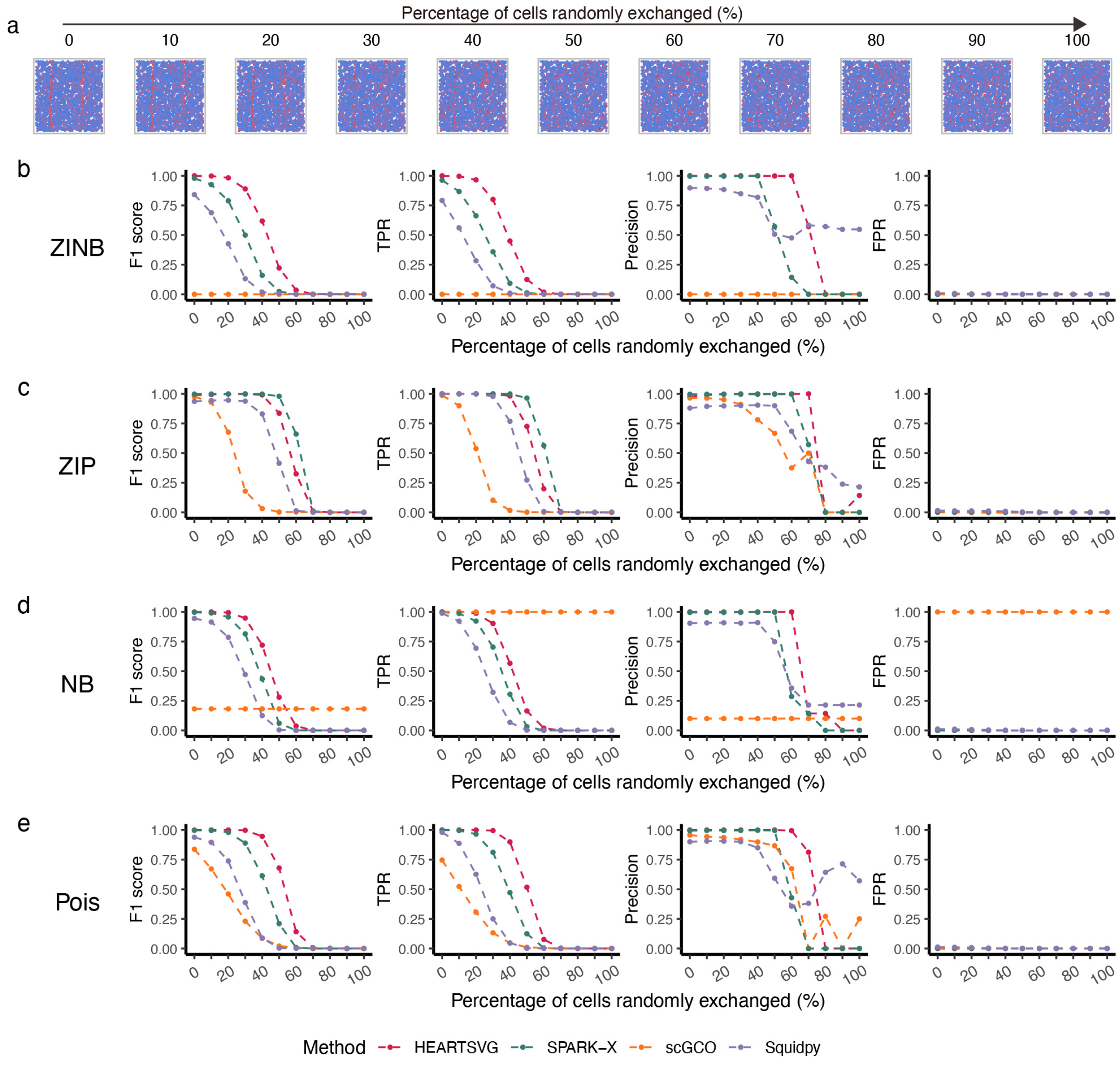


**Figure S70** Simulation results for identifying SVGs using simulated data with noise of 'Randomly Exchanging Expression Values of Selected Nodes.' **a,** Visualization of Pattern: Streaks with different percentage of cells random exchanges (%). **b-e,** Simulation results of four different methods (HEARTSVG, scGCO, SPARK-X and Squidpy) on simulated data generated by four distinct distributions (ZINB, ZIP, NB, Poisson). F1 score plots, TPR plots, Precision plots, and FPR plots compare the index values (y-axis) of HEARTSVG (red), scGCO (orange), SPARK-X (green), and Squidpy (purple) across different percentage of cells random exchanges (x-axis). Index values were calculated at the adjusted p-value cutoff of 0.05. Source data are provided with this paper.

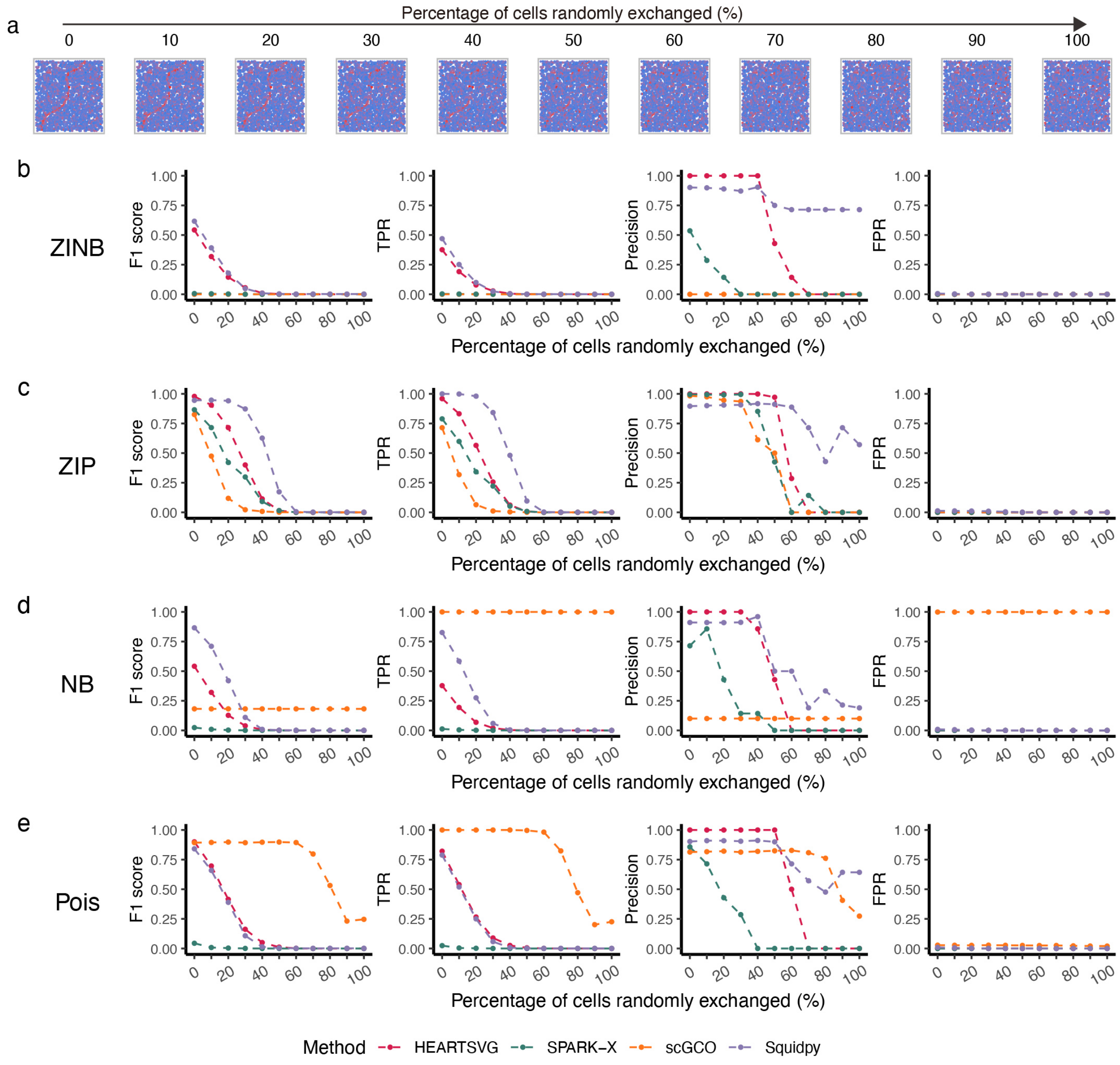


**Figure S71** Simulation results for identifying SVGs using simulated data with noise of 'Randomly Exchanging Expression Values of Selected Nodes.' **a,** Visualization of Pattern: Curve with different percentage of cells random exchanges (%). **b-e,** Simulation results of four different methods (HEARTSVG, scGCO, SPARK-X and Squidpy) on simulated data generated by four distinct distributions (ZINB, ZIP, NB, Poisson). F1 score plots, TPR plots, Precision plots, and FPR plots compare the index values (y-axis) of HEARTSVG (red), scGCO (orange), SPARK-X (green), and Squidpy (purple) across different percentage of cells random exchanges (x-axis). Index values were calculated at the adjusted p-value cutoff of 0.05. Source data are provided with this paper.

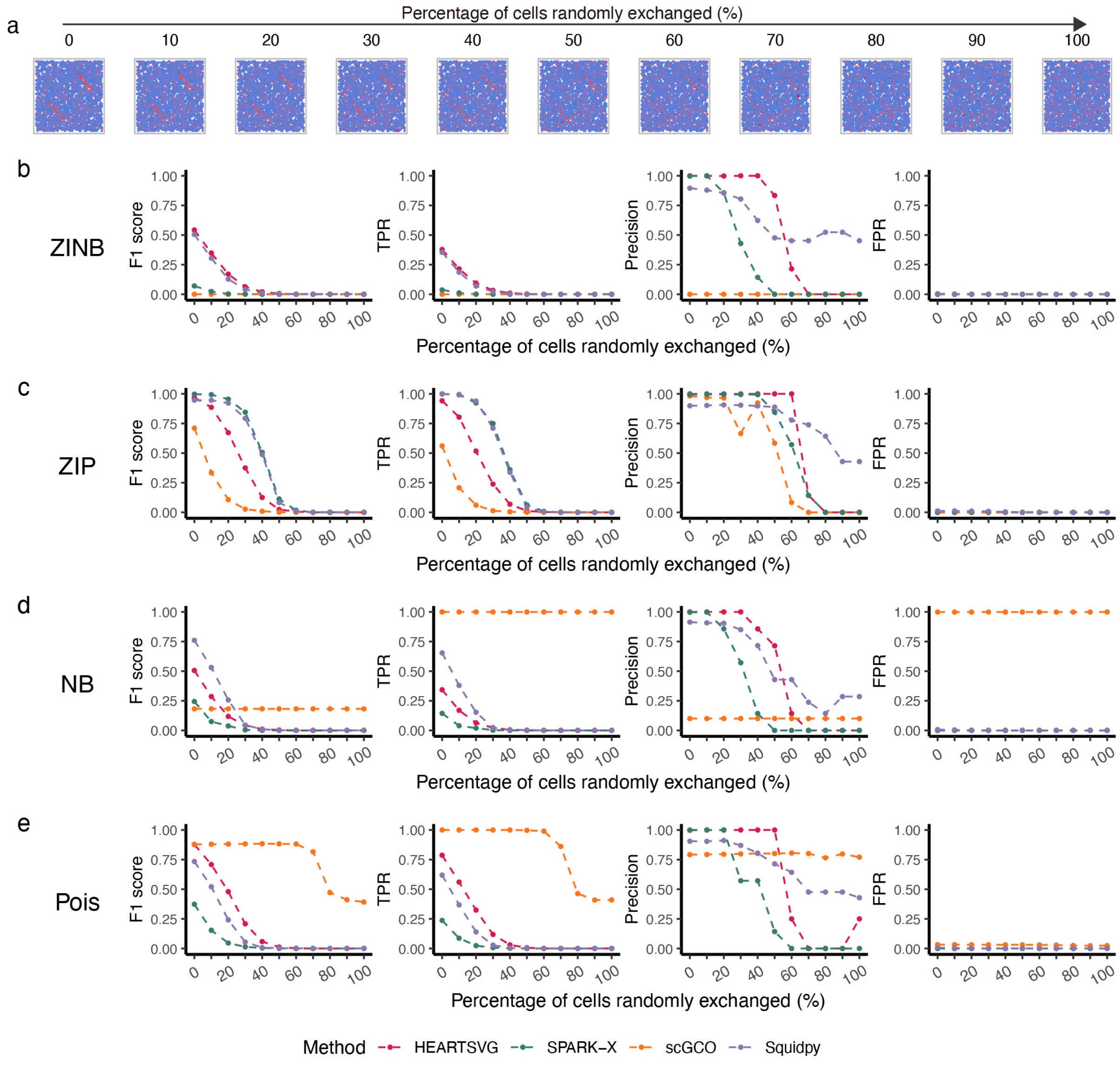


**Figure S72** Simulation results for identifying SVGs using simulated data with noise of 'Randomly Exchanging Expression Values of Selected Nodes.' **a,** Visualization of Pattern: Rectangles with different percentage of cells random exchanges (%). **b-e,** Simulation results of four different methods (HEARTSVG, scGCO, SPARK-X and Squidpy) on simulated data generated by four distinct distributions (ZINB, ZIP, NB, Poisson). F1 score plots, TPR plots, Precision plots, and FPR plots compare the index values (y-axis) of HEARTSVG (red), scGCO (orange), SPARK-X (green), and Squidpy (purple) across different percentage of cells random exchanges (x-axis). Index values were calculated at the adjusted p-value cutoff of 0.05. Source data are provided with this paper.

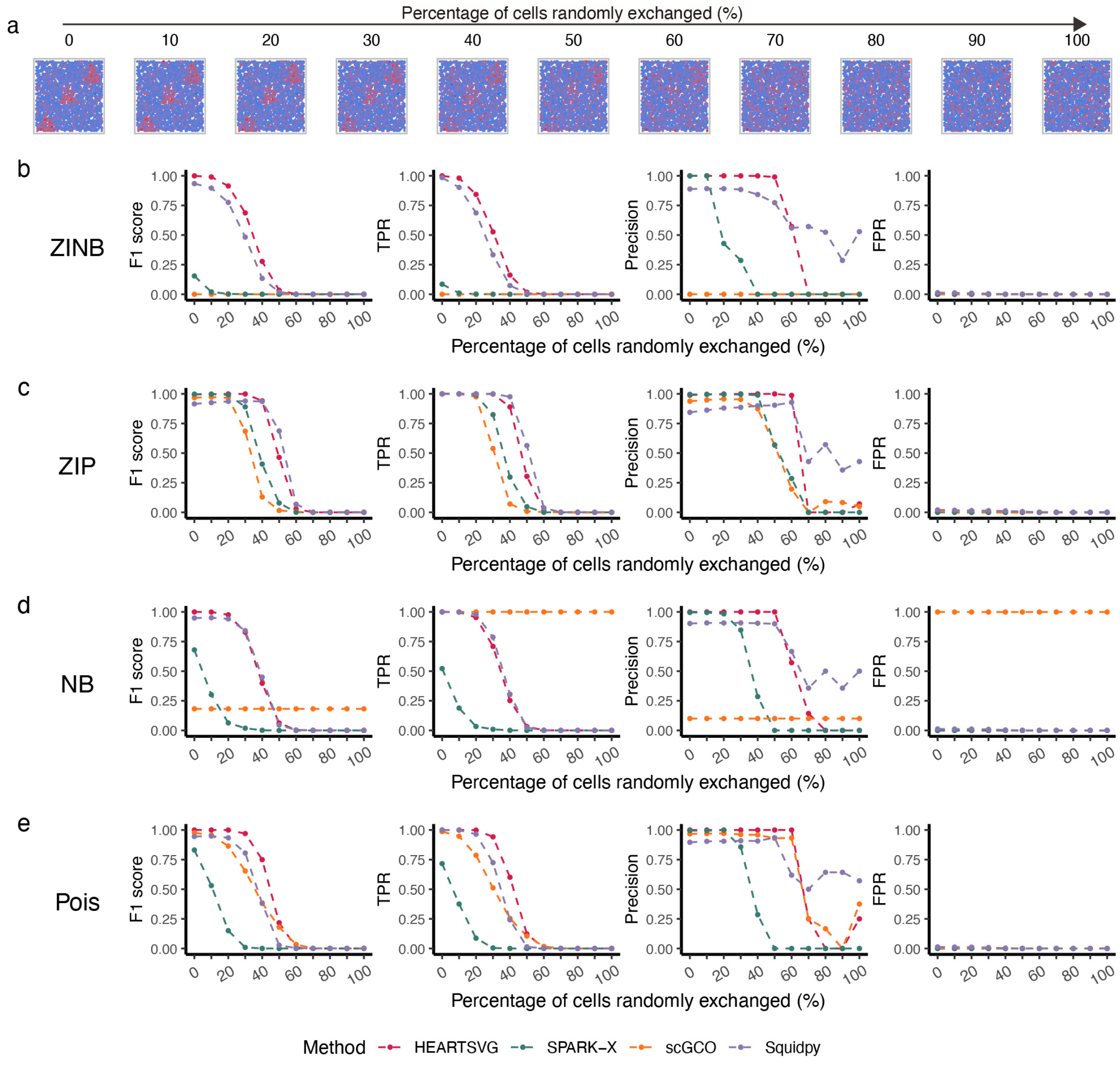


**Figure S73** Simulation results for identifying SVGs using simulated data with noise of 'Randomly Exchanging Expression Values of Selected Nodes.' **a,** Visualization of Pattern: Big triangles with different percentage of cells random exchanges (%). **b-e,** Simulation results of four different methods (HEARTSVG, scGCO, SPARK-X and Squidpy) on simulated data generated by four distinct distributions (ZINB, ZIP, NB, Poisson). F1 score plots, TPR plots, Precision plots, and FPR plots compare the index values (y-axis) of HEARTSVG (red), scGCO (orange), SPARK-X (green), and Squidpy (purple) across different percentage of cells random exchanges (x-axis). Index values were calculated at the adjusted p-value cutoff of 0.05. Source data are provided with this paper.

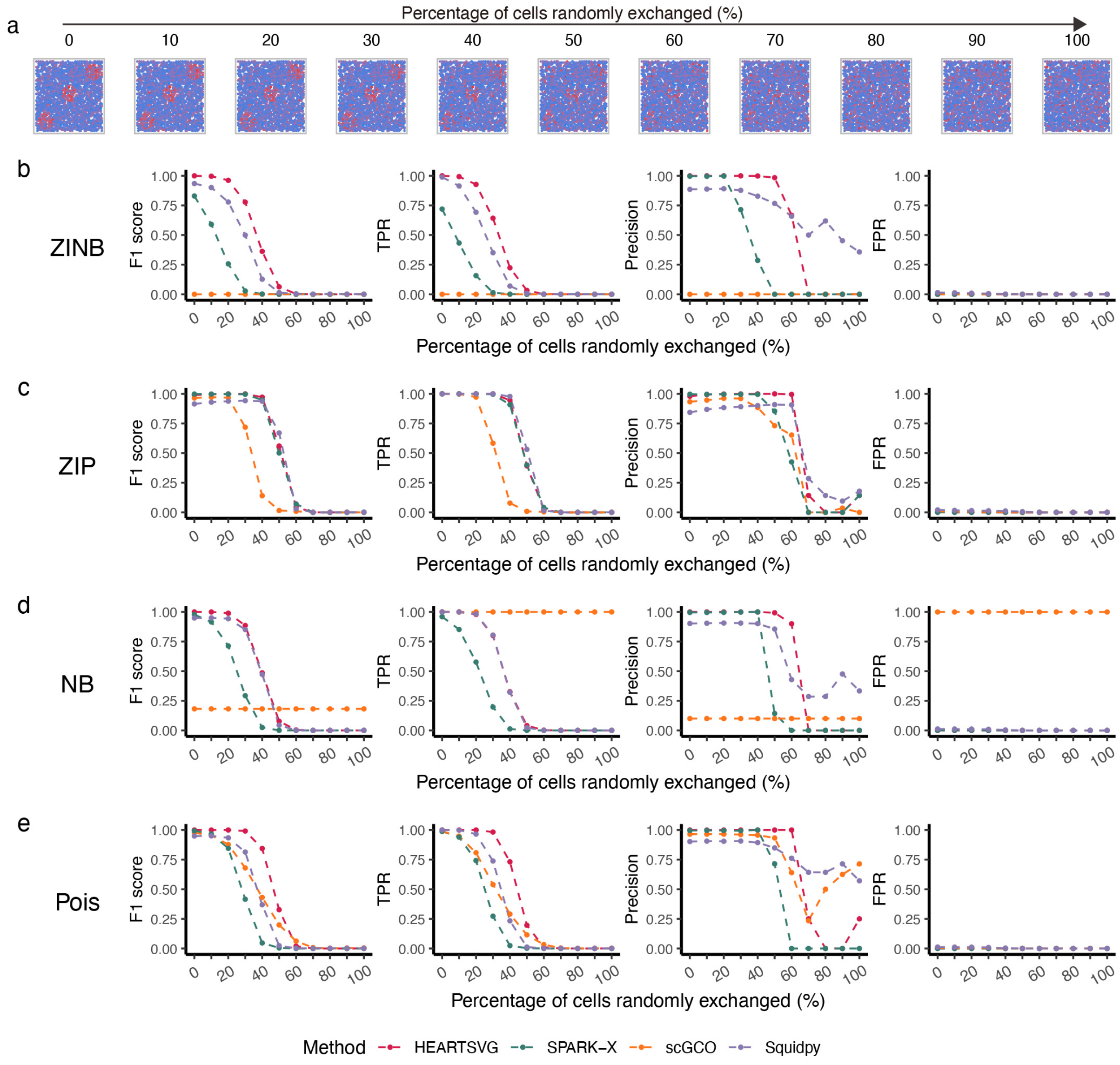


**Figure S74** Simulation results for identifying SVGs using simulated data with noise of 'Randomly Exchanging Expression Values of Selected Nodes.' **a,** Visualization of Pattern: Big circles with different percentage of cells random exchanges (%). **b-e,** Simulation results of four different methods (HEARTSVG, scGCO, SPARK-X and Squidpy) on simulated data generated by four distinct distributions (ZINB, ZIP, NB, Poisson). F1 score plots, TPR plots, Precision plots, and FPR plots compare the index values (y-axis) of HEARTSVG (red), scGCO (orange), SPARK-X (green), and Squidpy (purple) across different percentage of cells random exchanges (x-axis). Index values were calculated at the adjusted p-value cutoff of 0.05. Source data are provided with this paper.

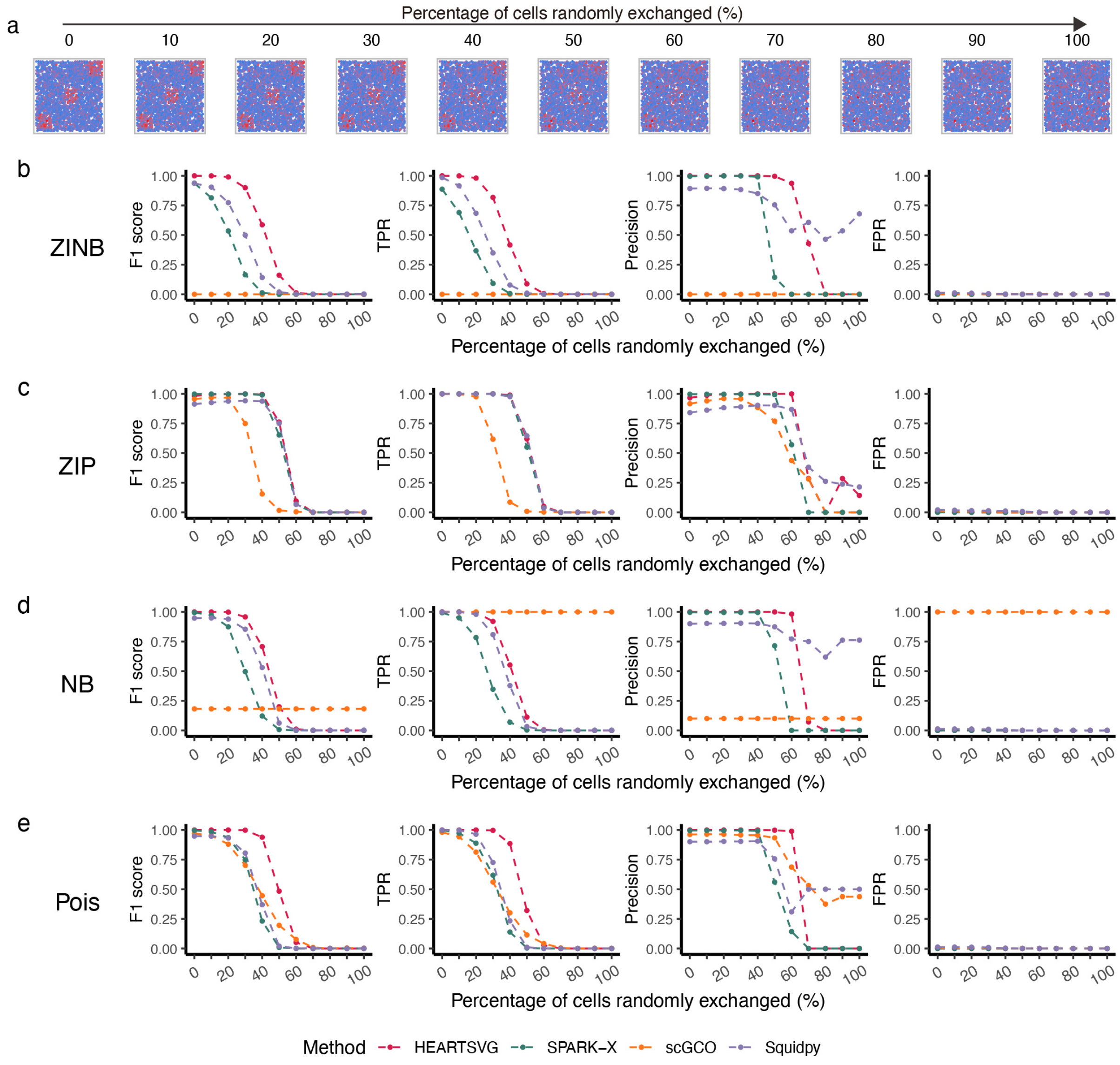


**Figure S75** Simulation results for identifying SVGs using simulated data with noise of 'Randomly Exchanging Expression Values of Selected Nodes.' a, Visualization of Pattern: Big squares with different percentage of cells random exchanges (%). b-e, Simulation results of four different methods (HEARTSVG, scGCO, SPARK-X and Squidpy) on simulated data generated by four distinct distributions (ZINB, ZIP, NB, Poisson). F1 score plots, TPR plots, Precision plots, and FPR plots compare the index values (y-axis) of HEARTSVG (red), scGCO (orange), SPARK-X (green), and Squidpy (purple) across different percentage of cells random exchanges (x-axis). Index values were calculated at the adjusted p-value cutoff of 0.05. Source data are provided with this paper.

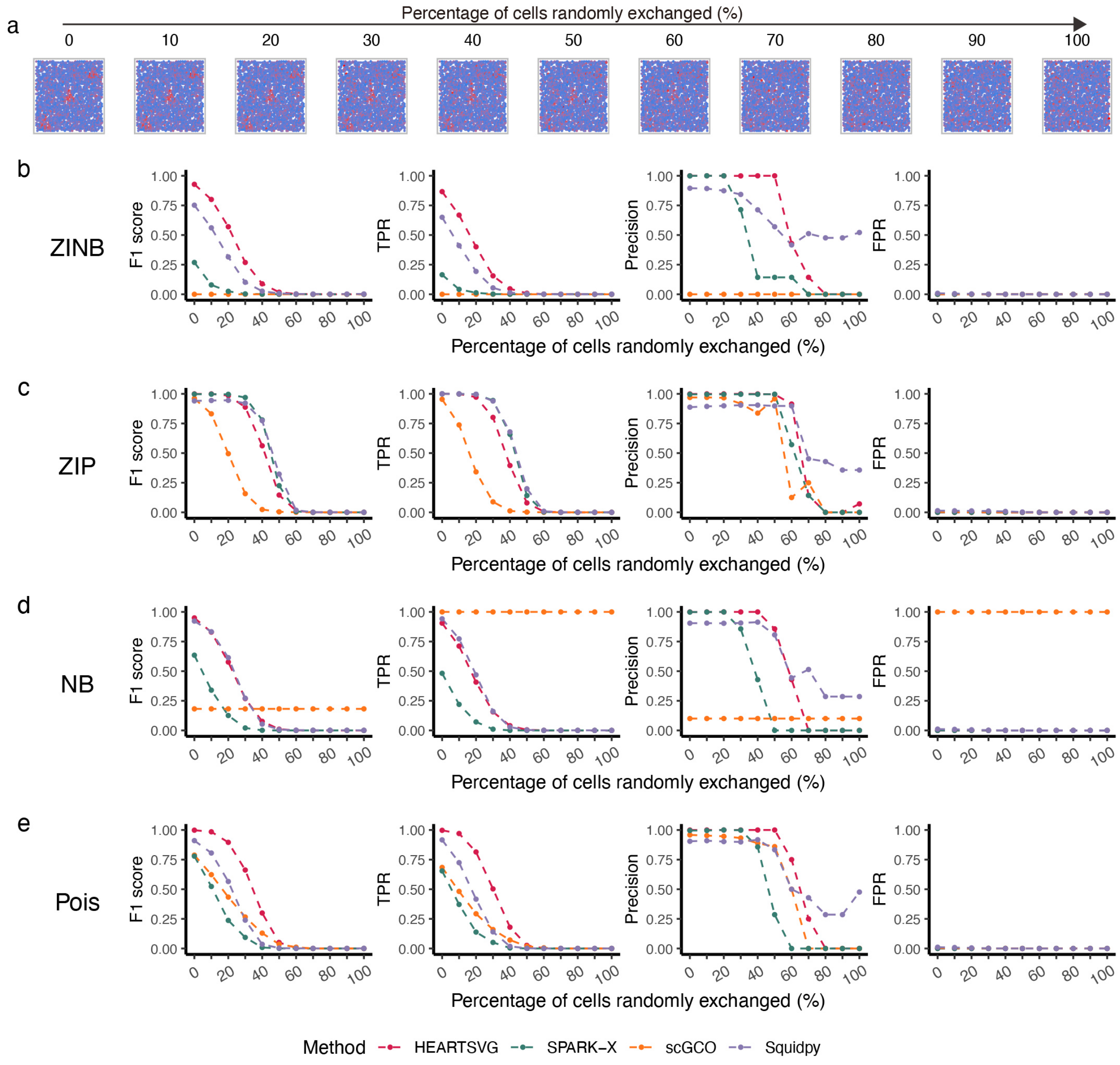


**Figure S76** Simulation results for identifying SVGs using simulated data with noise of 'Randomly Exchanging Expression Values of Selected Nodes.' **a,** Visualization of Pattern: Small triangles with different percentage of cells random exchanges (%). **b-e,** Simulation results of four different methods (HEARTSVG, scGCO, SPARK-X and Squidpy) on simulated data generated by four distinct distributions (ZINB, ZIP, NB, Poisson). F1 score plots, TPR plots, Precision plots, and FPR plots compare the index values (y-axis) of HEARTSVG (red), scGCO (orange), SPARK-X (green), and Squidpy (purple) across different percentage of cells random exchanges (x-axis). Index values were calculated at the adjusted p-value cutoff of 0.05. Source data are provided with this paper.

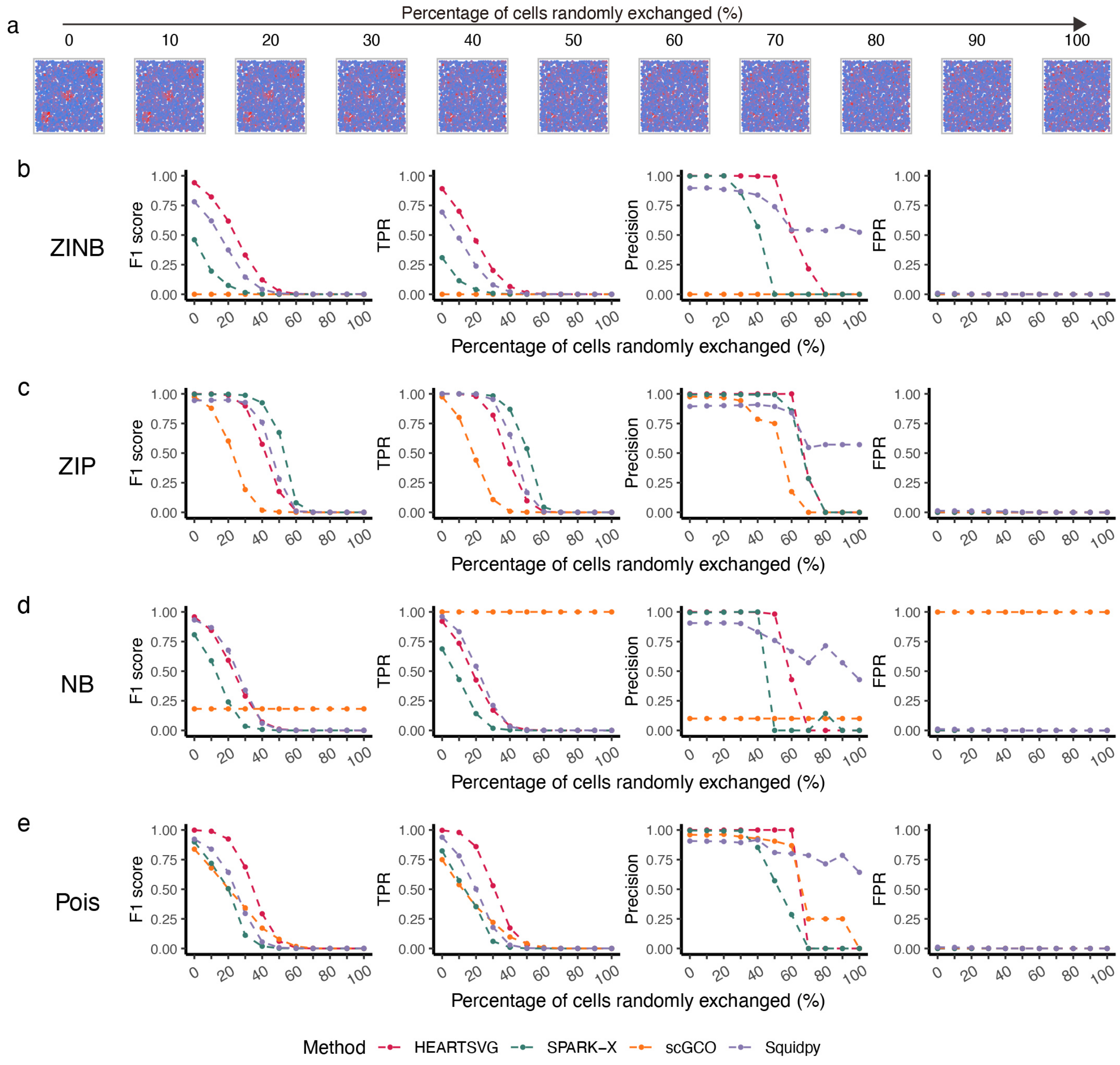


**Figure S77** Simulation results for identifying SVGs using simulated data with noise of 'Randomly Exchanging Expression Values of Selected Nodes.' **a,** Visualization of Pattern: Small circles with different percentage of cells random exchanges (%). **b-e,** Simulation results of four different methods (HEARTSVG, scGCO, SPARK-X and Squidpy) on simulated data generated by four distinct distributions (ZINB, ZIP, NB, Poisson). F1 score plots, TPR plots, Precision plots, and FPR plots compare the index values (y-axis) of HEARTSVG (red), scGCO (orange), SPARK-X (green), and Squidpy (purple) across different percentage of cells random exchanges (x-axis). Index values were calculated at the adjusted p-value cutoff of 0.05. Source data are provided with this paper.

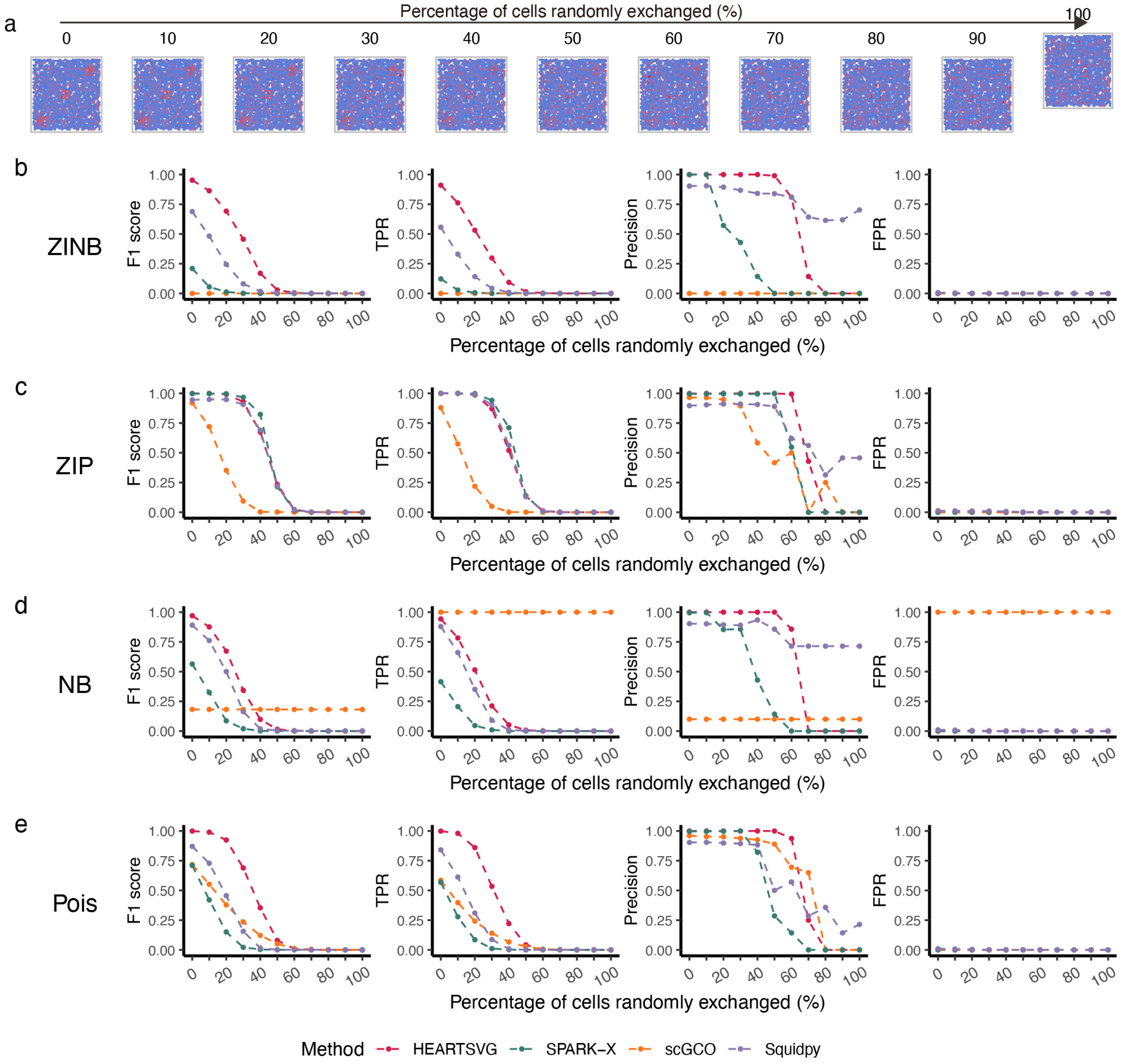


**Figure S78** Simulation results for identifying SVGs using simulated data with noise of 'Randomly Exchanging Expression Values of Selected Nodes.' **a,** Visualization of Pattern: Small squares with different percentage of cells random exchanges (%). **b-e,** Simulation results of four different methods (HEARTSVG, scGCO, SPARK-X and Squidpy) on simulated data generated by four distinct distributions (ZINB, ZIP, NB, Poisson). F1 score plots, TPR plots, Precision plots, and FPR plots compare the index values (y-axis) of HEARTSVG (red), scGCO (orange), SPARK-X (green), and Squidpy (purple) across different percentage of cells random exchanges (x-axis). Index values were calculated at the adjusted p-value cutoff of 0.05. Source data are provided with this paper.

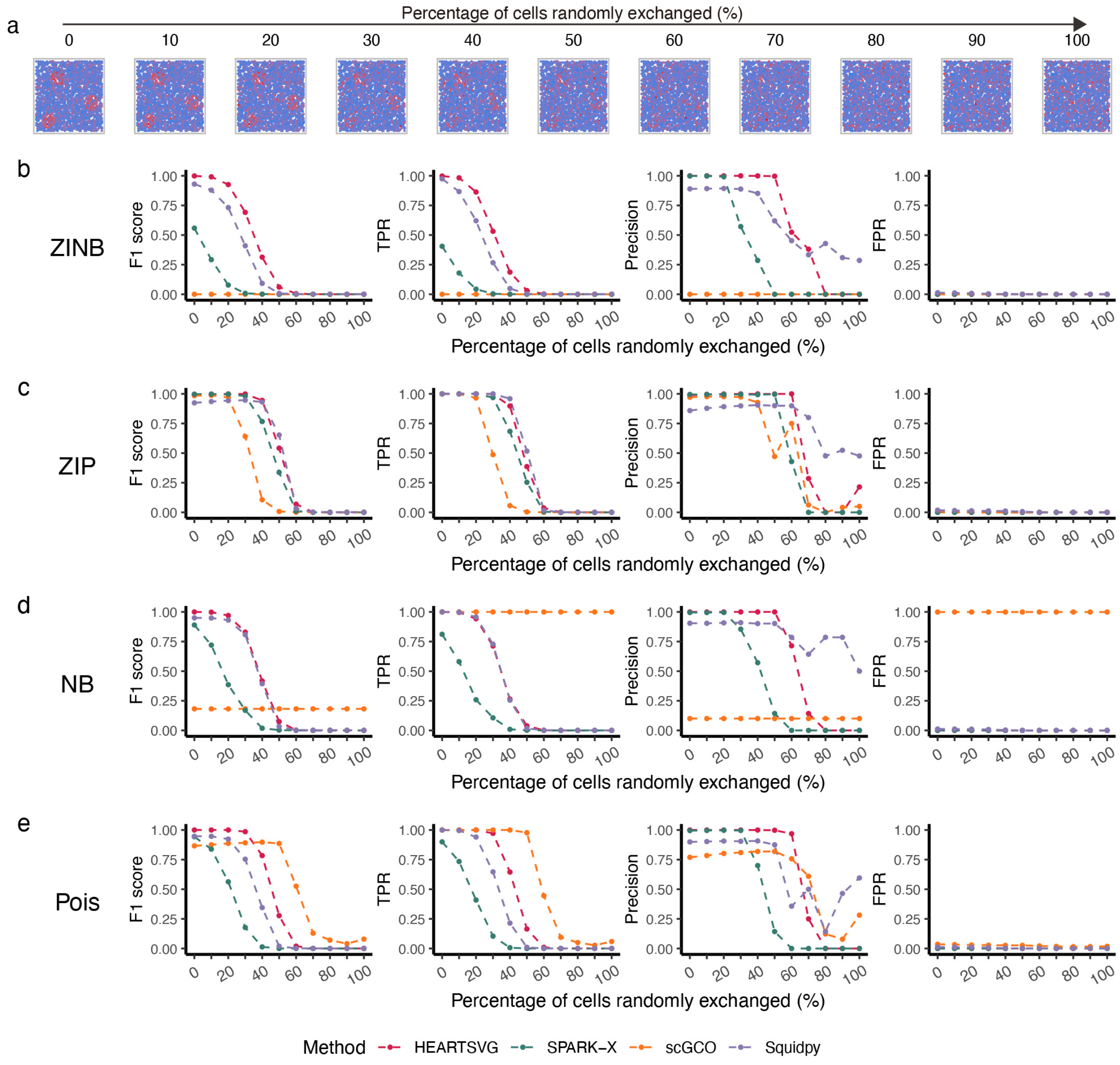


**Figure S79** Simulation results for identifying SVGs using simulated data with noise of 'Randomly Exchanging Expression Values of Selected Nodes.' **a,** Visualization of Pattern: Big circles II with different percentage of cells random exchanges (%). **b-e,** Simulation results of four different methods (HEARTSVG, scGCO, SPARK-X and Squidpy) on simulated data generated by four distinct distributions (ZINB, ZIP, NB, Poisson). F1 score plots, TPR plots, Precision plots, and FPR plots compare the index values (y-axis) of HEARTSVG (red), scGCO (orange), SPARK-X (green), and Squidpy (purple) across different percentage of cells random exchanges (x-axis). Index values were calculated at the adjusted p-value cutoff of 0.05. Source data are provided with this paper.

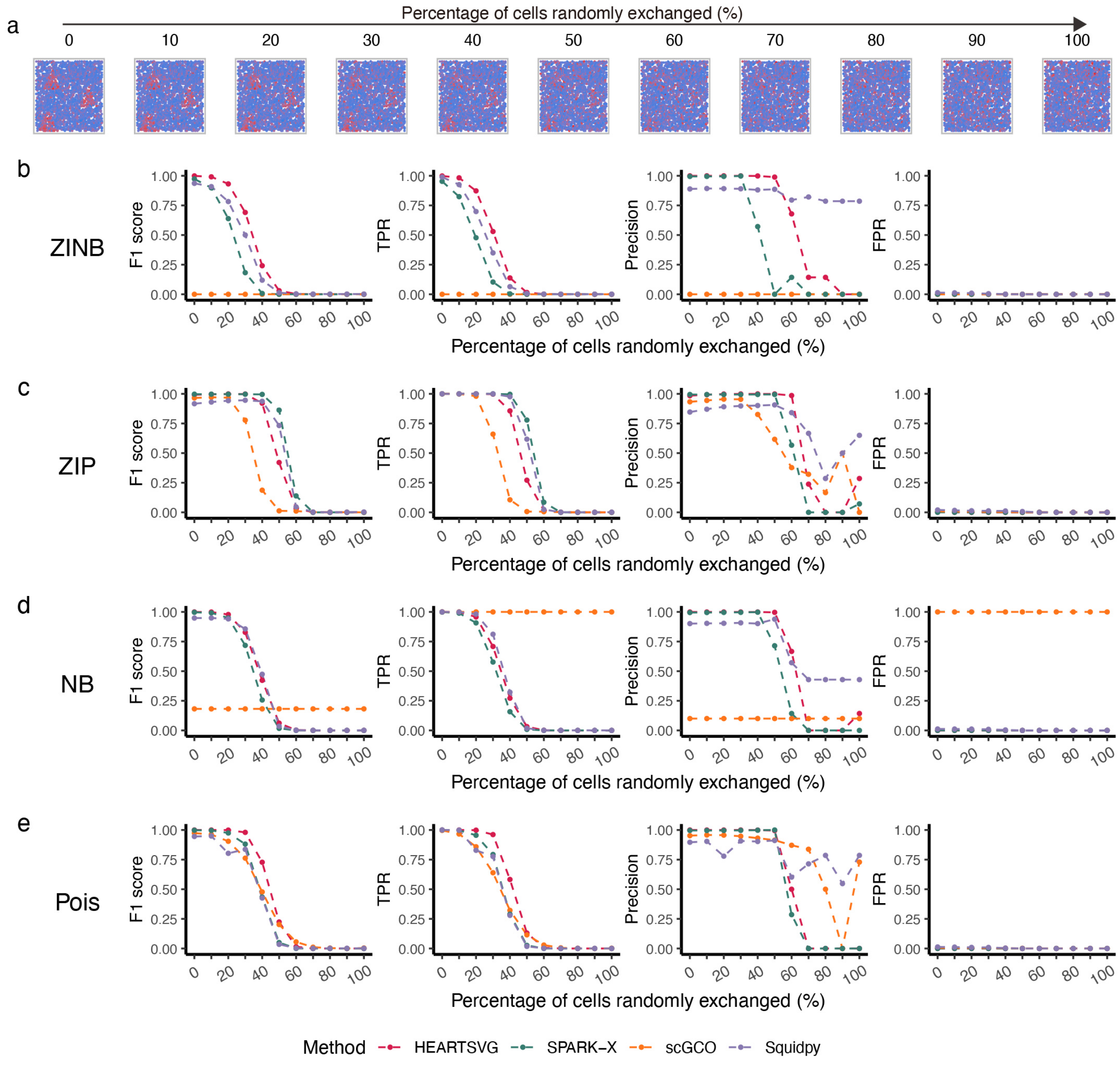


**Figure S80** Simulation results for identifying SVGs using simulated data with noise of 'Randomly Exchanging Expression Values of Selected Nodes.' **a,** Visualization of Pattern: Small triangles II with different percentage of cells random exchanges (%). **b-e,** Simulation results of four different methods (HEARTSVG, scGCO, SPARK-X and Squidpy) on simulated data generated by four distinct distributions (ZINB, ZIP, NB, Poisson). F1 score plots, TPR plots, Precision plots, and FPR plots compare the index values (y-axis) of HEARTSVG (red), scGCO (orange), SPARK-X (green), and Squidpy (purple) across different percentage of cells random exchanges (x-axis). Index values were calculated at the adjusted p-value cutoff of 0.05. Source data are provided with this paper.

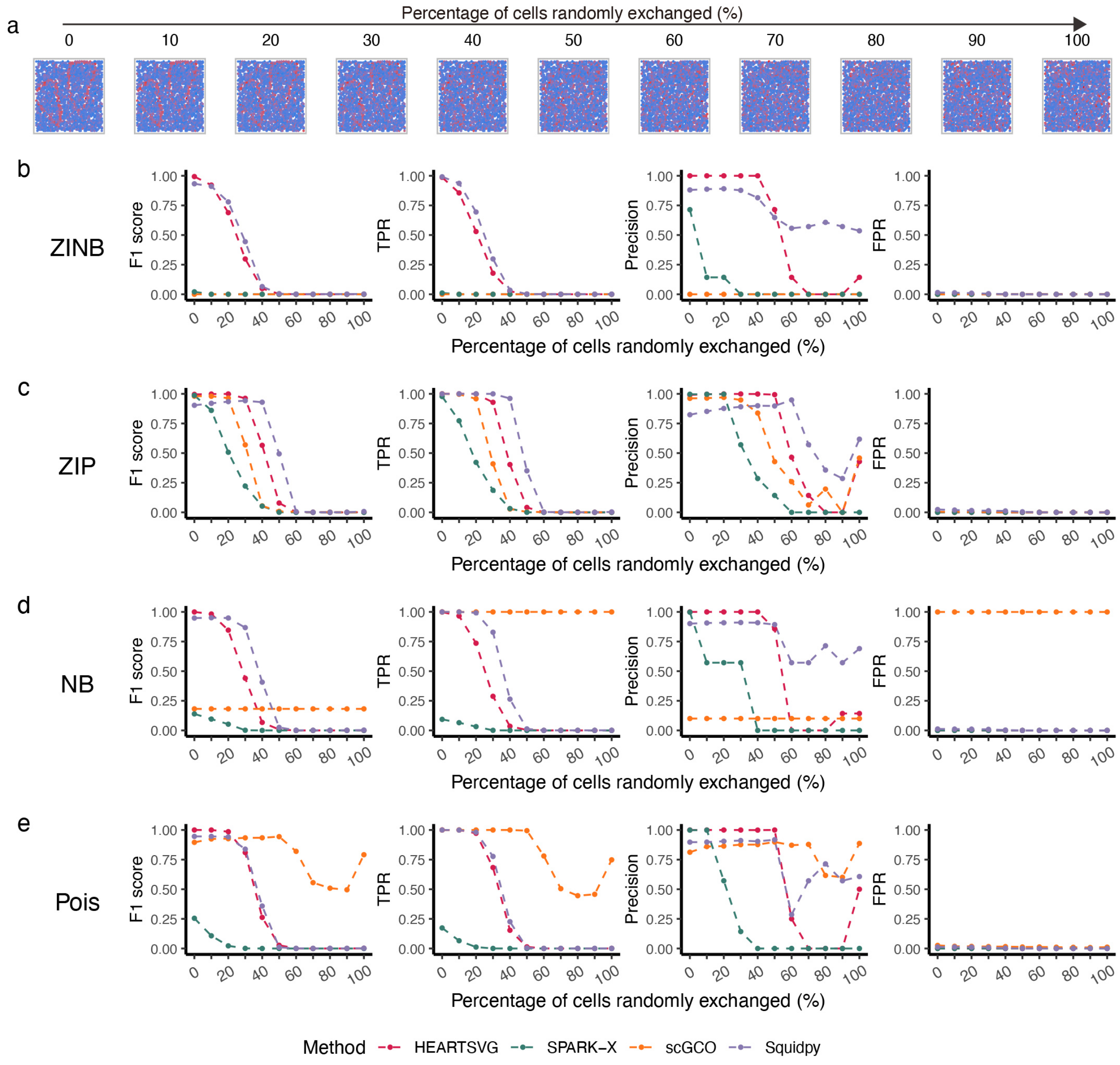


**Figure S81** Simulation results for identifying SVGs using simulated data with noise of 'Randomly Exchanging Expression Values of Selected Nodes.' **a,** Visualization of Pattern: Pattern I with different percentage of cells random exchanges (%). **b-e,** Simulation results of four different methods (HEARTSVG, scGCO, SPARK-X and Squidpy) on simulated data generated by four distinct distributions (ZINB, ZIP, NB, Poisson). F1 score plots, TPR plots, Precision plots, and FPR plots compare the index values (y-axis) of HEARTSVG (red), scGCO (orange), SPARK-X (green), and Squidpy (purple) across different percentage of cells random exchanges (x-axis). Index values were calculated at the adjusted p-value cutoff of 0.05. Source data are provided with this paper.

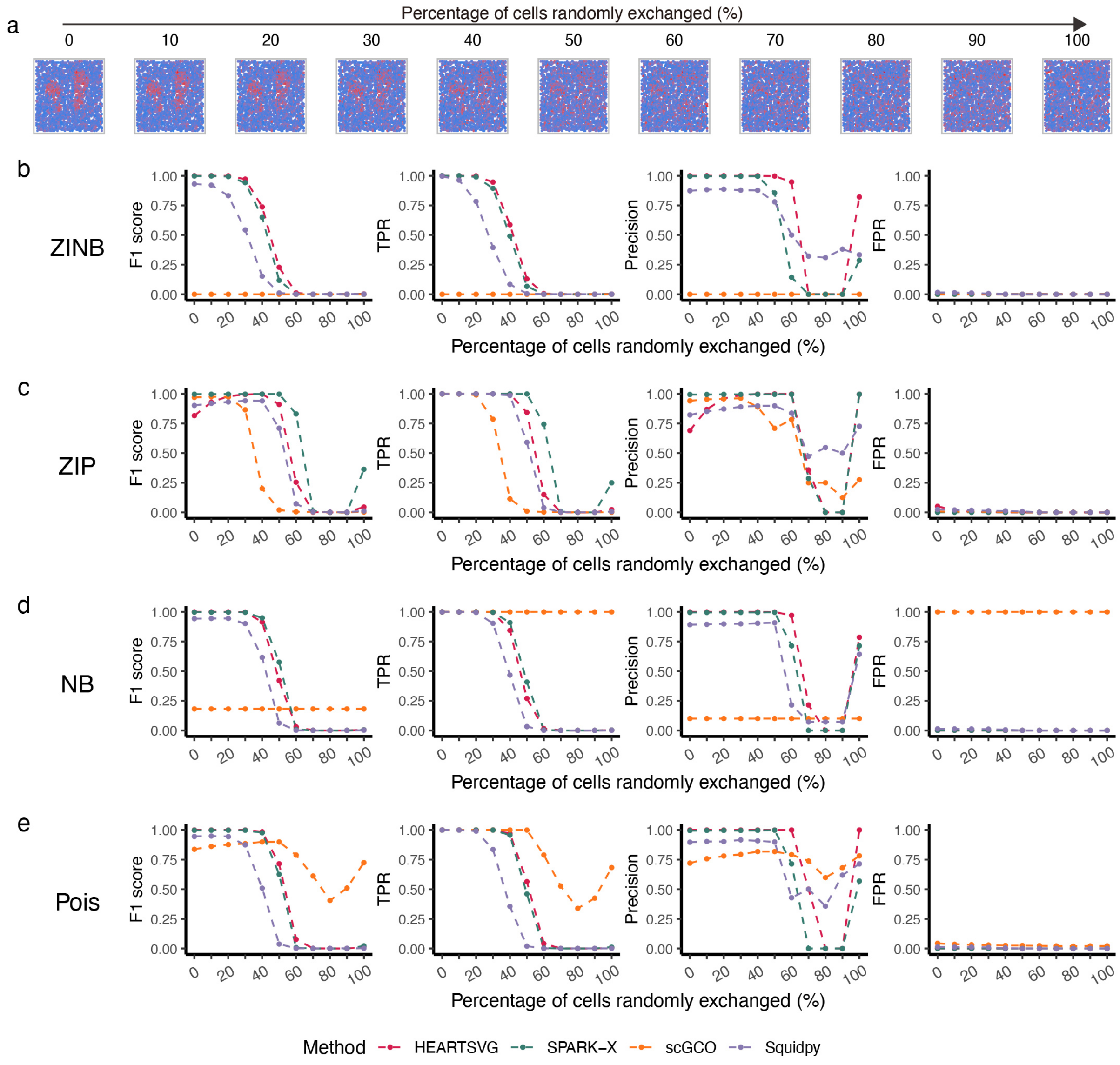


**Figure S82** Simulation results for identifying SVGs using simulated data with noise of 'Randomly Exchanging Expression Values of Selected Nodes.' **a,** Visualization of Pattern: Pattern II with different percentage of cells random exchanges (%). **b-e,** Simulation results of four different methods (HEARTSVG, scGCO, SPARK-X and Squidpy) on simulated data generated by four distinct distributions (ZINB, ZIP, NB, Poisson). F1 score plots, TPR plots, Precision plots, and FPR plots compare the index values (y-axis) of HEARTSVG (red), scGCO (orange), SPARK-X (green), and Squidpy (purple) across different percentage of cells random exchanges (x-axis). Index values were calculated at the adjusted p-value cutoff of 0.05. Source data are provided with this paper.

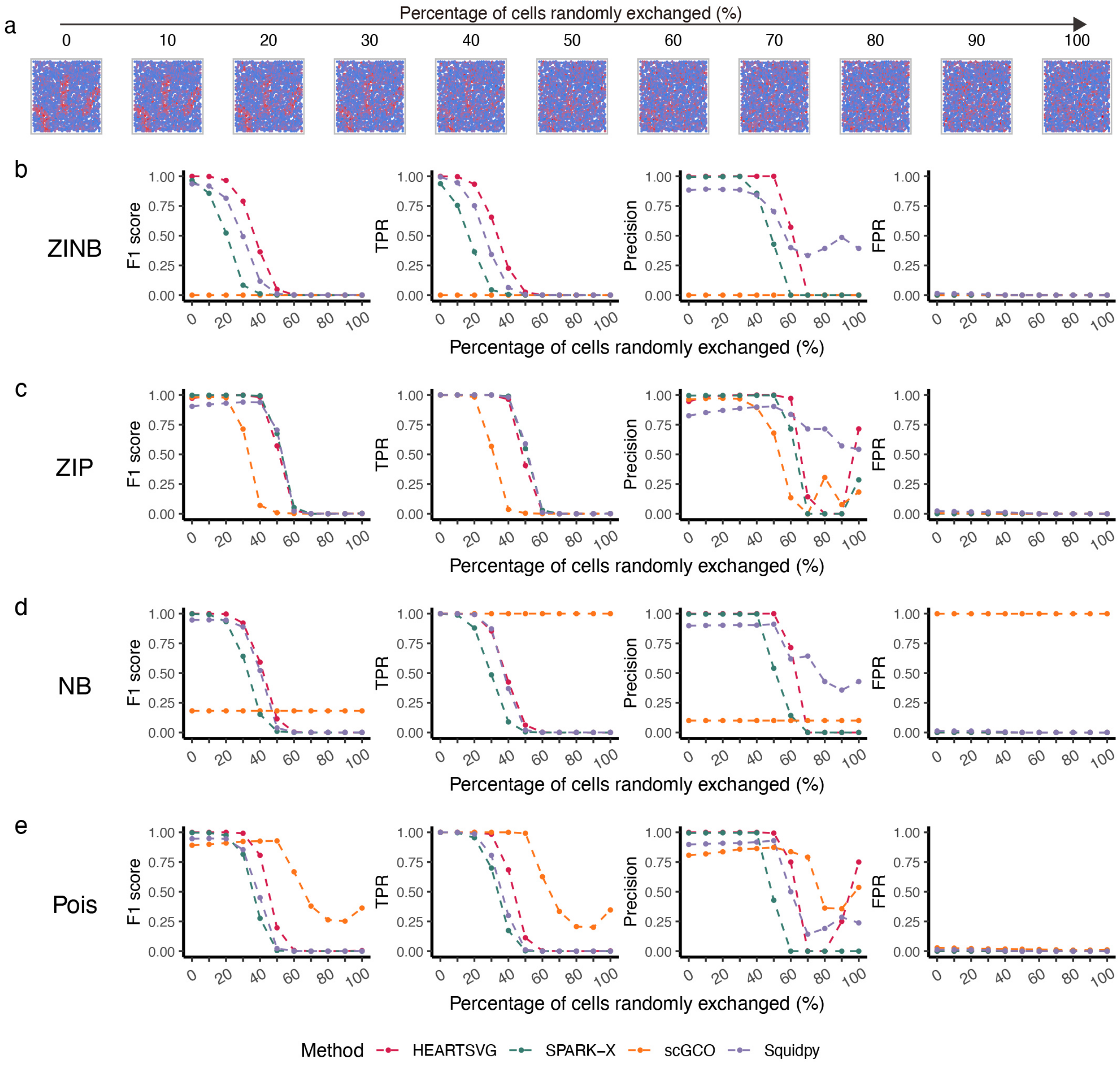


**Figure S83** Simulation results for identifying SVGs using simulated data with noise of 'Randomly Exchanging Expression Values of Selected Nodes.' **a,** Visualization of Pattern: Pattern III with different percentage of cells random exchanges (%). **b-e,** Simulation results of four different methods (HEARTSVG, scGCO, SPARK-X and Squidpy) on simulated data generated by four distinct distributions (ZINB, ZIP, NB, Poisson). F1 score plots, TPR plots, Precision plots, and FPR plots compare the index values (y-axis) of HEARTSVG (red), scGCO (orange), SPARK-X (green), and Squidpy (purple) across different percentage of cells random exchanges (x-axis). Index values were calculated at the adjusted p-value cutoff of 0.05. Source data are provided with this paper.

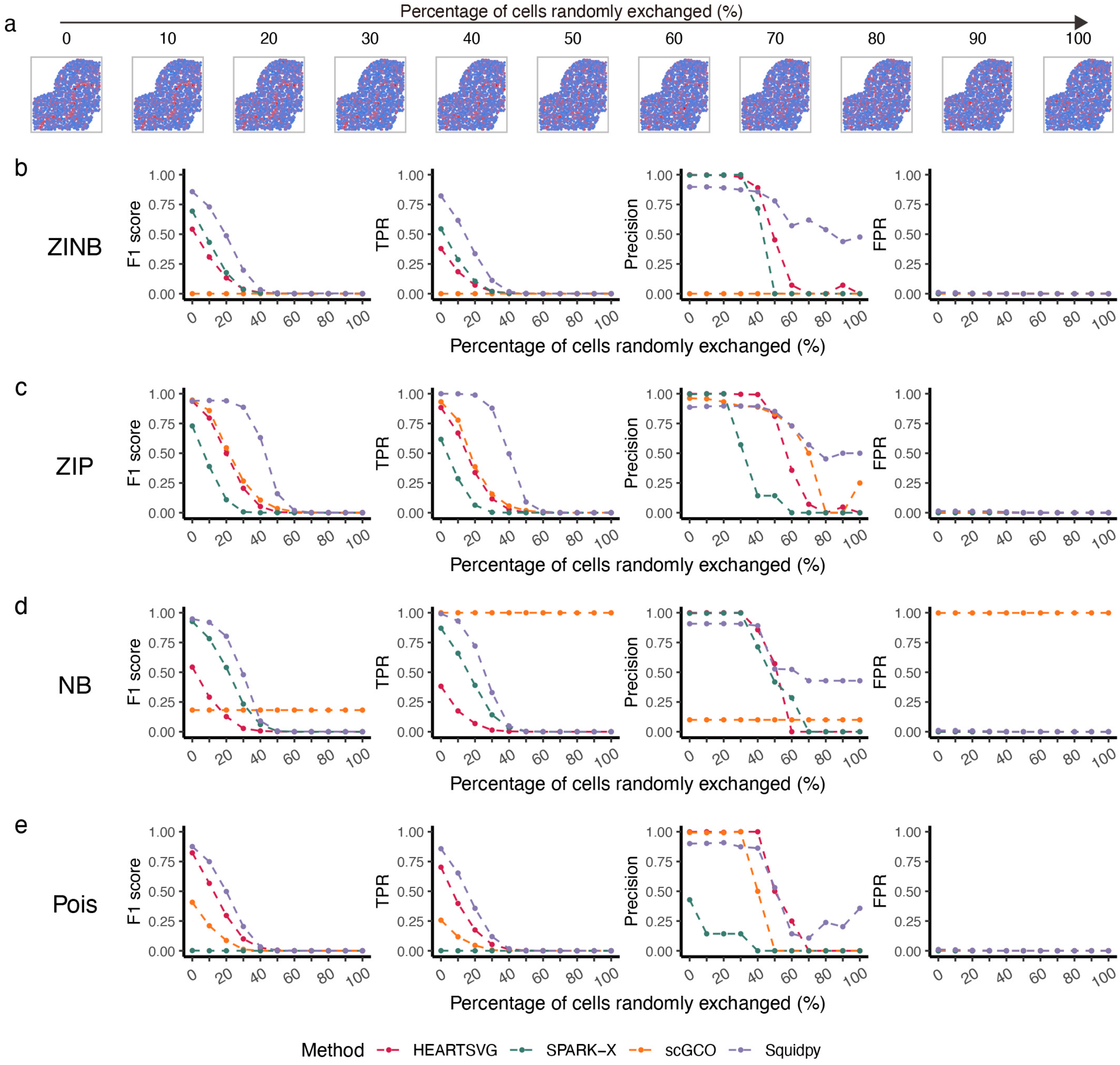


**Figure S84** Simulation results for identifying SVGs using simulated data with noise of 'Randomly Exchanging Expression Values of Selected Nodes.' **a,** Visualization of Pattern: Irreg pat I with different percentage of cells random exchanges (%). **b-e,** Simulation results of four different methods (HEARTSVG, scGCO, SPARK-X and Squidpy) on simulated data generated by four distinct distributions (ZINB, ZIP, NB, Poisson). F1 score plots, TPR plots, Precision plots, and FPR plots compare the index values (y-axis) of HEARTSVG (red), scGCO (orange), SPARK-X (green), and Squidpy (purple) across different percentage of cells random exchanges (x-axis). Index values were calculated at the adjusted p-value cutoff of 0.05. Source data are provided with this paper.

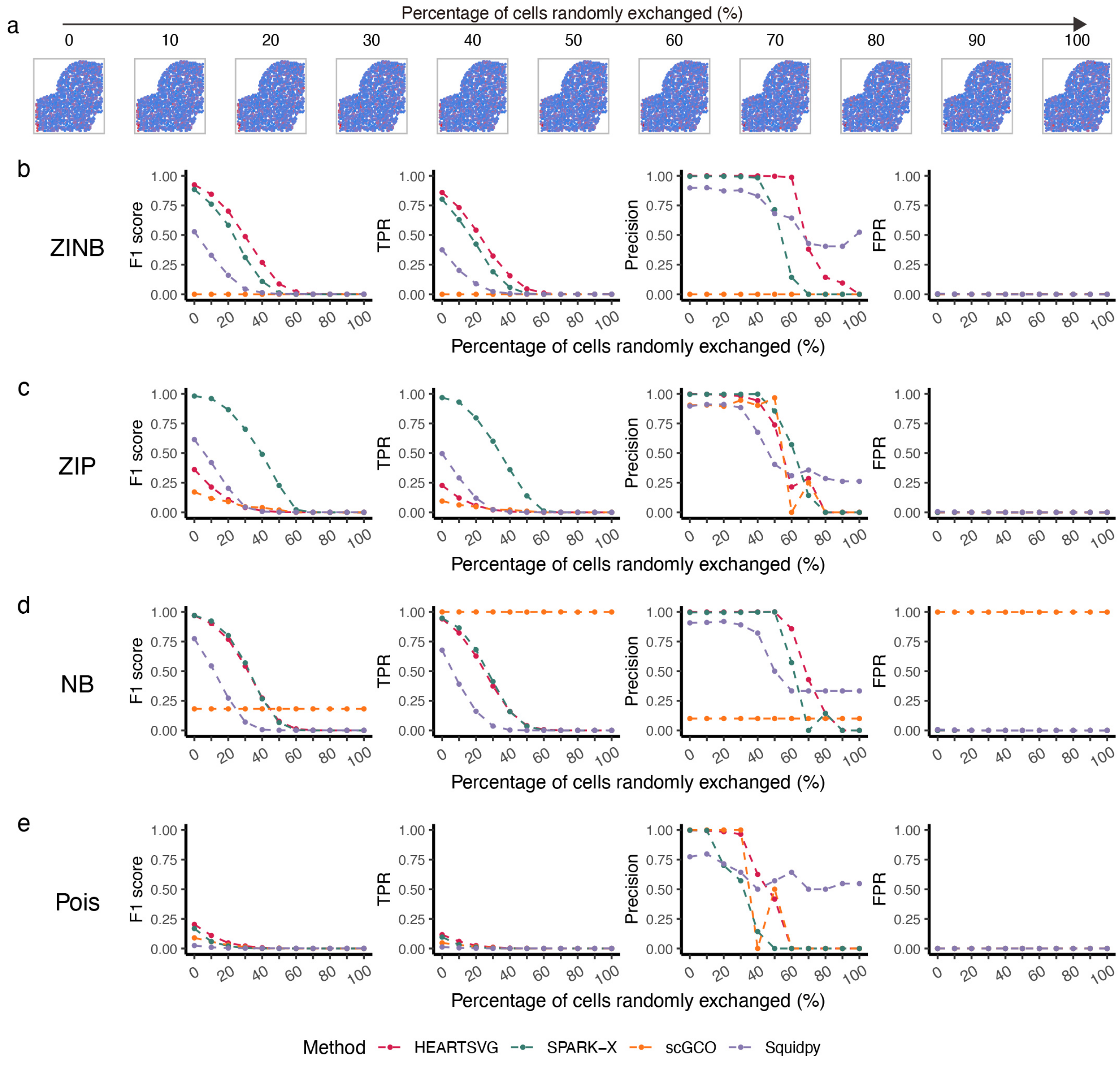


**Figure S85** Simulation results for identifying SVGs using simulated data with noise of 'Randomly Exchanging Expression Values of Selected Nodes.' **a,** Visualization of Pattern: Irreg pat II with different percentage of cells random exchanges (%). **b-e,** Simulation results of four different methods (HEARTSVG, scGCO, SPARK-X and Squidpy) on simulated data generated by four distinct distributions (ZINB, ZIP, NB, Poisson). F1 score plots, TPR plots, Precision plots, and FPR plots compare the index values (y-axis) of HEARTSVG (red), scGCO (orange), SPARK-X (green), and Squidpy (purple) across different percentage of cells random exchanges (x-axis). Index values were calculated at the adjusted p-value cutoff of 0.05. Source data are provided with this paper.

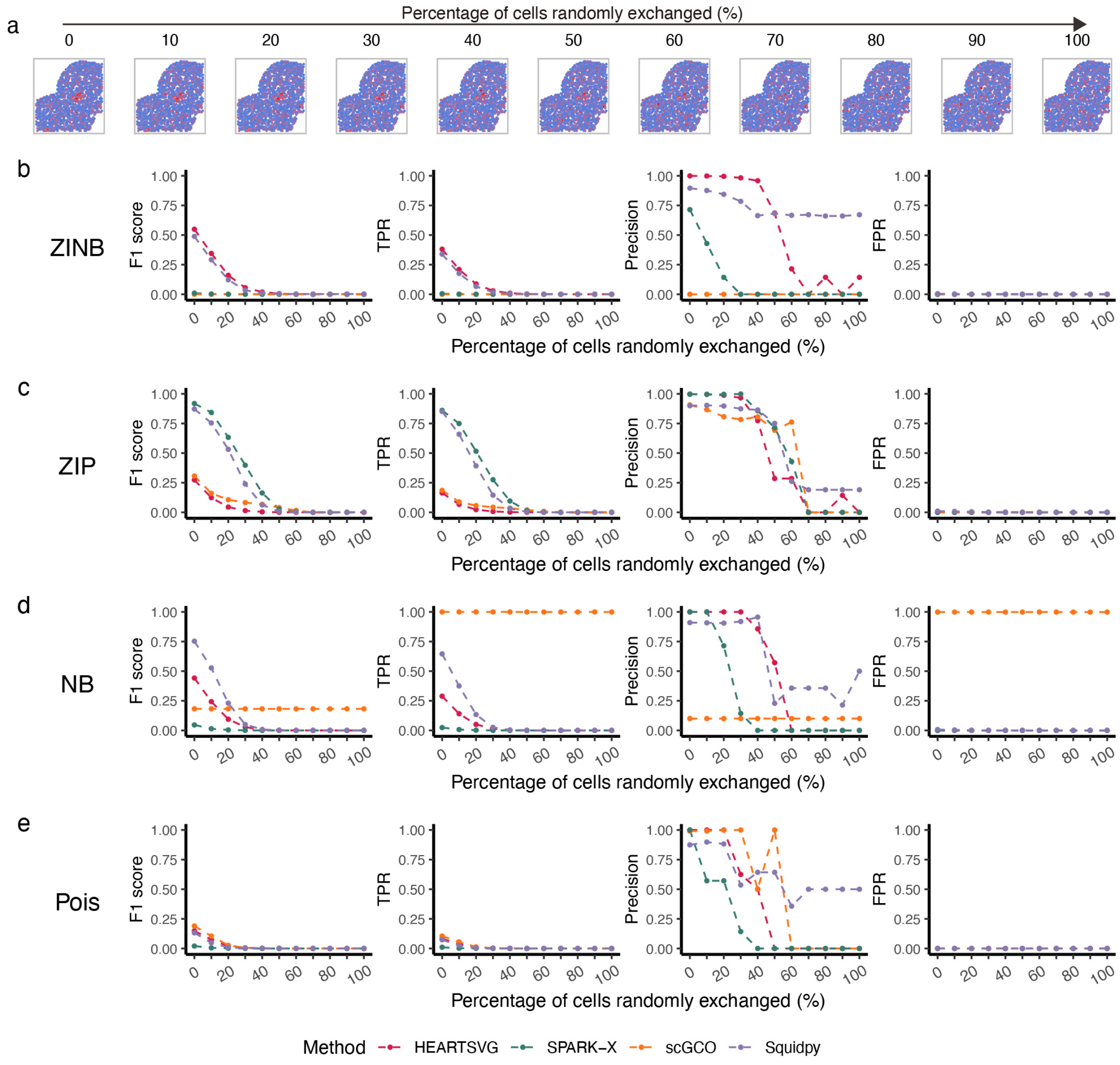


**Figure S86** Simulation results for identifying SVGs using simulated data with noise of 'Randomly Exchanging Expression Values of Selected Nodes.' **a,** Visualization of Pattern: Irreg pat III with different percentage of cells random exchanges (%). **b-e,** Simulation results of four different methods (HEARTSVG, scGCO, SPARK-X and Squidpy) on simulated data generated by four distinct distributions (ZINB, ZIP, NB, Poisson). F1 score plots, TPR plots, Precision plots, and FPR plots compare the index values (y-axis) of HEARTSVG (red), scGCO (orange), SPARK-X (green), and Squidpy (purple) across different percentage of cells random exchanges (x-axis). Index values were calculated at the adjusted p-value cutoff of 0.05. Source data are provided with this paper.

## 10.5 The normalization procedures by spatialDE and SPARK distorts the data characteristics, negatively affecting SVG detection.

In our simulations, SPARK and SpatialDE perform noticeably weaker compared to other methods. Both SPARK and SpatialDE use the **Gaussian process regression as the underlying data model**. It is well-known that spatial transcriptomics data do not follow a normal distribution. SPARK and SpatialDE employ additional normalization mechanisms to approximate the spatial transcriptomics data to a normal distribution before modeling and identifying SVGs[1]. However, the normalization mechanism of SPARK and SpatialDE removes excessive heterogeneity, including signals from SVGs, which limits their ability to identify SVGs. Fig. S9 displays SVGs' visualizations before and after SPARK normalization. These visualizations showed the effect of normalization mechanism on spatial gene expression data. The normalization mechanisms of SPARK and SpatialDE overcorrected the signals of SVGs. Nevertheless, to facilitate a comprehensive comparison of various methods, we still created **a new simulation with higher heterogeneity**. This simulated data possesses increased heterogeneity in order to mitigate the impact of normalization mechanisms. We maintained the expression distribution and parameters constant, while incorporating variations such as higher expression in the central circle for some SVG genes, and similar expression across three circles for others, as shown in Figure S10. Upon increasing the heterogeneity in the simulated dat**a,** SPARK and SpatialDE's performance improved, albeit still not on par with other methods. This was particularly evident in datasets with higher sparsity and dispersion (NB and ZINB), aligning with the findings reported for SPARK-X2 in the literature. Notably, enhancing the heterogeneity did not significantly alter the performance of the other methods compared to results from the previous revision.

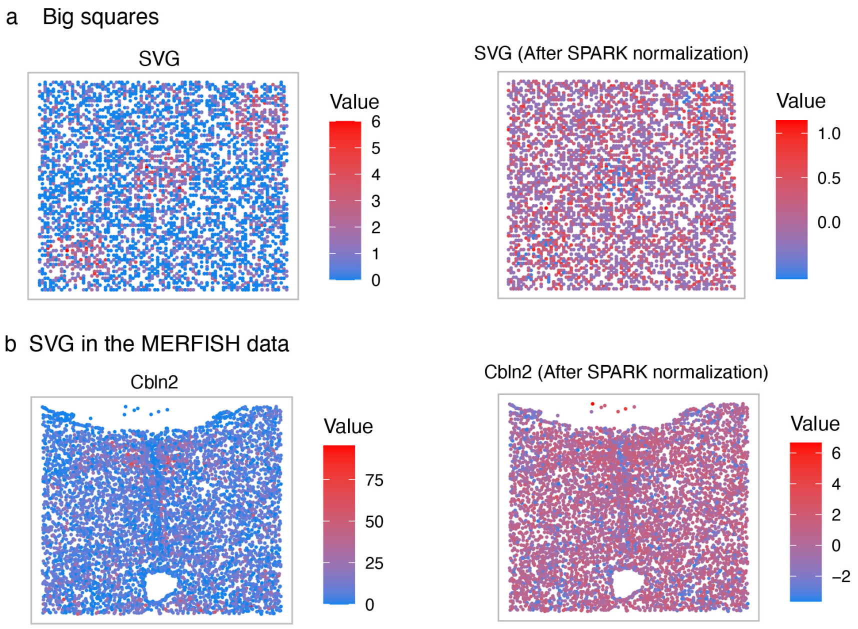


**Figure S87** **a,** SVGs' visualization of Big squares in the simulated data. **b,** SVG in the MERFISH data. The left plot shows the original spatial expression for SVGs. The right plot shows the spatial expression for SVGs after SPARK normalization. Source data are provided with this paper.

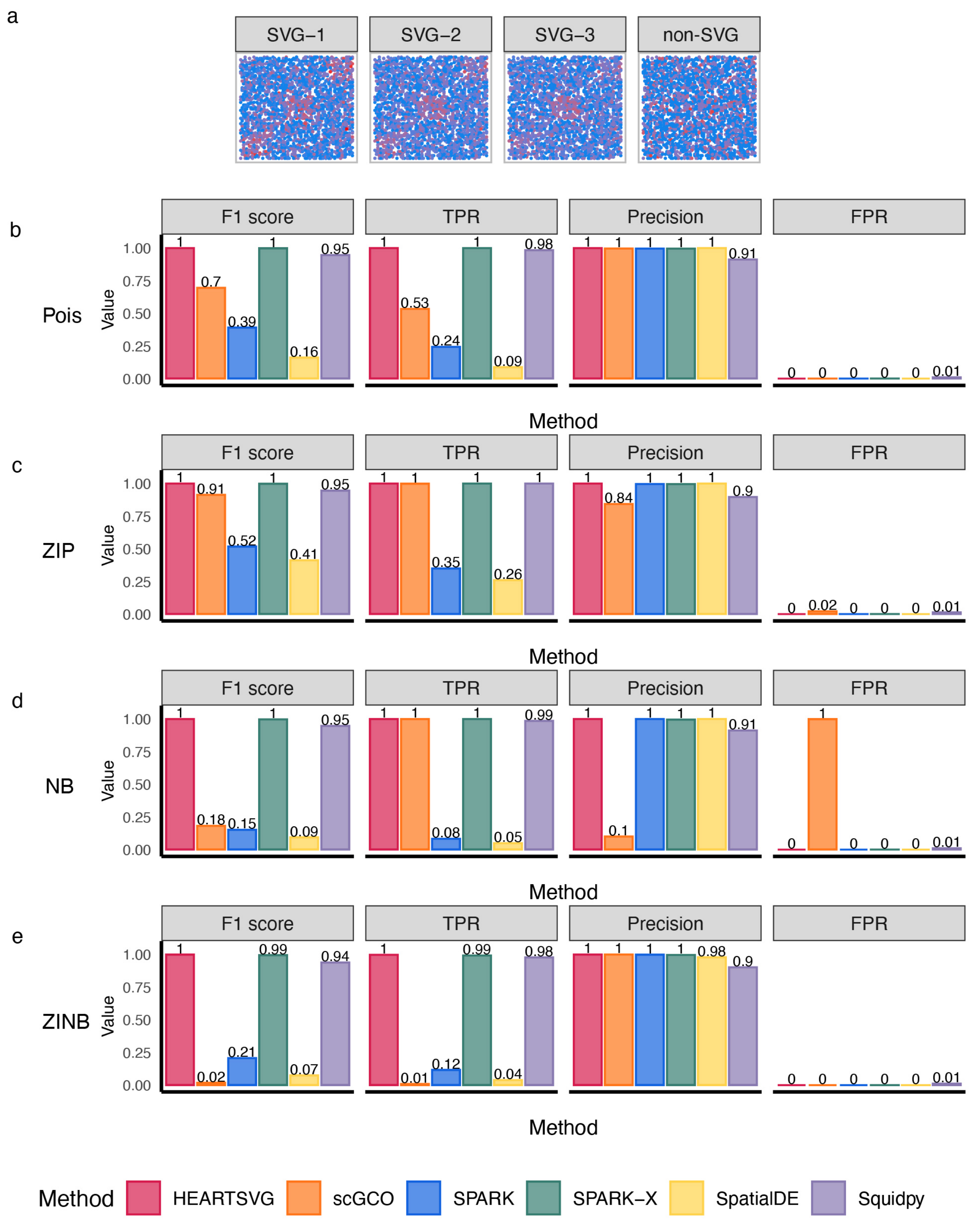


**Figure S88** Simulation results for identifying SVGs using simulated data with higher heterogeneity **a,** Visualization of SVGs and non-SVG. b-e, Simulation results of six different methods (HEARTSVG, scGCO, SPARK, SPARK-X, SpatialDE and Squidpy) on simulated data generated by four distinct distributions (ZINB, ZIP, NB, Poisson). The bar diagram shows F1 scores, TPRs, precisions, and FPRs. Index values were calculated at the adjusted p-value cutoff of 0.05. Source data are provided with this paper.

# 11. Sensitivity analyses of data characteristics

The data characteristics of different distributions (expression levels, degree of dispersion, and sparsity) significantly affect the performance of various methods in identifying SVGs. We conducted sensitivity analyses to pinpoint which steps in the analysis are particularly sensitive to variations in data characteristics.

## 11.1. Data characteristics similar, performance similar

Our analysis shows that when the data characteristics are aligned, each method's performance is relatively stable across different simulated datasets, regardless of the underlying distribution (Fig. S89-S91). We have added two new simulations in which we adjusted the parameters of all distributions (ZINB, ZIP, NB, Pois) to ensure the data characteristics (mean, dispersion, and sparsity levels) produced are similar. It is important to note that, due to the inherent properties of the distributions, ZIP and ZINB will inherently exhibit greater dispersion than Pois and NB under similar mean and variability levels. In simulated datasets with higher dispersion ($ZIP, ZINB$), scGCO showed lower $F_1$ $scores$. Furthermore, in simulated datasets with higher sparsity and lower expression levels (*mean level 0.25*), the SVG identification capabilities of all methods diminished.

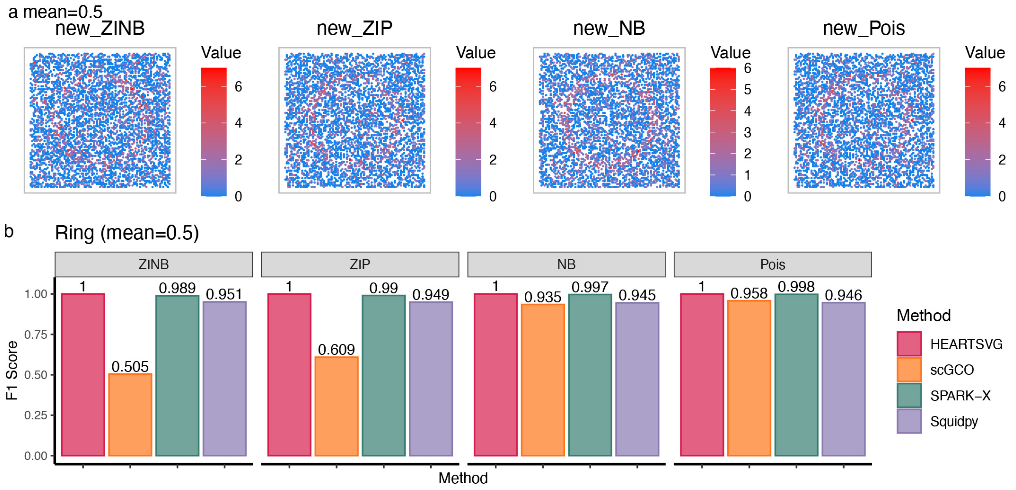


**Figure S89** **a,** Visualization of Ring Pattern for different distributions that share similar data characteristics (mean, dispersion, and sparsity level). **b,** Each method has similar $F_1$ $score$ across different simulated datasets. scGCO has lower $F_1$ $score$ on datasets characterized by higher dispersion, such as those from $ZIP$ and $ZINB$ distributions. Source data are provided with this paper.

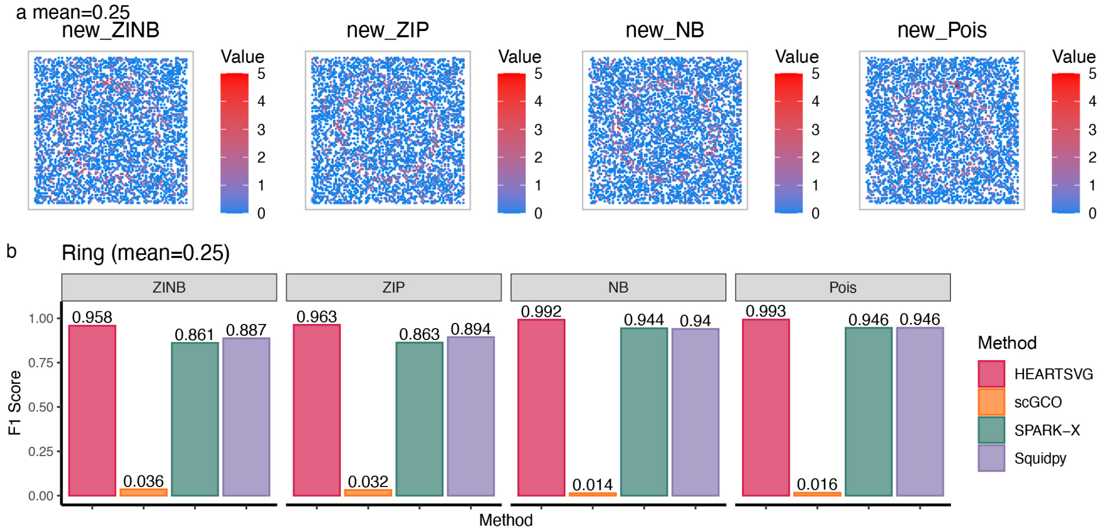


**Figure S90** **a,** Visualization of Ring Pattern for different distributions that share similar data characteristics (mean, dispersion, and sparsity level). b. Each method has similar $F_1$ *score* across different simulated dataset. Source data are provided with this paper.

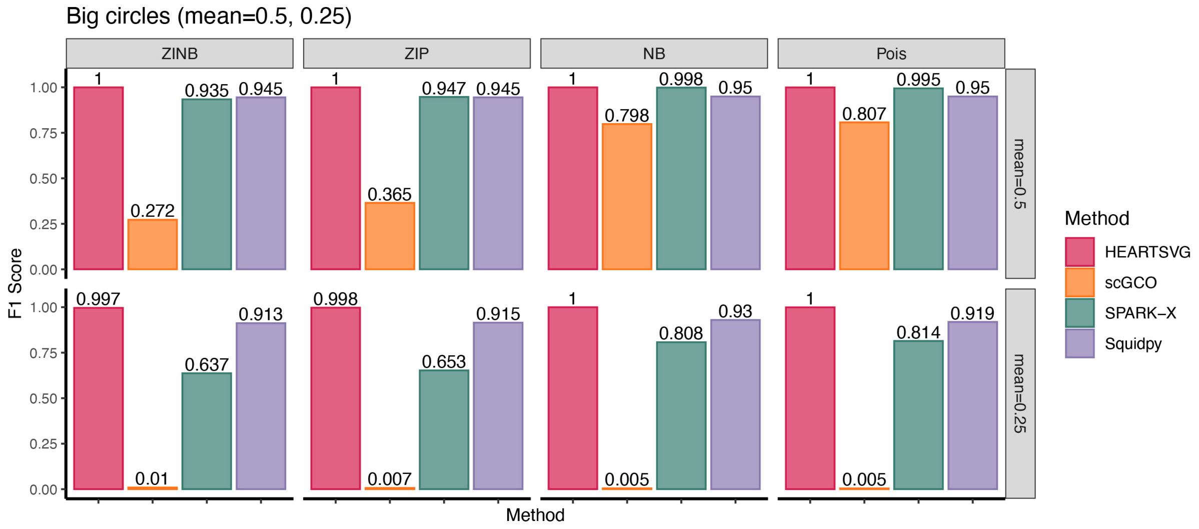


**Figure S91** Each method has similar $F_1$ *score* across different simulated dataset of Big circles Pattern. These datasets generated by different distributions sharing similar data characteristics (mean, dispersion, and sparsity level). Source data are provided with this paper.

**The parameter settings for the two new simulations are as follows.**

1) Simulations with medium sparsity and high expression levels.

For non-SVGs and the non-marked area of SVGs, the data characteristics (mean, dispersion, and sparsity level) generated by different distributions were adjusted to be close to a $mean = var = 0.5,\ P(X = 0) = 0.6$ (approximating $Pois(\lambda = 0.5)$). For the marked area of SVGs, the data characteristics were made to approximate a $mean = var = 1.5, P(X = 0) = 0.3$ (approximating $Pois(\lambda = 1.5)$). The specific parameters are as follows.

**Table S6 Parameters of different distributions close to a $mean = var = 0.5, P(X = 0) = 0.6$ and $mean = var = 1.5, P(X = 0) = 0.3$.**

| | non-SVG and non-marked area of SVGs | | | marked area of SVGs | | |
|---|---|---|---|---|---|---|
| | probability of extra zeros | non-zero part | | probability of extra zeros | non-zero part | |
| | | mu/lambda | size | | mu/lambda | size |
| Pois | - | 0.5 | - | - | 1.5 | |
| NB | - | 0.5 | 30 | - | 1.5 | 30 |
| ZIP | 0.4 | 0.833 | - | 0.2 | 1.8 | - |
| ZINB | 0.5 | 1 | 30 | 0.25 | 2 | 30 |

2) Simulations with high sparsity and low expression levels

For non-SVGs and the non-marked area of SVGs, the data characteristics (mean, dispersion, and sparsity level) generated by different distributions were adjusted to be close to a *mean=var=0.25,* P(X=0)=0.8 (approximating $Pois(\lambda = 0.25)$). For the marked area of SVGs, the data characteristics were made to approximate a *mean=var=0.75*, P(X=0)=0.5 (approximating $Pois(\lambda = 0.75)$). The specific parameters are as follows.

**Table S7 Parameters of different distributions close to a *mean=var=0.5,* P(X=0)=0.6 and *mean=var=1.5*, P(X=0)=0.3.**

| | non-SVG and non-marked area of SVGs | | | marked area of SVGs | | |
|---|---|---|---|---|---|---|
| | probability of extra zeros | non-zero part | | probability of extra zeros | non-zero part | |
| | | mu/lambda | size | | mu/lambda | size |
| Pois | - | 0.25 | - | - | 0.75 | |
| NB | - | 0.25 | 30 | - | 0.75 | 30 |
| ZIP | 0.5 | 0.5 | - | 0.2 | 0.95 | - |
| ZINB | 0.5 | 0.5 | 30 | 0.2 | 0.95 | 30 |

## 11.2. Effect of Data Dispersion on Method Performance

**We found that scGCO is significantly impacted by increased data dispersion, whereas HEARTSVG, SPARK-X, and Squidpy are robust under these conditions.**

In simulations of our manuscript, using $NB$ and $Pois$ distributions, HEARTSVG, SPARK-X, and Squidpy showed similar performances across both sets of simulated data. However, scGCO showed notable differences, performing significantly worse on the NB distribution than on the Pois distribution. The simulations with $NB(1.5,0.5)$ and $Pois(0.5)$ distributions had similar spatial expression patterns (Fig. S4a), equal means (Non-SVG: both $NB$ and $Pois$ with $\mu = 0.5$; SVG: both $NB$ and $Pois$ with $\mu = 1.5$) and similar sparsity levels (Fig. S4b). Yet, the $NB$ $(size = 1.5)$ distribution is more right-skewed, indicating stronger overdispersion (Fig. S92b).

As we know, with the '$size$' parameter in the $NB$ distribution increases, the data dispersion decreases. When '$size$' approaches infinity, the $NB(size, \mu)$ converges to a Poisson distribution $Pois(\lambda)$ with λ=μ. Fig-S1c demonstrated that, the $NB(size = 30)$ and Pois distributions' shapes are almost identical ( $\lambda = \mu$) (Fig. S92c). Therefore, we generated two sets of $NB$ simulation data with $size = 30$ and $size = 5$ (the $\mu$ parameter same as before) to compare with the previous $NB(1.5,0.5)$ and $Pois(0.5)$ distribution results. The simulation results (Fig. S93) showed that with $NB(size = 30)$, as dispersion decreases, scGCO's $F_1\,score$ significantly improves (Fig. S5b), aligning with the $F_1\,score$ seen with the Poisson distribution (Fig. S93c). We conducted similar simulations on the 'Big squares' pattern and obtained consistent results (Fig. S94). Compared to $NB(size = 30)$, $NB(size = 5)$, $NB(size = 1.5)$, with a reduced '$size$' leads to increased dispersion, significantly diminishing scGCO's ability to identify SVGs, while HEARTSVG, SPARK-X, and Squidpy showed no significant change, demonstrating greater robustness.

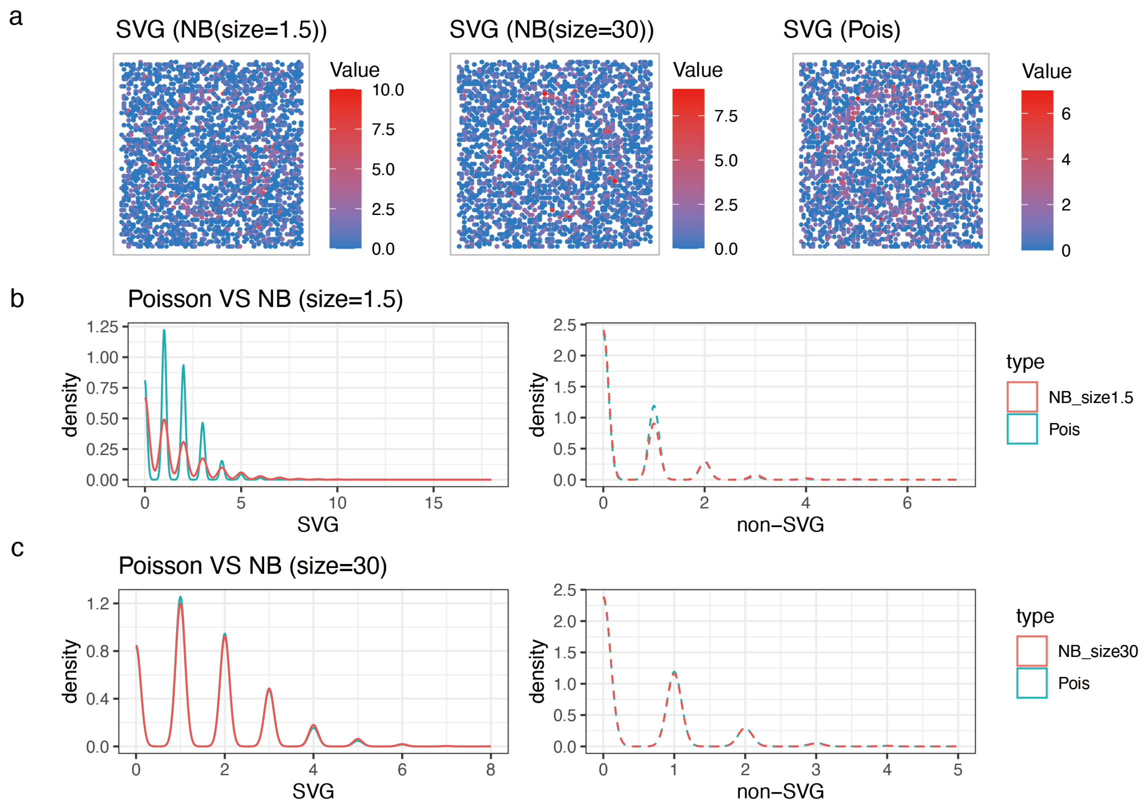


**Figure S92** **a,**Visualizations of the 'Ring pattern' SVGs. Gene expression distributions correspond to $NB(size = 1.5)$, $NB(size = 30)$, and Poisson distribution, with same 'mean' parameter. Their visual appearances are fundamentally similar. **b,** Density comparision of Pois and $NB(size = 1.5)$. $NB\ (size = 1.5)$ distribution is more right-skewed, indicating stronger overdispersion. **c,** Density comparision of Pois and $NB(size = 30)$. $NB(size = 30)$ and Pois distributions' shapes are almost identical.

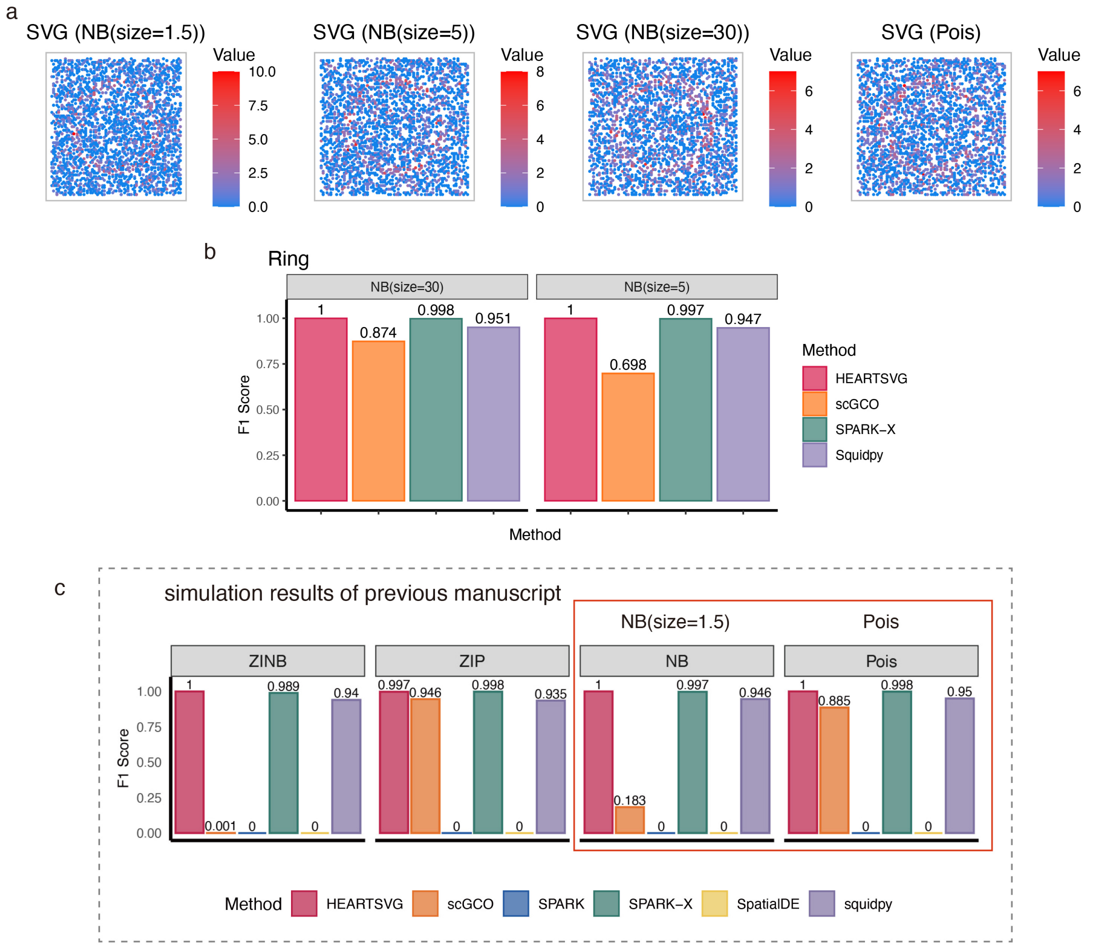

**Figure S93** **a,**Visualizations of the 'Ring pattern' SVGs. Gene expression distributions correspond to $NB(size = 1.5)$, $NB(size = 5)$, $NB(size = 30)$, and Poisson distribution, with same 'mean' parameter. Their visual appearances are similar. **b,** $F_1 score$ comparision of all methods on simulations using $NB(size = 5)$ and $NB(size = 30)$. **c,** $F_1 score$ comparision of all methods on simulations of previous manuscript. The SVG identification capability of scGCO diminished with high dispersion (small 'size' parameter). Source data are provided with this paper.

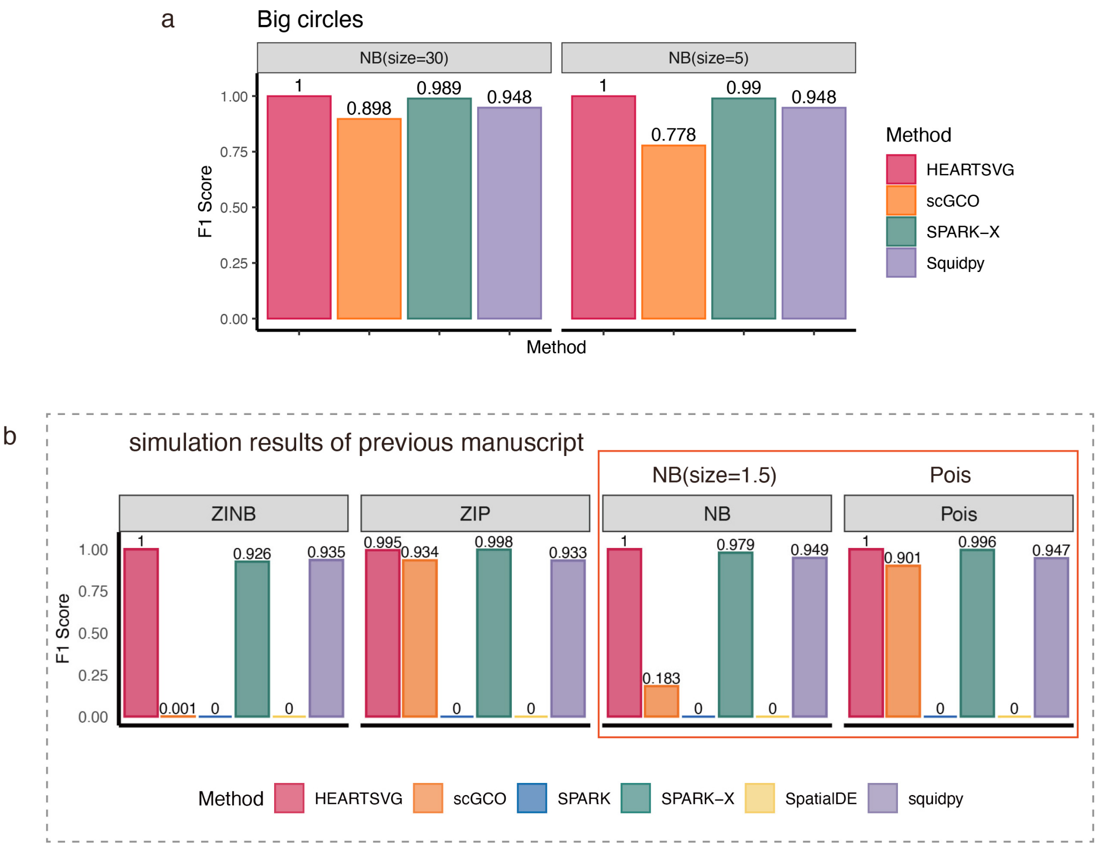


**Figure S94** Big circles Pattern. **a,** $F_1 score$ comparision of all methods on simulations using $NB(size = 5)$ and $NB(size = 30)$. **b,** $F_1 score$ comparision of all methods on simulations of previous manuscript. The SVG identification capability of scGCO diminished with high dispersion (small '$size$' parameter). Source data are provided with this paper.

## 11.3. Impact of Data Sparsity and Expression Levels on SVG Identification

**An increase in data sparsity and a decrease in overall expression levels generally diminishes all methods' capacities to identify SVGs.**

High sparsity often coexists with low expression levels in single-cell and spatial transcriptomics data. In simulations of our previous manuscript, we generated simulated scenarios with high sparsity and low expression levels using the $ZINB$ distribution. In these simulations, the non-SVGs had over 94% zeros, while the SVGs had over 60% zeros. High sparsity is common in data generated by techniques like Slide-seqV2, HDST, Visium HD and Stereo-seq at single-cell or subcellular resolution.

To further investigate this, we introduced a new $NB$ distribution with parameters (SVG: $NB(size = 0.5, \mu = 0.73)$, non-SVG: $NB(size = 0.065, \mu = 0.1)$), aiming to approximate the original $ZINB$ distribution (Fig. S95a). We generated simulated data

following this new $NB$ distribution and compared it with the previous $ZINB$ simulation results. The results (Fig. S95b) show that all methods performed on the new $NB$ simulated data that were generally consistent with the original $ZINB$ results. The comparison of the new $NB$ simulation with the original $NB$ results demonstrated that all methods' SVG identification capabilities decreased on the more sparsely distributed new $NB$ data.

Additionally, we conducted another simulation (Tab.S3). Using Poisson distributions with decreasing $\lambda$ values: ($Pois(\lambda = 0.5)$, $Pois(\lambda = 0.25)$, $Pois(\lambda = 0.1)$. As we know, for Poisson distribution, as the parameter $\lambda$ decreased, the data sparsity increased, and overall expression levels decreased. Similar to the previous simulation, all methods exhibited decreased SVG identification capabilities as λ decreased. HEARTSVG and Squidpy showed greater robustness to changes in sparsity than others.

**Table S8 new Poisson distributions parameters.**

| | | non-SVG and non-marked area of SVGs | marked area of SVGs |
|---|---|---|---|
| Pois (lambda) | $Pois(\lambda = 0.1)$ | 0.1 | 0.3 |
| | $Pois(\lambda = 0.25)$ | 0.25 | 0.75 |
| | $Pois(\lambda = 0.5)$ | 0.5 | 1.5 |

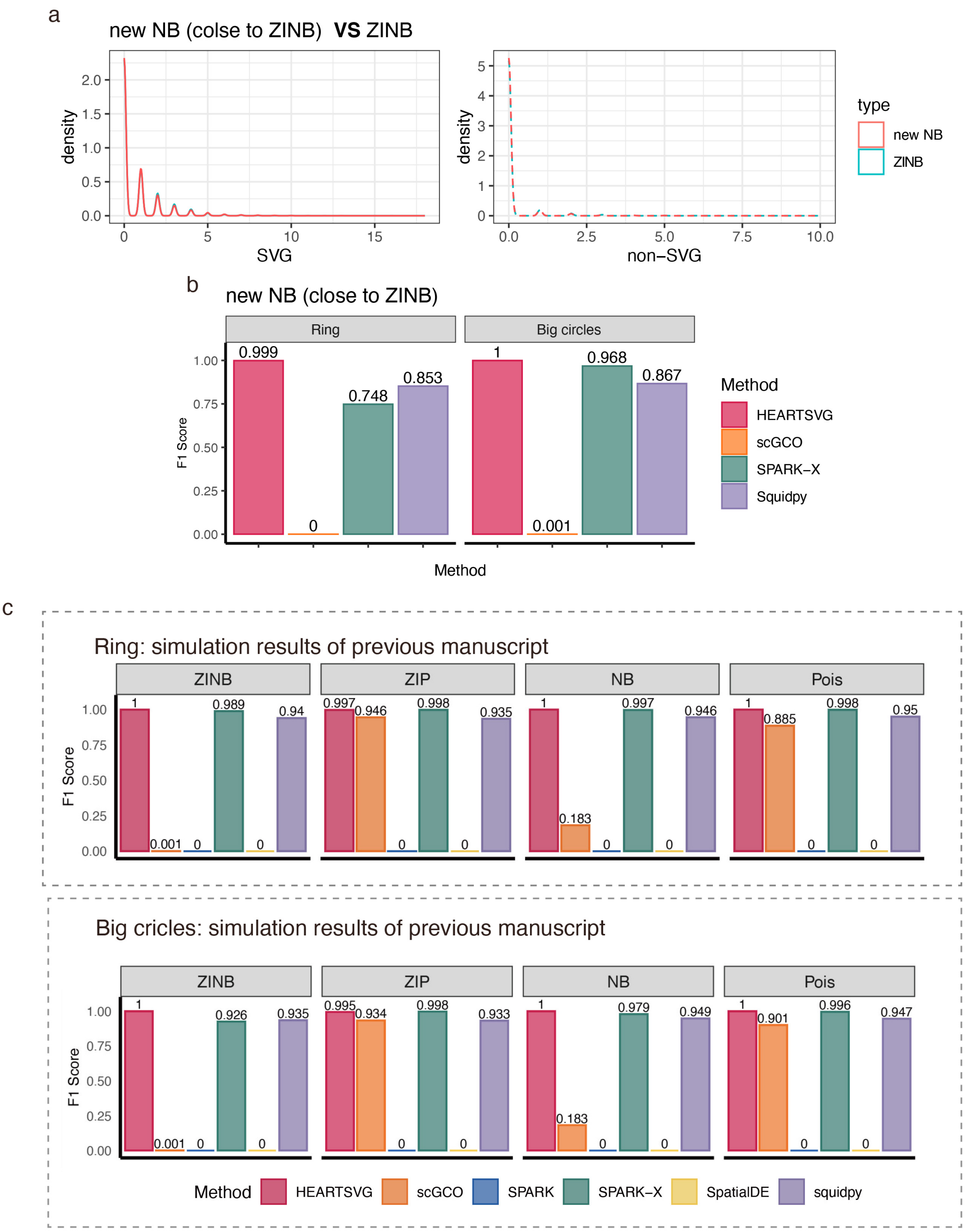


**Figure S95** **a,** Density comparision of new $NB$ and original $ZINB$. Their distributions' shapes are almost identical. **b,** $F_1 score$ comparision of all methods on simulated data from new $NB$ distributions. **c,** $F_1 score$ comparision of all methods on simulations of previous manuscript.

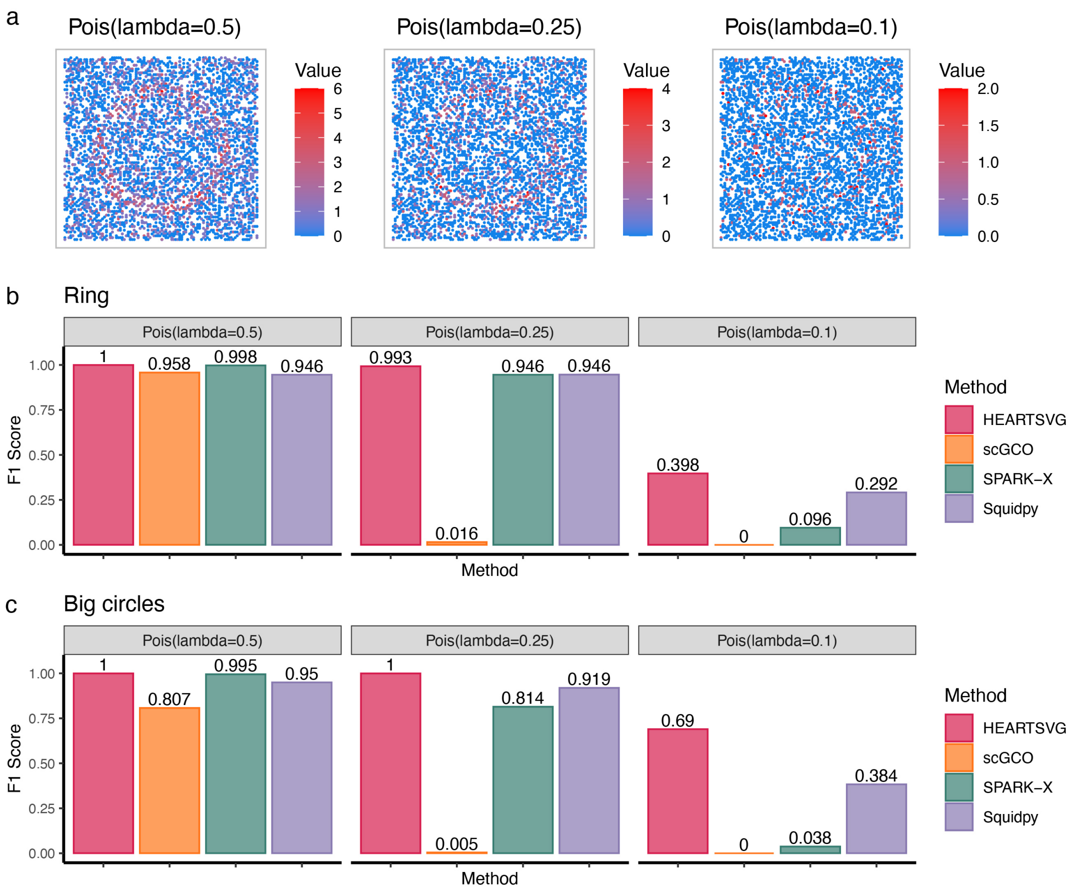


**Figure S96** **a,** Visualizations of the 'Ring pattern' SVGs. Gene expression distributions correspond to ($Pois(\lambda = 0.5)$, $Pois(\lambda = 0.25)$, $Pois(\lambda = 0.1)$. As the paraneter $\lambda$ decreased, the data sparsity increased, overall expression levels decreased, and visual clarity diminished. The color pattern distribution across the plots gets progressively sparser from left to right, illustrating the effect of decreasing the lambda parameter on the sparsity of the generated data. **b,** Ring Pattern, comparing the $F_1$ *scores* of four different methods across three simulated scenarios with varying $\lambda$ for Poisson distributions: 0.5, 0.25, and 0.1. **c,** Big circles Pattern, comparing the $F_1$ *scores* of four different methods across three simulated scenarios with varying $\lambda$ for Poisson distributions: 0.5, 0.25, and 0.1. The bar chart clearly visualizes the decreasing $F_1$ *scores* of four methods as data sparsity increases with decreasing $\lambda$ values. Source data are provided with this paper.

### 11.4. Uniform Hindrance: Low Cell/Spot Counts

A low count of cells or spots uniformly hinders all methods' abilities to identify SVGs effectively. Although unrelated to the data distribution and data characteristics, our research indeed found that the capability of all methods to identify SVGs diminishes when the dataset contains a smaller number of cells/spots. We generated a new simulations with the same Poisson distribution parameters as the previous simulation in the manuscript, but with the number of cells set to 500. Compared to the previous simulation results, the new simulation showed a marked decrease in the $F_1$ *scores* for all methods (Fig. S97).

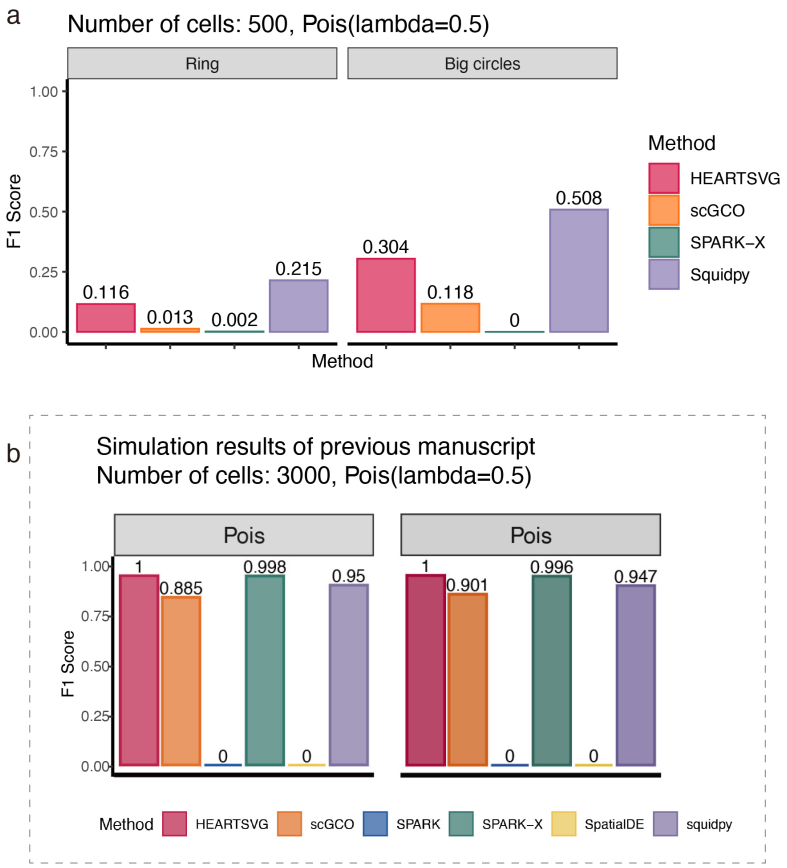


**Figure S97** **a,** New simulation results with the number of cells set to 500. **b,** Previous simulation results with the number of cells set to 3000. Source data are provided with this paper.

# 12. Comparison of average false discovery proportion (FDP)

We evaluated the average False Discovery Proportion (FDP) against the nominal False Discovery Rate (FDR) using noise-free simulated data at mean levels of 0.5 and 0.25 (Page 105，Table S6-S7 of this file for detailed simulation settings). We examined the average FDP of various methods at nominal FDR settings of 0.01, 0.05, and 0.1. Our findings (Fig. S98) indicate that Squidpy's average FDP consistently exceeded the nominal FDR. In contrast, HEARTSVG and SPARK-X maintained an average FDP below the nominal FDR consistently. For scGCO, the average FDP surpassed the nominal FDR at the mean level of 0.5 simulated data with moderate data sparsity. However, at the lower expression level with higher sparsity (mean level of 0.25), scGCO's average FDP fell below the nominal FDR, albeit with a concurrently low TPR. These results highlight the superior performance of HEARTSVG and SPARK-X in controlling false positives.

We noted that the literature[5] suggests "Simulation experiments relying on parametric models may offer an overly optimistic assessment of a method's efficacy". Given the inability to generate spatial transcriptomics simulated data for SVGs using the SimSeq algorithm, we embarked on an alternative intriguing endeavor. We analyzed the changes of average FDP as the capacity to identify SVGs deteriorated (reflected by a decrease in $F_1\ score$). Specifically, we modified the approach of adding Gaussian noise as Reviewer 3's comment. With this modified approach to Gaussian noise incorporation, we observed that as the level of noise increased, all patterns became increasingly difficult to detect (Fig. S99a), causing a decline in the $F_1\ scores$ of all methods towards zero (Fig. S99b). At a nominal FDR of 0.05, we analyzed how the average FDP of various methods altered with increasing noise. Our results (Fig. S99b) indicated that Squidpy's average FDP consistently exceeded the nominal FDR. In contrast, HEARTSVG and SPARK-X's average FDP remained below the nominal FDR. For scGCO, the average FDP began to rise with increasing noise levels and ultimately surpassed the nominal FDR.

In summary, HEARTSVG stands out in controlling false positives, indicating that its identified SVGs are highly credible. However, this may also suggest that HEARTSVG's selection is relatively conservative.

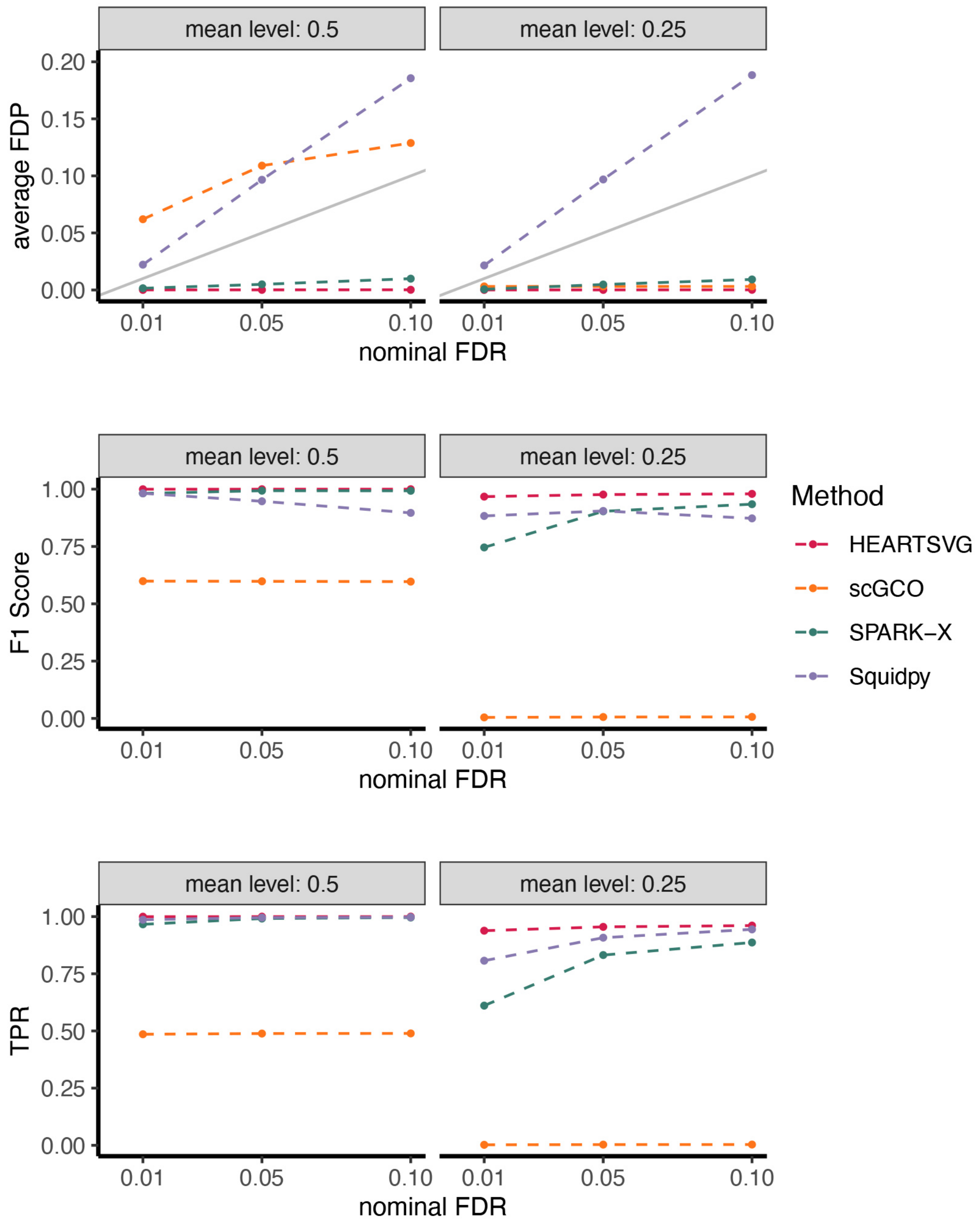


**Figure S98 a,** Plot of average FDP at different nominal FDR. The solid gray line represents an average FDP that is exactly equal to nominal FDR. **b,** Plot of average $F_1\ score$ at different nominal FDR. **c,** Plot of average TPR at different nominal FDR. Source data are provided with this paper.

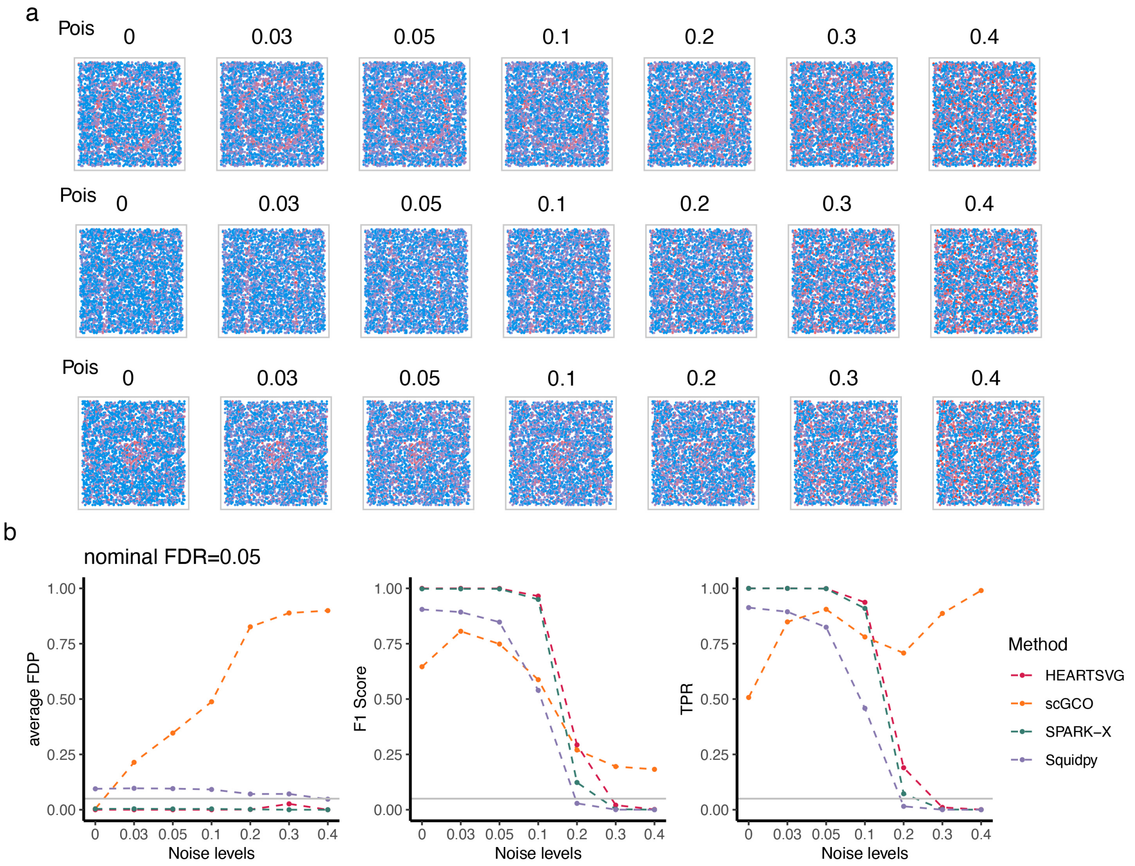


**Figure S99** **a,** Visualizations of spatial patterns, incorporating noise using the modified Gaussian noise addition approach. **b,** Plot of average FDP, $F_1\ score$ and TPR at nominal FDR=0.05. The solid gray line represents nominal FDR=0.05. Source data are provided with this paper.

# Supplementary References


1. Sun, S., Zhu, J. & Zhou, X. Statistical analysis of spatial expression patterns for spatially resolved transcriptomic studies. *Nat. Methods* **17**, 193–200 (2020).
2. Shah, S., Lubeck, E., Zhou, W. & Cai, L. In Situ Transcription Profiling of Single Cells Reveals Spatial Organization of Cells in the Mouse Hippocampus. *Neuron* **92**, 342–357 (2016).
3. Zhu, J., Sun, S. & Zhou, X. SPARK-X: non-parametric modeling enables scalable and robust detection of spatial expression patterns for large spatial transcriptomic studies. *Genome Biol.* **22**, 184 (2021).
4. Hu, J. *et al.* SpaGCN: Integrating gene expression, spatial location and histology to identify spatial domains and spatially variable genes by graph convolutional network. *Nat. Methods* **18**, 1342–1351 (2021).
5. Benidt, S. & Nettleton, D. SimSeq: a nonparametric approach to simulation of RNA-sequence datasets. *Bioinformatics* **31**, 2131–2140 (2015).
