# Supplementary material for: HEARTSVG: a fast and accurate method for identifying spatially variable genes in large-scale spatial transcriptomics
Source: Nat Commun. 2024 Jul 7;15:5700. doi: 10.1038/s41467-024-49846-1 (PMC11228050; doi:10.1038/s41467-024-49846-1)
Supplement: Supplementary file 2 — Reporting Summary [file 41467_2024_49846_MOESM2_ESM.pdf]

nature portfolio

Corresponding author(s): Zhangsheng Yu

Last updated by author(s): Jun 3, 2024

Reporting Summary

Nature Portfolio wishes to improve the reproducibility of the work that we publish. This form provides structure for consistency and transparency in reporting. For further information on Nature Portfolio policies, see our Editorial Policies and the Editorial Policy Checklist.

Statistics

For all statistical analyses, confirm that the following items are present in the figure legend, table legend, main text, or Methods section.

n/a	Confirmed	
☐	☒	The exact sample size (*n*) for each experimental group/condition, given as a discrete number and unit of measurement
☒	☐	A statement on whether measurements were taken from distinct samples or whether the same sample was measured repeatedly
☐	☒	The statistical test(s) used AND whether they are one- or two-sided<br>*Only common tests should be described solely by name; describe more complex techniques in the Methods section.*
☒	☐	A description of all covariates tested
☐	☒	A description of any assumptions or corrections, such as tests of normality and adjustment for multiple comparisons
☐	☒	A full description of the statistical parameters including central tendency (e.g. means) or other basic estimates (e.g. regression coefficient) AND variation (e.g. standard deviation) or associated estimates of uncertainty (e.g. confidence intervals)
☐	☒	For null hypothesis testing, the test statistic (e.g. *F*, *t*, *r*) with confidence intervals, effect sizes, degrees of freedom and *P* value noted<br>*Give P values as exact values whenever suitable.*
☒	☐	For Bayesian analysis, information on the choice of priors and Markov chain Monte Carlo settings
☒	☐	For hierarchical and complex designs, identification of the appropriate level for tests and full reporting of outcomes
☒	☐	Estimates of effect sizes (e.g. Cohen's *d*, Pearson's *r*), indicating how they were calculated

Our web collection on statistics for biologists contains articles on many of the points above.

Software and code

Policy information about availability of computer code

Data collection	No software was used for data collection.
Data analysis	HEARTSVG R package (version 1.1.0) is deposited in the GitHub （https://github.com/cz0316/HEARTSVG.git); The codes for analysis are deposited at https://github.com/cz0316/HEARTSVG.git. Other published algorithms, use to evaluate the performance of HEARTSVG are accessible from their respective GitHub repositories. These algorithms encompass SpatialDE (version 1.0.0, https://github.com/sales-lab/spatialDE), SPARK (version 1.1.1, https://github.com/xzhoulab/SPARK), and SPARK-X (version 1.1.1, https://github.com/xzhoulab/SPARK).<br><br>In addition, we also used the following software packages for data analysis:Seurat (https://github.com/satijalab/seurat, R package v4.0.1) sc-MEB(https://github.com/Shufeyangyi2015310117, R package V1.1),gprofiler2(https://cran.r-project.org/src/contrib/gprofiler2_0.2.3.tar.gz, R package V 0.2.3)

For manuscripts utilizing custom algorithms or software that are central to the research but not yet described in published literature, software must be made available to editors and reviewers. We strongly encourage code deposition in a community repository (e.g. GitHub). See the Nature Portfolio guidelines for submitting code & software for further information.

Data

Policy information about availability of data

All manuscripts must include a data availability statement. This statement should provide the following information, where applicable:

- Accession codes, unique identifiers, or web links for publicly available datasets
- A description of any restrictions on data availability
- For clinical datasets or third party data, please ensure that the statement adheres to our policy

All data analyzed in this manuscript are available in their raw form from the respective original authors. (1) The 10X Visium data of colorectal cancer are available at the Single-Cell Colorectal Cancer Liver Metastases (CRLM) Atlas (http://www.cancerdiversity.asia/scCRLM); (2) The Slide-seqV2 data are available at the Single Cell Portal (https://singlecell.broadinstitute.org/single_cell/study/SCP815); (3) The MERFISH datasets are available in the Dryad Digital Repository from https://doi.org/10.5061/dryad.8t8s248; (4) The mouse olfactory bulb data generated by high-definition spatial transcriptomics (HDST) are available at the NCBI Gene Expression Omnibus (GEO) database repository under accession code GSE130682; (5) The 10X Visium data of primary liver cancer are available at the Genome Sequence Archive (GSA) under accession code HRA000437; (6) The 10X Visium data of renal clear cell cancer brain metastasis are available at the NCBI Gene Expression Omnibus (GEO) database repository under accession code GSE179572. Source data are provided with this paper.

Research involving human participants, their data, or biological material

Policy information about studies with human participants or human data. See also policy information about sex, gender (identity/presentation), and sexual orientation and race, ethnicity and racism.

Reporting on sex and gender	This study's scope and objectives did not necessitate the inclusion of sex- and gender-based analyses. HEARTSVG primarily focuses on the development of the method for spatially variable gene identification in large-scale spatial transcriptomic data. As a result, the study does not directly involve reporting on sex and gender-related analyses or considerations. Therefore, information regarding sex and gender, including disaggregated data and consent for individual-level data sharing, has not been collected for this particular research.
Reporting on race, ethnicity, or other socially relevant groupings	In our study, we did not utilize any socially constructed or socially relevant categorization variables such as race, ethnicity, or other groupings.
Population characteristics	As a methodological research article, HEARTSVG aims to identify spatially variable genes in large-scale spatial transcriptomic data. This study did not collect population characteristics, such as age, genotypic information, diagnosis, or treatment categories.
Recruitment	In this study, the data used for analysis in HEARTSVG were publicly available datasets. As a methodological research article, we did not conduct participant recruitment or involve human subjects.
Ethics oversight	Ethics oversight and approval were not applicable to our research. This study did not conduct any experiments or studies that required ethics approval. All datasets used in this study were publicly available.

Note that full information on the approval of the study protocol must also be provided in the manuscript.

Field-specific reporting

Please select the one below that is the best fit for your research. If you are not sure, read the appropriate sections before making your selection.

[x] Life sciences [ ] Behavioural & social sciences [ ] Ecological, evolutionary & environmental sciences

For a reference copy of the document with all sections, see nature.com/documents/nr-reporting-summary-flat.pdf

Life sciences study design

All studies must disclose on these points even when the disclosure is negative.

Sample size	In this study, no formal sample size calculation was performed as the concept of sample size is not applicable to the methodology used in HEARTSVG. HEARTSVG is a computational method for spatially variable gene identification in spatial transcriptomic data, and the analysis is not based on traditional sample sizes but rather on the number of spatial spots and genes within the dataset. The sample sizes in the spatial transcriptomic datasets used were chosen to ensure comprehensive coverage of the spatial gene expression patterns and to provide sufficient information for the analysis.
Data exclusions	In this study, we excluded cells with no gene expression and genes with expression rates below 1% to satisfy computational requirements.
Replication	Replication is not applicable to our study. HEARTSVG focuses on the identification of spatially variable gene or spatial transcriptomic data analysis. As such, there are no experimental findings to be replicated or reproduced. The reproducibility in this context pertains to the computational implementation of the HEARTSVG method, which can be verified by providing the source code, algorithms, and detailed descriptions in the manuscript.
Randomization	Randomization is not applicable to our study. HEARTSVG is a computational method for spatially variable gene identification in spatial transcriptomic data, not involving experimental treatments or interventions.

Blinding	Blinding is not applicable to our study. Due to the nature of the identification of the spatially variable gene, blinding was not relevant to our study.

Reporting for specific materials, systems and methods

We require information from authors about some types of materials, experimental systems and methods used in many studies. Here, indicate whether each material, system or method listed is relevant to your study. If you are not sure if a list item applies to your research, read the appropriate section before selecting a response.

Materials & experimental systems

n/a	Involved in the study
☒	☐ Antibodies
☒	☐ Eukaryotic cell lines
☒	☐ Palaeontology and archaeology
☒	☐ Animals and other organisms
☒	☐ Clinical data
☒	☐ Dual use research of concern
☒	☐ Plants

Methods

n/a	Involved in the study
☒	☐ ChIP-seq
☒	☐ Flow cytometry
☒	☐ MRI-based neuroimaging
